# Supplementary material for: ATP synthase interactome analysis identifies a new subunit l as a modulator of permeability transition pore in yeast
Source: Sci Rep. 2023 Mar 7;13:3839. doi: 10.1038/s41598-023-30966-5 (PMC9992712; doi:10.1038/s41598-023-30966-5)
Supplement: Supplementary file 1 — Supplementary Information 1. [file 41598_2023_30966_MOESM1_ESM.pdf]

Sequence coverage (percent)	Accession	Mol wt	Peptides identified	Description	emPAI	BN_1	BN_2	BN_3	Pull_Rep 1	Pull_Rep 2	Pull_Rep 3	Only_Dimer	Only_monomer
0,125	Q0080	5818	6	Subunit 8 of the F0 sector of mitochondrial F1F0 ATP synthase; encoded on the mitochondrial genome; ATP8 and ATP6 mRNAs are not translated in the absence of the F1 sector of ATPase	5,94	0	0	0	1	1	0	0	0
0,71	YML081C-A	6683	93	Subunit of the mitochondrial F1F0 ATP synthase; F1F0 ATP synthase is a large, evolutionarily conserved enzyme complex required for ATP synthesis; termed subunit I or subunit j; does not correspond to known ATP synthase subunits in other organisms	54,86	1	1	0	1	0	1	0	0
0,92	YPL271W	6738	342	Epsilon subunit of the F1 sector of mitochondrial F1F0 ATP synthase; which is a large, evolutionarily conserved enzyme complex required for ATP synthesis; F1 translationally regulates ATP6 and ATP8 expression to achieve a balanced output of ATP synt	1524,25	1	1	0	1	1	1	0	0
0,25	YNL070W	6866	8	Component of the TOM (translocase of outer membrane) complex; responsible for recognition and initial import steps for all mitochondrially directed proteins; promotes assembly and stability of the TOM complex	2,21	0	1	0	0	0	0	0	0
0,32	YLR388W	6884	8	Protein component of the small (40S) ribosomal subunit; homologous to mammalian ribosomal protein S29 and bacterial S14; RPS29A has a paralog, RPS29B, that arose from the whole genome duplication	1,84	1	0	0	0	0	1	0	0
0,24	YBL039W-B	6914	2	Putative protein of unknown function	0,79	0	1	0	0	0	0	0	0
0,2	YMR256C	6928	16	Subunit VII of cytochrome c oxidase (Complex IV); Complex IV is the terminal member of the mitochondrial inner membrane electron transport chain	2,05	1	1	0	1	1	1	0	0
0,373	YDL067C	6959	97	Subunit VIIa of cytochrome c oxidase (Complex IV); Complex IV is the terminal member of the mitochondrial inner membrane electron transport chain	121,00	1	1	0	1	1	1	0	0
0,11	YBR162W-A	7360	2	Protein of unknown function; expression suppresses a secretory pathway mutation in E. coli; has similarity to the mammalian RAMP4 protein involved in secretion	0,50	1	0	0	1	0	0	0	1
0,364	YGR183C	7472	10	Subunit 9 of ubiquinol cytochrome-c reductase (Complex III); Complex III is a component of the mitochondrial inner membrane electron transport chain; required for electron transfer at the ubiquinol oxidase site of the complex	2,31	1	0	0	1	0	0	0	0
0,17	YDR119W-A	7504	17	Putative protein of unknown function; copurifies with respiratory chain supercomplexes composed of Complex III (ubiquinol-cytochrome c reductase) and Complex IV (cytochrome c oxidase)	0,96	1	1	0	1	0	0	0	0
0,81	YOL077W-A	7529	85	Subunit k of the mitochondrial F1F0 ATP synthase; F1F0 ATP synthase is a large, evolutionarily conserved enzyme complex required for ATP synthesis; associated only with the dimeric form of ATP synthase	31,90	1	1	0	1	1	1	0	0
0,42	YOR167C	7587	12	Protein component of the small (40S) ribosomal subunit; has an extraribosomal function in regulation of RPS28B, in which Rps28Ap binds to a decapping complex via Edc3p, which then binds to RPS28B mRNA leading to its decapping and degradation; homolo	4,33	1	0	0	0	0	0	0	0
0,1	YIR021W-A	7732	6	Putative protein of unknown function; identified by expression profiling and mass spectrometry	1,15	1	0	1	1	0	1	1	0
0,11	Q0130	7800	23	F0-ATP synthase subunit c (ATPase-associated proteolipid); encoded on the mitochondrial genome; mutation confers oligomycin resistance; expression is specifically dependent on the nuclear genes AEP1 and AEP2	2,31	0	1	0	1	1	0	0	0
0,33	YPR010C-A	7943	10	Putative protein of unknown function; conserved among Saccharomyces sensu stricto species	1,98	0	1	0	1	1	1	0	1
0,5	YML129C	7954	12	Mitochondrial cytochrome c oxidase (complex IV) assembly factor; also involved in translational regulation of Cox1p and prevention of Cox1p aggregation before assembly; associates with complex IV assembly intermediates and complex III/complex IV sup	6,29	1	1	0	1	0	1	1	0
0,13	YML009C	7968	3	Mitochondrial ribosomal protein of the large subunit	0,50	1	1	0	0	0	0	0	0
0,14	YIL156W-B	8273	6	Putative protein of unknown function; originally identified based on homology to <i>Ashbya gossypii</i> and other related yeasts	0,49	1	1	0	0	0	0	0	0
0,15	YKL023C-A	8514	2	Putative protein of unknown function	0,41	0	1	0	1	0	0	1	0
0,21	YKL065W-A	8526	12	Putative protein of unknown function	1,58	0	1	0	0	0	0	0	0
0,151	YBR298C-A	8576	11	Putative protein of unknown function; identified by gene-trapping, microarray-based expression analysis, and genome-wide homology searching	0,61	0	0	1	0	0	0	0	0
0,56	YHR001W-A	8587	68	Subunit of the ubiqunol-cytochrome c oxidoreductase complex; this complex comprises part of the mitochondrial respiratory chain; members include Cobp, Rip1p, Cyt1p, Cor1p, Qcr2p, Qcr6p, Qcr7p, Qcr8p, Qcr9p, and Qcr10p and comprises part of the mitoc	12,82	1	1	0	1	0	1	0	0
0,12	YER087C-B	8706	4	Beta subunit of Sec61p ER translocation complex (Sec61p-Sss1p-Sbh1p); involved in protein translocation into the endoplasmic reticulum; interacts with the exocyst complex and also with Rtn1p; cotranslationally N-acetylated by NatA; SBH1 has a paralo	0,51	1	1	0	1	0	0	0	0
0,41	YLR325C	8821	20	Ribosomal 60S subunit protein L38; homologous to mammalian ribosomal protein L38, no bacterial homolog	2,67	1	0	0	0	1	1	0	0

0,125	YDR086C	8995	10	Subunit of the Sec61p translocation complex (Sec61p-Sss1p-Sbh1p); this complex forms a channel for passage of secretory proteins through the endoplasmic reticulum membrane, and of the Ssh1p complex (Ssh1p-Sbh2p-Sss1p); interacts with Ost4p and Wbp1p	1,17	1	1	1	1	0	1	0	0
0,18	YLR395C	9015	27	Subunit VIII of cytochrome c oxidase (Complex IV); Complex IV is the terminal member of the mitochondrial inner membrane electron transport chain	1,27	1	1	0	1	1	1	0	0
0,38	YHR021C	9145	17	Protein component of the small (40S) ribosomal subunit; homologous to mammalian ribosomal protein S27, no bacterial homolog; RPS27B has a paralog, RPS27A, that arose from the whole genome duplication	3,18	1	0	0	1	0	1	0	0
0,34	YDR379C-A	9346	12	Mitochondrial protein involved in assembly of succinate dehydrogenase; has a role in maturation of the Sdh2p subunit; member of the LYR protein family; mutations in human ortholog SDHAF1 are associated with infantile leukoencephalopathy	1,73	0	1	0	0	0	1	0	0
0,27	YER074W-A	9430	4	Integral membrane protein required for ER to Golgi transport; localized to the Golgi, the ER, and COPII vesicles; interacts with Yip1p and Yif1p	1,91	0	0	0	0	1	1	0	0
0,24	YMR286W	9525	4	Mitochondrial ribosomal protein of the large subunit	0,80	0	1	0	0	0	1	0	0
0,23	YGL226C-A	9557	6	Zeta subunit of the oligosaccharyltransferase complex of the ER lumen; complex catalyzes asparagine-linked glycosylation of newly synthesized proteins	0,68	0	1	0	0	0	0	1	0
0,26	YER019C-A	9600	12	Ssh1p-Sss1p-Sbh2p complex component; involved in protein translocation into the endoplasmic reticulum; SBH2 has a paralog, SBH1, that arose from the whole genome duplication	1,31	1	1	0	1	1	1	0	0
0,567	YOR020W-A	9612	57	Putative protein of unknown function; conserved in A. gossypii; the authentic, non-tagged protein is detected in highly purified mitochondria in high-throughput studies	10,71	1	1	1	1	0	1	0	0
0,53	YDL181W	9864	53	Protein that inhibits ATP hydrolysis by the F1F0-ATP synthase; inhibitory function is enhanced by stabilizing proteins Stf1p and Stf2p; has a calmodulin-binding motif and binds calmodulin in vitro; INH1 has a paralog, STF1, that arose from the whole	9,33	1	1	0	1	1	1	0	0
0,34	YJL062W-A	9875	15	Mitochondrial protein required for cytochrome c oxidase assembly; also involved in translational regulation of Cox1p and prevention of Cox1p aggregation before assembly; located in the mitochondrial inner membrane	1,11	0	1	0	1	1	1	0	0
0,313	YLR038C	9965	12	Subunit VIb of cytochrome c oxidase; cytochrome c oxidase is also known as respiratory Complex IV and is the terminal member of the mitochondrial inner membrane electron transport chain; required for assembly of cytochrome c oxidase but not required	1,57	0	1	0	0	1	1	1	0
0,22	YDL130W-A	10113	3	Protein involved in regulation of the mitochondrial F1F0-ATP synthase; Stf1p and Stf2p act as stabilizing factors that enhance inhibitory action of the Inh1p protein; protein abundance increases in response to DNA replication stress; STF1 has a para	0,26	1	1	0	0	0	0	0	1
0,38	YNR037C	10269	4	Mitochondrial ribosomal protein of the small subunit; has similarity to E. coli S19 ribosomal protein	0,70	1	1	0	0	0	0	0	1
0,18	YPR043W	10314	3	Ribosomal 60S subunit protein L43A; null mutation confers a dominant lethal phenotype; homologous to mammalian ribosomal protein L37A, no bacterial homolog; RPL43A has a paralog, RPL43B, that arose from the whole genome duplication	0,85	0	1	0	0	0	0	0	0
0,31	YEL020W-A	10379	6	Essential protein of the mitochondrial intermembrane space; forms a complex with Tim10p (TIM10 complex) that delivers hydrophobic proteins to the TIM22 complex for insertion into the inner membrane	0,72	0	1	0	0	1	1	0	1
0,43	YCL057C-A	10401	33	Conserved component of the MICOS complex; MICOS (formerly MINOS or MitOS) is a mitochondrial inner membrane complex that extends into the intermembrane space and has a role in the maintenance of crista junctions, inner membrane architecture, and for	1,46	1	1	0	1	0	0	0	0
0,23	YHR005C-A	10482	2	Essential protein of the mitochondrial intermembrane space; forms a complex with Tim9p (TIM10 complex) that delivers hydrophobic proteins to the TIM22 complex for insertion into the inner membrane	0,60	0	1	0	0	0	0	0	1
0,1	YNL211C	10642	7	Putative protein of unknown function; green fluorescent protein (GFP)-fusion protein localizes to mitochondria; YNL211C is not an essential gene	0,47	0	1	0	0	0	0	0	0
0,89	YDR322C-A	10926	288	Subunit e of mitochondrial F1F0-ATPase; ATPase is a large, evolutionarily conserved enzyme complex required for ATP synthesis; essential for the dimeric and oligomeric state of ATP synthase, which in turn determines the shape of inner membrane crist	431,04	1	1	0	1	1	1	0	0
0,65	YJL166W	10967	57	Subunit 8 of ubiquinol cytochrome-c reductase (Complex III); Complex III is a component of the mitochondrial inner membrane electron transport chain; oriented facing the intermembrane space; expression is regulated by Abf1p and Cpf1p	4,46	1	1	0	1	0	0	0	0
0,122	YLR361C-A	11101	3	Putative protein of unknown function	0,45	0	0	0	1	0	0	0	0
0,14	YMR194W	11117	7	Ribosomal 60S subunit protein L36A; N-terminally acetylated; binds to 5.8 S rRNA; homologous to mammalian ribosomal protein L36, no bacterial homolog; RPL36A has a paralog, RPL36B, that arose from the whole genome duplication	0,46	1	1	0	0	0	1	0	0
0,22	YJR085C	11243	2	Protein of unknown function; GFP-fusion protein is induced in response to the DNA-damaging agent MMS; the authentic, non-tagged protein is detected in highly purified mitochondria in high-throughput studies; protein abundance increases in response t	0,44	0	1	0	0	0	0	0	0
0,67	YER048W-A	11259	55	Cysteine desulfurase (Nfs1p) activator; essential for the formation of the persulfide intermediate at the desulfurase active site during pyridoxal phosphate-dependent desulfuration of cysteine; required for mitochondrial iron-sulfur cluster biosynth	18,63	1	1	0	1	0	1	0	0

0,56	YDR377W	11305	352	Subunit f of the F0 sector of mitochondrial F1F0 ATP synthase; F1F0 ATP synthase is a large, evolutionarily conserved enzyme complex required for ATP synthesis	63,02	1	1	1	1	1	1	0	0
0,359	YLR043C	11344	3	Cytoplasmic thioredoxin isoenzyme; part of thioredoxin system which protects cells against oxidative and reductive stress; forms LMA1 complex with Pbi2p; acts as a cofactor for Tsa1p; required for ER-Golgi transport and vacuole inheritance; with Trx	1,97	0	0	0	0	0	1	0	0
0,12	YBR009C	11361	20	Histone H4; core histone protein required for chromatin assembly and chromosome function; one of two identical histone proteins (see also HHF2); contributes to telomeric silencing; N-terminal domain involved in maintaining genomic integrity	1,56	1	1	0	0	0	1	0	0
0,68	YOR020C	11365	60	Mitochondrial matrix co-chaperonin; inhibits the ATPase activity of Hsp60p, a mitochondrial chaperonin; involved in protein folding and sorting in the mitochondria; 10 kD heat shock protein with similarity to E. coli groES	5,06	1	1	0	1	0	0	0	0
0,28	YGL030W	11408	12	Ribosomal 60S subunit protein L30; involved in pre-rRNA processing in the nucleolus; autoregulates splicing of its transcript; homologous to mammalian ribosomal protein L30, no bacterial homolog	1,10	1	0	0	0	0	1	0	0
0,48	YMR225C	11469	16	Mitochondrial ribosomal protein of the large subunit; protein abundance increases in response to DNA replication stress	1,43	0	1	0	1	0	0	0	0
0,31	YGR027C	12032	7	Protein component of the small (40S) ribosomal subunit; homologous to mammalian ribosomal protein S25, no bacterial homolog; RPS25A has a paralog, RPS25B, that arose from the whole genome duplication	0,80	1	1	0	0	0	0	0	0
0,17	YDR115W	12080	6	Putative mitochondrial ribosomal protein of the large subunit; similar to E. coli L34 ribosomal protein; required for respiratory growth, as are most mitochondrial ribosomal proteins; protein increases in abundance and relocalizes to the plasma memb	0,69	1	1	0	0	1	1	0	0
0,24	YPL143W	12147	6	Ribosomal 60S subunit protein L33A; N-terminally acetylated; rpl33a null mutant exhibits slow growth while rpl33a rpl33b double null mutant is inviable; homologous to mammalian ribosomal protein L35A, no bacterial homolog; RPL33A has a paralog, RPL3	0,59	1	1	0	0	0	0	0	0
0,28	YER058W	12319	14	Protein required for assembly of cytochrome c oxidase	2,50	0	1	0	1	0	0	0	0
0,47	YJR048W	12345	70	Cytochrome c, isoform 1; also known as iso-1-cytochrome c; electron carrier of the mitochondrial intermembrane space that transfers electrons from ubiquinone-cytochrome c oxidoreductase to cytochrome c oxidase during cellular respiration; mutations	10,73	1	1	0	1	0	1	0	0
0,32	YBR262C	12380	11	Component of the MICOS complex; MICOS (formerly MINOS or MitOS) is a mitochondrial inner membrane complex that extends into the intermembrane space and has a role in the maintenance of crista junctions, inner membrane architecture, and formation of	1,58	0	1	0	1	0	1	0	1
0,39	YGR215W	12385	10	Mitochondrial ribosomal protein of the small subunit	0,76	1	1	0	0	0	0	0	0
0,182	YAL044W-A	12575	5	Putative protein of unknown function; similar to S. pombe uvi31 which is a putative DNA repair protein	0,57	0	1	0	0	0	1	0	0
0,12	YOL109W	12582	4	Peripheral membrane protein of the plasma membrane; interacts with Mid2p; regulates the cell integrity pathway mediated by Pkc1p and Slt2p; the authentic protein is detected in a phosphorylated state in highly purified mitochondria	0,55	1	1	0	0	0	0	0	0
0,22	YLR390W	12622	6	Putative protein of unknown function; the authentic, non-tagged protein is detected in highly purified mitochondria in high-throughput studies	2,24	0	0	0	1	1	1	0	0
0,17	YMR230W	12731	13	Protein component of the small (40S) ribosomal subunit; homologous to mammalian ribosomal protein S10, no bacterial homolog; RPS10B has a paralog, RPS10A, that arose from the whole genome duplication	1,00	1	1	0	0	0	1	0	0
0,25	YDR381C-A	12753	6	Protein of unknown function; localized to the mitochondrial outer membrane	0,97	0	1	0	1	0	0	0	0
0,24	YDL075W	12945	6	Ribosomal 60S subunit protein L31A; associates with karyopherin Sxm1p; loss of both Rpl31p and Rpl39p confers lethality; homologous to mammalian ribosomal protein L31, no bacterial homolog; RPL31A has a paralog, RPL31B, that arose from the whole gen	0,41	1	1	0	0	0	0	0	0
0,92	YPR020W	12970	164	Subunit g of the mitochondrial F1F0 ATP synthase; reversibly phosphorylated on two residues; unphosphorylated form is required for dimerization of the ATP synthase complex, which in turn determines oligomerization of the complex and the shape of inn	56,83	1	1	1	1	0	1	0	0
0,261	YOR327C	13006	5	Vesicle membrane receptor protein (v-SNARE); involved in the fusion between Golgi-derived secretory vesicles with the plasma membrane; Snc2p levels regulated by Vps45p; member of the synaptobrevin/VAMP family of R-type v-SNARE proteins; SNC2 has a p	0,55	1	0	0	1	0	1	0	1
0,202	YBL059C-A	13167	2	Protein involved in respiratory chain complex assembly or maintenance; protein of the mitochondrial intermembrane space; contains twin Cx9C motifs that can form coiled coil-helix-coiled-coil helix fold	0,87	0	0	0	1	0	0	0	0
0,18	YDR079W	13225	7	Chaperone that facilitates the assembly of cytochrome c oxidase; integral to the mitochondrial inner membrane; interacts with a subcomplex of subunits VII, VIIa, and VIII (Cox7p, Cox9p, and Cox8p) but not with the holoenzyme	1,01	0	1	0	1	0	1	0	0
0,35	YPL013C	13676	5	Mitochondrial ribosomal protein of the small subunit	1,15	0	1	0	0	0	0	0	0
0,24	YLR061W	13685	2	Ribosomal 60S subunit protein L22A; required for the oxidative stress response in yeast; homologous to mammalian ribosomal protein L22, no bacterial homolog; RPL22A has a paralog, RPL22B, that arose from the whole genome duplication	0,42	0	1	0	0	0	0	0	1

0,24	YFR049W	13772	7	Subunit of the mitochondrial alpha-ketoglutarate dehydrogenase; recruits E3 subunit (Lpd1p) to the E1-E2 (Kgd1p, Kgd2p) core; has similarity to human mitochondrial ribosomal protein MRP-S36	0,90	0	1	0	0	0	0	0	0
0,21	YBR255C-A	13824	2	Putative protein of unknown function; may interact with respiratory chain complexes III (ubiquinol-cytochrome c reductase) or IV (cytochrome c oxidase); identified by sequence comparison with hemiascomycetous yeast species	0,41	0	1	0	0	0	0	1	0
0,21	YIL052C	13861	6	Ribosomal 60S subunit protein L34B; homologous to mammalian ribosomal protein L34, no bacterial homolog; RPL34B has a paralog, RPL34A, that arose from the whole genome duplication	0,50	1	1	0	0	0	0	0	0
0,19	YDL136W	13947	4	Ribosomal 60S subunit protein L35B; homologous to mammalian ribosomal protein L35 and bacterial L29; RPL35B has a paralog, RPL35A, that arose from the whole genome duplication	0,34	0	1	0	0	0	0	0	0
0,12	YBL003C	13981	10	Histone H2A; core histone protein required for chromatin assembly and chromosome function; one of two nearly identical (see also HTA1) subtypes; DNA damage-dependent phosphorylation by Mec1p facilitates DNA repair; acetylated by Nat4p	0,56	1	1	0	1	0	1	0	0
0,295	YMR123W	14010	6	V-ATPase assembly factor; functions with other V-ATPase assembly factors in the ER to efficiently assemble the V-ATPase membrane sector (V0); protein abundance increases in response to DNA replication stress	1,43	0	0	0	1	0	0	0	0
0,19	YHL018W	14063	2	Putative 4a-hydroxytetrahydrobiopterin dehydratase; green fluorescent protein (GFP)-fusion protein localizes to mitochondria and is induced in response to the DNA-damaging agent MMS	0,40	0	1	0	0	0	0	1	0
0,4	YLR295C	14165	25	Subunit h of the F0 sector of mitochondrial F1F0 ATP synthase; F1F0 ATP synthase is a large, evolutionarily conserved enzyme complex required for ATP synthesis; protein abundance increases in response to DNA replication stress	1,67	0	1	0	1	0	0	0	0
0,28	YLR344W	14225	4	Ribosomal 60S subunit protein L26A; binds to 5.8S rRNA; non-essential even when paralog is also deleted; deletion has minimal affections on ribosome biosynthesis; homologous to mammalian ribosomal protein L26 and bacterial L24; RPL26A has a paralog,	1,10	0	1	0	0	0	0	1	0
0,17	YGR034W	14226	8	Ribosomal 60S subunit protein L26B; binds to 5.8S rRNA; non-essential even when paralog is also deleted; deletion has minimal affections on ribosome biosynthesis; homologous to mammalian ribosomal protein L26 and bacterial L24; RPL26B has a paralog,	0,50	1	1	0	0	0	0	0	0
0,2	YBL002W	14229	24	Histone H2B; core histone protein required for chromatin assembly and chromosome function; nearly identical to HTB1; Rad6p-Bre1p-Lge1p mediated ubiquitination regulates reassembly after DNA replication, transcriptional activation, meiotic DSB format	1,71	1	1	1	1	1	1	0	0
0,12	YOL012C	14274	4	Histone variant H2AZ; exchanged for histone H2A in nucleosomes by the SWR1 complex; involved in transcriptional regulation through prevention of the spread of silent heterochromatin; Htz1p-containing nucleosomes facilitate RNA Pol II passage by affe	0,48	0	0	1	0	1	1	0	0
0,171	YER072W	14419	3	Subunit of the vacuolar transporter chaperone (VTC) complex; VTC complex is involved in membrane trafficking, vacuolar polyphosphate accumulation, microautophagy and non-autophagic vacuolar fusion; also has mRNA binding activity; protein abundance i	1,37	0	0	0	1	0	0	0	0
0,31	YBL087C	14578	18	Ribosomal 60S subunit protein L23A; homologous to mammalian ribosomal protein L23 and bacterial L14; RPL23A has a paralog, RPL23B, that arose from the whole genome duplication	0,98	1	1	0	0	0	1	0	0
0,13	YCR031C	14585	10	Protein component of the small (40S) ribosomal subunit; required for ribosome assembly and 20S pre-rRNA processing; mutations confer cryptopleurine resistance; homologous to mammalian ribosomal protein S14 and bacterial S11; RPS14A has a paralog, RP	0,50	1	1	0	0	0	1	0	0
0,87	YDR529C	14613	145	Subunit 7 of ubiquinol cytochrome-c reductase (Complex III); Complex III is a component of the mitochondrial inner membrane electron transport chain; oriented facing the mitochondrial matrix; N-terminus appears to play a role in complex assembly	41,38	1	1	1	1	1	1	0	0
0,31	YCR083W	14651	18	Mitochondrial thioredoxin; highly conserved oxidoreductase required to maintain the redox homeostasis of the cell, forms the mitochondrial thioredoxin system with Trr2p, redox state is maintained by both Trr2p and Glr1p	2,78	1	1	1	0	0	0	0	0
0,23	YJL190C	14663	6	Protein component of the small (40S) ribosomal subunit; homologous to mammalian ribosomal protein S15A and bacterial S8; RPS22A has a paralog, RPS22B, that arose from the whole genome duplication	0,47	0	1	0	0	0	1	0	0
0,18	YBL092W	14762	6	Ribosomal 60S subunit protein L32; overexpression disrupts telomeric silencing; homologous to mammalian ribosomal protein L32, no bacterial homolog	1,03	0	1	0	0	0	0	0	0
0,16	YBR230C	14830	23	Mitochondrial outer membrane receptor for cytosolic ribosomes; integral protein of the outer membrane that interacts with the nascent chain-associated complex (NAC) bound to ribosomes, contributing to co-translational mitochondrial import; interacts	2,01	0	1	0	1	0	0	0	0
0,37	YKR094C	14830	19	Ubiquitin-ribosomal 60S subunit protein L40B fusion protein; cleaved to yield ubiquitin and ribosomal protein L40B; ubiquitin may facilitate assembly of the ribosomal protein into ribosomes; homologous to mammalian ribosomal protein L40, no bacteria	0,97	1	1	0	0	1	1	1	0
0,22	YOR103C	14918	2	Epsilon subunit of the oligosaccharyltransferase complex; located in the ER lumen; catalyzes asparagine-linked glycosylation of newly synthesized proteins	0,37	0	1	0	0	0	0	1	0
0,23	YKL003C	15011	18	Mitochondrial ribosomal protein of the small subunit; MRP17 exhibits genetic interactions with PET122, encoding a COX3-specific translational activator	0,74	0	1	0	0	0	0	0	0

0,186	YNL149C	15044	6	Essential protein required for maturation of Gas1p and Pho8p; involved in protein trafficking; GFP-fusion protein localizes to the ER and YFP-fusion protein to the nuclear envelope-ER network; null mutants have a cell separation defect	2,00	0	0	0	1	0	0	0	0
0,51	YGL191W	15069	100	Subunit VIa of cytochrome c oxidase; present in a subclass of cytochrome c oxidase complexes that may have a role in mimimizing generation of reactive oxygen species; not essential for cytochrome c oxidase activity but may modulate activity in respo	9,19	1	1	0	1	1	1	0	0
0,25	YHL001W	15201	15	Ribosomal 60S subunit protein L14B; homologous to mammalian ribosomal protein L14, no bacterial homolog; RPL14B has a paralog, RPL14A, that arose from the whole genome duplication; protein abundance increases in response to DNA replication stress	0,84	1	1	0	0	0	0	0	0
0,25	YKL170W	15217	7	Mitochondrial ribosomal protein of the large subunit; appears as two protein spots (YmL34 and YmL38) on two-dimensional SDS gels; protein abundance increases in response to DNA replication stress	0,99	0	1	0	0	0	0	0	0
0,38	YIL069C	15319	8	Protein component of the small (40S) ribosomal subunit; homologous to mammalian ribosomal protein S24, no bacterial homolog; RPS24B has a paralog, RPS24A, that arose from the whole genome duplication	0,98	1	1	0	0	0	0	0	0
0,103	YBR010W	15347	8	Histone H3; core histone protein required for chromatin assembly, part of heterochromatin-mediated telomeric and HM silencing; one of two identical histone H3 proteins (see HHT2); regulated by acetylation, methylation, and phosphorylation; H3K14 ace	0,75	0	1	1	0	1	1	0	0
0,35	YCR028C-A	15377	20	ssDNA-binding protein essential for mitochondrial genome maintenance; involved in mitochondrial DNA replication	2,02	0	1	0	0	0	0	0	0
0,439	YOR285W	15461	16	Thiosulfate sulfurtransferase; contains a rhodanese-like domain; localized to the mitochondrial outer membrane; protein abundance increases in response to DNA replication stress; similar to the human TSTD gene	3,86	0	0	0	1	1	1	0	0
0,35	YHR010W	15522	11	Ribosomal 60S subunit protein L27A; homologous to mammalian ribosomal protein L27, no bacterial homolog; RPL27A has a paralog, RPL27B, that arose from the whole genome duplication	1,56	0	1	0	0	0	0	0	0
0,16	YKL138C	15556	3	Mitochondrial ribosomal protein of the large subunit	0,50	0	1	0	0	0	0	0	0
0,26	YPR063C	15569	3	ER-localized protein of unknown function	0,50	0	1	0	0	0	0	0	0
0,25	YOL127W	15748	17	Ribosomal 60S subunit protein L25; primary rRNA-binding ribosomal protein component of large ribosomal subunit; binds to 25S rRNA via a conserved C-terminal motif; homologous to mammalian ribosomal protein L23A and bacterial L23	0,79	1	1	0	0	0	0	0	0
0,278	YDR511W	15773	5	Mitochondrial protein involved in assembly of succinate dehydrogenase; has a role in maturation of the Sdh2p subunit; localized to the mitochondrial intermembrane space; required for acetate utilization and gluconeogenesis; mutation in Drosophila or	1,87	0	0	0	1	0	0	0	0
0,55	YKR049C	15778	17	Putative redox protein containing a thioredoxin fold; the authentic, non-tagged protein is detected in highly purified mitochondria in high-throughput studies	4,51	0	1	0	0	0	0	0	0
0,23	YML024W	15836	9	Ribosomal protein 51 (rp51) of the small (40s) subunit; homologous to mammalian ribosomal protein S17, no bacterial homolog; RPS17A has a paralog, RPS17B, that arose from the whole genome duplication	0,54	1	1	0	0	0	0	0	0
0,29	YDL083C	15838	12	Protein component of the small (40S) ribosomal subunit; homologous to mammalian ribosomal protein S16 and bacterial S9; RPS16B has a paralog, RPS16A, that arose from the whole genome duplication	1,19	0	1	0	0	0	0	0	0
0,14	YER050C	15871	5	Mitochondrial ribosomal protein of the small subunit; has similarity to E. coli S18 ribosomal protein	0,49	0	1	0	0	0	0	0	0
0,26	YOL121C	15907	17	Protein component of the small (40S) ribosomal subunit; required for assembly and maturation of pre-40 S particles; homologous to mammalian ribosomal protein S19, no bacterial homolog; mutations in human RPS19 are associated with Diamond Blackfan an	0,69	1	1	0	0	0	1	0	0
0,34	YDR513W	15943	11	Cytoplasmic glutaredoxin; thioltransferase, glutathione-dependent disulfide oxidoreductase involved in maintaining redox state of target proteins, also exhibits glutathione peroxidase activity, expression induced in response to stress; GRX2 has two	0,98	0	1	0	1	0	0	0	1
0,18	YIL051C	15955	8	Mitochondrial protein required for transamination of isoleucine; but not of valine or leucine; may regulate specificity of branched-chain transaminases Bat1p and Bat2p; induction of expression in response to stress is mediated by a Hog1p-regulated a	0,64	1	1	0	0	0	0	0	0
0,3	YOL040C	15992	13	Protein component of the small (40S) ribosomal subunit; homologous to mammalian ribosomal protein S15 and bacterial S19	0,93	0	1	0	0	0	1	0	0
0,17	YPR100W	16251	5	Mitochondrial ribosomal protein of the large subunit	0,67	0	1	0	0	0	0	0	0
0,47	YJL104W	16252	26	Subunit of the import motor (PAM complex); the PAM complex is a component of the Translocase of the Inner Mitochondrial membrane (TIM23 complex); forms a 1:1 subcomplex with Pam18p and inhibits its cochaperone activity; contains a J-like domain	3,93	0	1	0	0	0	0	0	0
0,216	YNR022C	16389	5	Mitochondrial ribosomal protein of the large subunit; not essential for mitochondrial translation	1,42	0	0	0	0	0	1	0	0
0,17	YOR298C-A	16394	4	Transcriptional coactivator; bridges the DNA-binding region of Gcn4p and TATA-binding protein Spt15p; suppressor of frameshift mutations; protein abundance increases in response to DNA replication stress	0,77	0	1	1	0	0	0	0	0

0,14	YCR071C	16554	5	Mitochondrial ribosomal protein of the large subunit; conserved in metazoa, with similarity to human mitochondrial ribosomal protein MRPL49	0,65	0	1	0	0	0	0	0	0
0,54	YNL185C	16705	24	Mitochondrial ribosomal protein of the large subunit	2,96	0	1	0	0	0	0	0	0
0,28	YOR286W	16744	8	Protein with rhodanese activity; contains a rhodanese-like domain similar to Rdl1p, Uba4p, Tum1p, and Ych1p; overexpression causes a cell cycle delay; null mutant displays elevated frequency of mitochondrial genome loss	0,61	1	1	0	0	0	0	0	0
0,24	YNL131W	16780	8	Component of the TOM (Translocase of Outer Membrane) complex; responsible for initial import of mitochondrially directed proteins; mediates interaction between TOM and TIM complexes and acts as a receptor for precursor proteins	0,31	0	1	0	1	0	0	0	1
0,19	YIL087C	16913	4	Putative protein of unknown function; mitochondrial protein that physically interacts with Tim23p; null mutant displays reduced respiratory growth	0,63	0	1	0	0	0	0	0	0
0,2	YDR064W	17018	4	Protein component of the small (40S) ribosomal subunit; homologous to mammalian ribosomal protein S13 and bacterial S15	0,46	0	1	0	0	0	0	0	0
0,67	YDL004W	17067	69	Delta subunit of the central stalk of mitochondrial F1F0 ATP synthase; F1F0 ATP synthase is a large, evolutionarily conserved enzyme complex required for ATP synthesis; F1 translationally regulates ATP6 and ATP8 expression to achieve a balanced outp	3,66	1	1	1	1	0	0	0	0
0,36	YML026C	17084	20	Protein component of the small (40S) ribosomal subunit; homologous to mammalian ribosomal protein S18 and bacterial S13; RPS18B has a paralog, RPS18A, that arose from the whole genome duplication; protein abundance increases in response to DNA repli	1,24	1	1	0	0	0	1	0	0
0,37	YNL052W	17130	31	Subunit Va of cytochrome c oxidase; cytochrome c oxidase is the terminal member of the mitochondrial inner membrane electron transport chain; Cox5Ap is predominantly expressed during aerobic growth while its isoform Vb (Cox5Bp) is expressed during a	2,74	0	1	0	1	0	0	0	0
0,555	YGL187C	17316	26	Subunit IV of cytochrome c oxidase; the terminal member of the mitochondrial inner membrane electron transport chain; precursor N-terminal 25 residues are cleaved during mitochondrial import; phosphorylated; spermidine enhances translation	2,23	0	1	0	1	0	0	1	0
0,32	YFR033C	17339	10	Subunit 6 of the ubiquinol cytochrome-c reductase complex; the complex, also known as the cytochrome bc(1) complex or Complex III, is a component of the mitochondrial inner membrane electron transport chain; highly acidic protein; required for matur	0,44	0	1	0	0	0	0	0	0
0,15	YDR462W	17377	6	Mitochondrial ribosomal protein of the large subunit; protein abundance increases in response to DNA replication stress	0,61	0	1	0	0	0	0	0	0
0,53	YHR051W	17377	43	Subunit VI of cytochrome c oxidase (Complex IV); Complex IV is the terminal member of the mitochondrial inner membrane electron transport chain; expression is regulated by oxygen levels	3,93	0	1	1	1	0	1	0	0
0,28	YLR167W	17389	5	Fusion protein cleaved to yield ribosomal protein S31 and ubiquitin; ubiquitin may facilitate assembly of the ribosomal protein into ribosomes; interacts genetically with translation factor eIF2B; homologous to mammalian ribosomal protein S27A, no b	0,80	0	1	0	0	0	0	0	1
0,12	YGL031C	17603	6	Ribosomal 60S subunit protein L24A; not essential for translation but may be required for normal translation rate; homologous to mammalian ribosomal protein L24, no bacterial homolog; RPL24A has a paralog, RPL24B, that arose from the whole genome du	0,60	0	1	0	0	0	0	0	0
0,52	YMR158W	17689	13	Mitochondrial ribosomal protein of the small subunit	3,10	0	1	0	0	0	0	0	0
0,36	YIL065C	17814	9	Protein involved in mitochondrial fission and peroxisome abundance; may have a distinct role in tethering protein aggregates to mitochondria in order to retain them in the mother cell; required for localization of Dnm1p and Mdv1p during mitochondria	1,61	0	1	0	0	0	0	0	0
0,19	YDR025W	17830	12	Protein component of the small (40S) ribosomal subunit; homologous to mammalian ribosomal protein S11 and bacterial S17; N-terminally propionylated in vivo; RPS11A has a paralog, RPS11B, that arose from the whole genome duplication	1,01	0	1	0	0	0	0	0	0
0,291	YEL054C	17869	23	Ribosomal 60S subunit protein L12A; rpl12a rpl12b double mutant exhibits slow growth and slow translation; homologous to mammalian ribosomal protein L12 and bacterial L11; RPL12A has a paralog, RPL12B, that arose from the whole genome duplication	0,77	1	1	0	1	0	0	0	0
0,3	YLR008C	17899	18	Subunit of the import motor (PAM complex); the PAM complex is a component of the Translocase of the Inner Mitochondrial membrane (TIM23 complex); essential J-protein cochaperone that stimulates Ssc1p ATPase activity to drive import; inhibited by Pa	1,01	0	1	0	0	0	1	0	0
0,121	YPL135W	18021	4	Conserved protein of the mitochondrial matrix; performs a scaffolding function during assembly of iron-sulfur clusters, interacts physically and functionally with yeast frataxin (Yfh1p); isu1 isu2 double mutant is inviable; ISU1 has a paralog, ISU2,	0,37	0	1	0	1	0	0	0	0
0,195	YGL041W-A	18092	2	Putative protein of unknown function; conserved in fungi; identified by expression profiling and mass spectrometry	0,58	0	0	0	0	0	1	0	0
0,23	YBR191W	18277	9	Ribosomal 60S subunit protein L21A; homologous to mammalian ribosomal protein L21, no bacterial homolog; RPL21A has a paralog, RPL21B, that arose from the whole genome duplication	1,22	0	1	0	0	0	0	0	0
0,16	YIL016W	18341	4	Ribosome-associated protein; proposed to act in protein synthesis and nuclear pore complex biogenesis and maintenance as well as protein folding; has similarity to the mammalian BAG-1 protein	0,41	0	1	0	0	0	0	0	0
0,17	YJL096W	18376	9	Mitochondrial ribosomal protein of the large subunit	0,41	0	1	0	0	0	0	0	0

0,215	YOR150W	18544	9	Mitochondrial ribosomal protein of the large subunit; localizes to vacuole in response to H2O2	0,57	0	1	0	1	0	0	0	0
0,208	YML030W	18558	11	Cytochrome c oxidase subunit; required for assembly of the Complex III-Complex IV supercomplex, and for assembly of Cox13p and Rcf2p into cytochrome c oxidase; similar to Rcf2p, and either Rcf1p or Rcf2p is required for late-stage assembly of the Co	0,70	0	1	0	1	0	0	0	0
0,312	YGR076C	18575	6	Mitochondrial ribosomal protein of the large subunit; mutation confers increased replicative lifespan	1,21	0	0	0	1	0	1	0	0
0,52	YBR120C	18667	40	Mitochondrial protein required for translation of the COB mRNA; forms a complex with Cbp3p that binds to mt ribosomes near the polypeptide tunnel exit and promotes efficient translation of the COB mRNA; Cbp3p-Cbp6p complex also interacts with newly	8,26	0	1	0	0	0	0	0	0
0,18	YLR164W	18758	7	Putative alternate subunit of succinate dehydrogenase (SDH); mitochondrial inner membrane protein; genetic interaction with SDH4 suggests that Shh4p can function as a functional SDH subunit; a fraction copurifies with SDH subunit Sdh3p; expression i	0,56	0	1	0	0	0	0	0	0
0,27	YPR149W	19185	25	Protein of unknown function; contains transmembrane domains; involved in secretion of proteins that lack classical secretory signal sequences; component of the detergent-insoluble glycolipid-enriched complexes (DIGs); NCE102 has a paralog, FHN1, tha	1,58	0	1	0	1	0	0	0	0
0,26	YCR046C	19428	10	Mitochondrial ribosomal protein of the large subunit; required for respiration and for maintenance of the mitochondrial genome	1,13	0	1	0	0	0	0	0	0
0,2	YGR085C	19783	8	Ribosomal 60S subunit protein L11B; expressed at half the level of Rpl11Ap; involved in ribosomal assembly; depletion causes degradation of 60S proteins and RNA; homologous to mammalian ribosomal protein L11 and bacterial L5; RPL11B has a paralog, R	0,70	0	1	0	0	0	0	0	0
0,91	YKL016C	19797	486	Subunit d of the stator stalk of mitochondrial F1F0 ATP synthase; F1F0 ATP synthase is a large, evolutionarily conserved enzyme complex required for ATP synthesis	2281,85	1	1	0	1	0	0	0	0
0,22	YML073C	19949	7	Ribosomal 60S subunit protein L6A; N-terminally acetylated; binds 5.8S rRNA; homologous to mammalian ribosomal protein L6, no bacterial homolog; RPL6A has a paralog, RPL6B, that arose from the whole genome duplication	1,18	0	1	0	0	0	0	0	0
