# Supplementary material for: ATP synthase interactome analysis identifies a new subunit l as a modulator of permeability transition pore in yeast
Source: Sci Rep. 2023 Mar 7;13:3839. doi: 10.1038/s41598-023-30966-5 (PMC9992712; doi:10.1038/s41598-023-30966-5)
Supplement: Supplementary file 2 — Supplementary Information 2. [file 41598_2023_30966_MOESM2_ESM.pdf]

Protein complex and processes		
Ribosomal Proteins and Histones	YML009C	Mitochondrial ribosomal protein of the large subunit
	YMR286W	Mitochondrial ribosomal protein of the large subunit
	YOR150W	Mitochondrial ribosomal protein of the large subunit; localizes to vacuole in response to H2O2
	YGR076C	Mitochondrial ribosomal protein of the large subunit; mutation confers increased replicative lifespan
	YNR022C	Mitochondrial ribosomal protein of the large subunit; not essential for mitochondrial translation
	YMR225C	Mitochondrial ribosomal protein of the large subunit; protein abundance increases in response to DNA replication stress
	YGR215W	Mitochondrial ribosomal protein of the small subunit
	YNR037C	Mitochondrial ribosomal protein of the small subunit; has similarity to E. coli S19 ribosomal protein
	YOR167C	Protein component of the small (40S) ribosomal subunit; has an extraribosomal function in regulation of RPS28B, in which Rps28Ap binds to a decapping complex via Edc3p, which then binds to RPS28B mRNA leading to its decapping and degradation; homolo
	YMR230W	Protein component of the small (40S) ribosomal subunit; homologous to mammalian ribosomal protein S10, no bacterial homolog; RPS10B has a paralog, RPS10A, that arose from the whole genome duplication
	YJL190C	Protein component of the small (40S) ribosomal subunit; homologous to mammalian ribosomal protein S15A and bacterial S8; RPS22A has a paralog, RPS22B, that arose from the whole genome duplication
	YML026C	Protein component of the small (40S) ribosomal subunit; homologous to mammalian ribosomal protein S18 and bacterial S13; RPS18B has a paralog, RPS18A, that arose from the whole genome duplication; protein abundance increases in response to DNA repli
	YGR027C	Protein component of the small (40S) ribosomal subunit; homologous to mammalian ribosomal protein S25, no bacterial homolog; RPS25A has a paralog, RPS25B, that arose from the whole genome duplication
	YHR021C	Protein component of the small (40S) ribosomal subunit; homologous to mammalian ribosomal protein S27, no bacterial homolog; RPS27B has a paralog, RPS27A, that arose from the whole genome duplication
	YLR388W	Protein component of the small (40S) ribosomal subunit; homologous to mammalian ribosomal protein S29 and bacterial S14; RPS29A has a paralog, RPS29B, that arose from the whole genome duplication
	YOL121C	Protein component of the small (40S) ribosomal subunit; required for assembly and maturation of pre-40 S particles; homologous to mammalian ribosomal protein S19, no bacterial homolog; mutations in human RPS19 are associated with Diamond Blackfan an
	YCR031C	Protein component of the small (40S) ribosomal subunit; required for ribosome assembly and 20S pre-rRNA processing; mutations confer cryptopleurine resistance; homologous to mammalian ribosomal protein S14 and bacterial S11; RPS14A has a paralog, RP
	YEL054C	Ribosomal 60S subunit protein L12A; rpl12a rpl12b double mutant exhibits slow growth and slow translation; homologous to mammalian ribosomal protein L12 and bacterial L11; RPL12A has a paralog, RPL12B, that arose from the whole genome duplication
	YBL087C	Ribosomal 60S subunit protein L23A; homologous to mammalian ribosomal protein L23 and bacterial L14; RPL23A has a paralog, RPL23B, that arose from the whole genome duplication
	YOL127W	Ribosomal 60S subunit protein L25; primary rRNA-binding ribosomal protein component of large ribosomal subunit; binds to 25S rRNA via a conserved C-terminal motif; homologous to mammalian ribosomal protein L23A and bacterial L23
	YGL030W	Ribosomal 60S subunit protein L30; involved in pre-rRNA processing in the nucleolus; autoregulates splicing of its transcript; homologous to mammalian ribosomal protein L30, no bacterial homolog

	YDL075W	Ribosomal 60S subunit protein L31A; associates with karyopherin Sxm1p; loss of both Rpl31p and Rpl39p confers lethality; homologous to mammalian ribosomal protein L31, no bacterial homolog; RPL31A has a paralog, RPL31B, that arose from the whole gen	
	YMR194W	Ribosomal 60S subunit protein L36A; N-terminally acetylated; binds to 5.8 S rRNA; homologous to mammalian ribosomal protein L36, no bacterial homolog; RPL36A has a paralog, RPL36B, that arose from the whole genome duplication	
	YLR325C	Ribosomal 60S subunit protein L38; homologous to mammalian ribosomal protein L38, no bacterial homolog	
	YBL003C	Histone H2A; core histone protein required for chromatin assembly and chromosome function; one of two nearly identical (see also HTA1) subtypes; DNA damage-dependent phosphorylation by Mec1p facilitates DNA repair; acetylated by Nat4p	
	YBL002W	Histone H2B; core histone protein required for chromatin assembly and chromosome function; nearly identical to HTB1; Rad6p-Bre1p-Lge1p mediated ubiquitination regulates reassembly after DNA replication, transcriptional activation, meiotic DSB format	
	YBR010W	Histone H3; core histone protein required for chromatin assembly, part of heterochromatin-mediated telomeric and HM silencing; one of two identical histone H3 proteins (see HHT2); regulated by acetylation, methylation, and phosphorylation; H3K14 ace	
	YBR009C	Histone H4; core histone protein required for chromatin assembly and chromosome function; one of two identical histone proteins (see also HHF2); contributes to telomeric silencing; N-terminal domain involved in maintaining genomic integrity	
	YOL012C	Histone variant H2AZ; exchanged for histone H2A in nucleosomes by the SWR1 complex; involved in transcriptional regulation through prevention of the spread of silent heterochromatin; Htz1p-containing nucleosomes facilitate RNA Pol II passage by affe	
**ATP Synthase Complex**	Q0080	Subunit 8 of the F0 sector of mitochondrial F1F0 ATP synthase; encoded on the mitochondrial genome; ATP8 and ATP6 mRNAs are not translated in the absence of the F1 sector of ATPase	
	YML081C-A	Subunit i/j of the mitochondrial F1F0 ATP synthase; F1F0 ATP synthase is a large, evolutionarily conserved enzyme complex required for ATP synthesis; termed subunit I or subunit j; does not correspond to known ATP synthase subunits in other organisms	
	YPL271W	Epsilon subunit of the F1 sector of mitochondrial F1F0 ATP synthase; which is a large, evolutionarily conserved enzyme complex required for ATP synthesis; F1 translationally regulates ATP6 and ATP8 expression to achieve a balanced output of ATP synt	
	YOL077W-A	Subunit k of the mitochondrial F1F0 ATP synthase; F1F0 ATP synthase is a large, evolutionarily conserved enzyme complex required for ATP synthesis; associated only with the dimeric form of ATP synthase	
	Q0130	F0-ATP synthase subunit c (ATPase-associated proteolipid); encoded on the mitochondrial genome; mutation confers oligomycin resistance; expression is specifically dependent on the nuclear genes AEP1 and AEP2	
	YDR322C-A	Subunit e of mitochondrial F1F0-ATPase; ATPase is a large, evolutionarily conserved enzyme complex required for ATP synthesis; essential for the dimeric and oligomeric state of ATP synthase, which in turn determines the shape of inner membrane crist	
	YDR377W	Subunit f of the F0 sector of mitochondrial F1F0 ATP synthase; F1F0 ATP synthase is a large, evolutionarily conserved enzyme complex required for ATP synthesis	
	YPR020W	Subunit g of the mitochondrial F1F0 ATP synthase; reversibly phosphorylated on two residues; unphosphorylated form is required for dimerization of the ATP synthase complex, which in turn determines oligomerization of the complex and the shape of inn	
	YLR295C	Subunit h of the F0 sector of mitochondrial F1F0 ATP synthase; F1F0 ATP synthase is a large, evolutionarily conserved enzyme complex required for ATP synthesis; protein abundance increases in response to DNA replication stress	
	YDL004W	Delta subunit of the central stalk of mitochondrial F1F0 ATP synthase; F1F0 ATP synthase is a large, evolutionarily conserved enzyme complex required for ATP synthesis; F1 translationally regulates ATP6 and ATP8 expression to achieve a balanced outp	

	YKL016C	Subunit d of the stator stalk of mitochondrial F1F0 ATP synthase; F1F0 ATP synthase is a large, evolutionarily conserved enzyme complex required for ATP synthesis
**Complex IV and Cyt C**	YDR119W-A	Putative protein of unknown function; copurifies with respiratory chain supercomplexes composed of Complex III (ubiquinol-cytochrome c reductase) and Complex IV (cytochrome c oxidase), Cox26
	YNL052W	Subunit Va of cytochrome c oxidase; cytochrome c oxidase is the terminal member of the mitochondrial inner membrane electron transport chain; Cox5Ap is predominantly expressed during aerobic growth while its isoform Vb (Cox5Bp) is expressed during a
	YHR051W	Subunit VI of cytochrome c oxidase (Complex IV); Complex IV is the terminal member of the mitochondrial inner membrane electron transport chain; expression is regulated by oxygen levels
	YGL191W	Subunit VIa of cytochrome c oxidase; present in a subclass of cytochrome c oxidase complexes that may have a role in mimimizing generation of reactive oxygen species; not essential for cytochrome c oxidase activity but may modulate activity in respo
	YLR038C	Subunit VIb of cytochrome c oxidase; cytochrome c oxidase is also known as respiratory Complex IV and is the terminal member of the mitochondrial inner membrane electron transport chain; required for assembly of cytochrome c oxidase but not required
	YMR256C	Subunit VII of cytochrome c oxidase (Complex IV); Complex IV is the terminal member of the mitochondrial inner membrane electron transport chain
	YDL067C	Subunit VIIa of cytochrome c oxidase (Complex IV); Complex IV is the terminal member of the mitochondrial inner membrane electron transport chain
	YLR395C	Subunit VIII of cytochrome c oxidase (Complex IV); Complex IV is the terminal member of the mitochondrial inner membrane electron transport chain
	YGL187C	Subunit IV of cytochrome c oxidase; the terminal member of the mitochondrial inner membrane electron transport chain; precursor N-terminal 25 residues are cleaved during mitochondrial import; phosphorylated; spermidine enhances translation
	YML030W	Cytochrome c oxidase subunit; required for assembly of the Complex III-Complex IV supercomplex, and for assembly of Cox13p and Rcf2p into cytochrome c oxidase; similar to Rcf2p, and either Rcf1p or Rcf2p is required for late-stage assembly of the Co
	YJR048W	Cytochrome c, isoform 1; also known as iso-1-cytochrome c; electron carrier of the mitochondrial intermembrane space that transfers electrons from ubiquinone-cytochrome c oxidoreductase to cytochrome c oxidase during cellular respiration; mutations
	YJL062W-A	Mitochondrial protein required for cytochrome c oxidase assembly; also involved in translational regulation of Cox1p and prevention of Cox1p aggregation before assembly; located in the mitochondrial inner membrane
**Complex III**	YDR529C	Subunit 7 of ubiquinol cytochrome-c reductase (Complex III); Complex III is a component of the mitochondrial inner membrane electron transport chain; oriented facing the mitochondrial matrix; N-terminus appears to play a role in complex assembly
	YJL166W	Subunit 8 of ubiquinol cytochrome-c reductase (Complex III); Complex III is a component of the mitochondrial inner membrane electron transport chain; oriented facing the intermembrane space; expression is regulated by Abf1p and Cpf1p
	YGR183C	Subunit 9 of ubiquinol cytochrome-c reductase (Complex III); Complex III is a component of the mitochondrial inner membrane electron transport chain; required for electron transfer at the ubiquinol oxidase site of the complex
	YHR001W-A	Subunit of the ubiqunol-cytochrome c oxidoreductase complex; this complex comprises part of the mitochondrial respiratory chain; members include Cobp, Rip1p, Cyt1p, Cor1p, Qcr2p, Qcr6p, Qcr7p, Qcr8p, Qcr9p, and Qcr10p and comprises part of the mitoc
**MICOS, TIM/TOM Complex**	YBR262C	Component of the MICOS complex; MICOS (formerly MINOS or MitOS) is a mitochondrial inner membrane complex that extends into the intermembrane space and has a role in the maintenance of crista junctions, inner membrane architecture, and formation of
	YNL131W	Component of the TOM (Translocase of Outer Membrane) complex; responsible for initial import of mitochondrially directed proteins; mediates interaction between TOM and TIM complexes and acts as a receptor for precursor proteins
	YCL057C-A	Conserved component of the MICOS complex; MICOS (formerly MINOS or MitOS) is a mitochondrial inner membrane complex that extends into the intermembrane space and has a role in the maintenance of crista junctions, inner membrane architecture, and for
	YNL070W	Component of the TOM (translocase of outer membrane) complex; responsible for recognition and initial import steps for all mitochondrially directed proteins; promotes assembly and stability of the TOM complex
	YHR005C-A	Essential protein of the mitochondrial intermembrane space; forms a complex with Tim9p (TIM10 complex) that delivers hydrophobic proteins to the TIM22 complex for insertion into the inner membrane
	YIL087C	Putative protein of unknown function; mitochondrial protein that physically interacts with Tim23p; null mutant displays reduced respiratory growth
	YEL020W-A	Essential protein of the mitochondrial intermembrane space; forms a complex with Tim10p (TIM10 complex) that delivers hydrophobic proteins to the TIM22 complex for insertion into the inner membrane

**Redox and Fe-S cluster**	YOR285W	Thiosulfate sulfurtransferase; contains a rhodanese-like domain; localized to the mitochondrial outer membrane; protein abundance increases in response to DNA replication stress; similar to the human TSTD gene
	YPL135W	Conserved protein of the mitochondrial matrix; performs a scaffolding function during assembly of iron-sulfur clusters, interacts physically and functionally with yeast frataxin (Yfh1p); isu1 isu2 double mutant is inviable; ISU1 has a paralog, ISU2,
	YLR043C	Cytoplasmic thioredoxin isoenzyme; part of thioredoxin system which protects cells against oxidative and reductive stress; forms LMA1 complex with Pbi2p; acts as a cofactor for Tsa1p; required for ER-Golgi transport and vacuole inheritance; with Trx
	YCR083W	Mitochondrial thioredoxin; highly conserved oxidoreductase required to maintain the redox homeostasis of the cell, forms the mitochondrial thioredoxin system with Trr2p, redox state is maintained by both Trr2p and Glr1p
**Complex III LYR Proteins**	YDR511W	Mitochondrial protein involved in assembly of succinate dehydrogenase; has a role in maturation of the Sdh2p subunit; localized to the mitochondrial intermembrane space; required for acetate utilization and gluconeogenesis; mutation in Drosophila or
	YDR379C-A	Mitochondrial protein involved in assembly of succinate dehydrogenase; has a role in maturation of the Sdh2p subunit; member of the LYR protein family; mutations in human ortholog SDHAF1 are associated with infantile leukoencephalopathy
**Sec61 Translocation complex**	YER087C-B	Beta subunit of Sec61p ER translocation complex (Sec61p-Sss1p-Sbh1p); involved in protein translocation into the endoplasmic reticulum; interacts with the exocyst complex and also with Rtn1p; cotranslationally N-acetylated by NatA; SBH1 has a paralo
	YDR086C	Subunit of the Sec61p translocation complex (Sec61p-Sss1p-Sbh1p); this complex forms a channel for passage of secretory proteins through the endoplasmic reticulum membrane, and of the Ssh1p complex (Ssh1p-Sbh2p-Sss1p); interacts with Ost4p and Wbp1p
	YER019C-A	Ssh1p-Sss1p-Sbh2p complex component; involved in protein translocation into the endoplasmic reticulum; SBH2 has a paralog, SBH1, that arose from the whole genome duplication
**Uncharacterized**	YPR149W	Protein of unknown function; contains transmembrane domains; involved in secretion of proteins that lack classical secretory signal sequences; component of the detergent-insoluble glycolipid-enriched complexes (DIGs); NCE102 has a paralog, FHN1, tha
	YBR162W-A	Protein of unknown function; expression suppresses a secretory pathway mutation in E. coli; has similarity to the mammalian RAMP4 protein involved in secretion
	YLR361C-A	Putative protein of unknown function
	YPR010C-A	Putative protein of unknown function; conserved among Saccharomyces sensu stricto species
	YOR020W-A	Putative protein of unknown function; conserved in A. gossypii; the authentic, non-tagged protein is detected in highly purified mitochondria in high-throughput studies
	YGL041W-A	Putative protein of unknown function; conserved in fungi; identified by expression profiling and mass spectrometry
	YDR119W-A	Putative protein of unknown function; copurifies with respiratory chain supercomplexes composed of Complex III (ubiquinol-cytochrome c reductase) and Complex IV (cytochrome c oxidase)
	YIR021W-A	Putative protein of unknown function; identified by expression profiling and mass spectrometry
	YBR298C-A	Putative protein of unknown function; identified by gene-trapping, microarray-based expression analysis, and genome-wide homology searching
	YIL156W-B	Putative protein of unknown function; originally identified based on homology to <i>Ashbya gossypii</i> and other related yeasts
	YAL044W-A	Putative protein of unknown function; similar to S. pombe uvi31 which is a putative DNA repair protein
	YLR390W	Putative protein of unknown function; the authentic, non-tagged protein is detected in highly purified mitochondria in high-throughput studies
