# Supplementary material for: ATP synthase interactome analysis identifies a new subunit l as a modulator of permeability transition pore in yeast
Source: Sci Rep. 2023 Mar 7;13:3839. doi: 10.1038/s41598-023-30966-5 (PMC9992712; doi:10.1038/s41598-023-30966-5)
Supplement: Supplementary file 3 — Supplementary Information 3. [file 41598_2023_30966_MOESM3_ESM.pdf]

Uniprot_Acc	Protein description	Monomer	Dimer	Mol.wt	Prot.coverage	Peptides	emPAI_Monomer	emPAI_Dimer
P56381	ATP synthase subunit epsilon, mitochondrial OS=Homo sapiens OX=9606 GN=ATP5F1E PE=1 SV=1	1	1	5860	23,55	8	0,97	2,87
Q8N4H5	Mitochondrial import receptor subunit TOM5 homolog OS=Homo sapiens OX=9606 GN=TOMM5 PE=1 SV=1	1	1	6031	47,05	40	52,89	6,34
Q9P0U1	Mitochondrial import receptor subunit TOM7 homolog OS=Homo sapiens OX=9606 GN=TOMM7 PE=1 SV=1	1	0	6244	19,1	6	2,61	
Q96IX5	ATP synthase membrane subunit K, mitochondrial OS=Homo sapiens OX=9606 GN=ATP5MK PE=1 SV=1	1	1	6510	36,25	19	0,84	71,24
P56378	ATP synthase subunit ATP5MJ, mitochondrial OS=Homo sapiens OX=9606 GN=ATP5MJ PE=1 SV=1	0	1	6658	48,3	18		10,1
O75438	NADH dehydrogenase [ubiquinone] 1 beta subcomplex subunit 1 OS=Homo sapiens OX=9606 GN=NDUFB1 PE=1 SV=1	1	1	7014	15,5	10	2,16	4,61
Q14061	Cytochrome c oxidase copper chaperone OS=Homo sapiens OX=9606 GN=COX17 PE=1 SV=2	1	0	7253	25,4	1	0,75	
P15954	Cytochrome c oxidase subunit 7C, mitochondrial OS=Homo sapiens OX=9606 GN=COX7C PE=1 SV=1	1	1	7298	14,3	3	0,73	0,73
Q9UDW1	Cytochrome b-c1 complex subunit 9 OS=Homo sapiens OX=9606 GN=UQCR10 PE=1 SV=3	1	1	7304	24,6	5	2,01	0,73
P10176	Cytochrome c oxidase subunit 8A, mitochondrial OS=Homo sapiens OX=9606 GN=COX8A PE=1 SV=2	1	0	7631	13	1	0,69	
P60059	Protein transport protein Sec61 subunit gamma OS=Homo sapiens OX=9606 GN=SEC61G PE=1 SV=1	1	1	7793	19,1	4	0,68	0,68
P62857	40S ribosomal protein S28 OS=Homo sapiens OX=9606 GN=RPS28 PE=1 SV=1	1	1	7893	30,4	8	3,67	1,79
P56385	ATP synthase subunit e, mitochondrial OS=Homo sapiens OX=9606 GN=ATP5ME PE=1 SV=2	1	1	7928	48,55	68	1,79	59,73
Q96B49	Mitochondrial import receptor subunit TOM6 homolog OS=Homo sapiens OX=9606 GN=TOMM6 PE=1 SV=1	1	1	7997	31,75	16	3,57	1,75
P03928	ATP synthase protein 8 OS=Homo sapiens OX=9606 GN=MT-ATP8 PE=1 SV=1	1	1	8043	34,55	40	0,66	33,67
Q9UBI6	Guanine nucleotide-binding protein G(I)/G(S)/G(O) subunit gamma-12 OS=Homo sapiens OX=9606 GN=GNG12 PE=1 SV=3	1	1	8115	34,7	5	1,72	1,72
P63173	60S ribosomal protein L38 OS=Homo sapiens OX=9606 GN=RPL38 PE=1 SV=2	1	0	8270	34,3	4	3,32	
O43677	NADH dehydrogenase [ubiquinone] 1 subunit C1, mitochondrial OS=Homo sapiens OX=9606 GN=NDUFC1 PE=1 SV=1	1	0	8729	13,2	7	0,59	
P09669	Cytochrome c oxidase subunit 6C OS=Homo sapiens OX=9606 GN=COX6C PE=1 SV=2	1	1	8776	37,35	26	163,48	3,02
Q15843	NEDD8 OS=Homo sapiens OX=9606 GN=NEDD8 PE=1 SV=1	1	1	9066	15,45	2	0,56	0,56
P61960	Ubiquitin-fold modifier 1 OS=Homo sapiens OX=9606 GN=UFM1 PE=1 SV=1	0	1	9169	17,6	2		0,56
P24311	Cytochrome c oxidase subunit 7B, mitochondrial OS=Homo sapiens OX=9606 GN=COX7B PE=1 SV=2	1	1	9212	10	6	0,56	0,65
P63220	40S ribosomal protein S21 OS=Homo sapiens OX=9606 GN=RPS21 PE=1 SV=1	1	1	9220	14,45	2	0,56	0,56
O95167	NADH dehydrogenase [ubiquinone] 1 alpha subcomplex subunit 3 OS=Homo sapiens OX=9606 GN=NDUFA3 PE=1 SV=1	1	1	9273	21,4	5	1,4	0,55
P14406	Cytochrome c oxidase subunit 7A2, mitochondrial OS=Homo sapiens OX=9606 GN=COX7A2 PE=1 SV=1	1	1	9390	34,95	13	1,38	2,66
O00483	Cytochrome c oxidase subunit NDUFA4 OS=Homo sapiens OX=9606 GN=NDUFA4 PE=1 SV=1	1	1	9421	22,2	4	1,38	1,37
Q9NRP2	COX assembly mitochondrial protein 2 homolog OS=Homo sapiens OX=9606 GN=CMC2 PE=1 SV=1	1	1	9682	17,1	4	0,53	
Q71UM5	40S ribosomal protein S27-like OS=Homo sapiens OX=9606 GN=RPS27L PE=1 SV=3	0	1	9813	15,5	1		0,52
O14949	Cytochrome b-c1 complex subunit 8 OS=Homo sapiens OX=9606 GN=UQCRQ PE=1 SV=4	0	1	9900	37,8	10		4,23
P60468	Protein transport protein Sec61 subunit beta OS=Homo sapiens OX=9606 GN=SEC61B PE=1 SV=2	1	1	10025	15,6	2	0,51	0,51
P07108	Acyl-CoA-binding protein OS=Homo sapiens OX=9606 GN=DBI PE=1 SV=2	1	0	10038	23	8	0,51	
Q9BQ48	39S ribosomal protein L34, mitochondrial OS=Homo sapiens OX=9606 GN=MRPL34 PE=1 SV=1	1	1	10159	13	4	0,49	0,49
P14854	Cytochrome c oxidase subunit 6B1 OS=Homo sapiens OX=9606 GN=COX6B1 PE=1 SV=2	1	0	10414	26,7	7	2,25	
Q9NU23	LYR motif-containing protein 2 OS=Homo sapiens OX=9606 GN=LYRM2 PE=1 SV=1	1	1	10443	15,35	3	0,48	0,48
Q96FJ2	Dynein light chain 2, cytoplasmic OS=Homo sapiens OX=9606 GN=DYNLL2 PE=1 SV=1	1	1	10457	16,3	3	1,19	0,48

P63167	Dynein light chain 1, cytoplasmic OS=Homo sapiens OX=9606 GN=DYNLL1 PE=1 SV=1	1	0	10530	20,2	2	1,18	
P62072	Mitochondrial import inner membrane translocase subunit Tim10 OS=Homo sapiens OX=9606 GN=TIMM10 PE=1 SV=1	1	0	10554	22,2	5	2,21	
Q9Y5J7	Mitochondrial import inner membrane translocase subunit Tim9 OS=Homo sapiens OX=9606 GN=TIMM9 PE=1 SV=1	1	1	10599	21,9	5	2,21	0,47
Q49B96	Cytochrome c oxidase assembly protein COX19 OS=Homo sapiens OX=9606 GN=COX19 PE=1 SV=1	0	1	10615	11,1	1		0,47
Q9Y5L4	Mitochondrial import inner membrane translocase subunit Tim13 OS=Homo sapiens OX=9606 GN=TIMM13 PE=1 SV=1	1	1	10721	31,55	9	0,46	5,72
Q9HD34	LYR motif-containing protein 4 OS=Homo sapiens OX=9606 GN=LYRM4 PE=1 SV=1	1	1	10752	22	4	1,14	1,14
P62304	Small nuclear ribonucleoprotein E OS=Homo sapiens OX=9606 GN=SNRPE PE=1 SV=1	1	0	10854	12	2	1,13	
Q6IPR1	Electron transfer flavoprotein regulatory factor 1 OS=Homo sapiens OX=9606 GN=ETFRF1 PE=1 SV=2	1	1	10857	11,1	3	0,46	0,46
P05109	Protein S100-A8 OS=Homo sapiens OX=9606 GN=S100A8 PE=1 SV=1	1	1	10885	19,9	6	0,46	2,1
Q8TF09	Dynein light chain roadblock-type 2 OS=Homo sapiens OX=9606 GN=DYNLRB2 PE=1 SV=1	1	1	10905	23,45	7	5,6	0,46
P82921	28S ribosomal protein S21, mitochondrial OS=Homo sapiens OX=9606 GN=MRPS21 PE=1 SV=3	1	1	10909	23	5	0,46	2,1
P61604	10 kDa heat shock protein, mitochondrial OS=Homo sapiens OX=9606 GN=HSPE1 PE=1 SV=2	1	1	10925	78,45	491	2760,96	62,35
A0A096LP55	Cytochrome b-c1 complex subunit 6-like, mitochondrial OS=Homo sapiens OX=9606 GN=UQCRHL PE=3 SV=1	1	0	10973	23,1	1	0,45	
P01040	Cystatin-A OS=Homo sapiens OX=9606 GN=CSTA PE=1 SV=1	1	1	11000	65,8	30	12,67	18,84
P56134	ATP synthase subunit f, mitochondrial OS=Homo sapiens OX=9606 GN=ATP5MF PE=1 SV=3	1	1	11025	19,65	22	0,45	8,4
O43678	NADH dehydrogenase [ubiquinone] 1 alpha subcomplex subunit 2 OS=Homo sapiens OX=9606 GN=NDUFA2 PE=1 SV=3	1	1	11029	29,8	9	1,11	2,07
L0R6Q1	SLC35A4 upstream open reading frame protein OS=Homo sapiens OX=9606 GN=SLC35A4 PE=3 SV=1	1	1	11183	25,75	7	2	0,44
P04080	Cystatin-B OS=Homo sapiens OX=9606 GN=CSTB PE=1 SV=2	1	1	11190	18,35	4	0,44	1,08
O60220	Mitochondrial import inner membrane translocase subunit Tim8 A OS=Homo sapiens OX=9606 GN=TIMM8A PE=1 SV=1	1	0	11219	20,6	3	0,44	
Q5QNW6	Histone H2B type 2-F OS=Homo sapiens OX=9606 GN=H2BC18 PE=1 SV=3	0	1	11360	11,9	2	0,35	
P81605	Dermcidin OS=Homo sapiens OX=9606 GN=DCD PE=1 SV=2	1	1	11391	10	3	0,43	0,43
O43676	NADH dehydrogenase [ubiquinone] 1 beta subcomplex subunit 3 OS=Homo sapiens OX=9606 GN=NDUFB3 PE=1 SV=3	0	1	11395	10,2	1		0,43
Q86SG5	Protein S100-A7A OS=Homo sapiens OX=9606 GN=S100A7A PE=1 SV=3	0	1	11412	21,8	26		7,64
O75964	ATP synthase subunit g, mitochondrial OS=Homo sapiens OX=9606 GN=ATP5MG PE=1 SV=3	1	1	11421	45,8	74	0,45	73,73
P82909	Alpha-ketoglutarate dehydrogenase component 4 OS=Homo sapiens OX=9606 GN=MRPS36 PE=1 SV=2	1	1	11459	63,1	13	5,04	3,21
P31151	Protein S100-A7 OS=Homo sapiens OX=9606 GN=S100A7 PE=1 SV=4	1	1	11578	32,7	53	7,48	16,27
P05387	60S acidic ribosomal protein P2 OS=Homo sapiens OX=9606 GN=RPLP2 PE=1 SV=1	1	1	11658	70	20	4,84	4,84
Q9Y2R0	Cytochrome c oxidase assembly factor 3 homolog, mitochondrial OS=Homo sapiens OX=9606 GN=COA3 PE=1 SV=1	1	1	11724	18,4	4	1,01	0,42
Q9Y5J6	Mitochondrial import inner membrane translocase subunit Tim10 B OS=Homo sapiens OX=9606 GN=TIMM10B PE=1 SV=1	1	0	11807	46,6	6	4,75	
P99999	Cytochrome c OS=Homo sapiens OX=9606 GN=CYCS PE=1 SV=2	1	1	11855	58,1	57	63,08	14,99
P56181	NADH dehydrogenase [ubiquinone] flavoprotein 3, mitochondrial OS=Homo sapiens OX=9606 GN=NDUFV3 PE=1 SV=2	0	1	11990	14,8	1		0,41
P62942	Peptidyl-prolyl cis-trans isomerase FKBP1A OS=Homo sapiens OX=9606 GN=FKBP1A PE=1 SV=2	1	1	12000	12	2	0,41	0,41

Q5U5X0	Complex III assembly factor LYRM7 OS=Homo sapiens OX=9606 GN=LYRM7 PE=1 SV=1	1	1	12004	15,4	4	1,8	0,41
P10599	Thioredoxin OS=Homo sapiens OX=9606 GN=TXN PE=1 SV=3	1	1	12015	24,8	8	0,99	1,01
P35754	Glutaredoxin-1 OS=Homo sapiens OX=9606 GN=GLRX PE=1 SV=2	1	1	12053	10,85	4	0,98	0,41
P12074	Cytochrome c oxidase subunit 6A1, mitochondrial OS=Homo sapiens OX=9606 GN=COX6A1 PE=1 SV=4	1	0	12147	11,9	2	0,98	
Q53S33	BolA-like protein 3 OS=Homo sapiens OX=9606 GN=BOLA3 PE=1 SV=1	1	1	12163	24,75	6	1,75	0,96
Q9UII2	ATPase inhibitor, mitochondrial OS=Homo sapiens OX=9606 GN=ATP5IF1 PE=1 SV=1	1	1	12241	22,65	84	1,75	9,61
Q9BQC6	Ribosomal protein 63, mitochondrial OS=Homo sapiens OX=9606 GN=MRPL57 PE=1 SV=1	1	1	12259	16,15	5	0,4	0,96
Q96EL3	39S ribosomal protein L53, mitochondrial OS=Homo sapiens OX=9606 GN=MRPL53 PE=1 SV=1	1	1	12270	25,45	10	2,81	1,73
Q9GZT3	SRA stem-loop-interacting RNA-binding protein, mitochondrial OS=Homo sapiens OX=9606 GN=SLIRP PE=1 SV=1	1	1	12398	52,75	35	18,79	9,18
Q96DA6	Mitochondrial import inner membrane translocase subunit TIM14 OS=Homo sapiens OX=9606 GN=DNAJC19 PE=1 SV=3	1	1	12491	19,4	5	0,39	0,93
P18859	ATP synthase-coupling factor 6, mitochondrial OS=Homo sapiens OX=9606 GN=ATP5PF PE=1 SV=1	0	1	12580	62	25		36,18
O95182	NADH dehydrogenase [ubiquinone] 1 alpha subcomplex subunit 7 OS=Homo sapiens OX=9606 GN=NDUFA7 PE=1 SV=3	1	1	12601	20,35	6	0,92	1,66
Q9Y291	28S ribosomal protein S33, mitochondrial OS=Homo sapiens OX=9606 GN=MRPS33 PE=1 SV=1	0	1	12621	31,1	4		1,66
P14174	Macrophage migration inhibitory factor OS=Homo sapiens OX=9606 GN=MIF PE=1 SV=4	1	1	12639	10	5	1,66	0,39
P61803	Dolichyl-diphosphooligosaccharide--protein glycosyltransferase subunit DAD1 OS=Homo sapiens OX=9606 GN=DAD1 PE=1 SV=3	1	1	12660	15,05	4	0,39	0,92
O14548	Cytochrome c oxidase subunit 7A-related protein, mitochondrial OS=Homo sapiens OX=9606 GN=COX7A2L PE=1 SV=2	1	1	12664	10	2	0,39	0,39
O43920	NADH dehydrogenase [ubiquinone] iron-sulfur protein 5 OS=Homo sapiens OX=9606 GN=NDUFS5 PE=1 SV=3	1	1	12737	16,95	6	0,34	1,64
O75368	SH3 domain-binding glutamic acid-rich-like protein OS=Homo sapiens OX=9606 GN=SH3BGRL PE=1 SV=1	1	1	12766	29,85	12	1,64	0,91
P30046	D-dopachrome decarboxylase OS=Homo sapiens OX=9606 GN=DDT PE=1 SV=3	1	1	12818	29,65	14	2,64	0,91
P41567	Eukaryotic translation initiation factor 1 OS=Homo sapiens OX=9606 GN=EIF1 PE=1 SV=1	1	1	12839	19,45	3	0,9	0,38
Q96HJ9	Protein FMC1 homolog OS=Homo sapiens OX=9606 GN=FMC1 PE=1 SV=2	1	1	12855	19,05	5	0,9	0,9
P06702	Protein S100-A9 OS=Homo sapiens OX=9606 GN=S100A9 PE=1 SV=1	1	1	13291	43	16	3,72	2,46
Q16864	V-type proton ATPase subunit F OS=Homo sapiens OX=9606 GN=ATP6V1F PE=1 SV=2	0	1	13362	33,6	4		1,54
P60866	40S ribosomal protein S20 OS=Homo sapiens OX=9606 GN=RPS20 PE=1 SV=1	1	1	13478	10,1	5	0,36	0,36
Q16718	NADH dehydrogenase [ubiquinone] 1 alpha subcomplex subunit 5 OS=Homo sapiens OX=9606 GN=NDUFA5 PE=1 SV=3	1	1	13507	17,2	10	0,84	1,5
P14927	Cytochrome b-c1 complex subunit 7 OS=Homo sapiens OX=9606 GN=UQCRB PE=1 SV=2	1	1	13522	28,35	8	0,84	2,39
P0C0S5	Histone H2A.Z OS=Homo sapiens OX=9606 GN=H2AZ1 PE=1 SV=2	1	1	13545	9,75	4	0,36	0,36
Q96BP2	Coiled-coil-helix-coiled-coil-helix domain-containing protein 1 OS=Homo sapiens OX=9606 GN=CHCHD1 PE=1 SV=1	1	1	13694	17,8	6	0,83	0,35
P62851	40S ribosomal protein S25 OS=Homo sapiens OX=9606 GN=RPS25 PE=1 SV=1	1	1	13791	11,2	3	0,35	0,35
Q9Y3D7	Mitochondrial import inner membrane translocase subunit TIM16 OS=Homo sapiens OX=9606 GN=PAM16 PE=1 SV=2	1	1	13816	39,2	11	0,82	1,46
O75348	V-type proton ATPase subunit G 1 OS=Homo sapiens OX=9606 GN=ATP6V1G1 PE=1 SV=3	1	1	13863	12,25	6	0,35	1,45
P10606	Cytochrome c oxidase subunit 5B, mitochondrial OS=Homo sapiens OX=9606 GN=COX5B PE=1 SV=2	1	1	13915	38,4	25	9,87	2,29
O95298	NADH dehydrogenase [ubiquinone] 1 subunit C2 OS=Homo sapiens OX=9606 GN=NDUFC2 PE=1 SV=1	0	1	14235	24,4	3		0,79

P82932	28S ribosomal protein S6, mitochondrial OS=Homo sapiens OX=9606 GN=MRPS6 PE=1 SV=3	1	1	14275	25,6	9	0,78	1,38
P62805	Histone H4 OS=Homo sapiens OX=9606 GN=H4C1 PE=1 SV=2	1	1	14342	40,75	14	5,14	3,27
P05496	ATP synthase F(0) complex subunit C1, mitochondrial OS=Homo sapiens OX=9606 GN=ATP5MC1 PE=1 SV=2	0	1	14439	27,9	4		0,78
Q5JTJ3	Cytochrome c oxidase assembly factor 6 homolog OS=Homo sapiens OX=9606 GN=COA6 PE=1 SV=1	1	1	14449	11,2	2	0,33	0,33
Q8N6L1	Keratinocyte-associated protein 2 OS=Homo sapiens OX=9606 GN=KRTCAP2 PE=1 SV=2	0	1	14840	12,5	2		0,32
P25398	40S ribosomal protein S12 OS=Homo sapiens OX=9606 GN=RPS12 PE=1 SV=3	0	1	14905	13,6	2		0,32
Q9NPJ3	Acyl-coenzyme A thioesterase 13 OS=Homo sapiens OX=9606 GN=ACOT13 PE=1 SV=1	1	1	15065	22,15	9	2	0,73
Q7Z7F7	39S ribosomal protein L55, mitochondrial OS=Homo sapiens OX=9606 GN=MRPL55 PE=1 SV=1	1	1	15119	37,5	20	4,2	4,2
P56556	NADH dehydrogenase [ubiquinone] 1 alpha subcomplex subunit 6 OS=Homo sapiens OX=9606 GN=NDUFA6 PE=1 SV=4	1	1	15127	16,4	8	0,32	2,95
Q4U2R6	39S ribosomal protein L51, mitochondrial OS=Homo sapiens OX=9606 GN=MRPL51 PE=1 SV=1	0	1	15199	14,8	2		0,73
P07737	Profilin-1 OS=Homo sapiens OX=9606 GN=PFN1 PE=1 SV=2	1	1	15216	40,4	19	1,27	2,91
O60783	28S ribosomal protein S14, mitochondrial OS=Homo sapiens OX=9606 GN=MRPS14 PE=1 SV=1	0	1	15243	21,9	5		0,73
O95168	NADH dehydrogenase [ubiquinone] 1 beta subcomplex subunit 4 OS=Homo sapiens OX=9606 GN=NDUFB4 PE=1 SV=3	0	1	15256	31,8	7		1,27
Q8IXM3	39S ribosomal protein L41, mitochondrial OS=Homo sapiens OX=9606 GN=MRPL41 PE=1 SV=1	1	1	15430	28,85	9	1,24	1,93
Q01469	Fatty acid-binding protein 5 OS=Homo sapiens OX=9606 GN=FABP5 PE=1 SV=3	1	1	15497	15,95	12	1,23	1,91
Q9Y3D3	28S ribosomal protein S16, mitochondrial OS=Homo sapiens OX=9606 GN=MRPS16 PE=1 SV=1	0	1	15563	19	2		0,71
Q9Y6H1	Coiled-coil-helix-coiled-coil-helix domain-containing protein 2 OS=Homo sapiens OX=9606 GN=CHCHD2 PE=1 SV=1	1	0	15731	33,8	3	0,69	
Q9NZT1	Calmodulin-like protein 5 OS=Homo sapiens OX=9606 GN=CALML5 PE=1 SV=2	1	1	15883	25	11	1,19	0,69
Q6GMV3	Putative peptidyl-tRNA hydrolase PTRHD1 OS=Homo sapiens OX=9606 GN=PTRHD1 PE=1 SV=1	1	0	15909	11,4	2	0,3	
Q9P0M9	39S ribosomal protein L27, mitochondrial OS=Homo sapiens OX=9606 GN=MRPL27 PE=1 SV=1	1	1	16120	20,9	5	0,68	0,68
Q6P1L8	39S ribosomal protein L14, mitochondrial OS=Homo sapiens OX=9606 GN=MRPL14 PE=1 SV=1	1	1	16165	31,7	13	1,79	1,79
Q9Y3D5	28S ribosomal protein S18c, mitochondrial OS=Homo sapiens OX=9606 GN=MRPS18C PE=1 SV=1	1	1	16238	12,7	5	0,29	1,16
Q86SX6	Glutaredoxin-related protein 5, mitochondrial OS=Homo sapiens OX=9606 GN=GLRX5 PE=1 SV=2	0	1	16732	28	4		1,1
P20674	Cytochrome c oxidase subunit 5A, mitochondrial OS=Homo sapiens OX=9606 GN=COX5A PE=1 SV=2	1	1	16923	49,35	32	14,06	1,68
Q9Y6G3	39S ribosomal protein L42, mitochondrial OS=Homo sapiens OX=9606 GN=MRPL42 PE=1 SV=1	0	1	16935	21,8	4		0,63
P60660	Myosin light polypeptide 6 OS=Homo sapiens OX=9606 GN=MYL6 PE=1 SV=2	1	1	17090	14,39	5	0,28	1,65
Q9BX68	Adenosine 5~-monophosphoramidase HINT2 OS=Homo sapiens OX=9606 GN=HINT2 PE=1 SV=1	1	1	17208	38,05	20	3,27	1,63
Q04837	Single-stranded DNA-binding protein, mitochondrial OS=Homo sapiens OX=9606 GN=SSBP1 PE=1 SV=1	1	1	17249	40,55	15	4,44	0,27
P30049	ATP synthase subunit delta, mitochondrial OS=Homo sapiens OX=9606 GN=ATP5F1D PE=1 SV=2	1	1	17479	36,9	112	1,05	4,32
O14561	Acyl carrier protein, mitochondrial OS=Homo sapiens OX=9606 GN=NDUFAB1 PE=1 SV=3	1	1	17577	14,35	8	0,27	1,04
P62979	Ubiquitin-40S ribosomal protein S27a OS=Homo sapiens OX=9606 GN=RPS27A PE=1 SV=2	1	1	18296	21,5	30	0,98	2,12
Q8N5N7	39S ribosomal protein L50, mitochondrial OS=Homo sapiens OX=9606 GN=MRPL50 PE=1 SV=2	1	1	18484	20,9	10	0,57	1,46
P46783	40S ribosomal protein S10 OS=Homo sapiens OX=9606 GN=RPS10 PE=1 SV=1	1	1	18886	19	5	0,25	0,56
Q969H8	Myeloid-derived growth factor OS=Homo sapiens OX=9606 GN=MYDGF PE=1 SV=1	1	1	18897	20,75	9	0,28	2,02
Q13405	39S ribosomal protein L49, mitochondrial OS=Homo sapiens OX=9606 GN=MRPL49 PE=1 SV=1	0	1	19243	37,3	6		1,96

Q9BU61	NADH dehydrogenase [ubiquinone] 1 alpha subcomplex assembly factor 3 OS=Homo sapiens OX=9606 GN=NDUFAF3 PE=1 SV=1	0	1	20566	12	2		0,5
