# Supplementary material for: ATP synthase interactome analysis identifies a new subunit l as a modulator of permeability transition pore in yeast
Source: Sci Rep. 2023 Mar 7;13:3839. doi: 10.1038/s41598-023-30966-5 (PMC9992712; doi:10.1038/s41598-023-30966-5)
Supplement: Supplementary file 4 — Supplementary Information 4. [file 41598_2023_30966_MOESM4_ESM.pdf]

Protein complex and processes	Uniprot_Accession	Description
Ribosomal Proteins and Histones	Q9Y291	28S ribosomal protein S33, mitochondrial OS=Homo sapiens OX=9606 GN=MRPS33 PE=1 SV=1
	P82932	28S ribosomal protein S6, mitochondrial OS=Homo sapiens OX=9606 GN=MRPS6 PE=1 SV=3
	Q6P1L8	39S ribosomal protein L14, mitochondrial OS=Homo sapiens OX=9606 GN=MRPL14 PE=1 SV=1
	Q9P0M9	39S ribosomal protein L27, mitochondrial OS=Homo sapiens OX=9606 GN=MRPL27 PE=1 SV=1
	Q9BQ48	39S ribosomal protein L34, mitochondrial OS=Homo sapiens OX=9606 GN=MRPL34 PE=1 SV=1
	Q8IXM3	39S ribosomal protein L41, mitochondrial OS=Homo sapiens OX=9606 GN=MRPL41 PE=1 SV=1
	Q9Y6G3	39S ribosomal protein L42, mitochondrial OS=Homo sapiens OX=9606 GN=MRPL42 PE=1 SV=1
	Q13405	39S ribosomal protein L49, mitochondrial OS=Homo sapiens OX=9606 GN=MRPL49 PE=1 SV=1
	Q8N5N7	39S ribosomal protein L50, mitochondrial OS=Homo sapiens OX=9606 GN=MRPL50 PE=1 SV=2
	Q4U2R6	39S ribosomal protein L51, mitochondrial OS=Homo sapiens OX=9606 GN=MRPL51 PE=1 SV=1
	Q96EL3	39S ribosomal protein L53, mitochondrial OS=Homo sapiens OX=9606 GN=MRPL53 PE=1 SV=1
	Q7Z7F7	39S ribosomal protein L55, mitochondrial OS=Homo sapiens OX=9606 GN=MRPL55 PE=1 SV=1
	P46783	40S ribosomal protein S10 OS=Homo sapiens OX=9606 GN=RPS10 PE=1 SV=1
	P25398	40S ribosomal protein S12 OS=Homo sapiens OX=9606 GN=RPS12 PE=1 SV=3
	P60866	40S ribosomal protein S20 OS=Homo sapiens OX=9606 GN=RPS20 PE=1 SV=1
	P63220	40S ribosomal protein S21 OS=Homo sapiens OX=9606 GN=RPS21 PE=1 SV=1
	P62851	40S ribosomal protein S25 OS=Homo sapiens OX=9606 GN=RPS25 PE=1 SV=1
	Q71UM5	40S ribosomal protein S27-like OS=Homo sapiens OX=9606 GN=RPS27L PE=1 SV=3
	P62857	40S ribosomal protein S28 OS=Homo sapiens OX=9606 GN=RPS28 PE=1 SV=1
	P05387	60S acidic ribosomal protein P2 OS=Homo sapiens OX=9606 GN=RPLP2 PE=1 SV=1
	P63173	60S ribosomal protein L38 OS=Homo sapiens OX=9606 GN=RPL38 PE=1 SV=2
	Q9BQC6	Ribosomal protein 63, mitochondrial OS=Homo sapiens OX=9606 GN=MRPL57 PE=1 SV=1
	P62979	Ubiquitin-40S ribosomal protein S27a OS=Homo sapiens OX=9606 GN=RPS27A PE=1 SV=2
	P0C0S5	Histone H2A.Z OS=Homo sapiens OX=9606 GN=H2AZ1 PE=1 SV=2
	Q5QNW6	Histone H2B type 2-F OS=Homo sapiens OX=9606 GN=H2BC18 PE=1 SV=3
	P62805	Histone H4 OS=Homo sapiens OX=9606 GN=H4C1 PE=1 SV=2
ATP Synthase Complex	P05496	ATP synthase F(0) complex subunit C1, mitochondrial OS=Homo sapiens OX=9606 GN=ATP5MC1 PE=1 SV=2
	Q96IX5	ATP synthase membrane subunit K, mitochondrial OS=Homo sapiens OX=9606 GN=ATP5MK PE=1 SV=1
	P03928	ATP synthase protein 8 OS=Homo sapiens OX=9606 GN=MT-ATP8 PE=1 SV=1
	P56378	ATP synthase subunit ATP5MJ, mitochondrial OS=Homo sapiens OX=9606 GN=ATP5MJ PE=1 SV=1
	P30049	ATP synthase subunit delta, mitochondrial OS=Homo sapiens OX=9606 GN=ATP5F1D PE=1 SV=2
	P56385	ATP synthase subunit e, mitochondrial OS=Homo sapiens OX=9606 GN=ATP5ME PE=1 SV=2
	Q5VTU8	ATP synthase subunit epsilon-like protein, mitochondrial OS=Homo sapiens OX=9606 GN=ATP5F1EP2 PE=1 SV=1
	P56134	ATP synthase subunit f, mitochondrial OS=Homo sapiens OX=9606 GN=ATP5MF PE=1 SV=3
	O75964	ATP synthase subunit g, mitochondrial OS=Homo sapiens OX=9606 GN=ATP5MG PE=1 SV=3
	P18859	ATP synthase-coupling factor 6, mitochondrial OS=Homo sapiens OX=9606 GN=ATP5PF PE=1 SV=1
	Q9UII2	ATPase inhibitor, mitochondrial OS=Homo sapiens OX=9606 GN=ATP5IF1 PE=1 SV=1
Complex IV and Cyt C	Q9NRP2	COX assembly mitochondrial protein 2 homolog OS=Homo sapiens OX=9606 GN=CMC2 PE=1 SV=1

	P99999	Cytochrome c OS=Homo sapiens OX=9606 GN=CYCS PE=1 SV=2
	Q9Y2R0	Cytochrome c oxidase assembly factor 3 homolog, mitochondrial OS=Homo sapiens OX=9606 GN=COA3 PE=1 SV=1
	Q5JTJ3	Cytochrome c oxidase assembly factor 6 homolog OS=Homo sapiens OX=9606 GN=COA6 PE=1 SV=1
	Q49B96	Cytochrome c oxidase assembly protein COX19 OS=Homo sapiens OX=9606 GN=COX19 PE=1 SV=1
	Q14061	Cytochrome c oxidase copper chaperone OS=Homo sapiens OX=9606 GN=COX17 PE=1 SV=2
	P20674	Cytochrome c oxidase subunit 5A, mitochondrial OS=Homo sapiens OX=9606 GN=COX5A PE=1 SV=2
	P10606	Cytochrome c oxidase subunit 5B, mitochondrial OS=Homo sapiens OX=9606 GN=COX5B PE=1 SV=2
	P12074	Cytochrome c oxidase subunit 6A1, mitochondrial OS=Homo sapiens OX=9606 GN=COX6A1 PE=1 SV=4
	P14854	Cytochrome c oxidase subunit 6B1 OS=Homo sapiens OX=9606 GN=COX6B1 PE=1 SV=2
	P09669	Cytochrome c oxidase subunit 6C OS=Homo sapiens OX=9606 GN=COX6C PE=1 SV=2
	P14406	Cytochrome c oxidase subunit 7A2, mitochondrial OS=Homo sapiens OX=9606 GN=COX7A2 PE=1 SV=1
	O14548	Cytochrome c oxidase subunit 7A-related protein, mitochondrial OS=Homo sapiens OX=9606 GN=COX7A2L PE=1 SV=2
	P24311	Cytochrome c oxidase subunit 7B, mitochondrial OS=Homo sapiens OX=9606 GN=COX7B PE=1 SV=2
	P15954	Cytochrome c oxidase subunit 7C, mitochondrial OS=Homo sapiens OX=9606 GN=COX7C PE=1 SV=1
	P10176	Cytochrome c oxidase subunit 8A, mitochondrial OS=Homo sapiens OX=9606 GN=COX8A PE=1 SV=2
	O00483	Cytochrome c oxidase subunit NDUFA4 OS=Homo sapiens OX=9606 GN=NDUFA4 PE=1 SV=1
Complex III	A0A096LP55	Cytochrome b-c1 complex subunit 6-like, mitochondrial OS=Homo sapiens OX=9606 GN=UQCRHL PE=3 SV=1
	P14927	Cytochrome b-c1 complex subunit 7 OS=Homo sapiens OX=9606 GN=UQCRB PE=1 SV=2
	O14949	Cytochrome b-c1 complex subunit 8 OS=Homo sapiens OX=9606 GN=UQCRQ PE=1 SV=4
	Q9UDW1	Cytochrome b-c1 complex subunit 9 OS=Homo sapiens OX=9606 GN=UQCR10 PE=1 SV=3
Complex I	Q9BU61	NADH dehydrogenase [ubiquinone] 1 alpha subcomplex assembly factor 3 OS=Homo sapiens OX=9606 GN=NDUFAF3 PE=1 SV=1
	O43678	NADH dehydrogenase [ubiquinone] 1 alpha subcomplex subunit 2 OS=Homo sapiens OX=9606 GN=NDUFA2 PE=1 SV=3
	O95167	NADH dehydrogenase [ubiquinone] 1 alpha subcomplex subunit 3 OS=Homo sapiens OX=9606 GN=NDUFA3 PE=1 SV=1
	Q16718	NADH dehydrogenase [ubiquinone] 1 alpha subcomplex subunit 5 OS=Homo sapiens OX=9606 GN=NDUFA5 PE=1 SV=3
	P56556	NADH dehydrogenase [ubiquinone] 1 alpha subcomplex subunit 6 OS=Homo sapiens OX=9606 GN=NDUFA6 PE=1 SV=4
	O95182	NADH dehydrogenase [ubiquinone] 1 alpha subcomplex subunit 7 OS=Homo sapiens OX=9606 GN=NDUFA7 PE=1 SV=3
	O75438	NADH dehydrogenase [ubiquinone] 1 beta subcomplex subunit 1 OS=Homo sapiens OX=9606 GN=NDUFB1 PE=1 SV=1
	O43676	NADH dehydrogenase [ubiquinone] 1 beta subcomplex subunit 3 OS=Homo sapiens OX=9606 GN=NDUFB3 PE=1 SV=3
	O95168	NADH dehydrogenase [ubiquinone] 1 beta subcomplex subunit 4 OS=Homo sapiens OX=9606 GN=NDUFB4 PE=1 SV=3
	O43677	NADH dehydrogenase [ubiquinone] 1 subunit C1, mitochondrial OS=Homo sapiens OX=9606 GN=NDUFC1 PE=1 SV=1
	O95298	NADH dehydrogenase [ubiquinone] 1 subunit C2 OS=Homo sapiens OX=9606 GN=NDUFC2 PE=1 SV=1
	P56181	NADH dehydrogenase [ubiquinone] flavoprotein 3, mitochondrial OS=Homo sapiens OX=9606 GN=NDUFV3 PE=1 SV=2
	O43920	NADH dehydrogenase [ubiquinone] iron-sulfur protein 5 OS=Homo sapiens OX=9606 GN=NDUFS5 PE=1 SV=3
TIM/TOM Complex	Q9Y5J6	Mitochondrial import inner membrane translocase subunit Tim10 B OS=Homo sapiens OX=9606 GN=TIMM10B PE=1 SV=1
	P62072	Mitochondrial import inner membrane translocase subunit Tim10 OS=Homo sapiens OX=9606 GN=TIMM10 PE=1 SV=1
	Q9Y5L4	Mitochondrial import inner membrane translocase subunit Tim13 OS=Homo sapiens OX=9606 GN=TIMM13 PE=1 SV=1
	Q96DA6	Mitochondrial import inner membrane translocase subunit TIM14 OS=Homo sapiens OX=9606 GN=DNAJC19 PE=1 SV=3
	Q9Y3D7	Mitochondrial import inner membrane translocase subunit TIM16 OS=Homo sapiens OX=9606 GN=PAM16 PE=1 SV=2
	O60220	Mitochondrial import inner membrane translocase subunit Tim8 A OS=Homo sapiens OX=9606 GN=TIMM8A PE=1 SV=1
	Q9Y5J7	Mitochondrial import inner membrane translocase subunit Tim9 OS=Homo sapiens OX=9606 GN=TIMM9 PE=1 SV=1

	Q8N4H5	Mitochondrial import receptor subunit TOM5 homolog OS=Homo sapiens OX=9606 GN=TOMM5 PE=1 SV=1
	Q96B49	Mitochondrial import receptor subunit TOM6 homolog OS=Homo sapiens OX=9606 GN=TOMM6 PE=1 SV=1
	Q9P0U1	Mitochondrial import receptor subunit TOM7 homolog OS=Homo sapiens OX=9606 GN=TOMM7 PE=1 SV=1
S100 Calcium binding complex	P31151	Protein S100-A7 OS=Homo sapiens OX=9606 GN=S100A7 PE=1 SV=4
	Q86SG5	Protein S100-A7A OS=Homo sapiens OX=9606 GN=S100A7A PE=1 SV=3
	P05109	Protein S100-A8 OS=Homo sapiens OX=9606 GN=S100A8 PE=1 SV=1
	P06702	Protein S100-A9 OS=Homo sapiens OX=9606 GN=S100A9 PE=1 SV=1
Apoptosis (UP_KW)	P61803	Dolichyl-diphosphooligosaccharide--protein glycosyltransferase subunit DAD1 OS=Homo sapiens OX=9606 GN=DAD1 PE=1 SV=3
	P63167	Dynein light chain 1, cytoplasmic OS=Homo sapiens OX=9606 GN=DYNLL1 PE=1 SV=1
	Q96FJ2	Dynein light chain 2, cytoplasmic OS=Homo sapiens OX=9606 GN=DYNLL2 PE=1 SV=1
	Q9BX68	Adenosine 5~-monophosphoramidase HINT2 OS=Homo sapiens OX=9606 GN=HINT2 PE=1 SV=1
	Q969H8	Myeloid-derived growth factor OS=Homo sapiens OX=9606 GN=MYDGF PE=1 SV=1
Redox and Fe-S cluster	P10599	Thioredoxin OS=Homo sapiens OX=9606 GN=TXN PE=1 SV=3
	P35754	Glutaredoxin-1 OS=Homo sapiens OX=9606 GN=GLRX PE=1 SV=2
	Q86SX6	Glutaredoxin-related protein 5, mitochondrial OS=Homo sapiens OX=9606 GN=GLRX5 PE=1 SV=2
Complex I and III LYR Proteins	Q9NU23	LYR motif-containing protein 2 OS=Homo sapiens OX=9606 GN=LYRM2 PE=1 SV=1
	Q9HD34	LYR motif-containing protein 4 OS=Homo sapiens OX=9606 GN=LYRM4 PE=1 SV=1
	Q5U5X0	Complex III assembly factor LYRM7 OS=Homo sapiens OX=9606 GN=LYRM7 PE=1 SV=1
Sec61 Translocation complex	P60468	Protein transport protein Sec61 subunit beta OS=Homo sapiens OX=9606 GN=SEC61B PE=1 SV=2
	P60059	Protein transport protein Sec61 subunit gamma OS=Homo sapiens OX=9606 GN=SEC61G PE=1 SV=1
