# Supplementary material for: ATP synthase interactome analysis identifies a new subunit l as a modulator of permeability transition pore in yeast
Source: Sci Rep. 2023 Mar 7;13:3839. doi: 10.1038/s41598-023-30966-5 (PMC9992712; doi:10.1038/s41598-023-30966-5)
Supplement: Supplementary file 5 — Supplementary Information 5. [file 41598_2023_30966_MOESM5_ESM.pdf]

atp19 and mco10 related protein sequences in fungal genomes. Highlighted in red are where only one paralogs were found from the database.

Saccharomycotina (Subphylum)

Saccharomycetes (Class)

Saccharomycetales (Order)

Alloascoideaceae (Family)

CUG-Ser1 clade (Clade)

Cephaloascaceae (Family)

Debaryomycetaceae (Family)

Candida albicans SC5314

>XP_019330709.1 F1F0 ATP synthase subunit k [Candida albicans SC5314]
MGAAYQIFGKTFQPHQLALATLGSVVLLVLPKPWGPPSPTTPPIKASSPEEEKFIQEWLAKHTEEKH

>XP_019330759.1 hypothetical protein CAALFM_C203210WA [Candida albicans SC5314]
MAGAYTIFGKQVPAHILSIITLGSVAAGIAIPKFLPKDETKKEVAKPVAPIVQSKEDDFDLEKFINDLTKEESK

Meyerozyma sp. JA9

>RLV85501.1 hypothetical protein JA9_001681 [Meyerozyma sp. JA9]
MGAAYTIFGKQVPSHILSLLTLGSVAGVVAWPRSKSEPAATPAPVAAAASNKEEDFDLEKFIK

>RLV85829.1 hypothetical protein JA9_001827 [Meyerozyma sp. JA9]
MGAAYQILGKSVPSHYLSLATLGAVVLTVAPKPWGPGAPAHPQISASSPEEEKYVKEFLAKHAEKH

Candida parapsilosis

>XP_036668234.1 uncharacterized protein CPAR2_700780 [Candida parapsilosis]
MGAAYHIFGKTVQPHQLALATLGSVVLLVIPKPWTVKPAHPSINASSPEEDKFVKEWLAKHDVAV

>XP_036666693.1 uncharacterized protein CPAR2_211700 [Candida parapsilosis]
MGSAYTILGKQVPAHVLSILTLGSVAAGVAIPKVLGSNDAKKNVPAKPAPPVAQSKEDDFDLEKFINELTKEETK

Yamadazyma tenuis ATCC 10573

>XP_006684611.1 uncharacterized protein CANTEDRAFT_112898, partial [Yamadazyma tenuis ATCC 10573]
MGAAYTIFGKQVPSHILAILTLGSVAGVVAYPRSKSEATPTPTPAPAVTVASKEDDFDLEKFINDLSKEET

>XP_006685526.1 uncharacterized protein CANTEDRAFT_103812 [Yamadazyma tenuis ATCC 10573]
MAAAYTILGKSVPSHQLAIATLGAVAFVCIPKPWAPPAPAHPPTNASSAEEEKFIKEFLSKNQAEKH

Spathaspora sp. JA1

>RLV95954.1 hypothetical protein JA1_000592 [Spathaspora sp. JA1]
MGAAYTVFGKKVPAHILSIATLSSVAAFTTWSLVGPKGETKAVATPVAPISQSKEEDFDLEKILSDLTKEEAK

>RLV94766.1 hypothetical protein JA1_001611 [Spathaspora sp. JA1]
MGAAYTLLGKSFQPHQLAIATLGTVAYFIAPKPWASKEAPAGPAINASSPEEEKFVKEWLAKHAAEEKH

Debaryomyces hansenii CBS767

>XP_461789.1 DEHA2G05588p [Debaryomyces hansenii CBS767]

MGAAYTIFGRQVPSHILSIATLGTAAAVVAWPRAKKEAPPAQAAPVAQTKEEDFDLEKFLKYVYTD

>XP_461967.1 DEHA2G09680p [Debaryomyces hansenii CBS767]
MGAYSILGKSVPSHKLALATLGSVVLVVAPKPWGPPAPNHPQINASSKEEEKFVQEWLAKHSAEEKH

Candida metapsilosis

>KAG5418293.1 ATP19 [Candida metapsilosis]
MGAAYHILGRTVQPHQLALATLGSVVLLVIPKPWTVKPTHPSINASSPEEEKFVKEWLAKHEKVEEKH

>KAG5417157.1 hypothetical protein I9W82_004790 [Candida metapsilosis]
MGSAYTIFGKQVPAHVLSILTLGSVVAGVTIPKFFGSADANKDAAAKPAAPVVQSKEDDFDLEKFIKYVPSFEQPVLCGPQY

Spathaspora passalidarum NRRL Y-27907

>XP_007375798.1 hypothetical protein SPAPADRAFT_61584 [Spathaspora passalidarum NRRL Y-27907]
MGAAYTILGRTFQPHQLAIATLGAVVYLVAPKPWAAKEVSAGPAINASSPEEEKFVKEWLAKHTAEEKH

>XP_007374975.1 hypothetical protein SPAPADRAFT_60813 [Spathaspora passalidarum NRRL Y-27907]
MGAAYTILGKQVPAHILSIATLSAVAAGTTWSLMGPKTEAKAAPVAPVSQSKEEDFDFEKIFNDLTKEEAK

Candida orthopsilosis Co 90-125

>XP_003870909.1 Atp19 h [Candida orthopsilosis Co 90-125]
MGAAYHIFGKTVQPHQLALATLGSVVLLVIPKPWTVKPVHPSINASSPEEEKFVKEWLAKHEKTEEKH

>XP_003866927.1 hypothetical protein CORT_0A11040 [Candida orthopsilosis Co 90-125]
MGSAYTILGKQVPAHVLSILTLGSVAAGVAIPKLLRSNDVKKEAVAMPAAPVAQSKEDDFDLEKFLNDLTKEETK

Metschnikowiaceae (Family)

Clavispora lusitaniae

>KAF5212165.1 hypothetical protein E0198_001721 [Clavispora lusitaniae]
MGAAYTIFGKQVQPHILSILTLGSVALIAAWPREKTEKPAAPAPAVASKDEEFDLEKIISDFTKDEAK

>OVF07739.1 putative f1F0 ATP synthase subunit [Clavispora lusitaniae]
MVYRTAYIPDQLGSALSRLTHEKLTVESNFFSQSTTMGAAYTILGRSVQPHVLALATLGAVVFVAAPKPWGPPKPTHPAIGASSPEEEKFVKDFLAKHLEEKH

Metschnikowia persimmonesis

>KAF8003343.1 hypothetical protein HF325_002588 [Metschnikowia persimmonesis]
MGAAYTIFGKQVQPHILSLLTLGSVAGIVAWPRSVSAEKPAAPAPAAAQDDEFDLEKIISDFTKEETK

>KAF8004395.1 hypothetical protein HF325_001843 [Metschnikowia persimmonesis]
MGAAYTILGRSVQPNILALATLGSVVFVVAPKPWGPAKPVHPPINAASPEEEKFVQDFLAKHAEKH

[Candida] auris

>QRG39994.1 hypothetical protein FDK38_004455 [[Candida] auris]
MAGAEYKIFGRPVKPHVLSLLTLGAVVGIALWPEQNKAKKEFVDQTTKGPKPGDKDEEFDFQKIINDFTKDEK

>GBL47796.1 hypothetical protein CAJCM15448_00700 [[Candida] auris]
MGAAYQILGKSVQPHVLSLATLGLVAFVAMPKPWAINASSKEEEKFVQDFLAKHLEKSAEKH

Ascoideaceae (Family)

Ascoidea rubescens DSM 1968

>XP_020044616.1 hypothetical protein ASCRUDRAFT_78005 [Ascoidea rubescens DSM 1968]
MVSYYTVLGKKVPSHILAIATIVATVGGIALPKYIPSSKSESPKVVPPPVETSASKDEEFDFEKLIGDFLKEEQK

Dipodascaceae (Family)

Yarrowia lipolytica CLIB122

>XP_505986.1 YALI0F28347p [Yarrowia lipolytica CLIB122]
MFADYVNTVKTDYEHQIAQLEKQVTALKMGEQVDSISVTDPRENLPTFPLPIQRQKFLELVDRDKTNQIYVYSGEVTESPALWASRIENIMKTFNFSTCILETSQVVSKLLGGKALLLYRKQKNQVLPWVELKRLVHTLD
KPVLRSIAVHKELAKLEDTDLPIEYRIKMIRHWEPQLDPSPLGDRVVEVIRIIPEKEADIMKAVEGGISSFDQLLGIMGPKDESSHQPQKAEEQPPVLSYKQKRAAKDVRDKTCTYCHKEGHKSSQCFKRPKR

>XP_002143014.1 YALI0B11913p [Yarrowia lipolytica CLIB122]
MGAAYHILGKTVYPHQLAIGTIVSVVGGIVIASSGKKAEKPAAPAIQAGSSDEEKFIANFLKEQEAAEKK

Endomycetaceae (Family)

Lipomycetaceae (Family)

Phaffomycetaceae (Family)

Komagataella phaffii CBS 7435

>SOP82886.1 ATP synthase K chain, mitochondrial [Komagataella phaffii CBS 7435]
MGANYVIFGKTVRPHVVTLATLGSVAAGIAIPKFLPKDEPIQQANTPIQSAVETVKDGAAKAAETVKAGSDKVTSVVTGNSDDIDVEKLINDFVTSVDDRKQ

>XP_002492367.1 Hypothetical protein PAS_chr3_0161 [Komagataella phaffii GS115]
MGAAYTILGKTFQPHQLALATIGLVTLLAIPKPGGAKKETTPQINASSPEEEAFIKEYLAKHDQKHD

Cyberlindnera jadinii NRRL Y-1542

>XP_020072292.1 hypothetical protein CYBJADRAFT_166007 [Cyberlindnera jadinii NRRL Y-1542]
MGSAYTIFGKQVSSHWIAIATLSAALGLGVVSSSGSKAEASPKATPVPAAPVAEKSEDFDVEKLLNDFLKDDEEKK

>XP_020068349.1 hypothetical protein CYBJADRAFT_169532 [Cyberlindnera jadinii NRRL Y-1542]
MGSAYTILGRTVPAHQIAIGTLSAALLLAIPNPFAAAVKPTPKIEASSPEEKKFIEDYLKQAEAKAAEKH

Pichiaceae (Family)

Ogataea angusta

>sp|C0HK66|ATP19_PICAN ATP synthase subunit K, mitochondrial OS=Pichia angusta OX=870730 GN=ATP19 PE=1 SV=1
MAGAYTLFGKAIPPHHLAIATIGTVVALVAPKPWSPKVKLEPKIDASSPEEEKFIKEYLEKHL

>sp|C0HK67|ATPLN_PICAN ATP synthase subunit L OS=Pichia angusta OX=870730 PE=1 SV=1
MAAPYVIFGAKVPPHWLAIGTILTVVGGIYGPKAFSSPAAAAPAATPAAAPSSEPELDVEKAINDFLASDKKE

Pichia kudriavzevii CAB39-6420

>Pichia kudriavzevii_XP_029319699.1 uniprot ID: BOH78_2882
MMVSQGSSSWEFTYQHRHTGKPSTETKITMGAAYNILGKLVPPHYLAIGTILTVVGGVQLATLGGDKESAPAAAPKAPATSSSKDGELDVEKAIADFLASNEKA

>tr|A0A1V2LJW6|A0A1V2LJW6_PICKU ATP synthase subunit K, mitochondrial OS=Pichia kudriavzevii OX=4909 GN=BOH78_3494 PE=4 SV=1
MGANYNLLGRSIPPHYLSLATIGAVVLLAAPKPWAPAVKKEAEINASSKEEEAFKVITWSMNGLLFLACGGTPNWWIKSSLVNCQCGSMSNDLSKDKIGLDPLRQLPVKLSSSSVCRFITWNFTVGPFGGLESHM

Pichia membranifaciens

>GAV29134.1 hypothetical protein PMKS-002614 [Pichia membranifaciens]
MGAAYNILGKTVQPHWLAIGTIATVVGGANVNSILSAFGAAPAAAPAPTAATTPATASGGEFDVEKAINDFLASSDEKKEA

>XP_019015785.1 hypothetical protein PICMEDRAFT_18075 [Pichia membranifaciens NRRL Y-2026]
MGAAYNILGKAVPPHQLALGTIGAVVLLVLPKPWATTPKKEAKIDAANADEEKFIKAYLEKHA

Saccharomycetaceae (Family)

Lachancea mirantina

>SCU84269.1 LAMI_0C06876g1_1 [Lachancea mirantina]
MGAAYHILGRTVQPHQLSIATISAVVLLALPNPFATKVPKKPEVKAASAEEEKFIANYIKEHTAKAEKH

>SCV02746.1 LAMI_0H02608g1_1 [Lachancea mirantina]
MGAAYHILGRTVQPHTLAIATIAATVGGAVYSMTGSKPEKTQETSVDSSQKTTQSEPDIDIEKLVDQFIRDEKQ

Kazachstania saulgeensis

>SMN22890.1 similar to Saccharomyces cerevisiae YOL077W-A ATP19 Subunit k [Kazachstania saulgeensis]
MGGAYKIFGRTFQPHQLAIATLTTVAVIAAPNPFAAKKPKVAEIKAESAEEEAFIKTYIEKHSAEAK

>SMN17852.1 similar to Saccharomyces cerevisiae YOR020W-A Putative protein of unknown function [Kazachstania saulgeensis]
MGAAYRIFGKTFQPHQLAIATLLTVGIVGFTMTRKGGKSTIDPNIQLQPQRGVSGSQKSDNDDINVEKLLTELIEENDETVN

Kluyveromyces marxianus

>KAG0678637.1 hypothetical protein C6P43_000949 [Kluyveromyces marxianus]
MSGAYTILGRTVQPHQLALGTLGAVLLLVVPNPFKSKPAAKPLFTTSSKEEEDFIKAYLDKHVTNAEKH

>KAG0684191.1 hypothetical protein C6P41_002468 [Kluyveromyces marxianus]
MYEKFQDFTEILLREIFNHLNNKYSKMSGAYTILGRTVQPHQLALGTLGAVLLLVVPNPFKSKPAAKPLFTTSSKEEEDFIKAYLDKHVTNAEKH

Zygosaccharomyces parabailii

>AQZ17609.1 YOR020W-A [Zygosaccharomyces parabailii]
MGNAYTIFGKQVQPHFLAILTLGTAAGLGVWASTGKKDEAKTSAASKDNEEINVEKMIDDFISKESIDK

>AQZ11499.1 ATP19 (YOL077W-A) [Zygosaccharomyces parabailii]
MGHAYQILGRTCQPHQIAIATLGFVALLAAPNPFSKKQPKTVDFSASSPEEEKFIKEYVQKHSKAEH

Torulaspora delbrueckii

>XP_003681290.1 hypothetical protein TDEL_0D04950 [Torulaspora delbrueckii]
MGAGYQILGRHVPSHQLALGTLGLVALIVAPNPFAAKKPKSVDFNAGSKEEEQFIQNYLAKHAEAKEEH

>XP_003679015.1 hypothetical protein TDEL_0A04720 [Torulaspora delbrueckii]
MSPAYKILGMSVQPHVLAIGTLLATGAGVYLGTGKNEDQNKAESKEPVKQGESEVDVEKLLNQFLESETEKK

Zygotorulaspora mrakii

>XP_037143687.1 uncharacterized protein HG535_0C03110 [Zygotorulaspora mrakii]
MGAAYHIMGRTVPSHFLALGTIGLVALIAIPNPFSSKKGESIDFNAGSKDEEKFIKEYLAKHAGAPHKQ

>XP_037146627.1 uncharacterized protein HG535_0H02290 [Zygotorulaspora mrakii]
MGQAYKIFGMAVKPHYLAIATLLGTFGGAAYFTGGGSSTDNSNKIAIDEKVNQTDVSSENIDVEKLLDNLLKDSNEEKK

Saccharomyces cerevisiae

>sp|Q3E824|YO020_YEAST Uncharacterized protein YOR020W-A OS=Saccharomyces cerevisiae

MGAAYKVFGKTVQPHVLAISTFIATAAVASYFTTKPKTKNEGKNSSALSQQKSGESSNSDAMGKDDDVVKSIEGFLNDLEKDTRQDTKAN

>sp|P81451|ATP19_YEAST ATP synthase subunit K, mitochondrial OS=Saccharomyces cerevisiae
MGAAYHFMGKAIPPHQLAIGTLGLLGLLVVPNPFKSAKPKTVDIKTDNKDEEKFIENYLKKHSEKQDA

Saccharomycodaceae (Family)

Hanseniaspora osmophila

>OEJ80327.1 ATP synthase subunit K, mitochondrial [Hanseniaspora osmophila]
MGAAYNILGRSVLPHQLALGTFGAVIFFLLPNPFASKPQVQSVPIKAGSKDEEDFIKKYLQEHSTTEKK

>OEJ80702.1 hypothetical protein AWRI3579_g4070 [Hanseniaspora osmophila]
MGAYYKVLGAKIPSHFLAIGTYSAVIGGVLSMNKSKPADTANAPAAAAATVPVVPETSAKDEEFDLEKVLGSFLKEETK

Saccharomycodes ludwigii

>XP_045935018.1 hypothetical protein SCDLUD_002560 [Saccharomycodes ludwigii]
MAGGYTILGKHIPSHQLALTTFGLVSLLVIPNPFKSTKLPEPSINASNKEEEAFVKSYIKSKNEAIQKK

>XP_045933384.1 hypothetical protein SCDLUD_004894 [Saccharomycodes ludwigii]
MVASYTILGKSIPSHFLALGTFGAVIGGTLFAKSGSSAPAPVPTKKEQPATTAKSSNNEEFDVEKLIDSFLKEEDKK

Trichomonascaceae (Family)

Wickerhamiella sorbophila

>XP_024663554.1 hypothetical protein B9G98_01228 [Wickerhamiella sorbophila]
MGSAYKIFGRQVPAYQLSMATFGLIGAIVVAGTSGKKPAEAKPPIAAESSDEEKFIMDYLKKAEAESK

Trigonopsidaceae (Family)

Tortispora caseinolytica

>ODV91059.1 hypothetical protein CANCADRAFT_57423 [Tortispora caseinolytica NRRL Y-17796]
MAGGAYHIFGRNVYPHQLSIITLATITGAIVLSTSGKKSTASPNPPLNAASSDEEKFIMEYLAKAEKKE

Saccharomycetales incertae sedis

Diutina rugosa

>XP_034010333.1 uncharacterized protein DIURU_004930 [Diutina rugosa]
MGAAYHIMGRSVPAHQLAIATLGLVTLLAIPKPWNPAPKHPKIIAESEDEKKFIENYVQDHLKAEKH

Pachysolen tannophilus NRRL Y-2460

>ODV97039.1 hypothetical protein PACTADRAFT_1622 [Pachysolen tannophilus NRRL Y-2460]
MSGPAYQILGRSVQPHQLAIATLSTVLFFVIPKPWGSKTLASPSIGASSPEEEKFIKEYLAKKEKSE

Pezizomycotina (Subphylum)

Trichodelitschia bisporula

>KAF2403342.1 hypothetical protein EJ06DRAFT_553687 [Trichodelitschia bisporula]
MVAYYKIFGQQVGSHILAIATLSSVFAVSAISMRSPASAKSQGPPIQASSKDEESFIKEFVTKAEADKKH

Bipolaris oryzae ATCC 44560

>XP_007690537.1 hypothetical protein COCMIDRAFT_102152 [Bipolaris oryzae ATCC 44560]
MVAMYTIFGRQVGSHVLAIATLATTFTGAALSMGGKKAEDPTTPPINAKSSEEENFVKEYVKKAADKVSGK

Dothidotthia symphoricarpi CBS 119687

>XP_033519616.1 uncharacterized protein P153DRAFT_370532 [Dothidotthia symphoricarpi CBS 119687]
MVAMYTVFGRQVGSHVLAIATLSVTFAGAALSMGGSKAEASTQPPINAKTKEEESFVKDYVKKAAEKVQGKA

Bipolaris sorokiniana ND90Pr

>XP_007699259.1 uncharacterized protein COCSADRAFT_87990 [Bipolaris sorokiniana ND90Pr]
MVAMYTIFGRQVGSHVLAIATLATTFTGAALSMGGKKAEDPTTPPINAKSSEEENFVKEYVKKAADKVQGKQ

Letharia columbiana

>XP_037163959.1 uncharacterized protein HO173_007194 [Letharia columbiana]
MVAQYTIFGRQVGSHVLAMLTLGTFAAGTAFAMSGKEKAKEQGPPINASSKDEEEFIQEFLKNNGGEQKAKQ

Viridothelium virens

>KAF2236919.1 hypothetical protein EV356DRAFT_530436 [Viridothelium virens]
MVAMYTVFGRQVGSHVLAMGVLGTLFAGGFYAGSGSKKPSEQSPPINATSKDEENFIQEFLKNAEAEDNKQKH

Decorospora gaudefroyi

>KAF1830242.1 hypothetical protein BDW02DRAFT_573235 [Decorospora gaudefroyi]
MVAMYTIMGRQVGSHVLAIATLGTTFAGAALAMGGKKGEEKASGPPINAKSSEEENFVKEYVRKAKDTVQGKQ

Ascobolus immersus RN42

>RPA87580.1 hypothetical protein BJ508DRAFT_410340 [Ascobolus immersus RN42]
MVVYYPVFGKQVGSHVLAMLTLGTAATIGYVSTRGGSKEGKPTPPIVASSSDEEKFIKDFLKDIEADEKKTKAKH

Alectoria fallacina

>CAF9940478.1 hypothetical protein ALECFALPRED_008692 [Alectoria fallacina]
MVAQYTIFGRQVGSHVLAMLTLGTFAAGTTLAMGGKEKAKGQGPPINASSKDEEEFIQFVNFLEFLKNNGEEQKAKQ

Pleomassaria siparia CBS 279.74

>KAF2705562.1 hypothetical protein K504DRAFT_505968 [Pleomassaria siparia CBS 279.74]
MVAMYSIFGKQVGSHVLAIATLSTVGAVAYLSVGGSKPAESAGPAINAKSKEEESFIKYDSRVPATATTLHIRYRTKDFVKKAAGEKK

Bipolaris maydis

>KAH7549068.1 hypothetical protein BM1_10453 [Bipolaris maydis]
MVAMYTIFGRQVGSHVLAIATLATTFTGAALSMGGKKAEDPTTPPINAKSSEEENFVKCVSPYISLYPPTWSLAVSRETNQSCLYREYVKKAADKVQGKQ

Taphrinomycotina (Subphylum)

Schizosaccharomyces pombe

>NP_001343074.1 putative F0-ATPase subunit K [Schizosaccharomyces pombe]
MSVYTIAGRQFQAHQLSLAVLGSVFVGPVIYSKLFKRNKPLSAKDVPPLNAKSKEEEEFILKYIEEHK

Schizosaccharomyces cryophilus OY26

>XP_013022271.1 F0-ATPase subunit K [Schizosaccharomyces cryophilus OY26]
MSVYTIAGRQFQSHQLSLAVLGSVFVGPWVYSKLFKRQKALKEGEMPPLNASNKEEEAFILKYIKDHK

Schizosaccharomyces octosporus yFS286

>XP_013018657.1 F0-ATPase subunit K [Schizosaccharomyces octosporus yFS286]
MSVYTIAGRQFQSYQLSLTVLGSVFVGPWVYSKLFKRQKSLKEGEMPPLNASNKEEEAFILKYIKDHK
