# Supplementary material for: ATP synthase interactome analysis identifies a new subunit l as a modulator of permeability transition pore in yeast
Source: Sci Rep. 2023 Mar 7;13:3839. doi: 10.1038/s41598-023-30966-5 (PMC9992712; doi:10.1038/s41598-023-30966-5)
Supplement: Supplementary file 6 — Supplementary Information 6. [file 41598_2023_30966_MOESM6_ESM.pdf]

BAND 1

prot_hit_num	prot_acc	prot_desc	prot_mass	prot_matches	prot_cover	emPAI
11	Q0080	Subunit 8 of the F0 sector of mitochondrial F1F0 ATP synthase; encoded on the mitochondrial genome; ATP8 and ATP6 mRNAs are not translated in the absence of the F1 sector of ATPase	5818	2	12,5	2,96
3	YML081C-A	Subunit of the mitochondrial F1F0 ATP synthase; F1F0 ATP synthase is a large, evolutionarily conserved enzyme complex required for ATP synthesis; termed subunit I or subunit j; does not correspond to known ATP synthase subunits in other organisms	6683	12	59,3	122,71
4	YMR256C	Subunit VII of cytochrome c oxidase (Complex IV); Complex IV is the terminal member of the mitochondrial inner membrane electron transport chain	6928	4	15	2,22
1	YDL067C	Subunit VIIa of cytochrome c oxidase (Complex IV); Complex IV is the terminal member of the mitochondrial inner membrane electron transport chain	6959	44	37,3	313,75
5	YGR183C	Subunit 9 of ubiquinol cytochrome-c reductase (Complex III); Complex III is a component of the mitochondrial inner membrane electron transport chain; required for electron transfer at the ubiquinol oxidase site of the complex	7472	4	36,4	4,09
6	YDR119W-A	Putative protein of unknown function; copurifies with respiratory chain supercomplexes composed of Complex III (ubiquinol-cytochrome c reductase) and Complex IV (cytochrome c oxidase)	7504	1	16,7	0,71
2	YOL077W-A	Subunit k of the mitochondrial F1F0 ATP synthase; F1F0 ATP synthase is a large, evolutionarily conserved enzyme complex required for ATP synthesis; associated only with the dimeric form of ATP synthase	7529	12	50	23,8
15	YIR021W-A	Putative protein of unknown function; identified by expression profiling and mass spectrometry	7732	1	10	0,68
13	Q0130	F0-ATP synthase subunit c (ATPase-associated proteolipid); encoded on the mitochondrial genome; mutation confers oligomycin resistance; expression is specifically dependent on the nuclear genes AEP1 and AEP2	7811	1	10,5	0,68
8	YLR395C	Subunit VIII of cytochrome c oxidase (Complex IV); Complex IV is the terminal member of the mitochondrial inner membrane electron transport chain	9015	1	12,8	0,57
16	YHR021C	Protein component of the small (40S) ribosomal subunit; homologous to mammalian ribosomal protein S27, no bacterial homolog; RPS27B has a paralog, RPS27A, that arose from the whole genome duplication	9145	1	8,5	0,56
27	YDL064W	SUMO-conjugating enzyme involved in the Smt3p conjugation pathway; nuclear protein required for S- and M-phase cyclin degradation and mitotic control; involved in proteolysis mediated by the anaphase-promoting complex cyclosome (APCC)	18014	1	3,8	0,26
21	YHR121W	Protein of unknown function that may function in RNA processing; interacts with Pbp1p and Pbp4p and associates with ribosomes; contains an RNA-binding LSM domain and an AD domain; GFP-fusion protein is induced by the DNA-damaging agent MMS; relative	21357	1	5,3	0,22
28	YOR163W	Polyphosphate phosphatase; hydrolyzes diphosphorylated inositol polyphosphates and diadenosine polyphosphates; high specificity for diadenosine hexa- and pentaphosphates; contains endopolyphosphatase activity with a high affinity for polyphosphates,	21787	1	4,3	0,21
10	YDR435C	Carboxyl methyltransferase; methylates the C terminus of the protein phosphatase 2A catalytic subunit (Pph21p or Pph22p), which is important for complex formation with regulatory subunits; required for methionine to inhibit autophagy and promote gro	38012	1	2,1	0,12
7	YFL039C	Actin; structural protein involved in cell polarization, endocytosis, and other cytoskeletal functions	41891	1	2,7	0,11
20	YPL224C	Putative metal transporter involved in mitochondrial iron accumulation; MMT2 has a paralog, MMT1, that arose from the whole genome duplication	52516	1	1,2	0,08
12	YHR005C	Subunit of the G protein involved in pheromone response; GTP-binding alpha subunit of the heterotrimeric G protein; negatively regulates the mating pathway by sequestering G(beta)gamma and by triggering an adaptive response; activates Vps34p at the	54498	1	1,5	0,08
25	YML120C	NADH:ubiquinone oxidoreductase; transfers electrons from NADH to ubiquinone in the respiratory chain but does not pump protons, in contrast to the higher eukaryotic multisubunit respiratory complex I; phosphorylated; involved in Mn and H2O2 induced	57214	1	2,9	0,08
26	YLR343W	1,3-beta-glucanosyltransferase; involved with Gas4p in spore wall assembly; has similarity to Gas1p	63235	1	1,1	0,07
19	YLL018C	Aspartyl-tRNA synthetase, primarily cytoplasmic; homodimeric enzyme that catalyzes the specific aspartylation of tRNA(Asp); class II aminoacyl tRNA synthetase; binding to its own mRNA may confer autoregulation; shares five highly conserved amino aci	63647	1	1,3	0,07
22	YMR291W	Protein kinase of unknown cellular role; green fluorescent protein (GFP)-fusion protein localizes to the cytoplasm and nucleus; null mutant is sensitive to expression of the top1-T722A allele; not an essential gene; relocalizes from nucleus to cytop	66976	1	1,2	0,07
9	YMR106C	Subunit of the telomeric Ku complex (Yku70p-Yku80p); involved in telomere length maintenance, structure and telomere position effect; required for localization of telomerase ribonucleoprotein via interaction with the TLC1 guide RNA; relocates to sit	71824	1	1	0,06
29	YDR380W	Phenylpyruvate decarboxylase; catalyzes decarboxylation of phenylpyruvate to phenylacetaldehyde, which is the first specific step in the Ehrlich pathway; involved in protein N-terminal Met and Ala catabolism	72023	1	1,1	0,06

14	YER005W	Apyrase with wide substrate specificity; helps prevent inhibition of glycosylation by hydrolyzing nucleoside tri- and diphosphates that inhibit glycotransferases; partially redundant with Gda1p; mediates adenovirus E4orf4-induced toxicity	72434	1	1,3	0,06
23	YPL020C	Protease that specifically cleaves Smt3p protein conjugates; required for cell cycle progression; associates with nucleoporins and may interact with septin rings during telophase; sequestered to the nucleolus under stress conditions	72618	2	1,1	0,06
18	YKL179C	Golgi membrane protein with similarity to mammalian CASP; genetic interactions with GOS1 (encoding a Golgi snare protein) suggest a role in Golgi function	77524	2	1,2	0,12
24	YLL013C	Protein of the mitochondrial outer surface; links the Arp2/3 complex with the mitochore during anterograde mitochondrial movement; also binds to and promotes degradation of mRNAs for select nuclear-encoded mitochondrial proteins	98178	1	0,8	0,04
17	YBR245C	ATPase subunit of imitation-switch (ISWI) class chromatin remodelers; with Ioc3p forms Isw1a complex involved in repression of transcription initiation; with Ioc2p and Ioc4p forms Isw1b complex involved in regulation of transcription elongation; Isw	131534	1	0,5	0,03

BAND 2

prot_hit_num	prot_acc	prot_desc	prot_mass	prot_matches	prot_cover	emPAI
1	YPL271W	Epsilon subunit of the F1 sector of mitochondrial F1F0 ATP synthase; which is a large, evolutionarily conserved enzyme complex required for ATP synthesis; F1 translationally regulates ATP6 and ATP8 expression to achieve a balanced output of ATP synt	6738	15	62,9	34,08
8	YDL067C	Subunit VIIa of cytochrome c oxidase (Complex IV); Complex IV is the terminal member of the mitochondrial inner membrane electron transport chain	6959	1	13,6	0,78
4	YPR010C-A	Putative protein of unknown function; conserved among Saccharomyces sensu stricto species	7954	2	30,6	1,76
5	YER019C-A	Ssh1p-Sss1p-Sbh2p complex component; involved in protein translocation into the endoplasmic reticulum; SBH2 has a paralog, SBH1, that arose from the whole genome duplication	9600	3	22,7	1,35
3	YDL181W	Protein that inhibits ATP hydrolysis by the F1F0-ATP synthase; inhibitory function is enhanced by stabilizing proteins Stf1p and Stf2p; has a calmodulin-binding motif and binds calmodulin in vitro; INH1 has a paralog, STF1, that arose from the whole	9864	2	21,2	1,29
6	YDR322C-A	Subunit e of mitochondrial F1F0-ATPase; ATPase is a large, evolutionarily conserved enzyme complex required for ATP synthesis; essential for the dimeric and oligomeric state of ATP synthase, which in turn determines the shape of inner membrane crist	10926	1	10,4	0,46
2	YDR377W	Subunit f of the F0 sector of mitochondrial F1F0 ATP synthase; F1F0 ATP synthase is a large, evolutionarily conserved enzyme complex required for ATP synthesis	11305	4	19,8	1,07
20	YER139C	CTD phosphatase; dephosphorylates S5-P in the C-terminal domain of Rpo21p; has a cysteine-rich motif required for function and conserved in eukaryotes; shuttles between the nucleus and cytoplasm; RTR1 has a paralog, RTR2, that arose from the whole g	26565	1	2,7	0,17
27	YPL078C	Subunit b of the stator stalk of mitochondrial F1F0 ATP synthase; ATP synthase is a large, evolutionarily conserved enzyme complex required for ATP synthesis; contributes to the oligomerization of the complex, which in turn determines the shape of i	26965	1	3,7	0,17
9	YBR039W	Gamma subunit of the F1 sector of mitochondrial F1F0 ATP synthase; F1F0 ATP synthase is a large, evolutionarily conserved enzyme complex required for ATP synthesis	34443	1	3,5	0,13
26	YKL107W	Putative short-chain dehydrogenase/reductase; proposed to be a palmitoylated membrane protein	34674	1	2,9	0,13
13	YDR435C	Carboxyl methyltransferase; methylates the C terminus of the protein phosphatase 2A catalytic subunit (Pph21p or Pph22p), which is important for complex formation with regulatory subunits; required for methionine to inhibit autophagy and promote gro	38012	1	2,1	0,12
28	YKR059W	Translation initiation factor eIF4A; DEA(D/H)-box RNA helicase that couples ATPase activity to RNA binding and unwinding; forms a dumbbell structure of two compact domains connected by a linker; interacts with eIF4G; protein abundance increases in r	44840	1	2,3	0,1
19	YCL029C	Microtubule-associated protein; component of the interface between microtubules and kinetochore, involved in sister chromatid separation; essential in polyploid cells but not in haploid or diploid cells; ortholog of mammalian CLIP-170	51288	1	1,4	0,09
23	YPL224C	Putative metal transporter involved in mitochondrial iron accumulation; MMT2 has a paralog, MMT1, that arose from the whole genome duplication	52516	1	1,2	0,08
22	YDR388W	Actin-associated protein with roles in endocytosis and exocytosis; interacts with Rvs161p to regulate actin cytoskeleton, endocytosis, and viability following starvation or osmotic stress; recruited to bud tips by Gyl1p and Gyp5p during polarized gr	52799	1	1,5	0,08
16	YHR005C	Subunit of the G protein involved in pheromone response; GTP-binding alpha subunit of the heterotrimeric G protein; negatively regulates the mating pathway by sequestering G(beta)gamma and by triggering an adaptive response; activates Vps34p at the	54498	1	1,5	0,08
15	YPR169W	Protein required for biogenesis of the large ribosomal subunit; required for biogenesis of the large ribosomal subunit; interacts with proteins involved in RNA processing, ribosome biogenesis, ubiquitination and demethylation; similar to WDR55, a hu	55857	1	1,4	0,08
7	YBL099W	Alpha subunit of the F1 sector of mitochondrial F1F0 ATP synthase; which is a large, evolutionarily conserved enzyme complex required for ATP synthesis; F1 translationally regulates ATP6 and ATP8 expression to achieve a balanced output of ATP syntha	58629	5	5,7	0,33

10	YMR106C	Subunit of the telomeric Ku complex (Yku70p-Yku80p); involved in telomere length maintenance, structure and telomere position effect; required for localization of telomerase ribonucleoprotein via interaction with the TLC1 guide RNA; relocates to sit	71824	1	1	0,06
17	YER005W	Apyrase with wide substrate specificity; helps prevent inhibition of glycosylation by hydrolyzing nucleoside tri- and diphosphates that inhibit glycotransferases; partially redundant with Gda1p; mediates adenovirus E4orf4-induced toxicity	72434	2	1,3	0,06
25	YPL020C	Protease that specifically cleaves Smt3p protein conjugates; required for cell cycle progression; associates with nucleoporins and may interact with septin rings during telophase; sequestered to the nucleolus under stress conditions	72618	1	1,1	0,06
21	YNR019W	Acyl-CoA:sterol acyltransferase; endoplasmic reticulum enzyme that contributes the major sterol esterification activity in the presence of oxygen; ARE2 has a paralog, ARE1, that arose from the whole genome duplication	74431	1	1,1	0,06
24	YKL179C	Golgi membrane protein with similarity to mammalian CASP; genetic interactions with GOS1 (encoding a Golgi snare protein) suggest a role in Golgi function	77524	2	1,2	0,12
12	YHR031C	DNA helicase involved in rDNA replication and Ty1 transposition; binds to and suppresses DNA damage at G4 motifs in vivo; relieves replication fork pauses at telomeric regions; structurally and functionally related to Pif1p	81758	3	1,4	0,11
14	YMR089C	Mitochondrial inner membrane m-AAA protease component; mediates degradation of misfolded or unassembled proteins; also required for correct assembly of mitochondrial enzyme complexes	93503	1	0,7	0,05
11	YPR029C	Gamma-adaptin; large subunit of the clathrin-associated protein (AP-1) complex; binds clathrin; involved in vesicle mediated transport	94079	1	0,8	0,05
18	YFR016C	Putative protein of unknown function; green fluorescent protein (GFP)-fusion protein localizes to the cytoplasm and bud; interacts with Spa2p; YFR016C is not an essential gene	137844	1	0,6	0,03

BAND 3

prot_hit_num	prot_acc	prot_desc	prot_mass	prot_matches	prot_cover	emPAI
23	YFR032C-A	Ribosomal 60S subunit protein L29; not essential for translation, but required for proper joining of large and small ribosomal subunits and for normal translation rate; homologous to mammalian ribosomal protein L29, no bacterial homolog	6665	1	13,6	0,83
5	YPL271W	Epsilon subunit of the F1 sector of mitochondrial F1F0 ATP synthase; which is a large, evolutionarily conserved enzyme complex required for ATP synthesis; F1 translationally regulates ATP6 and ATP8 expression to achieve a balanced output of ATP synt	6738	1	17,7	0,81
11	YDL067C	Subunit VIIa of cytochrome c oxidase (Complex IV); Complex IV is the terminal member of the mitochondrial inner membrane electron transport chain	6959	1	13,6	0,78
18	YBR162W-A	Protein of unknown function; expression suppresses a secretory pathway mutation in E. coli; has similarity to the mammalian RAMP4 protein involved in secretion	7360	1	10,8	0,73
17	YKL023C-A	Putative protein of unknown function	8514	1	9,3	0,61
2	YHR001W-A	Subunit of the ubiquinol-cytochrome c oxidoreductase complex; this complex comprises part of the mitochondrial respiratory chain; members include Cobp, Rip1p, Cyt1p, Cor1p, Qcr2p, Qcr6p, Qcr7p, Qcr8p, Qcr9p, and Qcr10p and comprises part of the mitoc	8587	9	55,8	9,78
7	YDR086C	Subunit of the Sec61p translocation complex (Sec61p-Sss1p-Sbh1p); this complex forms a channel for passage of secretory proteins through the endoplasmic reticulum membrane, and of the Ssh1p complex (Ssh1p-Sbh2p-Sss1p); interacts with Ost4p and Wbp1p	8995	1	12,5	0,57
8	YJL062W-A	Mitochondrial protein required for cytochrome c oxidase assembly; also involved in translational regulation of Cox1p and prevention of Cox1p aggregation before assembly; located in the mitochondrial inner membrane	9875	2	12,9	0,51
1	YDR322C-A	Subunit e of mitochondrial F1F0-ATPase; ATPase is a large, evolutionarily conserved enzyme complex required for ATP synthesis; essential for the dimeric and oligomeric state of ATP synthase, which in turn determines the shape of inner membrane crist	10926	48	81,2	611,37
3	YDR377W	Subunit f of the F0 sector of mitochondrial F1F0 ATP synthase; F1F0 ATP synthase is a large, evolutionarily conserved enzyme complex required for ATP synthesis	11305	4	28,7	1,97
10	YOR020C	Mitochondrial matrix co-chaperonin; inhibits the ATPase activity of Hsp60p, a mitochondrial chaperonin; involved in protein folding and sorting in the mitochondria; 10 kD heat shock protein with similarity to E. coli groES	11365	1	10,4	0,44
4	YMR225C	Mitochondrial ribosomal protein of the large subunit; protein abundance increases in response to DNA replication stress	11469	2	11,2	0,43
14	YBL003C	Histone H2A; core histone protein required for chromatin assembly and chromosome function; one of two nearly identical (see also HTA1) subtypes; DNA damage-dependent phosphorylation by Mec1p facilitates DNA repair; acetylated by Nat4p	13981	1	6,8	0,34
36	YOR163W	Polyphosphate phosphatase; hydrolyzes diphosphorylated inositol polyphosphates and diadenosine polyphosphates; high specificity for diadenosine hexa- and pentaphosphates; contains endopolyphosphatase activity with a high affinity for polyphosphates,	21787	1	4,3	0,21
32	YBL030C	Major ADP/ATP carrier of the mitochondrial inner membrane; exchanges cytosolic ADP for mitochondrially synthesized ATP; also imports heme and ATP; phosphorylated; required for viability in many lab strains that carry a sal1 mutation; PET9 has a para	34632	1	2,8	0,13
35	YLR100W	3-keto sterol reductase; catalyzes the last of three steps required to remove two C-4 methyl groups from an intermediate in ergosterol biosynthesis; mutants are sterol auxotrophs	39757	1	2,3	0,11
6	YFL039C	Actin; structural protein involved in cell polarization, endocytosis, and other cytoskeletal functions	41891	1	2,7	0,11

22	YCL029C	Microtubule-associated protein; component of the interface between microtubules and kinetochore, involved in sister chromatid separation; essential in polyploid cells but not in haploid or diploid cells; ortholog of mammalian CLIP-170	51288	1	1,4	0,09
24	YPL224C	Putative metal transporter involved in mitochondrial iron accumulation; MMT2 has a paralog, MMT1, that arose from the whole genome duplication	52516	1	1,2	0,08
28	YHR005C	Subunit of the G protein involved in pheromone response; GTP-binding alpha subunit of the heterotrimeric G protein; negatively regulates the mating pathway by sequestering G(beta)gamma and by triggering an adaptive response; activates Vps34p at the	54498	1	1,5	0,08
16	YPR169W	Protein required for biogenesis of the large ribosomal subunit; required for biogenesis of the large ribosomal subunit; interacts with proteins involved in RNA processing, ribosome biogenesis, ubiquitination and demethylation; similar to WDR55, a hu	55857	1	1,4	0,08
37	YML056C	Inosine monophosphate dehydrogenase; catalyzes the rate-limiting step in the de novo synthesis of GTP; member of a four-gene family in S. cerevisiae, constitutively expressed; IMD4 has a paralog, IMD3, that arose from the whole genome duplication	56871	1	1,3	0,08
27	YER051W	JmjC domain family histone demethylase specific for H3-K36; similar to proteins found in human, mouse, drosophila, X. laevis, C. elegans, and S. pombe	57008	1	1,2	0,08
34	YDL146W	Protein involved in the regulation of endocytosis; transiently recruited to actin cortical patches in a SLA1-dependent manner after late coat component assembly; GFP-fusion protein localizes to the periphery, cytoplasm, bud, and bud neck	57026	1	1,4	0,08
12	YBL099W	Alpha subunit of the F1 sector of mitochondrial F1F0 ATP synthase; which is a large, evolutionarily conserved enzyme complex required for ATP synthesis; F1 translationally regulates ATP6 and ATP8 expression to achieve a balanced output of ATP syntha	58629	1	2,4	0,07
26	YMR291W	Protein kinase of unknown cellular role; green fluorescent protein (GFP)-fusion protein localizes to the cytoplasm and nucleus; null mutant is sensitive to expression of the top1-T722A allele; not an essential gene; relocalizes from nucleus to cytop	66976	1	1,2	0,07
38	YGL228W	Protein involved in outer spore wall assembly; likely involved directly in dityrosine layer assembly; putative GPI-anchored protein; overexpression causes growth arrest; SHE10 has a paralog, OSW7/YFR039C, that arose from the whole genome duplication	67334	1	1	0,06
9	YMR106C	Subunit of the telomeric Ku complex (Yku70p-Yku80p); involved in telomere length maintenance, structure and telomere position effect; required for localization of telomerase ribonucleoprotein via interaction with the TLC1 guide RNA; relocates to sit	71824	1	1	0,06
13	YER005W	Apyrase with wide substrate specificity; helps prevent inhibition of glycosylation by hydrolyzing nucleoside tri- and diphosphates that inhibit glycotransferases; partially redundant with Gda1p; mediates adenovirus E4orf4-induced toxicity	72434	1	1,3	0,06
19	YPL020C	Protease that specifically cleaves Smt3p protein conjugates; required for cell cycle progression; associates with nucleoporins and may interact with septin rings during telophase; sequestered to the nucleolus under stress conditions	72618	2	1,1	0,06
30	YGL026C	Tryptophan synthase; catalyzes the last step of tryptophan biosynthesis; regulated by the general control system of amino acid biosynthesis	76977	1	1	0,06
20	YKL179C	Golgi membrane protein with similarity to mammalian CASP; genetic interactions with GOS1 (encoding a Golgi snare protein) suggest a role in Golgi function	77524	1	1	0,06
21	YDR422C	Alternate beta-subunit of the Snf1p kinase complex; may confer substrate specificity; vacuolar protein containing KIS (Kinase-Interacting Sequence) and ASC (Association with Snf1 kinase Complex) domains involved in protein interactions	91243	1	1,5	0,05
15	YMR089C	Mitochondrial inner membrane m-AAA protease component; mediates degradation of misfolded or unassembled proteins; also required for correct assembly of mitochondrial enzyme complexes	93503	1	0,7	0,05
25	YFR016C	Putative protein of unknown function; green fluorescent protein (GFP)-fusion protein localizes to the cytoplasm and bud; interacts with Spa2p; YFR016C is not an essential gene	137844	1	0,6	0,03
33	YJR137C	Sulfite reductase beta subunit; involved in amino acid biosynthesis, transcription repressed by methionine	161519	2	0,8	0,03
31	YLR024C	Cytoplasmic ubiquitin-protein ligase (E3); required for ubiquitylation of Rpn4p; mediates formation of a Mub1p-Ubr2p-Rad6p complex	219490	1	0,4	0,02
29	YJL207C	AP-1 accessory protein; colocalizes with clathrin to the late-Golgi apparatus; involved in TGN-endosome transport; physically interacts with AP-1; similar to the mammalian p200; may interact with ribosomes; YJL207C is a non-essential gene	231123	1	0,3	0,02

BAND 4

prot_hit_num	prot_acc	prot_desc	prot_mass	prot_matches	prot_cover	emPAI
4	YML129C	Mitochondrial cytochrome c oxidase (complex IV) assembly factor; also involved in translational regulation of Cox1p and prevention of Cox1p aggregation before assembly; associates with complex IV assembly intermediates and complex III/complex IV sup	7954	5	50	11,61
8	YCL005W-A	Vacuolar H+ ATPase subunit e of the V-ATPase V0 subcomplex; essential for vacuolar acidification; interacts with the V-ATPase assembly factor Vma21p in the ER; involved in V0 biogenesis	8432	2	13,7	1,62
10	YER087C-B	Beta subunit of Sec61p ER translocation complex (Sec61p-Sss1p-Sbh1p); involved in protein translocation into the endoplasmic reticulum; interacts with the exocyst complex and also with Rtn1p; cotranslationally N-acetylated by NatA; SBH1 has a paralo	8706	1	12,2	0,6
11	YDR086C	Subunit of the Sec61p translocation complex (Sec61p-Sss1p-Sbh1p); this complex forms a channel for passage of secretory proteins through the endoplasmic reticulum membrane, and of the Ssh1p complex (Ssh1p-Sbh2p-Sss1p); interacts with Ost4p and Wbp1p	8995	1	12,5	0,57
2	YOR020W-A	Putative protein of unknown function; conserved in A. gossypii; the authentic, non-tagged protein is detected in highly purified mitochondria in high-throughput studies	9612	11	56,7	18,33

9	YDR322C-A	Subunit e of mitochondrial F1F0-ATPase; ATPase is a large, evolutionarily conserved enzyme complex required for ATP synthesis; essential for the dimeric and oligomeric state of ATP synthase, which in turn determines the shape of inner membrane crist	10926	1	10,4	0,46
14	YJL166W	Subunit 8 of ubiquinol cytochrome-c reductase (Complex III); Complex III is a component of the mitochondrial inner membrane electron transport chain; oriented facing the intermembrane space; expression is regulated by Abf1p and Cpf1p	10967	1	12,8	0,45
13	YER048W-A	Cysteine desulfurase (Nfs1p) activator; essential for the formation of the persulfide intermediate at the desulfurase active site during pyridoxal phosphate-dependent desulfuration of cysteine; required for mitochondrial iron-sulfur cluster biosynth	11259	2	16	1,08
1	YDR377W	Subunit f of the F0 sector of mitochondrial F1F0 ATP synthase; F1F0 ATP synthase is a large, evolutionarily conserved enzyme complex required for ATP synthesis	11305	21	38,6	53,28
5	YJR048W	Cytochrome c, isoform 1; also known as iso-1-cytochrome c; electron carrier of the mitochondrial intermembrane space that transfers electrons from ubiquinone-cytochrome c oxidoreductase to cytochrome c oxidase during cellular respiration; mutations	12345	4	23,9	2,81
12	YBR262C	Component of the MICOS complex; MICOS (formerly MINOS or MitOS) is a mitochondrial inner membrane complex that extends into the intermembrane space and has a role in the maintenance of crista junctions, inner membrane architecture, and formation of	12380	2	18,9	0,94
3	YPR020W	Subunit g of the mitochondrial F1F0 ATP synthase; reversibly phosphorylated on two residues; unphosphorylated form is required for dimerization of the ATP synthase complex, which in turn determines oligomerization of the complex and the shape of inn	12970	8	44,3	5,74
7	YDR079W	Chaperone that facilitates the assembly of cytochrome c oxidase; integral to the mitochondrial inner membrane; interacts with a subcomplex of subunits VII, VIIa, and VIII (Cox7p, Cox9p, and Cox8p) but not with the holoenzyme	13236	3	18	1,56
17	YBR230C	Mitochondrial outer membrane receptor for cytosolic ribosomes; integral protein of the outer membrane that interacts with the nascent chain-associated complex (NAC) bound to ribosomes, contributing to co-translational mitochondrial import; interacts	14885	1	6	0,32
30	YCR003W	Mitochondrial ribosomal protein of the large subunit; protein abundance increases in response to DNA replication stress	21652	1	3,3	0,21
34	YOR163W	Polyphosphate phosphatase; hydrolyzes diphosphorylated inositol polyphosphates and diadenosine polyphosphates; high specificity for diadenosine hexa- and pentaphosphates; contains endopolyphosphatase activity with a high affinity for polyphosphates,	21787	1	4,3	0,21
21	YER139C	CTD phosphatase; dephosphorylates S5-P in the C-terminal domain of Rpo21p; has a cysteine-rich motif required for function and conserved in eukaryotes; shuttles between the nucleus and cytoplasm; RTR1 has a paralog, RTR2, that arose from the whole g	26565	1	2,7	0,17
32	YBR088C	Proliferating cell nuclear antigen (PCNA); functions as the sliding clamp for DNA polymerase delta; may function as a docking site for other proteins required for mitotic and meiotic chromosomal DNA replication and for DNA repair	29126	1	2,7	0,16
16	YDR435C	Carboxyl methyltransferase; methylates the C terminus of the protein phosphatase 2A catalytic subunit (Pph21p or Pph22p), which is important for complex formation with regulatory subunits; required for methionine to inhibit autophagy and promote gro	38012	1	2,1	0,12
22	YCL029C	Microtubule-associated protein; component of the interface between microtubules and kinetochore, involved in sister chromatid separation; essential in polyploid cells but not in haploid or diploid cells; ortholog of mammalian CLIP-170	51288	1	1,4	0,09
20	YPL224C	Putative metal transporter involved in mitochondrial iron accumulation; MMT2 has a paralog, MMT1, that arose from the whole genome duplication	52516	1	1,2	0,08
6	YJR121W	Beta subunit of the F1 sector of mitochondrial F1F0 ATP synthase; which is a large, evolutionarily conserved enzyme complex required for ATP synthesis; F1 translationally regulates ATP6 and ATP8 expression to achieve a balanced output of ATP synthas	54817	2	5,1	0,17
28	YKL012W	U1 snRNP protein involved in splicing; interacts with the branchpoint-binding protein during the formation of the second commitment complex	69080	1	1	0,06
15	YMR106C	Subunit of the telomeric Ku complex (Yku70p-Yku80p); involved in telomere length maintenance, structure and telomere position effect; required for localization of telomerase ribonucleoprotein via interaction with the TLC1 guide RNA; relocates to sit	71824	2	1	0,06
19	YER005W	Apyrase with wide substrate specificity; helps prevent inhibition of glycosylation by hydrolyzing nucleoside tri- and diphosphates that inhibit glycotransferases; partially redundant with Gda1p; mediates adenovirus E4orf4-induced toxicity	72434	1	1,3	0,06
23	YPL020C	Protease that specifically cleaves Smt3p protein conjugates; required for cell cycle progression; associates with nucleoporins and may interact with septin rings during telophase; sequestered to the nucleolus under stress conditions	72618	1	1,1	0,06
24	YNR019W	Acyl-CoA:sterol acyltransferase; endoplasmic reticulum enzyme that contributes the major sterol esterification activity in the presence of oxygen; ARE2 has a paralog, ARE1, that arose from the whole genome duplication	74431	1	1,1	0,06
27	YGL026C	Tryptophan synthase; catalyzes the last step of tryptophan biosynthesis; regulated by the general control system of amino acid biosynthesis	76977	1	1	0,06
29	YKL179C	Golgi membrane protein with similarity to mammalian CASP; genetic interactions with GOS1 (encoding a Golgi snare protein) suggest a role in Golgi function	77524	1	1,2	0,06
31	YMR186W	Cytoplasmic chaperone of the Hsp90 family; plays a role in determining prion variants; redundant in function and nearly identical with Hsp82p, and together they are essential; expressed constitutively at 10-fold higher basal levels than HSP82 and in	80850	1	1	0,05
18	YMR089C	Mitochondrial inner membrane m-AAA protease component; mediates degradation of misfolded or unassembled proteins; also required for correct assembly of mitochondrial enzyme complexes	93503	1	0,7	0,05
26	YJL165C	Putative protein kinase; overexpression increases sodium and lithium tolerance, whereas gene disruption increases cation and low pH sensitivity and impairs potassium uptake, suggesting a role in regulation of Trk1p and/or Trk2p transporters; HAL5 ha	96251	1	0,8	0,05
33	YLR148W	Component of CORVET membrane tethering complex; vacuolar peripheral membrane protein that promotes vesicular docking/fusion reactions in conjunction with SNARE proteins, required for vacuolar biogenesis	108014	1	2,3	0,04
25	YFR016C	Putative protein of unknown function; green fluorescent protein (GFP)-fusion protein localizes to the cytoplasm and bud; interacts with Spa2p; YFR016C is not an essential gene	137844	2	0,6	0,03

BAND 5

prot_hit_num	prot_acc	prot_desc	prot_mass	prot_matches	prot_cover	emPAI
9	YCL057C-A	Conserved component of the MICOS complex; MICOS (formerly MINOS or MitOS) is a mitochondrial inner membrane complex that extends into the intermembrane space and has a role in the maintenance of crista junctions, inner membrane architecture, and for	10401	1	10,3	0,48
4	YLR361C-A	Putative protein of unknown function	11101	3	12,2	0,45
35	YER058W	Protein required for assembly of cytochrome c oxidase	12330	1	8,4	0,4
10	YLR390W	Putative protein of unknown function; the authentic, non-tagged protein is detected in highly purified mitochondria in high-throughput studies	12622	1	8	0,39
25	YEL039C	Cytochrome c isoform 2, expressed under hypoxic conditions; also known as iso-2-cytochrome c; electron carrier of the mitochondrial intermembrane space that transfers electrons from ubiquinone-cytochrome c oxidoreductase to cytochrome c oxidase duri	12638	1	9,7	0,39
11	YDR381C-A	Protein of unknown function; localized to the mitochondrial outer membrane	12764	1	9,6	0,38
6	YBL059C-A	Protein involved in respiratory chain complex assembly or maintenance; protein of the mitochondrial intermembrane space; contains twin Cx9C motifs that can form coiled coil-helix-coiled-coil helix fold	13167	2	20,2	0,87
21	YDR079W	Chaperone that facilitates the assembly of cytochrome c oxidase; integral to the mitochondrial inner membrane; interacts with a subcomplex of subunits VII, VIIa, and VIII (Cox7p, Cox9p, and Cox8p) but not with the holoenzyme	13236	1	6,3	0,37
15	YBR230C	Mitochondrial outer membrane receptor for cytosolic ribosomes; integral protein of the outer membrane that interacts with the nascent chain-associated complex (NAC) bound to ribosomes, contributing to co-translational mitochondrial import; interacts	14885	1	6	0,32
1	YGL191W	Subunit VIa of cytochrome c oxidase; present in a subclass of cytochrome c oxidase complexes that may have a role in mimimizing generation of reactive oxygen species; not essential for cytochrome c oxidase activity but may modulate activity in respo	15069	18	46,5	14,61
3	YDR511W	Mitochondrial protein involved in assembly of succinate dehydrogenase; has a role in maturation of the Sdh2p subunit; localized to the mitochondrial intermembrane space; required for acetate utilization and gluconeogenesis; mutation in Drosophila or	15773	5	27,8	1,87
2	YDL004W	Delta subunit of the central stalk of mitochondrial F1F0 ATP synthase; F1F0 ATP synthase is a large, evolutionarily conserved enzyme complex required for ATP synthesis; F1 translationally regulates ATP6 and ATP8 expression to achieve a balanced outp	17067	10	10	1,65
7	YNL052W	Subunit Va of cytochrome c oxidase; cytochrome c oxidase is the terminal member of the mitochondrial inner membrane electron transport chain; Cox5Ap is predominantly expressed during aerobic growth while its isoform Vb (Cox5Bp) is expressed during a	17130	2	12,4	0,63
5	YHR051W	Subunit VI of cytochrome c oxidase (Complex IV); Complex IV is the terminal member of the mitochondrial inner membrane electron transport chain; expression is regulated by oxygen levels	17388	5	22,3	2,3
8	YPL135W	Conserved protein of the mitochondrial matrix; performs a scaffolding function during assembly of iron-sulfur clusters, interacts physically and functionally with yeast frataxin (Yfh1p); isu1 isu2 double mutant is inviable; ISU1 has a paralog, ISU2,	18055	2	12,1	0,58
37	YOR163W	Polyphosphate phosphatase; hydrolyzes diphosphorylated inositol polyphosphates and diadenosine polyphosphates; high specificity for diadenosine hexa- and pentaphosphates; contains endopolyphosphatase activity with a high affinity for polyphosphates,	21787	1	4,3	0,21
22	YER139C	CTD phosphatase; dephosphorylates S5-P in the C-terminal domain of Rpo21p; has a cysteine-rich motif required for function and conserved in eukaryotes; shuttles between the nucleus and cytoplasm; RTR1 has a paralog, RTR2, that arose from the whole g	26565	1	2,7	0,17
26	YBR128C	Autophagy-specific subunit of phosphatidylinositol 3-kinase complex I; Atg14p targets complex I to the phagophore assembly site (PAS); required for localizing additional ATG proteins to the PAS; required for overflow degradation of misfolded protein	40940	1	2	0,11
18	YCL029C	Microtubule-associated protein; component of the interface between microtubules and kinetochore, involved in sister chromatid separation; essential in polyploid cells but not in haploid or diploid cells; ortholog of mammalian CLIP-170	51288	2	1,4	0,09
17	YPL224C	Putative metal transporter involved in mitochondrial iron accumulation; MMT2 has a paralog, MMT1, that arose from the whole genome duplication	52516	2	1,2	0,08
36	YDR388W	Actin-associated protein with roles in endocytosis and exocytosis; interacts with Rvs161p to regulate actin cytoskeleton, endocytosis, and viability following starvation or osmotic stress; recruited to bud tips by Gyl1p and Gyp5p during polarized gr	52799	1	1,5	0,08
28	YPL179W	Protein phosphatase that regulates the mating response; negatively regulates the MAP kinase signaling cascade during mating; member of the serine/threonine phosphatase PP1 family	61838	1	1,8	0,07
33	YKL005C	Negative regulator of transcription elongation; contains a TFIIS-like domain that associates with chromatin and a PHD domain that interacts with H3K4me3; multicopy suppressor of temperature-sensitive ess1 mutations, binds RNA polymerase II large sub	68429	1	1,2	0,06

12	YMR106C	Subunit of the telomeric Ku complex (Yku70p-Yku80p); involved in telomere length maintenance, structure and telomere position effect; required for localization of telomerase ribonucleoprotein via interaction with the TLC1 guide RNA; relocates to sit	71824	2	1	0,06
16	YER005W	Apyrase with wide substrate specificity; helps prevent inhibition of glycosylation by hydrolyzing nucleoside tri- and diphosphates that inhibit glycotransferases; partially redundant with Gda1p; mediates adenovirus E4orf4-induced toxicity	72434	2	1,3	0,06
27	YPL020C	Protease that specifically cleaves Smt3p protein conjugates; required for cell cycle progression; associates with nucleoporins and may interact with septin rings during telophase; sequestered to the nucleolus under stress conditions	72618	1	1,1	0,06
13	YMR058W	Ferro-O2-oxidoreductase; multicopper oxidase that oxidizes ferrous (Fe2+) to ferric iron (Fe3+) for subsequent cellular uptake by transmembrane permease Ftr1p; required for high-affinity iron uptake and involved in mediating resistance to copper ion	72656	2	0,9	0,06
19	YKL179C	Golgi membrane protein with similarity to mammalian CASP; genetic interactions with GOS1 (encoding a Golgi snare protein) suggest a role in Golgi function	77524	1	1	0,06
29	YHR031C	DNA helicase involved in rDNA replication and Ty1 transposition; binds to and suppresses DNA damage at G4 motifs in vivo; relieves replication fork pauses at telomeric regions; structurally and functionally related to Pif1p	81758	1	1,4	0,05
14	YMR089C	Mitochondrial inner membrane m-AAA protease component; mediates degradation of misfolded or unassembled proteins; also required for correct assembly of mitochondrial enzyme complexes	93503	1	0,7	0,05
30	YLL013C	Protein of the mitochondrial outer surface; links the Arp2/3 complex with the mitochore during anterograde mitochondrial movement; also binds to and promotes degradation of mRNAs for select nuclear-encoded mitochondrial proteins	98178	1	0,8	0,04
23	YDL102W	Catalytic subunit of DNA polymerase delta; required for chromosomal DNA replication during mitosis and meiosis, intragenic recombination, repair of double strand DNA breaks, and DNA replication during nucleotide excision repair (NER)	125680	1	0,5	0,03
24	YBR245C	ATPase subunit of imitation-switch (ISWI) class chromatin remodelers; with Ioc3p forms Isw1a complex involved in repression of transcription initiation; with Ioc2p and Ioc4p forms Isw1b complex involved in regulation of transcription elongation; Isw	131534	1	0,5	0,03
20	YFR016C	Putative protein of unknown function; green fluorescent protein (GFP)-fusion protein localizes to the cytoplasm and bud; interacts with Spa2p; YFR016C is not an essential gene	137844	2	0,6	0,03
34	YDR159W	mRNA export factor; required for biogenesis of the small ribosomal subunit; component of TREX-2 complex (Sac3p-Thp1p-Sus1p-Cdc31p) involved in transcription elongation and mRNA export from the nucleus; involved in post-transcriptional tethering of a	150217	1	0,5	0,03
38	YKL101W	Nim1p-related protein kinase; regulates the morphogenesis and septin checkpoints; associates with the assembled septin filament; required along with Hsl7p for bud neck recruitment, phosphorylation, and degradation of Swe1p	169902	1	1	0,03
31	YJL207C	AP-1 accessory protein; colocalizes with clathrin to the late-Golgi apparatus; involved in TGN-endosome transport; physically interacts with AP-1; similar to the mammalian p200; may interact with ribosomes; YJL207C is a non-essential gene	231123	1	0,3	0,02
32	YLR106C	Huge dynein-related AAA-type ATPase (midasin); forms extended pre-60S particle with the Rix1 complex (Rix1p-Ipi1p-Ipi3p); acts in removal of ribosomal biogenesis factors at successive steps of pre-60S assembly and export from nucleus	561182	1	0,2	0,01

BAND 6

prot_hit_num	prot_acc	prot_desc	prot_mass	prot_matches	prot_cover	emPAI
6	YLR295C	Subunit h of the F0 sector of mitochondrial F1F0 ATP synthase; F1F0 ATP synthase is a large, evolutionarily conserved enzyme complex required for ATP synthesis; protein abundance increases in response to DNA replication stress	14176	2	11,3	0,34
9	YBL002W	Histone H2B; core histone protein required for chromatin assembly and chromosome function; nearly identical to HTB1; Rad6p-Bre1p-Lge1p mediated ubiquitination regulates reassembly after DNA replication, transcriptional activation, meiotic DSB format	14229	3	17,6	1,4
7	YER072W	Subunit of the vacuolar transporter chaperone (VTC) complex; VTC complex is involved in membrane trafficking, vacuolar polyphosphate accumulation, microautophagy and non-autophagic vacuolar fusion; also has mRNA binding activity; protein abundance i	14419	3	17,1	1,37
4	YDR529C	Subunit 7 of ubiquinol cytochrome-c reductase (Complex III); Complex III is a component of the mitochondrial inner membrane electron transport chain; oriented facing the mitochondrial matrix; N-terminus appears to play a role in complex assembly	14613	7	29,9	2,1
2	YBR230C	Mitochondrial outer membrane receptor for cytosolic ribosomes; integral protein of the outer membrane that interacts with the nascent chain-associated complex (NAC) bound to ribosomes, contributing to co-translational mitochondrial import; interacts	14885	14	16,4	4,33
10	YLR104W	Putative protein of unknown function; mutant is deficient in amounts of cell wall mannosylphosphate and has long chronological lifespan; genetic interactions suggest a role in ER-associated protein degradation (ERAD)	15368	2	11,5	0,72
3	YOR285W	Thiosulfate sulfurtransferase; contains a rhodanese-like domain; localized to the mitochondrial outer membrane; protein abundance increases in response to DNA replication stress; similar to the human TSTD gene	15461	7	43,9	2,84
11	YDR513W	Cytoplasmic glutaredoxin; thioltransferase, glutathione-dependent disulfide oxidoreductase involved in maintaining redox state of target proteins, also exhibits glutathione peroxidase activity, expression induced in response to stress; GRX2 has two	15965	1	4,9	0,3
1	YGL187C	Subunit IV of cytochrome c oxidase; the terminal member of the mitochondrial inner membrane electron transport chain; precursor N-terminal 25 residues are cleaved during mitochondrial import; phosphorylated; spermidine enhances translation	17360	20	55,5	4,38
8	YGR174C	Mitochondrial protein required for assembly of cytochrome bc1 complex; interacts with the Cbp3p-Cbp6p complex and newly synthesized cytochrome b (Cobp) to promote assembly of Cobp into the cytochrome bc1 complex	20264	3	16,5	0,85
5	YDR178W	Membrane anchor subunit of succinate dehydrogenase (SDH); involved in coupling the oxidation of succinate to the transfer of electrons to ubiquinone as part of the TCA cycle and the mitochondrial respiratory chain; has similarity to human SDH subuni	20349	2	14,9	0,51

19	YPL090C	Protein component of the small (40S) ribosomal subunit; homologous to mammalian ribosomal protein S6, no bacterial homolog; RPS6A has a paralog, RPS6B, that arose from the whole genome duplication	27037	1	3,4	0,17
23	YAR015W	N-succinyl-5-aminoimidazole-4-carboxamide ribotide synthetase; required for ~de novo~ purine nucleotide biosynthesis; red pigment accumulates in mutant cells deprived of adenine; protein abundance increases in response to DNA replication stress	34639	1	2,3	0,13
28	YLR100W	3-keto sterol reductase; catalyzes the last of three steps required to remove two C-4 methyl groups from an intermediate in ergosterol biosynthesis; mutants are sterol auxotrophs	39757	1	2,3	0,11
25	YKL042W	Central plaque component of spindle pole body (SPB); involved in SPB duplication, may facilitate attachment of the SPB to the nuclear membrane	42359	1	2,2	0,1
16	YCL029C	Microtubule-associated protein; component of the interface between microtubules and kinetochore, involved in sister chromatid separation; essential in polyploid cells but not in haploid or diploid cells; ortholog of mammalian CLIP-170	51288	1	1,4	0,09
20	YPL224C	Putative metal transporter involved in mitochondrial iron accumulation; MMT2 has a paralog, MMT1, that arose from the whole genome duplication	52516	1	1,2	0,08
26	YER086W	Threonine deaminase, catalyzes first step in isoleucine biosynthesis; expression is under general amino acid control; ILV1 locus exhibits highly positioned nucleosomes whose organization is independent of known ILV1 regulation	64076	1	1	0,07
24	YNL094W	Phosphatidate phosphatase, converts phosphatidate to diacylglycerol; App1p, Pah1p, Dpp1p, and Lpp1p are responsible for all the phosphatidate phosphatase activity; component of cortical actin patches; interacts with components of endocytic pathway	66378	1	1,2	0,07
12	YMR106C	Subunit of the telomeric Ku complex (Yku70p-Yku80p); involved in telomere length maintenance, structure and telomere position effect; required for localization of telomerase ribonucleoprotein via interaction with the TLC1 guide RNA; relocates to sit	71824	1	1	0,06
14	YER005W	Apyrase with wide substrate specificity; helps prevent inhibition of glycosylation by hydrolyzing nucleoside tri- and diphosphates that inhibit glycotransferases; partially redundant with Gda1p; mediates adenovirus E4orf4-induced toxicity	72434	2	1,3	0,06
22	YPL020C	Protease that specifically cleaves Smt3p protein conjugates; required for cell cycle progression; associates with nucleoporins and may interact with septin rings during telophase; sequestered to the nucleolus under stress conditions	72618	1	1,1	0,06
15	YNR019W	Acyl-CoA:sterol acyltransferase; endoplasmic reticulum enzyme that contributes the major sterol esterification activity in the presence of oxygen; ARE2 has a paralog, ARE1, that arose from the whole genome duplication	74431	1	1,1	0,06
18	YGL026C	Tryptophan synthase; catalyzes the last step of tryptophan biosynthesis; regulated by the general control system of amino acid biosynthesis	76977	1	1	0,06
30	YDR436W	Serine/threonine protein phosphatase Z, isoform of Ppz1p; involved in regulation of potassium transport, which affects osmotic stability, cell cycle progression, and halotolerance	79184	1	1,3	0,06
29	YMR186W	Cytoplasmic chaperone of the Hsp90 family; plays a role in determining prion variants; redundant in function and nearly identical with Hsp82p, and together they are essential; expressed constitutively at 10-fold higher basal levels than HSP82 and in	80850	1	1	0,05
17	YMR089C	Mitochondrial inner membrane m-AAA protease component; mediates degradation of misfolded or unassembled proteins; also required for correct assembly of mitochondrial enzyme complexes	93503	1	0,7	0,05
13	YNL139C	Subunit of the THO complex; THO is required for efficient transcription elongation and involved in transcriptional elongation-associated recombination; required for LacZ RNA expression from certain plasmids	184671	1	0,4	0,02
21	YIL149C	Myosin-like protein associated with the nuclear envelope; nuclear basket protein that connects the nuclear pore complex with the nuclear interior; involved in the Tel1p pathway that controls telomere length; MLP2 has a paralog, MLP1, that arose from	195934	1	0,5	0,02
27	YBR136W	Genome integrity checkpoint protein and PI kinase superfamily member; Mec1p and Dun1p function in same pathway to regulate dNTP pools and telomere length; signal transducer required for cell cycle arrest and transcriptional responses to damaged or u	275108	1	0,6	0,02

BAND 7

prot_hit_num	prot_acc	prot_desc	prot_score	prot_mass	prot_matches	prot_matches_sig	prot_sequences	prot_sequences_sig	prot_cover	emPAI
1	YKL016C	Subunit d of the stator stalk of mitochondrial F1F0 ATP synthase; F1F0 ATP synthase is a large, evolutionarily conserved enzyme complex required for ATP synthesis	1427	19797	103	55	30	21	84,5	242,08
2	YDR032C	Protein with similarity to a family of flavodoxin-like proteins; induced by oxidative stress in a Yap1p dependent manner; the authentic, non-tagged protein is detected in highly purified mitochondria in high-throughput studies; protein abundance inc	193	21010	6	5	4	4	19,7	
2	YCR004C	Protein of unknown function; has sequence and structural similarity to flavodoxins; predicted to be palmitoylated; the authentic, non-tagged protein is detected in highly purified mitochondria in high-throughput studies	44	26505	1	1	1	1	2,8	
3	YMR264W	Ubiquitin-binding protein; endoplasmic reticulum membrane protein that recruits the ubiquitin-conjugating enzyme Ubc7p to the ER where it functions in protein degradation; contains a CUE domain that binds ubiquitin to facilitate intramolecular monou	152	22806	3	2	3	2	19,2	
4	YKL065C	Endoplasmic reticulum transmembrane protein; may interact with ribosomes, based on co-purification experiments; homolog of human BAP31 protein; YET1 has a paralog, YET2, that arose from the whole genome duplication	93	23559	3	2	3	2	15	

5	YPR165W	GTP-binding protein of the rho subfamily of Ras-like proteins; involved in establishment of cell polarity; regulates protein kinase C (Pkc1p) and the cell wall synthesizing enzyme 1,3-beta-glucan synthase (Fks1p and Gsc2p)	50	23537	1	1	1	1	4,8
6	YMR106C	Subunit of the telomeric Ku complex (Yku70p-Yku80p); involved in telomere length maintenance, structure and telomere position effect; required for localization of telomerase ribonucleoprotein via interaction with the TLC1 guide RNA; relocates to sit	44	71824	1	1	1	1	1
7	YCL009C	Regulatory subunit of acetolactate synthase; acetolactate synthase catalyzes the first step of branched-chain amino acid biosynthesis; enhances activity of the Ilv2p catalytic subunit, localizes to mitochondria	44	34308	1	1	1	1	1,9
8	YGR049W	Mitochondrial outer membrane protein of unknown function; predicted to have 4 transmembrane segments; import is mediated by Tom70p and Mim1p; interacts genetically with a cdc4 mutation; SCM4 has a paralog, ATG33, that arose from the whole genome dup	42	20505	3	1	3	1	13,4

BAND 8

prot_hit_num	prot_acc	prot_desc	prot_mass	prot_matches	prot_cover	emPAI
6	YOR327C	Vesicle membrane receptor protein (v-SNARE); involved in the fusion between Golgi-derived secretory vesicles with the plasma membrane; Snc2p levels regulated by Vps45p; member of the synaptobrevin/VAMP family of R-type v-SNARE proteins; SNC2 has a p	13006	3	26,1	0,89
3	YMR123W	V-ATPase assembly factor; functions with other V-ATPase assembly factors in the ER to efficiently assemble the V-ATPase membrane sector (V0); protein abundance increases in response to DNA replication stress	14010	6	29,5	1,43
2	YNL149C	Essential protein required for maturation of Gas1p and Pho8p; involved in protein trafficking; GFP-fusion protein localizes to the ER and YFP-fusion protein to the nuclear envelope-ER network; null mutants have a cell separation defect	15044	6	18,6	2
8	YNL131W	Component of the TOM (Translocase of Outer Membrane) complex; responsible for initial import of mitochondrially directed proteins; mediates interaction between TOM and TIM complexes and acts as a receptor for precursor proteins	16780	6	16,4	0,28
4	YEL034W	Translation elongation factor eIF-5A; required for translation of proteins containing polyproline stretches, including Bni1p, and this leads to a requirement for mating projection formation; structural homolog of bacterial EF-P; undergoes an essenti	17217	2	16,6	0,62
12	YEL054C	Ribosomal 60S subunit protein L12A; rpl12a rpl12b double mutant exhibits slow growth and slow translation; homologous to mammalian ribosomal protein L12 and bacterial L11; RPL12A has a paralog, RPL12B, that arose from the whole genome duplication	17869	6	29,1	1,01
15	YOR150W	Mitochondrial ribosomal protein of the large subunit; localizes to vacuole in response to H2O2	18566	4	21,5	0,57
9	YGR076C	Mitochondrial ribosomal protein of the large subunit; mutation confers increased replicative lifespan	18575	5	31,2	0,96
26	YML030W	Cytochrome c oxidase subunit; required for assembly of the Complex III-Complex IV supercomplex, and for assembly of Cox13p and Rcf2p into cytochrome c oxidase; similar to Rcf2p, and either Rcf1p or Rcf2p is required for late-stage assembly of the Co	18580	4	20,8	0,57
25	YIR022W	18kDa catalytic subunit of the Signal Peptidase Complex (SPC); the Signal Peptidase Complex cleaves the signal sequence of proteins targeted to the endoplasmic reticulum; other members are Spc1p, Spc2p, Spc3p, and Sec11p	18807	1	9	0,25
1	YPR149W	Protein of unknown function; contains transmembrane domains; involved in secretion of proteins that lack classical secretory signal sequences; component of the detergent-insoluble glycolipid-enriched complexes (DIGs); NCE102 has a paralog, FHN1, tha	19240	15	26	3,58
11	YPR028W	Membrane protein that interacts with Yip1p to mediate membrane traffic; interacts with Sey1p to maintain ER morphology; overexpression leads to cell death and accumulation of internal cell membranes; mutants have reduced phosphatidylserine transfer	20257	7	18,9	0,86
35	YGR049W	Mitochondrial outer membrane protein of unknown function; predicted to have 4 transmembrane segments; import is mediated by Tom70p and Mim1p; interacts genetically with a cdc4 mutation; SCM4 has a paralog, ATG33, that arose from the whole genome dup	20505	4	8	0,23
14	YMR072W	Mitochondrial DNA-binding protein; involved in mitochondrial DNA replication and recombination, member of HMG1 DNA-binding protein family; activity may be regulated by protein kinase A phosphorylation; ABF2 has a paralog, IXR1, that arose from the w	21548	11	38,8	1,64
29	YDL072C	Protein of unknown function; YET3 null mutant decreases the level of secreted invertase; homolog of human BAP31 protein; protein abundance increases in response to DNA replication stress	23002	1	5,4	0,2
33	YNR018W	Cytochrome c oxidase subunit; has a role in assembly of respiratory supercomplexes; similar to Rcf1p, and either Rcf1p or Rcf2p is required for late-stage assembly of the Cox12p and Cox13p subunits and for cytochrome c oxidase activity; associates w	25385	1	4	0,18
17	YCR004C	Protein of unknown function; has sequence and structural similarity to flavodoxins; predicted to be palmitoylated; the authentic, non-tagged protein is detected in highly purified mitochondria in high-throughput studies	26505	5	17,8	0,61

BAND 9

53	YJR045C	Hsp70 family ATPase; constituent of the import motor component of the Translocase of the Inner Mitochondrial membrane (TIM23 complex); involved in protein translocation and folding; subunit of SceI endonuclease; SSC1 has a paralog, ECM10, that arose	70585	1	2,4	0,06
21	YMR106C	Subunit of the telomeric Ku complex (Yku70p-Yku80p); involved in telomere length maintenance, structure and telomere position effect; required for localization of telomerase ribonucleoprotein via interaction with the TLC1 guide RNA; relocates to sit	71824	3	1	0,13
39	YER005W	Apyrase with wide substrate specificity; helps prevent inhibition of glycosylation by hydrolyzing nucleoside tri- and diphosphates that inhibit glycotransferases; partially redundant with Gda1p; mediates adenovirus E4orf4-induced toxicity	72434	3	1,3	0,06
46	YGL026C	Tryptophan synthase; catalyzes the last step of tryptophan biosynthesis; regulated by the general control system of amino acid biosynthesis	76977	1	1	0,06
48	YKL179C	Golgi membrane protein with similarity to mammalian CASP; genetic interactions with GOS1 (encoding a Golgi snare protein) suggest a role in Golgi function	77524	1	1,2	0,06
42	YKR001C	Dynamin-like GTPase required for vacuolar sorting; also involved in actin cytoskeleton organization, endocytosis, late Golgi-retention of some proteins, regulation of peroxisome biogenesis	78802	1	1	0,06
35	YHR031C	DNA helicase involved in rDNA replication and Ty1 transposition; binds to and suppresses DNA damage at G4 motifs in vivo; relieves replication fork pauses at telomeric regions; structurally and functionally related to Pif1p	81758	3	1,4	0,11
60	YDL239C	Protein required for spore wall formation; thought to mediate assembly of a Don1p-containing structure at the leading edge of the prospore membrane via interaction with spindle pole body components; potentially phosphorylated by Cdc28p; ADY3 has a p	91984	1	0,8	0,05
44	YMR089C	Mitochondrial inner membrane m-AAA protease component; mediates degradation of misfolded or unassembled proteins; also required for correct assembly of mitochondrial enzyme complexes	93503	1	0,7	0,05
66	YKL050C	Protein of unknown function; the YKL050W protein is a target of the SCFCdc4 ubiquitin ligase complex and YKL050W transcription is regulated by Azf1p; YKL050C has a paralog, EIS1, that arose from the whole genome duplication	103423	2	0,8	0,04
49	YBR112C	General transcriptional co-repressor; acts together with Tup1p; also acts as part of a transcriptional co-activator complex that recruits the SWI/SNF and SAGA complexes to promoters; can form the prion [OCT+]	107422	1	1	0,04
27	YLL003W	Centrin (Cdc31p)-binding protein required for SPB duplication; localizes to the half-bridge of the spindle pole body (SPB); required for progression through G(2)-M transition; phosphorylated by Cdc28p-Clb2p and by Cdc5p; dephosphorylated by Cdc14p;	113364	1	0,6	0,04
64	YOL145C	Component of the Paf1p complex involved in transcription elongation; binds to and modulates the activity of RNA polymerases I and II; required for expression of a subset of genes, including cyclin genes; involved in SER3 repression by helping to mai	125031	1	0,8	0,03
63	YNL106C	Polyphosphatidylinositol phosphatase; dephosphorylates a number of phosphatidylinositol phosphates (PtdInsPs, PIPs) to PI; involved in endocytosis; hyperosmotic stress causes translocation to actin patches; synaptojanin-like protein with a Sac1 doma	134047	1	0,8	0,03
68	YJL092W	DNA helicase and DNA-dependent ATPase; involved in DNA repair and checkpoint recovery, needed for proper timing of commitment to meiotic recombination and transition from Meiosis I to II; blocks trinucleotide repeat expansion; affects genome stabili	134679	1	0,5	0,03
55	YPR026W	Acid trehalase required for utilization of extracellular trehalose; involved in intracellular trehalose degradation during growth recovery after saline stress	137120	3	0,6	0,03
54	YJR137C	Sulfite reductase beta subunit; involved in amino acid biosynthesis, transcription repressed by methionine	161519	3	1,2	0,05

BAND 10

prot_hit_num	prot_acc	prot_desc	prot_mass	prot_matches	prot_cover	emPAI
22	YML081C-A	Subunit of the mitochondrial F1F0 ATP synthase; F1F0 ATP synthase is a large, evolutionarily conserved enzyme complex required for ATP synthesis; termed subunit I or subunit j; does not correspond to known ATP synthase subunits in other organisms	6683	4	13,6	2,33
1	YPL271W	Epsilon subunit of the F1 sector of mitochondrial F1F0 ATP synthase; which is a large, evolutionarily conserved enzyme complex required for ATP synthesis; F1 translationally regulates ATP6 and ATP8 expression to achieve a balanced output of ATP synt	6738	20	41,9	4,91
31	YLR388W	Protein component of the small (40S) ribosomal subunit; homologous to mammalian ribosomal protein S29 and bacterial S14; RPS29A has a paralog, RPS29B, that arose from the whole genome duplication	6888	2	14,3	0,79
49	YMR256C	Subunit VII of cytochrome c oxidase (Complex IV); Complex IV is the terminal member of the mitochondrial inner membrane electron transport chain	6928	1	10	0,79
20	YDL067C	Subunit VIIa of cytochrome c oxidase (Complex IV); Complex IV is the terminal member of the mitochondrial inner membrane electron transport chain	6959	3	30,5	2,16
32	YOL077W-A	Subunit k of the mitochondrial F1F0 ATP synthase; F1F0 ATP synthase is a large, evolutionarily conserved enzyme complex required for ATP synthesis; associated only with the dimeric form of ATP synthase	7529	2	10,3	0,71
50	YIR021W-A	Putative protein of unknown function; identified by expression profiling and mass spectrometry	7733	2	10	0,68
48	YBR126W-A	Protein of unknown function; identified by gene-trapping, microarray analysis, and genome-wide homology searches; mRNA identified as translated by ribosome profiling data; partially overlaps the dubious ORF YBR126W-B	7870	1	14,7	0,67
5	YPR010C-A	Putative protein of unknown function; conserved among Saccharomyces sensu stricto species	7955	4	33,3	1,75

30	YLR325C	Ribosomal 60S subunit protein L38; homologous to mammalian ribosomal protein L38, no bacterial homolog	8821	2	28,2	1,53
36	YLR395C	Subunit VIII of cytochrome c oxidase (Complex IV); Complex IV is the terminal member of the mitochondrial inner membrane electron transport chain	9017	1	12,8	0,57
21	YHR021C	Protein component of the small (40S) ribosomal subunit; homologous to mammalian ribosomal protein S27, no bacterial homolog; RPS27B has a paralog, RPS27A, that arose from the whole genome duplication	9150	3	36,6	2,82
34	YDR379C-A	Mitochondrial protein involved in assembly of succinate dehydrogenase; has a role in maturation of the Sdh2p subunit; member of the LYR protein family; mutations in human ortholog SDHAF1 are associated with infantile leukoencephalopathy	9346	3	30,4	1,4
14	YER074W-A	Integral membrane protein required for ER to Golgi transport; localized to the Golgi, the ER, and COPII vesicles; interacts with Yip1p and Yif1p	9430	3	27,1	1,37
9	YER019C-A	Ssh1p-Sss1p-Sbh2p complex component; involved in protein translocation into the endoplasmic reticulum; SBH2 has a paralog, SBH1, that arose from the whole genome duplication	9600	2	14,8	0,53
4	YDL181W	Protein that inhibits ATP hydrolysis by the F1F0-ATP synthase; inhibitory function is enhanced by stabilizing proteins Stf1p and Stf2p; has a calmodulin-binding motif and binds calmodulin in vitro; INH1 has a paralog, STF1, that arose from the whole	9864	8	30,6	4,22
51	YLR038C	Subunit VIb of cytochrome c oxidase; cytochrome c oxidase is also known as respiratory Complex IV and is the terminal member of the mitochondrial inner membrane electron transport chain; required for assembly of cytochrome c oxidase but not required	10014	1	9,6	0,5
93	YNR034W-A	Putative protein of unknown function; expression is regulated by Msn2p/Msn4p; YNR034W-A has a paralog, YCR075W-A, that arose from the whole genome duplication	10775	1	8,2	0,46
16	YDL045W-A	Mitochondrial ribosomal protein of the small subunit; contains twin cysteine-x9-cysteine motifs; oxidized by Mia40p during import into mitochondria	10916	4	9,5	0,46
12	YDR322C-A	Subunit e of mitochondrial F1F0-ATPase; ATPase is a large, evolutionarily conserved enzyme complex required for ATP synthesis; essential for the dimeric and oligomeric state of ATP synthase, which in turn determines the shape of inner membrane crist	10927	2	30,2	1,12
116	YMR194W	Ribosomal 60S subunit protein L36A; N-terminally acetylated; binds to 5.8 S rRNA; homologous to mammalian ribosomal protein L36, no bacterial homolog; RPL36A has a paralog, RPL36B, that arose from the whole genome duplication	11117	1	10	0,45
8	YDR377W	Subunit f of the F0 sector of mitochondrial F1F0 ATP synthase; F1F0 ATP synthase is a large, evolutionarily conserved enzyme complex required for ATP synthesis	11305	5	19,8	1,06
18	YLR043C	Cytoplasmic thioredoxin isoenzyme; part of thioredoxin system which protects cells against oxidative and reductive stress; forms LMA1 complex with Pbi2p; acts as a cofactor for Tsa1p; required for ER-Golgi transport and vacuole inheritance; with Trx	11344	3	35,9	1,97
94	YBR009C	Histone H4; core histone protein required for chromatin assembly and chromosome function; one of two identical histone proteins (see also HHF2); contributes to telomeric silencing; N-terminal domain involved in maintaining genomic integrity	11361	1	11,7	0,44
29	YDR115W	Putative mitochondrial ribosomal protein of the large subunit; similar to E. coli L34 ribosomal protein; required for respiratory growth, as are most mitochondrial ribosomal proteins; protein increases in abundance and relocalizes to the plasma memb	12081	2	16,2	0,97
45	YBR262C	Component of the MICOS complex; MICOS (formerly MINOS or MitOS) is a mitochondrial inner membrane complex that extends into the intermembrane space and has a role in the maintenance of crista junctions, inner membrane architecture, and formation of	12380	1	10,4	0,39
17	YLR390W	Putative protein of unknown function; the authentic, non-tagged protein is detected in highly purified mitochondria in high-throughput studies	12624	2	22,3	0,92
38	YPR020W	Subunit g of the mitochondrial F1F0 ATP synthase; reversibly phosphorylated on two residues; unphosphorylated form is required for dimerization of the ATP synthase complex, which in turn determines oligomerization of the complex and the shape of inn	12971	2	15,7	0,89
40	YBL003C	Histone H2A; core histone protein required for chromatin assembly and chromosome function; one of two nearly identical (see also HTA1) subtypes; DNA damage-dependent phosphorylation by Mec1p facilitates DNA repair; acetylated by Nat4p	13981	3	12,1	0,8
40	YOL012C	Histone variant H2AZ; exchanged for histone H2A in nucleosomes by the SWR1 complex; involved in transcriptional regulation through prevention of the spread of silent heterochromatin; Htz1p-containing nucleosomes facilitate RNA Pol II passage by affe	14274	2	11,9	0,78
119	YJR114W	Dubious open reading frame; unlikely to encode a functional protein, based on available experimental and comparative sequence data; partially overlaps the verified ORF RSM7/YJR113C	14304	1	7,7	0,33
25	YBL087C	Ribosomal 60S subunit protein L23A; homologous to mammalian ribosomal protein L23 and bacterial L14; RPL23A has a paralog, RPL23B, that arose from the whole genome duplication	14580	3	16,8	0,77
26	YCR031C	Protein component of the small (40S) ribosomal subunit; required for ribosome assembly and 20S pre-rRNA processing; mutations confer cryptopleurine resistance; homologous to mammalian ribosomal protein S14 and bacterial S11; RPS14A has a paralog, RP	14586	2	9,5	0,33
13	YDR529C	Subunit 7 of ubiquinol cytochrome-c reductase (Complex III); Complex III is a component of the mitochondrial inner membrane electron transport chain; oriented facing the mitochondrial matrix; N-terminus appears to play a role in complex assembly	14614	3	18,9	0,76
19	YKR094C	Ubiquitin-ribosomal 60S subunit protein L40B fusion protein; cleaved to yield ubiquitin and ribosomal protein L40B; ubiquitin may facilitate assembly of the ribosomal protein into ribosomes; homologous to mammalian ribosomal protein L40, no bacteria	14835	2	7	0,32
28	YGL191W	Subunit VIa of cytochrome c oxidase; present in a subclass of cytochrome c oxidase complexes that may have a role in mimimizing generation of reactive oxygen species; not essential for cytochrome c oxidase activity but may modulate activity in respo	15070	4	22,5	1,28

46	YBR010W	Histone H3; core histone protein required for chromatin assembly, part of heterochromatin-mediated telomeric and HM silencing; one of two identical histone H3 proteins (see HHT2); regulated by acetylation, methylation, and phosphorylation; H3K14 ace	15347	2	5,1	0,31
7	YOR285W	Thiosulfate sulfurtransferase; contains a rhodanese-like domain; localized to the mitochondrial outer membrane; protein abundance increases in response to DNA replication stress; similar to the human TSTD gene	15462	5	18,7	0,71
88	YOL121C	Protein component of the small (40S) ribosomal subunit; required for assembly and maturation of pre-40 S particles; homologous to mammalian ribosomal protein S19, no bacterial homolog; mutations in human RPS19 are associated with Diamond Blackfan an	15907	1	7,6	0,3
15	YOL040C	Protein component of the small (40S) ribosomal subunit; homologous to mammalian ribosomal protein S15 and bacterial S19	15992	2	9,2	0,3
24	YNR022C	Mitochondrial ribosomal protein of the large subunit; not essential for mitochondrial translation	16389	4	21,6	1,13
105	YML026C	Protein component of the small (40S) ribosomal subunit; homologous to mammalian ribosomal protein S18 and bacterial S13; RPS18B has a paralog, RPS18A, that arose from the whole genome duplication; protein abundance increases in response to DNA repli	17085	1	7,5	0,27
33	YLR008C	Subunit of the import motor (PAM complex); the PAM complex is a component of the Translocase of the Inner Mitochondrial membrane (TIM23 complex); essential J-protein cochaperone that stimulates Ssc1p ATPase activity to drive import; inhibited by Pa	17899	1	8,9	0,26
123	YDL094C	Dubious open reading frame; unlikely to encode a functional protein, based on available experimental and comparative sequence data; partially overlaps verfied gene PMT5/YDL093W; YDL094C is not essential	19854	1	5,9	0,23
42	YGR174C	Mitochondrial protein required for assembly of cytochrome bc1 complex; interacts with the Cbp3p-Cbp6p complex and newly synthesized cytochrome b (Cobp) to promote assembly of Cobp into the cytochrome bc1 complex	20265	1	10,6	0,23
68	YGR082W	Component of the TOM (translocase of outer membrane) complex; responsible for recognition and initial import steps for all mitochondrially directed proteins; acts as a receptor for incoming precursor proteins	20304	1	3,3	0,23
6	YBL090W	Mitochondrial ribosomal protein of the small subunit; MRP21 exhibits genetic interactions with mutations in the COX2 and COX3 mRNA 5~-untranslated leader sequences	20440	5	14,1	1,26
74	YML011C	Protein involved in nucleotide excision repair; green fluorescent protein (GFP)-fusion protein localizes to the nucleus	20547	1	4,5	0,22
135	YMR255W	Coiled-coiled protein of unknown function; identified as a high-copy suppressor of a dbp5 mutation; protein abundance increases in response to DNA replication stress	21558	1	3,7	0,21
100	YJL030W	Component of the spindle-assembly checkpoint complex; delays onset of anaphase in cells with defects in mitotic spindle assembly; forms a complex with Mad1p; regulates APC/C activity during prometaphase and metaphase of meiosis I; gene dosage imbala	22386	2	6,1	0,2
11	YDR298C	Subunit 5 of the stator stalk of mitochondrial F1F0 ATP synthase; F1F0 ATP synthase is a large, evolutionarily conserved enzyme complex required for ATP synthesis; homologous to bovine subunit OSCP (oligomycin sensitivity-conferring protein); phosph	22858	3	10,8	0,44
10	YNR018W	Cytochrome c oxidase subunit; has a role in assembly of respiratory supercomplexes; similar to Rcf1p, and either Rcf1p or Rcf2p is required for late-stage assembly of the Cox12p and Cox13p subunits and for cytochrome c oxidase activity; associates w	25386	2	4,9	0,18
47	YPL078C	Subunit b of the stator stalk of mitochondrial F1F0 ATP synthase; ATP synthase is a large, evolutionarily conserved enzyme complex required for ATP synthesis; contributes to the oligomerization of the complex, which in turn determines the shape of i	26966	2	7	0,36
65	YPL090C	Protein component of the small (40S) ribosomal subunit; homologous to mammalian ribosomal protein S6, no bacterial homolog; RPS6A has a paralog, RPS6B, that arose from the whole genome duplication	27038	2	8,9	0,36
62	YJL063C	Mitochondrial ribosomal protein of the large subunit	27044	1	2,5	0,17
113	YKL009W	Protein involved in mRNA turnover and ribosome assembly; required at post-transcriptional step for efficient retrotransposition; localizes to the nucleolus	27157	1	3,4	0,17
107	YGR271C-A	Essential protein required for maturation of 18S rRNA; null mutant is sensitive to hydroxyurea and is delayed in recovering from alpha-factor arrest; green fluorescent protein (GFP)-fusion protein localizes to the nucleolus	27163	1	4,3	0,17
69	YER188W	Dubious open reading frame; unlikely to encode a functional protein, based on available experimental and comparative sequence data; large-scale analyses show mRNA expression increases under anaerobic conditions and two-hybrid interactions with Sst2p	27816	2	5	0,16
97	YNL129W	Nicotinamide riboside kinase; catalyzes the phosphorylation of nicotinamide riboside and nicotinic acid riboside in salvage pathways for NAD+ biosynthesis	27846	1	3,8	0,16
53	YOR145C	Essential nucleolar protein required for pre-18S rRNA processing; interacts with Dim1p, an 18S rRNA dimethyltransferase, and also with Nob1p, which is involved in proteasome biogenesis; contains a KH domain	30313	2	4	0,15
86	YPR183W	Dolichol phosphate mannose (Dol-P-Man) synthase of the ER membrane; catalyzes the formation of Dol-P-Man from Dol-P and GDP-Man; required for glycosyl phosphatidylinositol membrane anchoring, O mannosylation, and protein glycosylation	30517	1	3,7	0,15
79	YLR284C	Peroxisomal delta3,delta2-enoyl-CoA isomerase; hexameric protein that converts 3-hexenoyl-CoA to trans-2-hexenoyl-CoA, essential for the beta-oxidation of unsaturated fatty acids, oleate-induced; ECI1 has a paralog, DCI1, that arose from the whole g	32026	1	2,1	0,14
3	YJR077C	Mitochondrial phosphate carrier; imports inorganic phosphate into mitochondria; functionally redundant with Pic2p but more abundant than Pic2p under normal conditions; phosphorylated	32965	7	15,4	0,89

117	YIL124W	Bifunctional triacylglycerol lipase and 1-acyl DHAP reductase; NADPH-dependent 1-acyl dihydroxyacetone phosphate reductase involved in phosphatidic acid biosynthesis; lipid droplet triacylglycerol lipase involved in the mobilization of non-polar lip	33025	1	6,7	0,14
120	YLR003C	Putative subunit of the 90S preribosome processome complex; overexpression rescues supressor mutant of mcm10; null mutant is viable; relocalizes from nucleus to cytoplasm upon DNA replication stress	33490	1	3,4	0,13
39	YLR340W	Conserved ribosomal protein P0 of the ribosomal stalk; involved in interaction between translational elongation factors and the ribosome; phosphorylated on serine 302; homologous to mammalian ribosomal protein LP0 and bacterial L10	33696	1	3,5	0,13
142	YFR041C	Type I membrane protein with a J domain; required to preserve the folding capacity of the endoplasmic reticulum; loss of the non-essential ERJ5 gene leads to a constitutively induced unfolded protein response	34286	2	3,1	0,13
58	YJR009C	Glyceraldehyde-3-phosphate dehydrogenase (GAPDH), isozyme 2; involved in glycolysis and gluconeogenesis; tetramer that catalyzes reaction of glyceraldehyde-3-phosphate to 1,3 bis-phosphoglycerate; detected in cytoplasm and cell wall; protein abundan	35940	1	2,1	0,12
37	YJR141W	Essential protein of unknown function	40456	2	2,3	0,11
78	YPR191W	Subunit 2 of ubiquinol cytochrome-c reductase (Complex III); Complex III is a component of the mitochondrial inner membrane electron transport chain; phosphorylated; transcription is regulated by Hap1p, Hap2p/Hap3p, and heme	40511	1	2,4	0,11
75	YNL134C	Protein of unknown function; similar to dehydrogenases from other model organisms; green fluorescent protein (GFP)-fusion protein localizes to both the cytoplasm and nucleus; protein abundance increases in response to DNA replication stress	41371	1	3,2	0,11
27	YFL039C	Actin; structural protein involved in cell polarization, endocytosis, and other cytoskeletal functions	41895	2	2,7	0,11
114	YER048C	Nuclear type II J heat shock protein of the E. coli dnaJ family; contains a leucine zipper-like motif, binds to non-native substrates for presentation to Ssa3p, may function during protein translocation, assembly and disassembly	44885	1	3,1	0,1
23	YHR107C	Component of the septin ring that is required for cytokinesis; septins are GTP-binding proteins that assemble into rod-like hetero-oligomers that can associate with other rods to form filaments; septin rings at the mother-bud neck act as scaffolds f	46755	2	2,5	0,09
150	YGR263C	Sterol deacetylase; component of the sterol acetylation/deacetylation cycle along with Atf2p; active both in the endoplasmic reticulum (ER) and in lipid droplets; integral membrane protein with active site in the ER lumen; green fluorescent protein	49031	1	1,7	0,09
99	YPR108W	Essential non-ATPase regulatory subunit of the 26S proteasome; similar to another S. cerevisiae regulatory subunit, Rpn5p, as well as to mammalian proteasome subunits	49218	2	2,1	0,09
56	YOR274W	Delta 2-isopentenyl pyrophosphate:tRNA isopentenyl transferase; required for biosynthesis of isopentenyladenosine in mitochondrial and cytoplasmic tRNAs; also has a role in tRNA gene-mediated silencing; gene encodes two isozymic forms; converts to a	50611	2	1,6	0,09
59	YHR004C	Probable catalytic subunit of Nem1p-Spo7p phosphatase holoenzyme; regulates nuclear growth by controlling phospholipid biosynthesis, required for normal nuclear envelope morphology and sporulation; homolog of the human protein Dullard	50842	2	2	0,09
80	YHR146W	Protein that binds to cruciform DNA structures; CRP1 has a paralog, MDG1, that arose from the whole genome duplication	51201	2	1,7	0,09
90	YCL029C	Microtubule-associated protein; component of the interface between microtubules and kinetochore, involved in sister chromatid separation; essential in polyploid cells but not in haploid or diploid cells; ortholog of mammalian CLIP-170	51292	1	1,4	0,09
54	YBR029C	Phosphatidate cytidylyltransferase (CDP-diglyceride synthetase); an enzyme that catalyzes that conversion of CTP + phosphate into diphosphate + CDP-diaclglyerol, a critical step in the synthesis of all major yeast phospholipids	52485	2	1,5	0,08
144	YKL143W	Component of the GSE complex; GSE is required for proper sorting of amino acid permease Gap1p; required for ribosomal small subunit export from nucleus; required for growth at low temperature	53358	1	1,9	0,08
128	YHR036W	Essential nuclear envelope integral membrane protein; identified as a suppressor of a conditional mutation in the major karyopherin, CRM1; homologous to and interacts with Brr6p, a nuclear envelope protein involved in nuclear export	53700	1	4,5	0,08
64	YHR005C	Subunit of the G protein involved in pheromone response; GTP-binding alpha subunit of the heterotrimeric G protein; negatively regulates the mating pathway by sequestering G(beta)gamma and by triggering an adaptive response; activates Vps34p at the	54506	1	1,5	0,08
132	YJR121W	Beta subunit of the F1 sector of mitochondrial F1F0 ATP synthase; which is a large, evolutionarily conserved enzyme complex required for ATP synthesis; F1 translationally regulates ATP6 and ATP8 expression to achieve a balanced output of ATP synthas	54818	1	2,3	0,08
127	YDR473C	Splicing factor; component of the U4/U6-U5 snRNP complex	56125	1	1,7	0,08
2	YBL099W	Alpha subunit of the F1 sector of mitochondrial F1F0 ATP synthase; which is a large, evolutionarily conserved enzyme complex required for ATP synthesis; F1 translationally regulates ATP6 and ATP8 expression to achieve a balanced output of ATP syntha	58630	10	11	0,54
151	YIL104C	Chaperone protein; required for the assembly of box H/ACA snoRNPs and thus for pre-rRNA processing; functions as an RNA mimic; forms a complex with Naf1p and interacts with H/ACA snoRNP components Nhp2p and Cbf5p; homology with known Hsp90p cochaper	59288	1	2,6	0,07
137	YDR212W	Alpha subunit of chaperonin-containing T-complex; complex mediates protein folding in the cytosol; involved in actin cytoskeleton maintenance; overexpression in neurons suppresses formation of pathogenic conformations of huntingtin protein	60907	2	2	0,07
35	YMR145C	Mitochondrial external NADH dehydrogenase; type II NAD(P)H:quinone oxidoreductase that catalyzes the oxidation of cytosolic NADH; Nde1p and Nde2p provide cytosolic NADH to the mitochondrial respiratory chain; NDE1 has a paralog, NDE2, that arose fro	62851	1	2	0,07
85	YLR343W	1,3-beta-glucanosyltransferase; involved with Gas4p in spore wall assembly; has similarity to Gas1p	63250	1	1,1	0,07
110	YER158C	Protein of unknown function; potentially phosphorylated by Cdc28p; YER158C has a paralog, AFR1, that arose from the whole genome duplication	63904	1	1,4	0,07
41	YMR291W	Protein kinase of unknown cellular role; green fluorescent protein (GFP)-fusion protein localizes to the cytoplasm and nucleus; null mutant is sensitive to expression of the top1-T722A allele; not an essential gene; relocalizes from nucleus to cytop	66990	3	2,4	0,13

92	YLR248W	Protein kinase involved in response to oxidative and osmotic stress; identified as suppressor of S. pombe cell cycle checkpoint mutations; similar to CaM (calmodulin) kinases; RCK2 has a paralog, RCK1, that arose from the whole genome duplication	68309	2	2,1	0,06
147	YKL005C	Negative regulator of transcription elongation; contains a TFIIS-like domain that associates with chromatin and a PHD domain that interacts with H3K4me3; multicopy suppressor of temperature-sensitive ess1 mutations, binds RNA polymerase II large sub	68439	1	1,2	0,06
121	YKR084C	GTPase with similarity to translation release factors; together with binding partner Dom34p, facilitates ribosomal subunit dissociation and peptidyl-tRNA release when translation is stalled, particularly in 3~ UTRs; genetically implicated in mRNA no	68977	1	1,1	0,06
81	YMR198W	Kinesin-associated protein; required for both karyogamy and mitotic spindle organization, interacts stably and specifically with Kar3p and may function to target this kinesin to a specific cellular role; locus encodes a long and short transcript wit	69722	2	2,7	0,06
136	YDL224C	Putative RNA binding protein; regulates the cell size requirement for passage through Start and commitment to cell division; WHI4 has a paralog, WHI3, that arose from the whole genome duplication	70995	1	1,2	0,06
43	YMR106C	Subunit of the telomeric Ku complex (Yku70p-Yku80p); involved in telomere length maintenance, structure and telomere position effect; required for localization of telomerase ribonucleoprotein via interaction with the TLC1 guide RNA; relocates to sit	71834	1	1	0,06
96	YHR124W	Meiosis-specific transcription factor; required for exit from pachytene and for full meiotic recombination; activates middle sporulation genes; competes with Sum1p for binding to promoters containing middle sporulation elements (MSE)	71899	1	2,6	0,06
148	YGL110C	Protein of unknown function; has a CUE domain that binds ubiquitin, which may facilitate intramolecular monoubiquitination	72172	1	1,8	0,06
84	YER005W	Apyrase with wide substrate specificity; helps prevent inhibition of glycosylation by hydrolyzing nucleoside tri- and diphosphates that inhibit glycotransferases; partially redundant with Gda1p; mediates adenovirus E4orf4-induced toxicity	72445	1	1,3	0,06
73	YPL020C	Protease that specifically cleaves Smt3p protein conjugates; required for cell cycle progression; associates with nucleoporins and may interact with septin rings during telophase; sequestered to the nucleolus under stress conditions	72623	2	1,1	0,06
104	YLR413W	Putative protein of unknown function; not an essential gene; YLR413W has a paralog, FAT3, that arose from the whole genome duplication	73173	1	2,5	0,06
91	YKL079W	Kinesin-like myosin passenger-protein; interacts with Myo2p; controls actin cable structure and dynamics; proposed to be involved in exocytosis	74102	1	1,1	0,06
55	YJL034W	ATPase involved in protein import into the ER; also acts as a chaperone to mediate protein folding in the ER and may play a role in ER export of soluble proteins; regulates the unfolded protein response via interaction with Ire1p	74480	1	1,9	0,06
108	Q0120	Mitochondrial mRNA maturase; forms a complex with Nam2p to mediate splicing of the bI4 intron of the COB gene; encoded by both exon and intron sequences of partially processed COB mRNA	75053	1	1,3	0,06
82	YNR008W	Acyltransferase that catalyzes diacylglycerol esterification; one of several acyltransferases that contribute to triglyceride synthesis; Lro1p and Dga1p can O-acylate ceramides; putative homolog of human lecithin cholesterol acyltransferase	75635	2	1,4	0,06
67	YFR048W	Cytosolic protein required for sporulation	76099	1	1,4	0,06
61	YDR172W	Translation termination factor eRF3; has a role in mRNA deadenylation and decay; altered protein conformation creates the [PSI(+)] prion that modifies cellular fitness, alters translational fidelity by affecting reading frame selection, and results	76793	2	1,5	0,06
44	YKL179C	Golgi membrane protein with similarity to mammalian CASP; genetic interactions with GOS1 (encoding a Golgi snare protein) suggest a role in Golgi function	77525	4	2,8	0,18
134	YML016C	Serine/threonine protein phosphatase Z, isoform of Ppz2p; involved in regulation of potassium transport, which affects osmotic stability, cell cycle progression, and halotolerance	78139	1	1,4	0,06
77	YLR396C	ATP-binding protein that is a subunit of the HOPS and CORVET complexes; essential for protein sorting, vesicle docking, and fusion at the vacuole; binds to SNARE domains	79628	3	1,7	0,11
52	YHR031C	DNA helicase involved in rDNA replication and Ty1 transposition; binds to and suppresses DNA damage at G4 motifs in vivo; relieves replication fork pauses at telomeric regions; structurally and functionally related to Pif1p	81762	2	1,4	0,11
124	YGR186W	TFIIF (Transcription Factor II) largest subunit; involved in both transcription initiation and elongation of RNA polymerase II; homologous to human RAP74	82260	1	1,1	0,05
83	YDL138W	Plasma membrane high glucose sensor that regulates glucose transport; contains 12 predicted transmembrane segments and a long C-terminal tail required for induction of hexose transporters; RGT2 has a paralog, SNF3, that arose from the whole genome d	83629	2	1,2	0,05
70	YFL009W	F-box protein required for both the G1/S and G2/M phase transitions; modular substrate specificity factor which associates with core SCF (Cdc53p, Skp1p and Hrt1p/Rbx1p) to form the SCFCdc4 complex; SCFCdc4 acts as a ubiquitin-protein ligase directin	86501	2	1,4	0,05
141	YCL045C	Member of conserved endoplasmic reticulum membrane complex; involved in efficient folding of proteins in the ER; null mutant displays induction of the unfolded protein response; interacts with Gal80p; homologous to worm H17B01.4/EMC-1, fly CG2943, a	87475	1	1,3	0,05
129	YJR143C	Protein O-mannosyltransferase; transfers mannose residues from dolichyl phosphate-D-mannose to protein serine/threonine residues; appears to form homodimers in vivo and does not complex with other Pmt proteins; target for new antifungals	88548	1	1	0,05
122	YER001W	Alpha-1,3-mannosyltransferase; integral membrane glycoprotein of the Golgi complex, required for addition of alpha1,3-mannose linkages to N-linked and O-linked oligosaccharides, one of five S. cerevisiae proteins of the MNN1 family	89054	1	0,8	0,05
140	YHR182W	Protein of unknown function; green fluorescent protein (GFP)-fusion protein localizes to the cell periphery and cytoplasm; relocalizes from bud neck to cytoplasm upon DNA replication stress	90865	1	1,3	0,05
111	YGL229C	Protein required for function of the Sit4p protein phosphatase; member of a family of similar proteins that form complexes with Sit4p, including Sap155p, Sap185p, and Sap190p; SAP4 has a paralog, SAP155, that arose from the whole genome duplication	95191	1	1,2	0,05
102	Q0050	Reverse transcriptase required for splicing of the COX1 pre-mRNA; encoded by a mobile group II intron within the mitochondrial COX1 gene	96770	2	0,8	0,04
106	YER151C	Ubiquitin-specific protease involved in transport and osmotic response; interacts with Bre5p to co-regulate anterograde and retrograde transport between the ER and Golgi; involved in transcription elongation in response to osmostress through phospho	102202	2	0,8	0,04

125	YKR064W	Putative transcriptional repressor with Zn(2)-Cys(6) finger; negatively regulates transcription in response to oleate levels, based on mutant phenotype and localization to oleate-responsive promoters; the authentic, non-tagged protein is detected in	103035	1	0,7	0,04
101	YMR066W	Mitochondrial protein of unknown function	105204	1	2,2	0,04
63	YML111W	Component of the Rsp5p E3-ubiquitin ligase complex; involved in intracellular amino acid permease sorting, functions in heat shock element mediated gene expression, essential for growth in stress conditions; BUL2 has a paralog, BUL1, that arose from	105533	1	0,9	0,04
66	YBL106C	Protein with roles in exocytosis and cation homeostasis; functions in docking and fusion of post-Golgi vesicles with plasma membrane; regulates cell proliferation and colony development via the Rho1-Tor1 pathway; interacts with SNARE protein Sec9p;	112365	2	0,7	0,04
95	YDR430C	Lysine-specific metalloprotease of the pitrilysin family; metalloprotease of the intermembrane space; degrades proteins and presequence peptides cleaved from imported proteins; required for normal mitochondrial morphology	112458	3	0,9	0,04
149	YDR359C	Component of the NuA4 histone acetyltransferase complex; acts as a platform for assembly of NuA4 subunits into the native complex; required for initiation of pre-meiotic DNA replication, likely due to its requirement for expression of IME1	112839	1	0,9	0,04
145	YDL223C	Shmoo tip protein, substrate of Hub1p ubiquitin-like protein; mutants are defective for mating projection formation, thereby implicating Hbt1p in polarized cell morphogenesis; HBT1 has a paralog, YNL195C, that arose from the whole genome duplication	113548	1	1,1	0,04
138	YEL061C	Kinesin motor protein; involved in mitotic spindle assembly and chromosome segregation	113762	1	1,6	0,04
126	YDR325W	Subunit of the condensin complex; required for establishment and maintenance of chromosome condensation, chromosome segregation and chromatin binding of the condensin complex; required for clustering of tRNA genes at the nucleolus; required for repl	118648	1	1,4	0,04
112	YLL016W	Non-essential Ras guanine nucleotide exchange factor (GEF); localized to the membrane; expressed in poor nutrient conditions and on nonfermentable carbon sources; contains a stop codon in S288C, full-length gene includes YLL017W; SDC25 has a paralog	122581	1	1	0,04
115	YBR059C	Ser-Thr protein kinase; member (with Ark1p and Prk1p) of the Ark kinase family; involved in endocytosis and actin cytoskeleton organization	124261	1	0,9	0,03
60	YDL102W	Catalytic subunit of DNA polymerase delta; required for chromosomal DNA replication during mitosis and meiosis, intragenic recombination, repair of double strand DNA breaks, and DNA replication during nucleotide excision repair (NER)	125700	1	0,5	0,03
57	YLR096W	Serine/threonine protein kinase involved in regulation of exocytosis; localizes to the cytoplasmic face of the plasma membrane; KIN2 has a paralog, KIN1, that arose from the whole genome duplication	128607	1	0,6	0,03
89	YPR010C	RNA polymerase I second largest subunit A135	136700	1	1,1	0,03
98	YOL138C	Subunit of the SEA (Seh1-associated) complex; SEA is a coatomer-related complex that associates dynamically with the vacuole; null mutation suppresses cdc13-1 temperature sensitivity; has N-terminal WD-40 repeats and a C-terminal RING motif	150585	1	0,6	0,03
130	YBL047C	Scaffold protein involved in the formation of early endocytic sites; putative regulator of cytokinesis; homo-oligomerization is required for localization to and organization of endocytic sites; has a network of interactions with other endocytic prot	150866	1	0,9	0,03
87	YNL250W	Subunit of MRX complex with Mre11p and Xrs2p; complex is involved in processing double-strand DNA breaks in vegetative cells, initiation of meiotic DSBs, telomere maintenance, and nonhomologous end joining; forms nuclear foci upon DNA replication st	152997	3	1,3	0,06
133	YLR384C	Subunit of Elongator complex; Elongator is required for modification of wobble nucleosides in tRNA; maintains structural integrity of Elongator; homolog of human IKAP, mutations in which cause familial dysautonomia (FD)	153707	1	0,7	0,03
72	YIL126W	ATPase component of the RSC chromatin remodeling complex; required for expression of early meiotic genes; promotes base excision repair in chromatin; essential helicase-related protein homologous to Snf2p	156994	1	0,4	0,03
143	YML072C	Cortical ER protein involved in ER-plasma membrane tethering; one of 6 proteins (Ist2p, Scs2p, Scs22p, Tcb1p, Tcb2p, Tcb3p) that connect ER to the plasma membrane (PM) and regulate PM phosphatidylinositol-4-phosphate (PI4P) levels by controlling acc	171434	1	0,5	0,03
131	YOR326W	Type V myosin motor involved in actin-based transport of cargos; required for the polarized delivery of secretory vesicles, the vacuole, late Golgi elements, peroxisomes, and the mitotic spindle; MYO2 has a paralog, MYO4, that arose from the whole g	181380	2	0,6	0,02
139	YNL139C	Subunit of the THO complex; THO is required for efficient transcription elongation and involved in transcriptional elongation-associated recombination; required for LacZ RNA expression from certain plasmids	184686	1	0,4	0,02
103	YGL206C	Clathrin heavy chain; subunit of the major coat protein involved in intracellular protein transport and endocytosis; the clathrin triskelion is a trimeric molecule composed of three heavy chains that radiate from a vertex and three light chains whic	187929	1	0,5	0,02
146	YMR229C	RNA binding protein involved in synthesis of both 18S and 5.8S rRNAs; component of both the ribosomal small subunit (SSU) processosome and the 90S preribosome; acts as part of a Mak21p-Noc2p-Rrp5p module that associates with nascent pre-rRNA during	193712	1	1,2	0,02
76	YKR031C	Phospholipase D; catalyzes the hydrolysis of phosphatidylcholine, producing choline and phosphatidic acid; involved in Sec14p-independent secretion; required for meiosis and spore formation; differently regulated in secretion and meiosis; participat	196011	2	0,4	0,02
109	YER132C	Protein with an N-terminal kelch-like domain; putative negative regulator of early meiotic gene expression; required, with Mds3p, for growth under alkaline conditions; PMD1 has a paralog, MDS3, that arose from the whole genome duplication	196133	1	0,9	0,02
71	YLR422W	Protein of unknown function with similarity to human DOCK proteins; interacts with Ino4p; green fluorescent protein (GFP)-fusion protein localizes to the cytoplasm, YLR422W is not an essential protein; DOCK proteins act as guanine nucleotide exchang	223220	4	0,8	0,04

152	YPR117W	Putative protein of unknown function	286768	1	0,5	0,01
118	YLR087C	Protein required for fermentation at low temperature; plays a role in the maturation of secretory proteins; the authentic, non-tagged protein is detected in highly purified mitochondria in high-throughput studies	339784	1	0,4	0,01

BAND 11

prot_hit_num	prot_acc	prot_desc	prot_mass	prot_matches	prot_cover	emPAI
101	YPL152W-A	Protein of unknown function; identified by gene-trapping, microarray-based expression analysis, and genome-wide homology searching	3893	1	21,9	1,7
8	YPL271W	Epsilon subunit of the F1 sector of mitochondrial F1F0 ATP synthase; which is a large, evolutionarily conserved enzyme complex required for ATP synthesis; F1 translationally regulates ATP6 and ATP8 expression to achieve a balanced output of ATP synt	6738	1	17,7	0,81
2	YHR001W-A	Subunit of the ubiqunol-cytochrome c oxidoreductase complex; this complex comprises part of the mitochondrial respiratory chain; members include Cobp, Rip1p, Cyt1p, Cor1p, Qcr2p, Qcr6p, Qcr7p, Qcr8p, Qcr9p, and Qcr10p and comprises part of the mitoc	8587	12	44,2	5,68
9	YDR379C-A	Mitochondrial protein involved in assembly of succinate dehydrogenase; has a role in maturation of the Sdh2p subunit; member of the LYR protein family; mutations in human ortholog SDHAF1 are associated with infantile leukoencephalopathy	9346	1	20,3	0,55
12	YMR286W	Mitochondrial ribosomal protein of the large subunit	9525	1	16,3	0,53
6	YJL062W-A	Mitochondrial protein required for cytochrome c oxidase assembly; also involved in translational regulation of Cox1p and prevention of Cox1p aggregation before assembly; located in the mitochondrial inner membrane	9875	4	34,1	1,29
10	YEL020W-A	Essential protein of the mitochondrial intermembrane space; forms a complex with Tim10p (TIM10 complex) that delivers hydrophobic proteins to the TIM22 complex for insertion into the inner membrane	10427	1	9,2	0,48
1	YDR322C-A	Subunit e of mitochondrial F1F0-ATPase; ATPase is a large, evolutionarily conserved enzyme complex required for ATP synthesis; essential for the dimeric and oligomeric state of ATP synthase, which in turn determines the shape of inner membrane crist	10927	21	56,2	5,58
7	YKR094C	Ubiquitin-ribosomal 60S subunit protein L40B fusion protein; cleaved to yield ubiquitin and ribosomal protein L40B; ubiquitin may facilitate assembly of the ribosomal protein into ribosomes; homologous to mammalian ribosomal protein L40, no bacteria	14835	2	11,7	0,74
11	YBR010W	Histone H3; core histone protein required for chromatin assembly, part of heterochromatin-mediated telomeric and HM silencing; one of two identical histone H3 proteins (see HHT2); regulated by acetylation, methylation, and phosphorylation; H3K14 ace	15347	1	5,1	0,31
88	YDL094C	Dubious open reading frame; unlikely to encode a functional protein, based on available experimental and comparative sequence data; partially overlaps verfied gene PMT5/YDL093W; YDL094C is not essential	19854	1	5,9	0,23
85	YMR255W	Coiled-coiled protein of unknown function; identified as a high-copy suppressor of a dbp5 mutation; protein abundance increases in response to DNA replication stress	21558	2	3,7	0,21
15	YFL005W	Rab family GTPase; essential for vesicle-mediated exocytic secretion and autophagy; associates with the exocyst component Sec15p and may regulate polarized delivery of transport vesicles to the exocyst at the plasma membrane	23665	1	5,1	0,19
113	YML095C	Single-stranded DNA endonuclease (with Rad1p); cleaves single-stranded DNA during nucleotide excision repair and double-strand break repair; subunit of Nucleotide Excision Repair Factor 1 (NEF1); homolog of human ERCC1 protein	24354	1	3,3	0,19
27	YKL119C	Integral membrane protein required for V-ATPase function; not an actual component of the vacuolar H+-ATPase (V-ATPase) complex; functions in the assembly of the V-ATPase; localized to the endoplasmic reticulum (ER); involved in methionine restrictio	25387	2	4,2	0,18
79	YPL199C	Putative protein of unknown function; predicted to be palmitoylated	26915	1	6,7	0,17
24	YKL009W	Protein involved in mRNA turnover and ribosome assembly; required at post-transcriptional step for efficient retrotransposition; localizes to the nucleolus	27157	2	3,4	0,17
56	YGR271C-A	Essential protein required for maturation of 18S rRNA; null mutant is sensitive to hydroxyurea and is delayed in recovering from alpha-factor arrest; green fluorescent protein (GFP)-fusion protein localizes to the nucleolus	27163	1	4,3	0,17
58	YPL213W	Component of U2 snRNP complex; disruption causes reduced U2 snRNP levels; physically interacts with Msl1p; putative homolog of human U2A~ snRNP protein	27292	1	7,6	0,17
100	YER188W	Dubious open reading frame; unlikely to encode a functional protein, based on available experimental and comparative sequence data; large-scale analyses show mRNA expression increases under anaerobic conditions and two-hybrid interactions with Sst2p	27816	1	5	0,16
78	YMR157C	Protein of unknown function; null mutant displays reduced respiratory growth and elevated frequency of mitochondrial genome loss; the authentic, non-tagged protein is detected in purified mitochondria in high-throughput studies	29348	1	5,1	0,15
5	YDR099W	14-3-3 protein, minor isoform; controls proteome at post-transcriptional level, binds proteins and DNA, involved in regulation of many processes including exocytosis, vesicle transport, Ras/MAPK signaling, and rapamycin-sensitive signaling; protein	31100	3	6,6	0,31

59	YNR068C	Putative protein of unknown function; exhibits homology to C-terminal end of Bul1p; expressed as a readthrough product of BSC5, the readthrough locus being termed BUL3; the BUL3 readthrough product is involved in ubiquitin-mediated sorting of plasma	31489	1	5,9	0,14
61	YOR028C	Basic leucine zipper (bZIP) transcription factor of the yAP-1 family; physically interacts with the Tup1-Cyc8 complex and recruits Tup1p to its targets; mediates pleiotropic drug resistance and salt tolerance; nuclearly localized under oxidative str	33071	1	3,4	0,14
63	YBL030C	Major ADP/ATP carrier of the mitochondrial inner membrane; exchanges cytosolic ADP for mitochondrially synthesized ATP; also imports heme and ATP; phosphorylated; required for viability in many lab strains that carry a sal1 mutation; PET9 has a para	34636	1	3,1	0,13
105	YNR028W	Peptidyl-prolyl cis-trans isomerase (cyclophilin); catalyzes the cis-trans isomerization of peptide bonds N-terminal to proline residues; potential role in the secretory pathway; CPR8 has a paralog, CPR4, that arose from the whole genome duplication	35040	1	3,6	0,13
68	YJL052W	Glyceraldehyde-3-phosphate dehydrogenase (GAPDH), isozyme 1; involved in glycolysis and gluconeogenesis; tetramer that catalyzes the reaction of glyceraldehyde-3-phosphate to 1,3 bis-phosphoglycerate; detected in the cytoplasm and cell wall; protein	35844	1	2,7	0,12
94	YJL088W	Ornithine carbamoyltransferase; also known as carbamoylphosphate:L-ornithine carbamoyltransferase; catalyzes the biosynthesis of the arginine precursor citrulline	38227	1	5,9	0,12
35	YIL019W	Protein required for pre-rRNA processing; also required for 40S ribosomal subunit assembly	38866	1	2,6	0,11
45	YPR057W	snRNP protein component of spliceosomal snRNPs; required for pre-mRNA splicing and snRNP biogenesis; in null mutant newly-synthesized snRNAs are destabilized and 3~-end processing is slowed	39951	2	2,3	0,11
95	YER127W	Essential protein involved in maturation of 18S rRNA; depletion leads to inhibited pre-rRNA processing and reduced polysome levels; localizes primarily to the nucleolus	40827	1	2	0,11
43	YBR128C	Autophagy-specific subunit of phosphatidylinositol 3-kinase complex I; Atg14p targets complex I to the phagophore assembly site (PAS); required for localizing additional ATG proteins to the PAS; required for overflow degradation of misfolded protein	40949	1	2	0,11
66	YGL148W	Bifunctional chorismate synthase and flavin reductase; catalyzes the conversion of 5-enolpyruvylshikimate 3-phosphate (EPSP) to form chorismate, which is a precursor to aromatic amino acids; protein abundance increases in response to DNA replication	41103	3	2,9	0,23
18	YNL134C	Protein of unknown function; similar to dehydrogenases from other model organisms; green fluorescent protein (GFP)-fusion protein localizes to both the cytoplasm and nucleus; protein abundance increases in response to DNA replication stress	41371	2	3,2	0,11
82	YBR065C	Pre-mRNA splicing factor; facilitates the cooperative formation of U2/U6 helix II in association with stem II in the spliceosome, function may be regulated by Slu7p	41479	1	2,7	0,11
3	YFL039C	Actin; structural protein involved in cell polarization, endocytosis, and other cytoskeletal functions	41895	5	9,1	0,35
39	Q0075	Protein of unknown function; encoded within an intron of the mitochondrial COX1 gene; translational initiation codon is predicted to be ATA rather than ATG	42368	2	2,5	0,1
67	YPL138C	Subunit of COMPASS (Set1C); a complex which methylates histone H3 on lysine 4 and is required in telomeric transcriptional silencing; interacts with Orc2p; PHD finger domain protein similar to human CGBP, an unmethylated CpG binding protein; relocal	42486	2	5,4	0,1
28	YPL014W	Putative protein of unknown function; green fluorescent protein (GFP)-fusion protein localizes to the cytoplasm and to the nucleus	42683	2	3,9	0,1
102	YDR161W	Putative protein of unknown function; non-essential gene; proposed function in rRNA and ribosome biosynthesis based on transcriptional co-regulation; genetic interactions suggest a role in ER-associated protein degradation (ERAD)	43148	2	3,4	0,1
98	YOL043C	DNA N-glycosylase and apurinic/apyrimidinic (AP) lyase; involved in base excision repair, localizes to the nucleus; sumoylated; NTG2 has a paralog, NTG1, that arose from the whole genome duplication	44512	1	1,6	0,1
26	YIL136W	Mitochondrial outer membrane protein of unknown function; major constituent of the outer membrane, located on the outer (cytosolic) face; interacts with porin (Por1p) and with Om14p; imported via the presequence pathway involving the TOM and TIM23 c	44553	1	2,3	0,1
73	YLR082C	Protein of unknown function; overexpression suppresses the lethality caused by a rad53 null mutation	45286	1	2,3	0,1
4	YPR080W	Translational elongation factor EF-1 alpha; also encoded by TEF2; functions in the binding reaction of aminoacyl-tRNA (AA-tRNA) to ribosomes; may also have a role in tRNA re-export from the nucleus; TEF1 has a paralog, TEF2, that arose from the whol	50407	2	2,4	0,09
107	YLR080W	Integral membrane component of ER-derived COPII-coated vesicles; functions in ER to Golgi transport; EMP46 has a paralog, EMP47, that arose from the whole genome duplication	51088	2	2	0,09
37	YGR223C	Phosphatidylinositol 3,5-bisphosphate-binding protein; plays a role in micronucleophagy; belongs to the PROPPIN family of proteins; predicted to fold as a seven-bladed beta-propeller; displays punctate cytoplasmic localization	51667	2	1,3	0,09
96	YOL041C	Nucleolar protein involved in pre-25S rRNA processing; also involved in biogenesis of large 60S ribosomal subunit; contains an RNA recognition motif (RRM); binds to Ebp2; similar to Nop13p and Nsr1p	52142	1	2,4	0,08
42	YPL224C	Putative metal transporter involved in mitochondrial iron accumulation; MMT2 has a paralog, MMT1, that arose from the whole genome duplication	52518	2	3,3	0,17
57	YNR001C	Citrate synthase; catalyzes the condensation of acetyl coenzyme A and oxaloacetate to form citrate; the rate-limiting enzyme of the TCA cycle; nuclear encoded mitochondrial protein; CIT1 has a paralog, CIT2, that arose from the whole genome duplicat	53385	2	2,3	0,08

91	YMR239C	Nuclear dsRNA-specific ribonuclease (RNase III); involved in rDNA transcription, rRNA processing and U2 snRNA 3~ end formation by cleavage of a stem-loop structure at the 3~ end of U2 snRNA; involved in polyadenylation-independent transcription term	54095	1	1,7	0,08
25	YHR005C	Subunit of the G protein involved in pheromone response; GTP-binding alpha subunit of the heterotrimeric G protein; negatively regulates the mating pathway by sequestering G(beta)gamma and by triggering an adaptive response; activates Vps34p at the	54506	1	1,5	0,08
104	YMR044W	Member of a complex (Isw1b) with Isw1p and Ioc2p; interacts directly with H3K36me3 nucleosomes through its PWWP domain to recruit the Isw1b complex to open reading frames in a Set2p-dependent manner; Isw1b exhibits nucleosome-stimulated ATPase activ	55682	1	2,7	0,08
29	YPR119W	B-type cyclin involved in cell cycle progression; activates Cdc28p to promote the transition from G2 to M phase; accumulates during G2 and M, then targeted via a destruction box motif for ubiquitin-mediated degradation by the proteasome; CLB2 has a	56502	1	1,2	0,08
81	YGL012W	C-24(28) sterol reductase; catalyzes the final step in ergosterol biosynthesis; mutants are viable, but lack ergosterol	56698	1	1,5	0,08
87	YMR065W	Protein required for nuclear membrane fusion during karyogamy; localizes to the membrane with a soluble portion in the endoplasmic reticulum lumen, may form a complex with Jem1p and Kar2p; similar to zebrafish Brambleberry protein; expression of the	58924	2	4,4	0,15
41	YLR286C	Endochitinase; required for cell separation after mitosis; transcriptional activation during the G1 phase of the cell cycle is mediated by transcription factor Ace2p	59674	1	2,1	0,07
99	YDR212W	Alpha subunit of chaperonin-containing T-complex; complex mediates protein folding in the cytosol; involved in actin cytoskeleton maintenance; overexpression in neurons suppresses formation of pathogenic conformations of huntingtin protein	60907	1	2	0,07
46	Q0065	Endonuclease I-SceII; encoded by a mobile group I intron within the mitochondrial COX1 gene; intron is normally spliced by the BI4p maturase but AI4p can mutate to acquire the same maturase activity	63444	1	2,3	0,07
116	YDR062W	Component of serine palmitoyltransferase; responsible along with Lcb1p for the first committed step in sphingolipid synthesis, which is the condensation of serine with palmitoyl-CoA to form 3-ketosphinganine	63882	1	1,1	0,07
33	YCR088W	Actin-binding protein of the cortical actin cytoskeleton; important for activation of the Arp2/3 complex that plays a key role actin in cytoskeleton organization; inhibits barbed-end actin filament elongation; phosphorylation within its Proline-Rich	65594	2	1,7	0,07
60	YOR073W	Component of the spindle checkpoint; involved in sensing lack of tension on mitotic chromosomes; protects centromeric Rec8p at meiosis I; required for accurate chromosomal segregation at meiosis II and for mitotic chromosome stability; recruits cond	66724	1	1,5	0,07
62	YMR227C	TFIID subunit (67 kDa); involved in RNA polymerase II transcription initiation	67572	1	3,2	0,06
23	YNL225C	Component of the spindle pole body outer plaque; required for spindle orientation and mitotic nuclear migration; CNM67 has a paralog, ADY3, that arose from the whole genome duplication	67764	2	2,1	0,06
50	YLR248W	Protein kinase involved in response to oxidative and osmotic stress; identified as suppressor of S. pombe cell cycle checkpoint mutations; similar to CaM (calmodulin) kinases; RCK2 has a paralog, RCK1, that arose from the whole genome duplication	68309	2	2,1	0,06
84	YKR084C	GTPase with similarity to translation release factors; together with binding partner Dom34p, facilitates ribosomal subunit dissociation and peptidyl-tRNA release when translation is stalled, particularly in 3~ UTRs; genetically implicated in mRNA no	68977	2	1,1	0,06
74	YPR155C	Protein that regulates expression of Fo-F1 ATP synthase subunits; involved in the regulation of mitochondrial expression of subunits 6 (Atp6p) and 8 (Atp8p) of the Fo-F1 ATP synthase; functions with Nca3p	71281	1	2,8	0,06
13	YMR106C	Subunit of the telomeric Ku complex (Yku70p-Yku80p); involved in telomere length maintenance, structure and telomere position effect; required for localization of telomerase ribonucleoprotein via interaction with the TLC1 guide RNA; relocates to sit	71834	1	1	0,06
30	YER005W	Apyrase with wide substrate specificity; helps prevent inhibition of glycosylation by hydrolyzing nucleoside tri- and diphosphates that inhibit glycotransferases; partially redundant with Gda1p; mediates adenovirus E4orf4-induced toxicity	72445	2	1,3	0,06
53	YLR413W	Putative protein of unknown function; not an essential gene; YLR413W has a paralog, FAT3, that arose from the whole genome duplication	73173	2	2,5	0,06
83	YHR091C	Mitochondrial arginyl-tRNA synthetase; mutations in human ortholog are associated with pontocerebellar hypoplasia type 6; MSR1 has a paralog, YDR341C, that arose from the whole genome duplication	73995	1	1,2	0,06
115	YNR008W	Acyltransferase that catalyzes diacylglycerol esterification; one of several acyltransferases that contribute to triglyceride synthesis; Lro1p and Dga1p can O-acylate ceramides; putative homolog of human lecithin cholesterol acyltransferase	75635	1	1,4	0,06
32	YDR172W	Translation termination factor eRF3; has a role in mRNA deadenylation and decay; altered protein conformation creates the [PSI(+)] prion that modifies cellular fitness, alters translational fidelity by affecting reading frame selection, and results	76793	2	1,5	0,06
20	YKL179C	Golgi membrane protein with similarity to mammalian CASP; genetic interactions with GOS1 (encoding a Golgi snare protein) suggest a role in Golgi function	77525	4	1,2	0,12
51	YMR186W	Cytoplasmic chaperone of the Hsp90 family; plays a role in determining prion variants; redundant in function and nearly identical with Hsp82p, and together they are essential; expressed constitutively at 10-fold higher basal levels than HSP82 and in	80850	1	1,7	0,05
97	YOR022C	Putative carboxylic ester hydrolase; similar to bovine phospholipase A1; the authentic, non-tagged protein is detected in highly purified mitochondria in high-throughput studies	82009	1	1,1	0,05
44	YGR186W	TFIIF (Transcription Factor II) largest subunit; involved in both transcription initiation and elongation of RNA polymerase II; homologous to human RAP74	82260	1	1,1	0,05
118	YKL168C	Putative serine/threonine protein kinase with unknown cellular role; KKQ8 has a paralog, HAL5, that arose from the whole genome duplication	83466	1	1	0,05

54	YFL009W	F-box protein required for both the G1/S and G2/M phase transitions; modular substrate specificity factor which associates with core SCF (Cdc53p, Skp1p and Hrt1p/Rbx1p) to form the SCFCdc4 complex; SCFCdc4 acts as a ubiquitin-protein ligase directin	86501	1	1,4	0,05
110	YHR205W	AGC family protein kinase; functional ortholog of mammalian S6 kinase; phosphorylated by Tor1p and required for TORC1-mediated regulation of ribosome biogenesis, translation initiation, and entry into G0 phase; involved in transactivation of osmostr	92276	1	1,1	0,05
38	YDR251W	Essential protein of unknown function; exhibits variable expression during colony morphogenesis; overexpression permits survival without protein phosphatase 2A, inhibits growth, and induces a filamentous phenotype; PAM1 has a paralog, SVL3, that aro	93105	1	1,7	0,05
17	YMR089C	Mitochondrial inner membrane m-AAA protease component; mediates degradation of misfolded or unassembled proteins; also required for correct assembly of mitochondrial enzyme complexes	93508	2	0,7	0,05
55	YMR165C	Mg2+-dependent phosphatidate (PA) phosphatase; dephosphorylates PA to yield diacylglycerol; responsible for de novo lipid synthesis and formation of lipid droplets; phosphorylation by Pho80p-Pho85p decreases catalytic activity and alters Pah1p local	95147	1	0,8	0,05
86	YAL041W	Guanine nucleotide exchange factor for Cdc42p; also known as a GEF or GDP-release factor; required for polarity establishment and maintenance, and mutants have morphological defects in bud formation and shmooing; relocalizes from nucleus to cytoplas	97285	1	0,8	0,04
103	YOR211C	Mitochondrial GTPase, present in complex with Ugo1p and Fzo1p; required for mitochondrial morphology, fusion, and genome maintenance; exists as long and short form with different distributions; ratio of long to short forms is regulated by Psd1p; hom	99522	2	0,9	0,04
69	YPL147W	Subunit of a heterodimeric peroxisomal ABC transport complex; required for import of long-chain fatty acids into peroxisomes; similarity to human adrenoleukodystrophy transporter ABCD1 and ABCD2 and ALD-related proteins; mutations in ABCD1 cause X-l	100310	1	1	0,04
111	YLR386W	Enzyme regulator; involved in synthesis of phosphatidylinositol 3,5-bisphosphate, in control of trafficking of some proteins to the vacuole lumen via the MVB, and in maintenance of vacuole size and acidity; binds negative (Fig4p) and positive (Fab1p	100464	1	1	0,04
77	YKR064W	Putative transcriptional repressor with Zn(2)-Cys(6) finger; negatively regulates transcription in response to oleate levels, based on mutant phenotype and localization to oleate-responsive promoters; the authentic, non-tagged protein is detected in	103035	1	0,7	0,04
64	YMR066W	Mitochondrial protein of unknown function	105204	1	2,2	0,04
47	YBR084W	Mitochondrial C1-tetrahydrofolate synthase; involved in interconversion between different oxidation states of tetrahydrofolate (THF); provides activities of formyl-THF synthetase, methenyl-THF cyclohydrolase, and methylene-THF dehydrogenase	106615	1	1,1	0,04
80	YGR240C	Alpha subunit of heterooctameric phosphofructokinase; involved in glycolysis, indispensable for anaerobic growth, activated by fructose-2,6-bisphosphate and AMP, mutation inhibits glucose induction of cell cycle-related genes	108599	1	1,1	0,04
70	YBL085W	Protein implicated in polar growth; functionally redundant with Boi2p; interacts with bud-emergence protein Bem1p; contains an SH3 (src homology 3) domain and a PH (pleckstrin homology) domain; relocalizes from bud neck to cytoplasm upon DNA replica	109461	1	1,1	0,04
89	YMR231W	Histone E3 ligase, component of CORVET membrane tethering complex; peripheral vacuolar membrane protein required for protein trafficking and vacuole biogenesis; interacts with Pep7p; involved in ubiquitylation and degradation of excess histones	118448	1	0,8	0,04
112	YIL128W	Component of cytosolic iron-sulfur protein assembly (CIA) machinery; acts at a late step of Fe-S cluster assembly; forms the CIA targeting complex with Cia1p and Cia2p that directs Fe-S cluster incorporation into a subset of proteins involved in met	118562	1	0,6	0,04
93	YKR086W	DEAH-box RNA helicase involved in second catalytic step of splicing and in exon ligation; exhibits ATP-dependent RNA unwinding activity; mediates the release of Yju2p and Cwc25p in the second step; in the absence of ATP, stabilizes the binding of Cw	122388	1	1,1	0,04
71	YGL013C	Transcription factor that regulates the pleiotropic drug response; zinc cluster protein that is a master regulator involved in recruiting other zinc cluster proteins to pleiotropic drug response elements (PDREs) to fine tune the regulation of multid	123109	1	0,9	0,04
92	YOL145C	Component of the Paf1p complex involved in transcription elongation; binds to and modulates the activity of RNA polymerases I and II; required for expression of a subset of genes, including cyclin genes; involved in SER3 repression by helping to mai	125038	1	0,8	0,03
19	YPR030W	Nuclear ubiquitin protein ligase binding protein; may regulate utilization of nonfermentable carbon sources and endocytosis of plasma membrane proteins; overproduction suppresses chs5 spa2 lethality at high temp; ubiquitinated by Rsp5p, deubiquitina	125410	2	0,7	0,03
40	YER013W	DEAH-box RNA-dependent ATPase/ATP-dependent RNA helicase; associates with lariat intermediates before the second catalytic step of splicing; mediates ATP-dependent mRNA release from the spliceosome and unwinds RNA duplexes; required for proofreading	130625	1	0,9	0,03
36	YAL001C	Subunit of RNA polymerase III transcription initiation factor complex; part of the TauB domain of TFIIIC that binds DNA at the BoxB promoter sites of tRNA and similar genes; cooperates with Tfc6p in DNA binding; largest of six subunits of the RNA po	132432	2	0,9	0,03

108	YLR032W	DNA helicase/Ubiquitin ligase; involved in error-free branch of DNA damage tolerance (DDT) pathway; proposed to promote replication fork regression during postreplication repair by template switching; stimulates synthesis of free and PCNA-bound poly	134846	1	0,6	0,03
14	YPR122W	Haploid specific endoprotease of a-factor mating pheromone; performs one of two N-terminal cleavages during maturation of a-factor mating pheromone; required for axial budding pattern of haploid cells	139172	2	0,6	0,03
72	YDR227W	SIR protein involved in assembly of silent chromatin domains; silent information regulator (SIR) along with SIR2 and SIR3; involved in assembly of silent chromatin domains at telomeres and the silent mating-type loci; potentially phosphorylated by C	152259	1	0,9	0,03
106	YML049C	Protein involved in pre-mRNA splicing; component of the pre-spliceosome; associates with U2 snRNA; involved in ER to Golgi transport	155021	1	0,7	0,03
22	YIL126W	ATPase component of the RSC chromatin remodeling complex; required for expression of early meiotic genes; promotes base excision repair in chromatin; essential helicase-related protein homologous to Snf2p	156994	1	0,4	0,03
117	YJR092W	Anillin-like protein involved in bud-site selection; required for the axial budding pattern; localizes with septins to the bud neck in mitosis and may constitute an axial landmark for the next round of budding; required for the formation and disasse	165082	1	0,4	0,03
48	YMR259C	Protein that interacts with Trm7p for 2~-O-methylation of C32 of substrate tRNAs; green fluorescent protein (GFP)-fusion protein localizes to the cytoplasm; non-essential gene; functionally complemented by human THADA	165271	1	0,5	0,03
109	YKL101W	Nim1p-related protein kinase; regulates the morphogenesis and septin checkpoints; associates with the assembled septin filament; required along with Hsl7p for bud neck recruitment, phosphorylation, and degradation of Swe1p	169909	1	0,5	0,03
34	YLL015W	ABC type transmembrane transporter of MRP/CFTR family; found in vacuolar membrane, involved in the transport of unconjugated bilirubin and in heavy metal detoxification via glutathione conjugates, along with Ycf1p	177748	2	1,2	0,05
76	YOR326W	Type V myosin motor involved in actin-based transport of cargos; required for the polarized delivery of secretory vesicles, the vacuole, late Golgi elements, peroxisomes, and the mitotic spindle; MYO2 has a paralog, MYO4, that arose from the whole g	181380	2	0,6	0,02
114	YDR420W	Mucin family member that functions as an osmosensor in the HOG pathway; functions in the Sho1p-mediated HOG pathway with Msb2p; proposed to be a negative regulator of filamentous growth; mutant displays defects in beta-1,3 glucan synthesis and bud s	189124	1	0,4	0,02
90	YMR162C	Trans-golgi network aminophospholipid translocase (flippase); type 4 P-type ATPase; involved in phospholipid translocation, contributing to the maintenance of membrane lipid asymmetry in post-Golgi secretory vesicles; role in protein trafficking bet	189418	1	0,5	0,02
49	YKR031C	Phospholipase D; catalyzes the hydrolysis of phosphatidylcholine, producing choline and phosphatidic acid; involved in Sec14p-independent secretion; required for meiosis and spore formation; differently regulated in secretion and meiosis; participat	196011	2	0,4	0,02
21	YPL082C	Essential protein involved in regulation of transcription; removes Spt15p (TBP) from DNA via its C-terminal ATPase activity; may have a role in ensuring that soluble TBP is available to bind TATA-less promoters; forms a complex with TBP that binds T	210772	1	0,4	0,02
75	YGR032W	Catalytic subunit of 1,3-beta-glucan synthase; involved in formation of the inner layer of the spore wall; activity positively regulated by Rho1p and negatively by Smk1p; GSC2 has a paralog, FKS1, that arose from the whole genome duplication	218706	1	0,4	0,02
52	YLR422W	Protein of unknown function with similarity to human DOCK proteins; interacts with Ino4p; green fluorescent protein (GFP)-fusion protein localizes to the cytoplasm, YLR422W is not an essential protein; DOCK proteins act as guanine nucleotide exchang	223220	2	0,4	0,02
65	YCR093W	Component of the CCR4-NOT1 core complex; this complex has multiple roles in regulating mRNA levels including regulation of transcription and destabilizing mRNAs by deadenylation; basal transcription factor	240882	1	0,4	0,02
16	YJR066W	PIK-related protein kinase and rapamycin target; subunit of TORC1, a complex that controls growth in response to nutrients by regulating translation, transcription, ribosome biogenesis, nutrient transport and autophagy; involved in meiosis; TOR1 has	282760	3	0,9	0,03
31	YLR106C	Huge dynein-related AAA-type ATPase (midasin); forms extended pre-60S particle with the Rix1 complex (Rix1p-Ipi1p-Ipi3p); acts in removal of ribosomal biogenesis factors at successive steps of pre-60S assembly and export from nucleus	561221	2	0,2	0,01

BAND 12

prot_hit_num	prot_acc	prot_desc	prot_mass	prot_matches	prot_cover	emPAI
13	YML129C	Mitochondrial cytochrome c oxidase (complex IV) assembly factor; also involved in translational regulation of Cox1p and prevention of Cox1p aggregation before assembly; associates with complex IV assembly intermediates and complex III/complex IV sup	7954	5	50	6,59
20	YDR086C	Subunit of the Sec61p translocation complex (Sec61p-Sss1p-Sbh1p); this complex forms a channel for passage of secretory proteins through the endoplasmic reticulum membrane, and of the Ssh1p complex (Ssh1p-Sbh2p-Sss1p); interacts with Ost4p and Wbp1p	8996	2	12,5	0,57
1	YOR020W-A	Putative protein of unknown function; conserved in A. gossypii; the authentic, non-tagged protein is detected in highly purified mitochondria in high-throughput studies	9612	14	45,6	7,29

36	YBR058C-A	Protein that stimulates the activity of serine palmitoyltransferase; involved in sphingolipid biosynthesis; Lcb1p and Lcb2p are the two components of serine palmitoyltransferase	9631	1	12,5	0,53
7	YLR038C	Subunit VIb of cytochrome c oxidase; cytochrome c oxidase is also known as respiratory Complex IV and is the terminal member of the mitochondrial inner membrane electron transport chain; required for assembly of cytochrome c oxidase but not required	10014	5	28,9	1,27
118	YNR034W-A	Putative protein of unknown function; expression is regulated by Msn2p/Msn4p; YNR034W-A has a paralog, YCR075W-A, that arose from the whole genome duplication	10775	1	8,2	0,46
35	YDL045W-A	Mitochondrial ribosomal protein of the small subunit; contains twin cysteine-x9-cysteine motifs; oxidized by Mia40p during import into mitochondria	10916	2	9,5	0,46
5	YDR322C-A	Subunit e of mitochondrial F1F0-ATPase; ATPase is a large, evolutionarily conserved enzyme complex required for ATP synthesis; essential for the dimeric and oligomeric state of ATP synthase, which in turn determines the shape of inner membrane crist	10927	10	56,2	8,62
27	YJR010C-A	Subunit of the signal peptidase complex (SPC); SPC cleaves the signal sequence from proteins targeted to the endoplasmic reticulum (ER); homolog of the SPC12 subunit of mammalian signal peptidase complex; protein abundance increases in response to D	10986	2	11,7	0,45
4	YER048W-A	Cysteine desulfurase (Nfs1p) activator; essential for the formation of the persulfide intermediate at the desulfurase active site during pyridoxal phosphate-dependent desulfuration of cysteine; required for mitochondrial iron-sulfur cluster biosynth	11259	17	67	38,04
2	YDR377W	Subunit f of the F0 sector of mitochondrial F1F0 ATP synthase; F1F0 ATP synthase is a large, evolutionarily conserved enzyme complex required for ATP synthesis	11305	28	29,7	3,27
50	YBR009C	Histone H4; core histone protein required for chromatin assembly and chromosome function; one of two identical histone proteins (see also HHF2); contributes to telomeric silencing; N-terminal domain involved in maintaining genomic integrity	11361	2	11,7	0,44
9	YGL030W	Ribosomal 60S subunit protein L30; involved in pre-rRNA processing in the nucleolus; autoregulates splicing of its transcript; homologous to mammalian ribosomal protein L30, no bacterial homolog	11408	3	27,6	1,05
53	YBR268W	Mitochondrial ribosomal protein of the large subunit	11999	1	9,5	0,41
6	YJR048W	Cytochrome c, isoform 1; also known as iso-1-cytochrome c; electron carrier of the mitochondrial intermembrane space that transfers electrons from ubiquinone-cytochrome c oxidoreductase to cytochrome c oxidase during cellular respiration; mutations	12348	10	46,8	6,44
14	YBR262C	Component of the MICOS complex; MICOS (formerly MINOS or MitOS) is a mitochondrial inner membrane complex that extends into the intermembrane space and has a role in the maintenance of crista junctions, inner membrane architecture, and formation of	12380	4	32,1	1,7
26	YAL044W-A	Putative protein of unknown function; similar to S. pombe uvi31 which is a putative DNA repair protein	12587	2	18,2	0,93
10	YMR230W	Protein component of the small (40S) ribosomal subunit; homologous to mammalian ribosomal protein S10, no bacterial homolog; RPS10B has a paralog, RPS10A, that arose from the whole genome duplication	12731	6	38,1	1,64
17	YPR020W	Subunit g of the mitochondrial F1F0 ATP synthase; reversibly phosphorylated on two residues; unphosphorylated form is required for dimerization of the ATP synthase complex, which in turn determines oligomerization of the complex and the shape of inn	12971	5	27	1,6
30	YOR327C	Vesicle membrane receptor protein (v-SNARE); involved in the fusion between Golgi-derived secretory vesicles with the plasma membrane; Snc2p levels regulated by Vps45p; member of the synaptobrevin/VAMP family of R-type v-SNARE proteins; SNC2 has a p	13007	1	10,4	0,37
46	YDR079W	Chaperone that facilitates the assembly of cytochrome c oxidase; integral to the mitochondrial inner membrane; interacts with a subcomplex of subunits VII, VIIa, and VIII (Cox7p, Cox9p, and Cox8p) but not with the holoenzyme	13237	1	6,3	0,37
45	YGR020C	Subunit F of the V1 peripheral membrane domain of V-ATPase; part of the electrogenic proton pump found throughout the endomembrane system; required for the V1 domain to assemble onto the vacuolar membrane; the V1 peripheral membrane domain of vacuol	13453	1	7,6	0,36
51	YDL157C	Putative protein of unknown function; the authentic, non-tagged protein is detected in highly purified mitochondria in high-throughput studies	13670	1	7,6	0,35
55	YBL002W	Histone H2B; core histone protein required for chromatin assembly and chromosome function; nearly identical to HTB1; Rad6p-Bre1p-Lge1p mediated ubiquitination regulates reassembly after DNA replication, transcriptional activation, meiotic DSB format	14229	1	6,9	0,34
19	YJL190C	Protein component of the small (40S) ribosomal subunit; homologous to mammalian ribosomal protein S15A and bacterial S8; RPS22A has a paralog, RPS22B, that arose from the whole genome duplication	14675	2	12,3	0,33
40	YKR094C	Ubiquitin-ribosomal 60S subunit protein L40B fusion protein; cleaved to yield ubiquitin and ribosomal protein L40B; ubiquitin may facilitate assembly of the ribosomal protein into ribosomes; homologous to mammalian ribosomal protein L40, no bacteria	14835	1	7	0,32
8	YGL191W	Subunit VIa of cytochrome c oxidase; present in a subclass of cytochrome c oxidase complexes that may have a role in mimimizing generation of reactive oxygen species; not essential for cytochrome c oxidase activity but may modulate activity in respo	15070	7	29,5	2
88	YNR022C	Mitochondrial ribosomal protein of the large subunit; not essential for mitochondrial translation	16389	1	10,8	0,29
15	YML026C	Protein component of the small (40S) ribosomal subunit; homologous to mammalian ribosomal protein S18 and bacterial S13; RPS18B has a paralog, RPS18A, that arose from the whole genome duplication; protein abundance increases in response to DNA repli	17085	4	29,5	1,65

24	YHR051W	Subunit VI of cytochrome c oxidase (Complex IV); Complex IV is the terminal member of the mitochondrial inner membrane electron transport chain; expression is regulated by oxygen levels	17389	2	14,9	0,61
34	YGL041W-A	Putative protein of unknown function; conserved in fungi; identified by expression profiling and mass spectrometry	18092	2	19,5	0,58
121	YLR170C	Small subunit of the clathrin-associated adaptor complex AP-1; AP-1 is involved in protein sorting at the trans-Golgi network; homolog of the sigma subunit of the mammalian clathrin AP-1 complex	18314	1	7,1	0,26
74	YGR076C	Mitochondrial ribosomal protein of the large subunit; mutation confers increased replicative lifespan	18575	1	8,9	0,25
62	YPR028W	Membrane protein that interacts with Yip1p to mediate membrane traffic; interacts with Sey1p to maintain ER morphology; overexpression leads to cell death and accumulation of internal cell membranes; mutants have reduced phosphatidylserine transfer	20257	1	4,4	0,23
52	YGR174C	Mitochondrial protein required for assembly of cytochrome bc1 complex; interacts with the Cbp3p-Cbp6p complex and newly synthesized cytochrome b (Cobp) to promote assembly of Cobp into the cytochrome bc1 complex	20265	1	5,9	0,23
95	YML011C	Protein involved in nucleotide excision repair; green fluorescent protein (GFP)-fusion protein localizes to the nucleus	20547	1	4,5	0,23
136	YMR255W	Coiled-coiled protein of unknown function; identified as a high-copy suppressor of a dbp5 mutation; protein abundance increases in response to DNA replication stress	21558	1	3,7	0,21
12	YCR003W	Mitochondrial ribosomal protein of the large subunit; protein abundance increases in response to DNA replication stress	21655	4	13,7	0,79
44	YDR298C	Subunit 5 of the stator stalk of mitochondrial F1F0 ATP synthase; F1F0 ATP synthase is a large, evolutionarily conserved enzyme complex required for ATP synthesis; homologous to bovine subunit OSCP (oligomycin sensitivity-conferring protein); phosph	22858	1	5,2	0,2
32	YDR041W	Mitochondrial ribosomal protein of the small subunit; has similarity to E. coli S10 ribosomal protein; essential for viability, unlike most other mitoribosomal proteins	23467	1	5,9	0,2
49	YER183C	5,10-methenyltetrahydrofolate synthetase; involved in folic acid biosynthesis	24333	1	10,4	0,19
22	YPL078C	Subunit b of the stator stalk of mitochondrial F1F0 ATP synthase; ATP synthase is a large, evolutionarily conserved enzyme complex required for ATP synthesis; contributes to the oligomerization of the complex, which in turn determines the shape of i	26966	4	13,1	0,59
21	YER120W	Integral ER membrane protein, regulates phospholipid metabolism; one of 6 proteins (Ist2p, Scs2p, Scs22p, Tcb1p, Tcb2p, Tcb3p) that connect ER to the plasma membrane (PM) and regulate PI4P levels by controlling access of Sac1p phosphatase to its sub	27025	2	5,7	0,17
43	YJL063C	Mitochondrial ribosomal protein of the large subunit	27044	1	3,4	0,17
71	YGR271C-A	Essential protein required for maturation of 18S rRNA; null mutant is sensitive to hydroxyurea and is delayed in recovering from alpha-factor arrest; green fluorescent protein (GFP)-fusion protein localizes to the nucleolus	27163	1	4,3	0,17
97	YMR157C	Protein of unknown function; null mutant displays reduced respiratory growth and elevated frequency of mitochondrial genome loss; the authentic, non-tagged protein is detected in purified mitochondria in high-throughput studies	29348	1	5,1	0,15
78	YOR145C	Essential nucleolar protein required for pre-18S rRNA processing; interacts with Dim1p, an 18S rRNA dimethyltransferase, and also with Nob1p, which is involved in proteasome biogenesis; contains a KH domain	30313	1	4	0,15
108	YPR183W	Dolichol phosphate mannose (Dol-P-Man) synthase of the ER membrane; catalyzes the formation of Dol-P-Man from Dol-P and GDP-Man; required for glycosyl phosphatidylinositol membrane anchoring, O mannosylation, and protein glycosylation	30517	1	4,9	0,15
129	YNL055C	Mitochondrial porin (voltage-dependent anion channel); outer membrane protein required for maintenance of mitochondrial osmotic stability and mitochondrial membrane permeability; couples the glutathione pools of the intermembrane space (IMS) and the	30526	1	3,5	0,15
75	YNR068C	Putative protein of unknown function; exhibits homology to C-terminal end of Bul1p; expressed as a readthrough product of BSC5, the readthrough locus being termed BUL3; the BUL3 readthrough product is involved in ubiquitin-mediated sorting of plasma	31489	2	5,9	0,14
57	YOR106W	Syntaxin-like vacuolar t-SNARE; functions with Vam7p in vacuolar protein trafficking; mediates docking/fusion of late transport intermediates with the vacuole; has an acidic di-leucine sorting signal and C-terminal transmembrane region	32710	1	8,8	0,14
11	YJR077C	Mitochondrial phosphate carrier; imports inorganic phosphate into mitochondria; functionally redundant with Pic2p but more abundant than Pic2p under normal conditions; phosphorylated	32965	3	10	0,29
124	YLR003C	Putative subunit of the 90S preribosome processome complex; overexpression rescues supressor mutant of mcm10; null mutant is viable; relocalizes from nucleus to cytoplasm upon DNA replication stress	33490	1	3,4	0,13
29	YLR340W	Conserved ribosomal protein P0 of the ribosomal stalk; involved in interaction between translational elongation factors and the ribosome; phosphorylated on serine 302; homologous to mammalian ribosomal protein LP0 and bacterial L10	33696	1	5,1	0,13
47	YML052W	Plasma membrane protein, component of eisosomes; long-lived protein that remains stable in eisosomes of mother cells while other eisosome proteins, Pil1p and Lsp1p, turn over; may function to anchor the eisosome in place; sporulation and plasma memb	34098	1	3,6	0,13
98	YKL107W	Putative short-chain dehydrogenase/reductase; proposed to be a palmitoylated membrane protein	34677	1	2,9	0,13
41	YOL086C	Alcohol dehydrogenase; fermentative isozyme active as homo- or heterotetramers; required for the reduction of acetaldehyde to ethanol, the last step in the glycolytic pathway; ADH1 has a paralog, ADH5, that arose from the whole genome duplication	37290	1	3,7	0,12
102	YBL033C	GTP cyclohydrolase II; catalyzes the first step of the riboflavin biosynthesis pathway	38714	2	2,6	0,11

28	YPR191W	Subunit 2 of ubiquinol cytochrome-c reductase (Complex III); Complex III is a component of the mitochondrial inner membrane electron transport chain; phosphorylated; transcription is regulated by Hap1p, Hap2p/Hap3p, and heme	40511	3	7,6	0,37
127	Q0075	Protein of unknown function; encoded within an intron of the mitochondrial COX1 gene; translational initiation codon is predicted to be ATA rather than ATG	42368	1	2,5	0,1
69	YPL014W	Putative protein of unknown function; green fluorescent protein (GFP)-fusion protein localizes to the cytoplasm and to the nucleus	42683	1	3,9	0,1
120	YKL219W	Protein of unknown function; member of the DUP380 subfamily of conserved, often subtelomerically-encoded proteins	49035	1	2,5	0,09
93	YPR108W	Essential non-ATPase regulatory subunit of the 26S proteasome; similar to another S. cerevisiae regulatory subunit, Rpn5p, as well as to mammalian proteasome subunits	49218	2	2,1	0,09
117	YDL001W	Cytoplasmic protein required for sporulation	50074	1	3	0,09
33	YPR080W	Translational elongation factor EF-1 alpha; also encoded by TEF2; functions in the binding reaction of aminoacyl-tRNA (AA-tRNA) to ribosomes; may also have a role in tRNA re-export from the nucleus; TEF1 has a paralog, TEF2, that arose from the whol	50407	1	2,6	0,09
76	YOR274W	Delta 2-isopentenyl pyrophosphate:tRNA isopentenyl transferase; required for biosynthesis of isopentenyladenosine in mitochondrial and cytoplasmic tRNAs; also has a role in tRNA gene-mediated silencing; gene encodes two isozymic forms; converts to a	50611	1	1,6	0,09
64	YHR004C	Probable catalytic subunit of Nem1p-Spo7p phosphatase holoenzyme; regulates nuclear growth by controlling phospholipid biosynthesis, required for normal nuclear envelope morphology and sporulation; homolog of the human protein Dullard	50842	2	2	0,09
60	YCL029C	Microtubule-associated protein; component of the interface between microtubules and kinetochore, involved in sister chromatid separation; essential in polyploid cells but not in haploid or diploid cells; ortholog of mammalian CLIP-170	51292	1	1,4	0,09
68	YLR431C	Peripheral membrane protein required for autophagy and CVT; required for cytoplasm-to-vacuole targeting (Cvt) pathway and efficient macroautophagy; cycles between the phagophore assembly site (PAS) and non-PAS locations; forms a complex with Atg9p a	51741	1	2,6	0,09
135	YAR071W	One of three repressible acid phosphatases; glycoprotein that is transported to the cell surface by the secretory pathway; induced by phosphate starvation and coordinately regulated by PHO4 and PHO2; PHO11 has a paralog, PHO12, that arose from a seg	53247	1	1,7	0,08
73	YLR359W	Adenylosuccinate lyase; catalyzes two steps in the ~de novo~ purine nucleotide biosynthetic pathway; expression is repressed by adenine and activated by Bas1p and Pho2p; mutations in human ortholog ADSL cause adenylosuccinase deficiency	54708	1	1,9	0,08
3	YJR121W	Beta subunit of the F1 sector of mitochondrial F1F0 ATP synthase; which is a large, evolutionarily conserved enzyme complex required for ATP synthesis; F1 translationally regulates ATP6 and ATP8 expression to achieve a balanced output of ATP synthas	54818	15	13,9	0,72
107	YMR177W	Putative metal transporter involved in mitochondrial iron accumulation; MMT1 has a paralog, MMT2, that arose from the whole genome duplication	56465	1	2	0,08
23	YBL099W	Alpha subunit of the F1 sector of mitochondrial F1F0 ATP synthase; which is a large, evolutionarily conserved enzyme complex required for ATP synthesis; F1 translationally regulates ATP6 and ATP8 expression to achieve a balanced output of ATP syntha	58630	4	3,3	0,16
115	YDR212W	Alpha subunit of chaperonin-containing T-complex; complex mediates protein folding in the cytosol; involved in actin cytoskeleton maintenance; overexpression in neurons suppresses formation of pathogenic conformations of huntingtin protein	60907	1	2	0,07
37	YHR007C	Lanosterol 14-alpha-demethylase; catalyzes the C-14 demethylation of lanosterol to form 4,4~~-dimethyl cholesta-8,14,24-triene-3-beta-ol in the ergosterol biosynthesis pathway; member of the cytochrome P450 family; associated and coordinately regula	60913	1	2,5	0,07
94	YKR016W	Component of the MICOS complex; MICOS (formerly MINOS or MitOS) is a mitochondrial inner membrane complex that extends into the intermembrane space and has a role in the maintenance of crista junctions, inner membrane architecture, and formation of	61625	1	2,8	0,07
86	YNL154C	Palmitoylated plasma membrane-bound casein kinase I (CK1) isoform; shares redundant functions with Yck1p in morphogenesis, proper septin assembly, endocytic trafficking, and glucose sensing; stabilized by Sod1p binding in the presence of glucose and	62215	1	1,5	0,07
25	YMR145C	Mitochondrial external NADH dehydrogenase; type II NAD(P)H:quinone oxidoreductase that catalyzes the oxidation of cytosolic NADH; Nde1p and Nde2p provide cytosolic NADH to the mitochondrial respiratory chain; NDE1 has a paralog, NDE2, that arose fro	62851	3	5,4	0,22
18	YER086W	Threonine deaminase, catalyzes first step in isoleucine biosynthesis; expression is under general amino acid control; ILV1 locus exhibits highly positioned nucleosomes whose organization is independent of known ILV1 regulation	64081	4	4	0,14
100	YOR027W	Hsp90 cochaperone; interacts with the Ssa group of the cytosolic Hsp70 chaperones and activates Ssa1p ATPase activity; interacts with Hsp90 chaperones and inhibits their ATPase activity; homolog of mammalian Hop	66398	1	1,9	0,07
126	YOR386W	DNA photolyase involved in photoreactivation; repairs pyrimidine dimers in the presence of visible light; induced by DNA damage; regulated by transcriptional repressor Rph1p	66753	1	1,9	0,07
122	YDR200C	Protein required for cytoplasm to vacuole targeting of proteins; forms a complex with Far3p and Far7p to Far11p involved in recovery from pheromone-induced cell cycle arrest; mutant has increased aneuploidy tolerance; VPS64 has a paralog, FAR10, tha	67416	1	1,7	0,06
96	YMR282C	Mitochondrial protein; likely involved in translation of the mitochondrial OLI1 mRNA; exhibits genetic interaction with the OLI1 mRNA 5~-untranslated leader	68119	1	2,8	0,06

83	YLR248W	Protein kinase involved in response to oxidative and osmotic stress; identified as suppressor of S. pombe cell cycle checkpoint mutations; similar to CaM (calmodulin) kinases; RCK2 has a paralog, RCK1, that arose from the whole genome duplication	68309	2	2,1	0,06
128	YKL005C	Negative regulator of transcription elongation; contains a TFIIS-like domain that associates with chromatin and a PHD domain that interacts with H3K4me3; multicopy suppressor of temperature-sensitive ess1 mutations, binds RNA polymerase II large sub	68439	1	1,2	0,06
72	YJR045C	Hsp70 family ATPase; constituent of the import motor component of the Translocase of the Inner Mitochondrial membrane (TIM23 complex); involved in protein translocation and folding; subunit of SceI endonuclease; SSC1 has a paralog, ECM10, that arose	70585	1	2,1	0,06
90	YDL224C	Putative RNA binding protein; regulates the cell size requirement for passage through Start and commitment to cell division; WHI4 has a paralog, WHI3, that arose from the whole genome duplication	70995	1	1,2	0,06
134	YJL085W	Subunit of the exocyst complex; the exocyst mediates polarized targeting and tethering of post-Golgi secretory vesicles to active sites of exocytosis prior to SNARE-mediated fusion; PtdIns[4,5]P2-binding protein that localizes to exocytic sites in a	71539	1	1,3	0,06
39	YMR106C	Subunit of the telomeric Ku complex (Yku70p-Yku80p); involved in telomere length maintenance, structure and telomere position effect; required for localization of telomerase ribonucleoprotein via interaction with the TLC1 guide RNA; relocates to sit	71834	2	1	0,06
111	YDR380W	Phenylpyruvate decarboxylase; catalyzes decarboxylation of phenylpyruvate to phenylacetaldehyde, which is the first specific step in the Ehrlich pathway; involved in protein N-terminal Met and Ala catabolism	72035	1	1,1	0,06
116	YGL110C	Protein of unknown function; has a CUE domain that binds ubiquitin, which may facilitate intramolecular monoubiquitination	72172	2	1,8	0,06
63	YER005W	Apyrase with wide substrate specificity; helps prevent inhibition of glycosylation by hydrolyzing nucleoside tri- and diphosphates that inhibit glycotransferases; partially redundant with Gda1p; mediates adenovirus E4orf4-induced toxicity	72445	2	1,3	0,06
31	YIL155C	Mitochondrial glycerol-3-phosphate dehydrogenase; expression is repressed by both glucose and cAMP and derepressed by non-fermentable carbon sources in a Snf1p, Rsf1p, Hap2/3/4/5 complex dependent manner	72807	2	1,2	0,06
101	Q0120	Mitochondrial mRNA maturase; forms a complex with Nam2p to mediate splicing of the bI4 intron of the COB gene; encoded by both exon and intron sequences of partially processed COB mRNA	75053	1	1,3	0,06
125	YNR008W	Acyltransferase that catalyzes diacylglycerol esterification; one of several acyltransferases that contribute to triglyceride synthesis; Lro1p and Dga1p can O-acylate ceramides; putative homolog of human lecithin cholesterol acyltransferase	75635	1	1,4	0,06
61	YFR048W	Cytosolic protein required for sporulation	76099	1	1,4	0,06
91	YDR107C	Protein with a role in cellular adhesion and filamentous growth; similar to Tmn3p; member of the evolutionarily conserved Transmembrane Nine family of proteins with nine membrane-spanning segments; TMN2 has a paralog, EMP70, that arose from the whol	76935	1	1,2	0,06
59	YKL179C	Golgi membrane protein with similarity to mammalian CASP; genetic interactions with GOS1 (encoding a Golgi snare protein) suggest a role in Golgi function	77525	3	2,5	0,12
105	YLR335W	Nucleoporin involved in nucleocytoplasmic transport; binds to either the nucleoplasmic or cytoplasmic faces of the nuclear pore complex depending on Ran-GTP levels; also has a role in chromatin organization	77892	1	0,8	0,06
16	YOR317W	Long chain fatty acyl-CoA synthetase; activates imported fatty acids with a preference for C12:0-C16:0 chain lengths; functions in long chain fatty acid import; accounts for most acyl-CoA synthetase activity; localized to lipid particles; involved i	78571	3	3	0,11
119	YLR035C	Protein involved in mismatch repair and meiotic recombination; only certain frameshift intermediates are mismatch repair substrates; forms a complex with Mlh1p	78721	1	1,9	0,06
38	YKR001C	Dynamin-like GTPase required for vacuolar sorting; also involved in actin cytoskeleton organization, endocytosis, late Golgi-retention of some proteins, regulation of peroxisome biogenesis	78804	1	1,1	0,06
65	YLR396C	ATP-binding protein that is a subunit of the HOPS and CORVET complexes; essential for protein sorting, vesicle docking, and fusion at the vacuole; binds to SNARE domains	79628	3	1,7	0,11
58	YHR031C	DNA helicase involved in rDNA replication and Ty1 transposition; binds to and suppresses DNA damage at G4 motifs in vivo; relieves replication fork pauses at telomeric regions; structurally and functionally related to Pif1p	81762	1	1,4	0,05
54	YPR024W	Catalytic subunit of the i-AAA protease complex; complex is located in the mitochondrial inner membrane; responsible for degradation of unfolded or misfolded mitochondrial gene products; serves as a nonconventional translocation motor to pull PNPase	82185	1	1,3	0,05
132	YPL158C	Protein that regulates Cdc42p and Rho1p; functions in the late steps of cytokinesis and cell separation; sustains Rho1p at the cell division site after actomyosin ring contraction; inhibits the activation of Cdc42-Cla4 at the cell division site to p	85026	1	0,8	0,05
66	YFL009W	F-box protein required for both the G1/S and G2/M phase transitions; modular substrate specificity factor which associates with core SCF (Cdc53p, Skp1p and Hrt1p/Rbx1p) to form the SCFCdc4 complex; SCFCdc4 acts as a ubiquitin-protein ligase directin	86501	2	1,4	0,05
99	YJL033W	DEAD box RNA helicase; component of the SSU; interacts with Bfr2p and Enp2p; high-copy number suppression of a U14 snoRNA processing mutant suggests an involvement in 18S rRNA synthesis	87428	1	1,2	0,05
114	YJR143C	Protein O-mannosyltransferase; transfers mannose residues from dolichyl phosphate-D-mannose to protein serine/threonine residues; appears to form homodimers in vivo and does not complex with other Pmt proteins; target for new antifungals	88548	1	1	0,05
77	YPL032C	Protein of unknown function; mutant phenotype suggests a potential role in vacuolar function; green fluorescent protein (GFP)-fusion protein localizes to the cell periphery, cytoplasm, bud, and bud neck; relocalizes from bud neck to cytoplasm upon D	92261	1	0,8	0,05
137	YHR205W	AGC family protein kinase; functional ortholog of mammalian S6 kinase; phosphorylated by Tor1p and required for TORC1-mediated regulation of ribosome biogenesis, translation initiation, and entry into G0 phase; involved in transactivation of osmostr	92276	1	1	0,05

85	YOR018W	Alpha-arrestin involved in ubiquitin-dependent endocytosis; activating dephosphorylation relays glucose signaling to transporter endocytosis; calcineurin dephosphorylation is required for Rsp5p-dependent internalization of agonist-occupied Ste2p, as	92583	1	1,2	0,05
70	YMR089C	Mitochondrial inner membrane m-AAA protease component; mediates degradation of misfolded or unassembled proteins; also required for correct assembly of mitochondrial enzyme complexes	93508	1	0,7	0,05
92	YHR103W	Protein involved in bud growth; involved in the transport of cell wall components from the Golgi to the cell surface; similar in structure and functionally redundant with Sbe2p; SBE22 has a paralog, SBE2, that arose from the whole genome duplication	96860	1	0,9	0,04
133	YOR211C	Mitochondrial GTPase, present in complex with Ugo1p and Fzo1p; required for mitochondrial morphology, fusion, and genome maintenance; exists as long and short form with different distributions; ratio of long to short forms is regulated by Psd1p; hom	99522	1	0,9	0,04
87	YPL147W	Subunit of a heterodimeric peroxisomal ABC transport complex; required for import of long-chain fatty acids into peroxisomes; similarity to human adrenoleukodystrophy transporter ABCD1 and ABCD2 and ALD-related proteins; mutations in ABCD1 cause X-l	100310	2	1,8	0,09
89	YLR045C	Microtubule-associated protein (MAP) of the XMAP215/Dis1 family; regulates microtubule dynamics during spindle orientation and metaphase chromosome alignment; interacts with spindle pole body component Spc72p	101203	2	1,4	0,04
80	YOR129C	Arf3p polarization-specific docking factor; required for the polarized distribution of the ADP-ribosylation factor, Arf3p; participates in polarity development and maintenance of a normal haploid budding pattern; interacts with Cnm7p	102766	1	1,3	0,04
113	YKR064W	Putative transcriptional repressor with Zn(2)-Cys(6) finger; negatively regulates transcription in response to oleate levels, based on mutant phenotype and localization to oleate-responsive promoters; the authentic, non-tagged protein is detected in	103035	2	1,5	0,09
131	YLR336C	Essential nuclear protein; required for biogenesis of the small ribosomal subunit; has a possible role in the osmoregulatory glycerol response; putative homolog of human NOM1 which is implicated in acute myeloid leukemia	103079	1	0,9	0,04
56	YMR066W	Mitochondrial protein of unknown function	105204	1	2,2	0,04
104	YDR325W	Subunit of the condensin complex; required for establishment and maintenance of chromosome condensation, chromosome segregation and chromatin binding of the condensin complex; required for clustering of tRNA genes at the nucleolus; required for repl	118648	1	1,4	0,04
109	YBR059C	Ser-Thr protein kinase; member (with Ark1p and Prk1p) of the Ark kinase family; involved in endocytosis and actin cytoskeleton organization	124261	1	0,9	0,03
84	YPR030W	Nuclear ubiquitin protein ligase binding protein; may regulate utilization of nonfermentable carbon sources and endocytosis of plasma membrane proteins; overproduction suppresses chs5 spa2 lethality at high temp; ubiquitinated by Rsp5p, deubiquitina	125410	1	0,7	0,03
67	YDL102W	Catalytic subunit of DNA polymerase delta; required for chromosomal DNA replication during mitosis and meiosis, intragenic recombination, repair of double strand DNA breaks, and DNA replication during nucleotide excision repair (NER)	125700	1	0,5	0,03
42	YBL105C	Protein serine/threonine kinase; essential for cell wall remodeling during growth; localized to sites of polarized growth and the mother-daughter bud neck; homolog of the alpha, beta, and gamma isoforms of mammalian protein kinase C (PKC)	132786	3	1,3	0,07
48	YPR122W	Haploid specific endoprotease of a-factor mating pheromone; performs one of two N-terminal cleavages during maturation of a-factor mating pheromone; required for axial budding pattern of haploid cells	139172	3	0,6	0,03
112	YJR137C	Sulfite reductase beta subunit; involved in amino acid biosynthesis, transcription repressed by methionine	161525	2	1,2	0,05
103	YLR320W	Subunit of E3 ubiquitin ligase complex involved in replication repair; stabilizes protein components of the replication fork, such as the fork-pausing complex and leading strand polymerase, preventing fork collapse and promoting efficient recovery d	168680	1	0,5	0,03
110	YPL058C	Plasma membrane ATP-binding cassette (ABC) transporter; weak-acid-inducible multidrug transporter required for weak organic acid resistance; induced by sorbate and benzoate and regulated by War1p; mutants exhibit sorbate hypersensitivity	172753	1	0,7	0,02
106	YPL167C	Catalytic subunit of DNA polymerase zeta; involved in translesion synthesis during post-replication repair; required for mutagenesis induced by DNA damage; involved in double-strand break repair; forms a complex with Rev7p, Pol31p and Pol32p	174588	1	0,9	0,02
82	YNR031C	MAP kinase kinase kinase of HOG1 mitogen-activated signaling pathway; interacts with Ssk1p, leading to autophosphorylation and activation of Ssk2p which phosphorylates Pbs2p; also mediates actin cytoskeleton recovery from osmotic stress; a HOG-indep	181284	1	0,4	0,02
130	YOR326W	Type V myosin motor involved in actin-based transport of cargos; required for the polarized delivery of secretory vesicles, the vacuole, late Golgi elements, peroxisomes, and the mitotic spindle; MYO2 has a paralog, MYO4, that arose from the whole g	181380	1	0,6	0,02
81	YKR031C	Phospholipase D; catalyzes the hydrolysis of phosphatidylcholine, producing choline and phosphatidic acid; involved in Sec14p-independent secretion; required for meiosis and spore formation; differently regulated in secretion and meiosis; participat	196011	2	0,4	0,02
79	YLR422W	Protein of unknown function with similarity to human DOCK proteins; interacts with Ino4p; green fluorescent protein (GFP)-fusion protein localizes to the cytoplasm, YLR422W is not an essential protein; DOCK proteins act as guanine nucleotide exchang	223220	2	0,4	0,02

123	YDR457W	E3 ubiquitin ligase of the hect-domain class; has a role in mRNA export from the nucleus and may regulate transcriptional coactivators; involved in degradation of excess histones; interacts with Dia2p and is required for Dia2p degradation; required	376037	1	0,2	0,01

BAND 13

prot_hit_num	prot_acc	prot_desc	prot_mass	prot_matches	prot_cover	emPAI
35	YIR021W-A	Putative protein of unknown function; identified by expression profiling and mass spectrometry	7733	2	10	0,68
46	YBR298C-A	Putative protein of unknown function; identified by gene-trapping, microarray-based expression analysis, and genome-wide homology searching	8576	5	15,1	0,61
15	YDR086C	Subunit of the Sec61p translocation complex (Sec61p-Sss1p-Sbh1p); this complex forms a channel for passage of secretory proteins through the endoplasmic reticulum membrane, and of the Ssh1p complex (Ssh1p-Sbh2p-Sss1p); interacts with Ost4p and Wbp1p	8996	3	12,5	0,57
11	YOR020W-A	Putative protein of unknown function; conserved in A. gossypii; the authentic, non-tagged protein is detected in highly purified mitochondria in high-throughput studies	9612	4	32,2	1,33
4	YDR377W	Subunit f of the F0 sector of mitochondrial F1F0 ATP synthase; F1F0 ATP synthase is a large, evolutionarily conserved enzyme complex required for ATP synthesis	11305	14	29,7	3,26
9	YPR020W	Subunit g of the mitochondrial F1F0 ATP synthase; reversibly phosphorylated on two residues; unphosphorylated form is required for dimerization of the ATP synthase complex, which in turn determines oligomerization of the complex and the shape of inn	12971	12	33	2,56
59	YOR189W	Component of the INO80 chromatiin remodeling complex; target of the Mec1p/Tel1p DNA damage signaling pathway; proposed to link chromatin remodeling to replication checkpoint responses	13084	1	10,3	0,37
76	YOR102W	Dubious open reading frame; unlikely to encode a functional protein, based on available experimental and comparative sequence data; extensively overlaps essential OST2 gene encoding a subunit of the ER lumen oligosaccharyltransferase complex	13510	1	9,5	0,36
16	YBL002W	Histone H2B; core histone protein required for chromatin assembly and chromosome function; nearly identical to HTB1; Rad6p-Bre1p-Lge1p mediated ubiquitination regulates reassembly after DNA replication, transcriptional activation, meiotic DSB format	14229	4	12,2	0,79
41	YOL012C	Histone variant H2AZ; exchanged for histone H2A in nucleosomes by the SWR1 complex; involved in transcriptional regulation through prevention of the spread of silent heterochromatin; Htz1p-containing nucleosomes facilitate RNA Pol II passage by affe	14274	1	6,7	0,33
36	YDR529C	Subunit 7 of ubiquinol cytochrome-c reductase (Complex III); Complex III is a component of the mitochondrial inner membrane electron transport chain; oriented facing the mitochondrial matrix; N-terminus appears to play a role in complex assembly	14614	1	5,5	0,33
8	YCR083W	Mitochondrial thioredoxin; highly conserved oxidoreductase required to maintain the redox homeostasis of the cell, forms the mitochondrial thioredoxin system with Trr2p, redox state is maintained by both Trr2p and Glr1p	14655	8	27,6	2,09
33	YBR010W	Histone H3; core histone protein required for chromatin assembly, part of heterochromatin-mediated telomeric and HM silencing; one of two identical histone H3 proteins (see HHT2); regulated by acetylation, methylation, and phosphorylation; H3K14 ace	15347	2	5,1	0,31
69	YOR298C-A	Transcriptional coactivator; bridges the DNA-binding region of Gcn4p and TATA-binding protein Spt15p; suppressor of frameshift mutations; protein abundance increases in response to DNA replication stress	16394	2	4,6	0,29
30	YDL004W	Delta subunit of the central stalk of mitochondrial F1F0 ATP synthase; F1F0 ATP synthase is a large, evolutionarily conserved enzyme complex required for ATP synthesis; F1 translationally regulates ATP6 and ATP8 expression to achieve a balanced outp	17068	2	4,4	0,27
45	YHR051W	Subunit VI of cytochrome c oxidase (Complex IV); Complex IV is the terminal member of the mitochondrial inner membrane electron transport chain; expression is regulated by oxygen levels	17389	1	6,8	0,27
38	YMR255W	Coiled-coiled protein of unknown function; identified as a high-copy suppressor of a dbp5 mutation; protein abundance increases in response to DNA replication stress	21558	3	3,7	0,21
20	YEL024W	Ubiquinol-cytochrome-c reductase; a Rieske iron-sulfur protein of the mitochondrial cytochrome bc1 complex; transfers electrons from ubiquinol to cytochrome c1 during respiration; during import, Rip1p is first imported into the mitochondrial matrix	23640	2	4,2	0,19
29	YPL078C	Subunit b of the stator stalk of mitochondrial F1F0 ATP synthase; ATP synthase is a large, evolutionarily conserved enzyme complex required for ATP synthesis; contributes to the oligomerization of the complex, which in turn determines the shape of i	26966	1	3,7	0,17

BAND 14

prot_hit_num	prot_acc	prot_desc	prot_mass	prot_matches	prot_cover	emPAI

60	YDR119W-A	Putative protein of unknown function; copurifies with respiratory chain supercomplexes composed of Complex III (ubiquinol-cytochrome c reductase) and Complex IV (cytochrome c oxidase)	7505	1	16,7	0,71
40	YIR021W-A	Putative protein of unknown function; identified by expression profiling and mass spectrometry	7733	1	10	0,68
38	YBR298C-A	Putative protein of unknown function; identified by gene-trapping, microarray-based expression analysis, and genome-wide homology searching	8576	6	15,1	0,61
10	YDR322C-A	Subunit e of mitochondrial F1F0-ATPase; ATPase is a large, evolutionarily conserved enzyme complex required for ATP synthesis; essential for the dimeric and oligomeric state of ATP synthase, which in turn determines the shape of inner membrane crist	10927	14	30,2	5,59
35	YER048W-A	Cysteine desulfurase (Nfs1p) activator; essential for the formation of the persulfide intermediate at the desulfurase active site during pyridoxal phosphate-dependent desulfuration of cysteine; required for mitochondrial iron-sulfur cluster biosynth	11259	2	9,6	0,44
9	YDR377W	Subunit f of the F0 sector of mitochondrial F1F0 ATP synthase; F1F0 ATP synthase is a large, evolutionarily conserved enzyme complex required for ATP synthesis	11305	12	20,8	1,97
11	YOR020C	Mitochondrial matrix co-chaperonin; inhibits the ATPase activity of Hsp60p, a mitochondrial chaperonin; involved in protein folding and sorting in the mitochondria; 10 kD heat shock protein with similarity to E. coli groES	11365	11	33	1,97
4	YPR020W	Subunit g of the mitochondrial F1F0 ATP synthase; reversibly phosphorylated on two residues; unphosphorylated form is required for dimerization of the ATP synthase complex, which in turn determines oligomerization of the complex and the shape of inn	12971	23	33	2,56
19	YBL002W	Histone H2B; core histone protein required for chromatin assembly and chromosome function; nearly identical to HTB1; Rad6p-Bre1p-Lge1p mediated ubiquitination regulates reassembly after DNA replication, transcriptional activation, meiotic DSB format	14229	3	6,9	0,34
20	YDR529C	Subunit 7 of ubiquinol cytochrome-c reductase (Complex III); Complex III is a component of the mitochondrial inner membrane electron transport chain; oriented facing the mitochondrial matrix; N-terminus appears to play a role in complex assembly	14614	3	12,6	0,76
14	YCR083W	Mitochondrial thioredoxin; highly conserved oxidoreductase required to maintain the redox homeostasis of the cell, forms the mitochondrial thioredoxin system with Trr2p, redox state is maintained by both Trr2p and Glr1p	14655	5	22	1,33
39	YGL191W	Subunit VIa of cytochrome c oxidase; present in a subclass of cytochrome c oxidase complexes that may have a role in mimimizing generation of reactive oxygen species; not essential for cytochrome c oxidase activity but may modulate activity in respo	15070	1	7,8	0,32
86	YOR298C-A	Transcriptional coactivator; bridges the DNA-binding region of Gcn4p and TATA-binding protein Spt15p; suppressor of frameshift mutations; protein abundance increases in response to DNA replication stress	16394	1	4,6	0,29
32	YDL004W	Delta subunit of the central stalk of mitochondrial F1F0 ATP synthase; F1F0 ATP synthase is a large, evolutionarily conserved enzyme complex required for ATP synthesis; F1 translationally regulates ATP6 and ATP8 expression to achieve a balanced outp	17068	2	4,4	0,28
7	YHR051W	Subunit VI of cytochrome c oxidase (Complex IV); Complex IV is the terminal member of the mitochondrial inner membrane electron transport chain; expression is regulated by oxygen levels	17389	16	33,1	2,3
28	YKL016C	Subunit d of the stator stalk of mitochondrial F1F0 ATP synthase; F1F0 ATP synthase is a large, evolutionarily conserved enzyme complex required for ATP synthesis	19797	1	5,7	0,23
51	YMR255W	Coiled-coiled protein of unknown function; identified as a high-copy suppressor of a dbp5 mutation; protein abundance increases in response to DNA replication stress	21558	4	3,7	0,21
41	YHR153C	Meiosis-specific protein involved in synaptonemal complex assembly; implicated in regulation of crossover formation; required for sporulation	23175	2	5,1	0,2
80	YGL183C	Protein required for recombination and meiotic nuclear division; forms a complex with Hop2p, which is involved in chromosome pairing and repair of meiotic double-strand breaks	26299	1	4,1	0,17
16	YPL078C	Subunit b of the stator stalk of mitochondrial F1F0 ATP synthase; ATP synthase is a large, evolutionarily conserved enzyme complex required for ATP synthesis; contributes to the oligomerization of the complex, which in turn determines the shape of i	26966	5	10,7	0,59
50	YPL090C	Protein component of the small (40S) ribosomal subunit; homologous to mammalian ribosomal protein S6, no bacterial homolog; RPS6A has a paralog, RPS6B, that arose from the whole genome duplication	27038	1	5,5	0,17
44	YIL020C	Enzyme that catalyzes the fourth step in the histidine pathway; Phosphoribosylformimino-5-aminoimidazole carboxamide ribotide isomerase; mutations cause histidine auxotrophy and sensitivity to Cu, Co, and Ni salts	30002	3	3,4	0,15
5	YNL055C	Mitochondrial porin (voltage-dependent anion channel); outer membrane protein required for maintenance of mitochondrial osmotic stability and mitochondrial membrane permeability; couples the glutathione pools of the intermembrane space (IMS) and the	30526	13	32,9	2,45
81	YIL124W	Bifunctional triacylglycerol lipase and 1-acyl DHAP reductase; NADPH-dependent 1-acyl dihydroxyacetone phosphate reductase involved in phosphatidic acid biosynthesis; lipid droplet triacylglycerol lipase involved in the mobilization of non-polar lip	33025	3	2,4	0,14
29	YGR231C	Subunit of the prohibitin complex (Phb1p-Phb2p); prohibitin is a 1.2 MDa ring-shaped inner mitochondrial membrane chaperone that stabilizes newly synthesized proteins; determinant of replicative life span; involved in mitochondrial segregation; proh	34443	1	2,3	0,13
12	YBR039W	Gamma subunit of the F1 sector of mitochondrial F1F0 ATP synthase; F1F0 ATP synthase is a large, evolutionarily conserved enzyme complex required for ATP synthesis	34445	6	16,4	0,84
58	YGL043W	General transcription elongation factor TFIIS; enables RNA polymerase II to read through blocks to elongation by stimulating cleavage of nascent transcripts stalled at transcription arrest sites; maintains RNAPII elongation activity on ribosomal pro	35228	2	2,6	0,13
34	YJR095W	Mitochondrial succinate-fumarate transporter; transports succinate into and fumarate out of the mitochondrion; required for ethanol and acetate utilization	35492	1	2,5	0,13

8	YPR191W	Subunit 2 of ubiquinol cytochrome-c reductase (Complex III); Complex III is a component of the mitochondrial inner membrane electron transport chain; phosphorylated; transcription is regulated by Hap1p, Hap2p/Hap3p, and heme	40511	11	15,8	0,87
77	YBL016W	Mitogen-activated serine/threonine protein kinase involved in mating; phosphoactivated by Ste7p; substrates include Ste12p, Far1p, Bni1p, Sst2p; inhibits invasive growth during mating by phosphorylating Tec1p, promoting its; inhibits recruitment of	41094	1	3,7	0,11
23	YFL039C	Actin; structural protein involved in cell polarization, endocytosis, and other cytoskeletal functions	41895	5	10,1	0,35
42	Q0075	Protein of unknown function; encoded within an intron of the mitochondrial COX1 gene; translational initiation codon is predicted to be ATA rather than ATG	42368	3	2,5	0,1
67	YGL181W	Protein involved in Arf3p regulation and in transcription regulation; localizes to the nucleus and to endocytic patches; contains an N-terminal Zn-finger and ArfGAP homology domain, a C-terminal glutamine-rich region, and a UBA (ubiquitin associated	44669	1	2,3	0,1
53	YGR122W	Protein that may be involved in pH regulation; probable ortholog of A. nidulans PalC, which is involved in pH regulation and binds to the ESCRT-III complex; null mutant does not properly process Rim101p and has decreased resistance to rapamycin; GFP	45740	7	3,2	0,1
74	YBL058W	UBX (ubiquitin regulatory X) domain-containing protein; regulates Glc7p phosphatase activity; shp1 mutants are impaired in growth and mitotic progression; functions in growth and mitotic progression require Cdc48p binding; mitotic phenotype is cause	47075	1	2,8	0,09
79	YMR068W	Component of a complex containing the Tor2p kinase and other proteins; complex may have a role in regulation of cell growth	47458	1	3,3	0,09
69	YOR375C	NADP(+)-dependent glutamate dehydrogenase; synthesizes glutamate from ammonia and alpha-ketoglutarate; rate of alpha-ketoglutarate utilization differs from Gdh3p; expression regulated by nitrogen and carbon sources; GDH1 has a paralog, GDH3, that ar	49887	1	2	0,09
21	YBL045C	Core subunit of the ubiquinol-cytochrome c reductase complex; the ubiquinol-cytochrome c reductase complex (bc1 complex) is a component of the mitochondrial inner membrane electron transport chain	50255	3	3,3	0,18
26	YDR148C	Dihydrolipoyl transsuccinylase; component of the mitochondrial alpha-ketoglutarate dehydrogenase complex, which catalyzes the oxidative decarboxylation of alpha-ketoglutarate to succinyl-CoA in the TCA cycle; phosphorylated	50457	2	3	0,18
85	YPL133C	Transcription factor involved in regulating gluconeogenesis; also involved in the regulation of glyoxylate cycle genes; member of the zinc cluster family of proteins; confers resistance to ketoconazole	50804	1	2	0,09
82	YBR207W	Putative high affinity iron transporter; involved in transport of intravacuolar stores of iron; forms complex with Fet5p; expression is regulated by iron; proposed to play indirect role in endocytosis; protein abundance increases in response to DNA	51793	1	2,4	0,08
30	YHR005C	Subunit of the G protein involved in pheromone response; GTP-binding alpha subunit of the heterotrimeric G protein; negatively regulates the mating pathway by sequestering G(beta)gamma and by triggering an adaptive response; activates Vps34p at the	54506	2	1,5	0,08
13	YJR121W	Beta subunit of the F1 sector of mitochondrial F1F0 ATP synthase; which is a large, evolutionarily conserved enzyme complex required for ATP synthesis; F1 translationally regulates ATP6 and ATP8 expression to achieve a balanced output of ATP synthas	54818	5	9,6	0,36
78	YDR473C	Splicing factor; component of the U4/U6-U5 snRNP complex	56125	1	1,5	0,08
45	YML056C	Inosine monophosphate dehydrogenase; catalyzes the rate-limiting step in the de novo synthesis of GTP; member of a four-gene family in S. cerevisiae, constitutively expressed; IMD4 has a paralog, IMD3, that arose from the whole genome duplication	56880	2	1,3	0,08
49	YHR048W	Presumed antiporter of the major facilitator superfamily; member of the 12-spanner drug:H(+) antiporter DHA1 family; expression of gene is up-regulated in cells exhibiting reduced susceptibility to azoles	58380	1	1,9	0,08
6	YBL099W	Alpha subunit of the F1 sector of mitochondrial F1F0 ATP synthase; which is a large, evolutionarily conserved enzyme complex required for ATP synthesis; F1 translationally regulates ATP6 and ATP8 expression to achieve a balanced output of ATP syntha	58630	11	9,2	0,43
52	YDR323C	Adaptor protein involved in vesicle-mediated vacuolar protein sorting; multivalent adaptor protein; facilitates vesicle-mediated vacuolar protein sorting by ensuring high-fidelity vesicle docking and fusion, which are essential for targeting of vesi	60508	1	1,9	0,07
72	YNL048W	Alpha-1,2-mannosyltransferase; catalyzes sequential addition of the two terminal alpha 1,2-mannose residues to the Man5GlcNAc2-PP-dolichol intermediate during asparagine-linked glycosylation in the ER	63509	2	1,3	0,07
75	YDR026C	RNA polymerase I termination factor; binds to rDNA terminator element, required for efficient Pol I termination; required for rDNA silencing at NTS1; facilities association of Sir2p with NTS1, contributes to rDNA stability and cell longevity; intera	66717	1	1,4	0,07
76	YKL217W	Monocarboxylate/proton symporter of the plasma membrane; transport activity is dependent on the pH gradient across the membrane; mediates high-affinity uptake of carbon sources lactate, pyuvate, and acetate, and also of the micronutrient selenite, w	69969	1	1,8	0,06
37	YPR074C	Transketolase; catalyzes conversion of xylulose-5-phosphate and ribose-5-phosphate to sedoheptulose-7-phosphate and glyceraldehyde-3-phosphate in the pentose phosphate pathway; needed for synthesis of aromatic amino acids; TKL1 has a paralog, TKL2,	73876	1	1,8	0,06
83	YPL104W	Mitochondrial aspartyl-tRNA synthetase; required for acylation of aspartyl-tRNA; yeast and bacterial aspartyl-, asparaginyl-, and lysyl-tRNA synthetases contain regions with high sequence similarity, suggesting a common ancestral gene	75877	1	1,1	0,06
73	YHR015W	Putative RNA-binding protein; interacts with Mex67p, which is a component of the nuclear pore involved in nuclear mRNA export; MIP6 has a paralog, PES4, that arose from the whole genome duplication	76452	1	1,4	0,06
57	YDR172W	Translation termination factor eRF3; has a role in mRNA deadenylation and decay; altered protein conformation creates the [PSI(+)] prion that modifies cellular fitness, alters translational fidelity by affecting reading frame selection, and results	76793	1	1,5	0,06

43	YKR001C	Dynamin-like GTPase required for vacuolar sorting; also involved in actin cytoskeleton organization, endocytosis, late Golgi-retention of some proteins, regulation of peroxisome biogenesis	78804	1	1,1	0,06
65	YLR233C	TLC1 RNA-associated factor involved in telomere length regulation; recruitment subunit of telomerase; has G-quadruplex promoting activity required for telomere elongation; possible role in activating telomere-bound Est2p-TLC1-RNA; EST1 has a paralog	82502	1	1,1	0,05
68	YKR024C	Putative ATP-dependent RNA helicase of the DEAD-box family; involved in ribosomal biogenesis; required at post-transcriptional step for efficient retrotransposition; essential for growth under anaerobic conditions	83778	1	0,9	0,05
47	YHR182W	Protein of unknown function; green fluorescent protein (GFP)-fusion protein localizes to the cell periphery and cytoplasm; relocalizes from bud neck to cytoplasm upon DNA replication stress	90865	2	1,3	0,05
48	YOR144C	Subunit of an alternative replication factor C complex; important for DNA replication and genome integrity; suppresses spontaneous DNA damage; involved in homologous recombination-mediated repair and telomere homeostasis; required for PCNA (Pol30p)	91669	2	1,5	0,05
66	YKR079C	tRNA 3~-end processing endonuclease tRNase Z; also localized to mitochondria and interacts genetically with Rex2 exonuclease; homolog of the human candidate prostate cancer susceptibility gene ELAC2	97219	1	1,4	0,04
64	YER032W	Protein involved in 3~ mRNA processing; interacts with Ref2p; APCC(Cdh1) substrate; potential Cdc28p substrate	99389	2	2,1	0,09
59	YDR285W	Transverse filament protein of the synaptonemal complex; required for normal levels of meiotic recombination and pairing between homologous chromosome during meiosis; required for meiotic recombination between non-allelc sites; potential Cdc28p subs	100380	1	0,9	0,04
27	YPL036W	Plasma membrane H+-ATPase; isoform of Pma1p, involved in pumping protons out of the cell; regulator of cytoplasmic pH and plasma membrane potential	102630	2	2,3	0,09
56	YDR430C	Lysine-specific metalloprotease of the pitrilysin family; metalloprotease of the intermembrane space; degrades proteins and presequence peptides cleaved from imported proteins; required for normal mitochondrial morphology	112458	3	0,9	0,04
84	YPR026W	Acid trehalase required for utilization of extracellular trehalose; involved in intracellular trehalose degradation during growth recovery after saline stress	137125	1	0,6	0,03
36	YNL250W	Subunit of MRX complex with Mre11p and Xrs2p; complex is involved in processing double-strand DNA breaks in vegetative cells, initiation of meiotic DSBs, telomere maintenance, and nonhomologous end joining; forms nuclear foci upon DNA replication st	152997	4	1,5	0,06
61	YJR137C	Sulfite reductase beta subunit; involved in amino acid biosynthesis, transcription repressed by methionine	161525	2	0,8	0,03
54	YMR247C	RING domain E3 ubiquitin ligase; involved in ubiquitin-mediated degradation of non-stop proteins; component of ribosome-bound RQC (ribosome quality control) complex required for degradation of polypeptides arising from stalled translation; degrades	181290	2	0,4	0,02
62	YLR247C	E3 ubiquitin ligase and putative helicase; involved in synthesis-dependent strand annealing-mediated homologous recombination; ensures precise end-joining along with Srs2p in the Yku70p/Yku80p/Lig4p-dependent nonhomologous end joining (NHEJ) pathway	181971	2	0,5	0,02
55	YNL139C	Subunit of the THO complex; THO is required for efficient transcription elongation and involved in transcriptional elongation-associated recombination; required for LacZ RNA expression from certain plasmids	184686	2	0,6	0,02
46	YDR283C	Protein kinase; phosphorylates the alpha-subunit of translation initiation factor eIF2 (Sui2p) in response to starvation; activated by uncharged tRNAs and the Gcn1p-Gcn20p complex; contributes to DNA damage checkpoint control	190481	3	0,4	0,02
63	YMR229C	RNA binding protein involved in synthesis of both 18S and 5.8S rRNAs; component of both the ribosomal small subunit (SSU) processosome and the 90S preribosome; acts as part of a Mak21p-Noc2p-Rrp5p module that associates with nascent pre-rRNA during	193712	1	0,6	0,02
70	YBR208C	Urea amidolyase; contains both urea carboxylase and allophanate hydrolase activities, degrades urea to CO2 and NH3; expression sensitive to nitrogen catabolite repression and induced by allophanate, an intermediate in allantoin degradation; protein	203503	1	0,5	0,02
71	YLR024C	Cytoplasmic ubiquitin-protein ligase (E3); required for ubiquitylation of Rpn4p; mediates formation of a Mub1p-Ubr2p-Rad6p complex	219539	1	0,4	0,02
33	YLR422W	Protein of unknown function with similarity to human DOCK proteins; interacts with Ino4p; green fluorescent protein (GFP)-fusion protein localizes to the cytoplasm, YLR422W is not an essential protein; DOCK proteins act as guanine nucleotide exchang	223220	5	0,4	0,02

BAND 15

prot_hit_num	prot_acc	prot_desc	prot_mass	prot_matches	prot_cover	emPAI
39	YML081C-A	Subunit of the mitochondrial F1F0 ATP synthase; F1F0 ATP synthase is a large, evolutionarily conserved enzyme complex required for ATP synthesis; termed subunit I or subunit j; does not correspond to known ATP synthase subunits in other organisms	6683	10	42,4	21,69

4	YPL271W	Epsilon subunit of the F1 sector of mitochondrial F1F0 ATP synthase; which is a large, evolutionarily conserved enzyme complex required for ATP synthesis; F1 translationally regulates ATP6 and ATP8 expression to achieve a balanced output of ATP synt	6738	36	91,9	5478,58
48	YLR388W	Protein component of the small (40S) ribosomal subunit; homologous to mammalian ribosomal protein S29 and bacterial S14; RPS29A has a paralog, RPS29B, that arose from the whole genome duplication	6884	4	32,1	2,36
92	YMR256C	Subunit VII of cytochrome c oxidase (Complex IV); Complex IV is the terminal member of the mitochondrial inner membrane electron transport chain	6928	2	15	0,83
117	YDL061C	Protein component of the small (40S) ribosomal subunit; homologous to mammalian ribosomal protein S29 and bacterial S14; RPS29B has a paralog, RPS29A, that arose from the whole genome duplication	6951	1	17,9	0,82
53	YDL067C	Subunit VIIa of cytochrome c oxidase (Complex IV); Complex IV is the terminal member of the mitochondrial inner membrane electron transport chain	6959	4	32,2	4,99
121	YBR162W-A	Protein of unknown function; expression suppresses a secretory pathway mutation in E. coli; has similarity to the mammalian RAMP4 protein involved in secretion	7360	1	10,8	0,77
47	YGR183C	Subunit 9 of ubiquinol cytochrome-c reductase (Complex III); Complex III is a component of the mitochondrial inner membrane electron transport chain; required for electron transfer at the ubiquinol oxidase site of the complex	7472	4	27,3	2,09
33	YDR119W-A	Putative protein of unknown function; copurifies with respiratory chain supercomplexes composed of Complex III (ubiquinol-cytochrome c reductase) and Complex IV (cytochrome c oxidase)	7504	4	16,7	0,74
68	YOL077W-A	Subunit k of the mitochondrial F1F0 ATP synthase; F1F0 ATP synthase is a large, evolutionarily conserved enzyme complex required for ATP synthesis; associated only with the dimeric form of ATP synthase	7529	4	23,5	4,3
22	YOR167C	Protein component of the small (40S) ribosomal subunit; has an extraribosomal function in regulation of RPS28B, in which Rps28Ap binds to a decapping complex via Edc3p, which then binds to RPS28B mRNA leading to its decapping and degradation; homolo	7587	6	41,8	4,3
125	YML009C	Mitochondrial ribosomal protein of the large subunit	7968	1	12,9	0,69
130	YCL005W-A	Vacuolar H+ ATPase subunit e of the V-ATPase V0 subcomplex; essential for vacuolar acidification; interacts with the V-ATPase assembly factor Vma21p in the ER; involved in V0 biogenesis	8432	1	12,3	0,65
88	YNL015W	Cytosolic inhibitor of vacuolar proteinase B (PRB1); required for efficient vacuole inheritance; with thioredoxin forms protein complex LMA1, which assists in priming SNARE molecules and promotes vacuole fusion; protein abundance increases in respon	8585	2	9,3	0,64
30	YHR001W-A	Subunit of the ubiqunol-cytochrome c oxidoreductase complex; this complex comprises part of the mitochondrial respiratory chain; members include Cobp, Rip1p, Cyt1p, Cor1p, Qcr2p, Qcr6p, Qcr7p, Qcr8p, Qcr9p, and Qcr10p and comprises part of the mitoc	8587	7	55,8	6,25
90	YER087C-B	Beta subunit of Sec61p ER translocation complex (Sec61p-Sss1p-Sbh1p); involved in protein translocation into the endoplasmic reticulum; interacts with the exocyst complex and also with Rtn1p; cotranslationally N-acetylated by NatA; SBH1 has a paralo	8706	1	12,2	0,63
15	YLR325C	Ribosomal 60S subunit protein L38; homologous to mammalian ribosomal protein L38, no bacterial homolog	8821	10	32,1	1,63
34	YDR086C	Subunit of the Sec61p translocation complex (Sec61p-Sss1p-Sbh1p); this complex forms a channel for passage of secretory proteins through the endoplasmic reticulum membrane, and of the Ssh1p complex (Ssh1p-Sbh2p-Sss1p); interacts with Ost4p and Wbp1p	8995	3	12,5	0,6
42	YLR395C	Subunit VIII of cytochrome c oxidase (Complex IV); Complex IV is the terminal member of the mitochondrial inner membrane electron transport chain	9015	3	12,8	0,6
32	YHR021C	Protein component of the small (40S) ribosomal subunit; homologous to mammalian ribosomal protein S27, no bacterial homolog; RPS27B has a paralog, RPS27A, that arose from the whole genome duplication	9145	7	37,8	3,06
161	YDR379C-A	Mitochondrial protein involved in assembly of succinate dehydrogenase; has a role in maturation of the Sdh2p subunit; member of the LYR protein family; mutations in human ortholog SDHAF1 are associated with infantile leukoencephalopathy	9346	1	16,5	0,58
64	YER019C-A	Ssh1p-Sss1p-Sbh2p complex component; involved in protein translocation into the endoplasmic reticulum; SBH2 has a paralog, SBH1, that arose from the whole genome duplication	9600	1	14,8	0,56
119	YOR020W-A	Putative protein of unknown function; conserved in A. gossypii; the authentic, non-tagged protein is detected in highly purified mitochondria in high-throughput studies	9612	1	20	0,55
97	YKR057W	Protein component of the small (40S) ribosomal subunit; homologous to mammalian ribosomal protein S21, no bacterial homolog; RPS21A has a paralog, RPS21B, that arose from the whole genome duplication	9797	1	10,3	0,55
26	YDL181W	Protein that inhibits ATP hydrolysis by the F1F0-ATP synthase; inhibitory function is enhanced by stabilizing proteins Stf1p and Stf2p; has a calmodulin-binding motif and binds calmodulin in vitro; INH1 has a paralog, STF1, that arose from the whole	9864	8	31,8	7,67
109	YDL130W-A	Protein involved in regulation of the mitochondrial F1F0-ATP synthase; Stf1p and Stf2p act as stabilizing factors that enhance inhibitory action of the Inh1p protein; protein abundance increases in response to DNA replication stress; STF1 has a para	10113	1	9,3	0,53
120	YNR037C	Mitochondrial ribosomal protein of the small subunit; has similarity to E. coli S19 ribosomal protein	10269	1	13,2	0,51
93	YCL057C-A	Conserved component of the MICOS complex; MICOS (formerly MINOS or MitOS) is a mitochondrial inner membrane complex that extends into the intermembrane space and has a role in the maintenance of crista junctions, inner membrane architecture, and for	10401	1	10,3	0,51
18	YDR322C-A	Subunit e of mitochondrial F1F0-ATPase; ATPase is a large, evolutionarily conserved enzyme complex required for ATP synthesis; essential for the dimeric and oligomeric state of ATP synthase, which in turn determines the shape of inner membrane crist	10926	9	21,9	2,26
23	YJL166W	Subunit 8 of ubiquinol cytochrome-c reductase (Complex III); Complex III is a component of the mitochondrial inner membrane electron transport chain; oriented facing the intermembrane space; expression is regulated by Abf1p and Cpf1p	10967	8	43,6	3,77
66	YMR194W	Ribosomal 60S subunit protein L36A; N-terminally acetylated; binds to 5.8 S rRNA; homologous to mammalian ribosomal protein L36, no bacterial homolog; RPL36A has a paralog, RPL36B, that arose from the whole genome duplication	11117	3	17	1,17

19	YER048W-A	Cysteine desulfurase (Nfs1p) activator; essential for the formation of the persulfide intermediate at the desulfurase active site during pyridoxal phosphate-dependent desulfuration of cysteine; required for mitochondrial iron-sulfur cluster biosynth	11259	10	50	13,62
8	YDR377W	Subunit f of the F0 sector of mitochondrial F1F0 ATP synthase; F1F0 ATP synthase is a large, evolutionarily conserved enzyme complex required for ATP synthesis	11305	22	36,6	19,85
13	YBR009C	Histone H4; core histone protein required for chromatin assembly and chromosome function; one of two identical histone proteins (see also HHF2); contributes to telomeric silencing; N-terminal domain involved in maintaining genomic integrity	11361	10	38,8	3,57
6	YOR020C	Mitochondrial matrix co-chaperonin; inhibits the ATPase activity of Hsp60p, a mitochondrial chaperonin; involved in protein folding and sorting in the mitochondria; 10 kD heat shock protein with similarity to E. coli groES	11365	20	67,9	8,76
20	YGL030W	Ribosomal 60S subunit protein L30; involved in pre-rRNA processing in the nucleolus; autoregulates splicing of its transcript; homologous to mammalian ribosomal protein L30, no bacterial homolog	11408	5	27,6	1,12
111	YDR115W	Putative mitochondrial ribosomal protein of the large subunit; similar to E. coli L34 ribosomal protein; required for respiratory growth, as are most mitochondrial ribosomal proteins; protein increases in abundance and relocalizes to the plasma memb	12080	1	6,7	0,43
80	YPL143W	Ribosomal 60S subunit protein L33A; N-terminally acetylated; rpl33a null mutant exhibits slow growth while rpl33a rpl33b double null mutant is inviable; homologous to mammalian ribosomal protein L35A, no bacterial homolog; RPL33A has a paralog, RPL3	12147	1	9,3	0,43
7	YJR048W	Cytochrome c, isoform 1; also known as iso-1-cytochrome c; electron carrier of the mitochondrial intermembrane space that transfers electrons from ubiquinone-cytochrome c oxidoreductase to cytochrome c oxidase during cellular respiration; mutations	12345	18	48,6	10,61
55	YGR215W	Mitochondrial ribosomal protein of the small subunit	12385	2	10,9	0,42
63	YOL109W	Peripheral membrane protein of the plasma membrane; interacts with Mid2p; regulates the cell integrity pathway mediated by Pkc1p and Slt2p; the authentic protein is detected in a phosphorylated state in highly purified mitochondria	12582	3	12,4	1,81
7	YEL039C	Cytochrome c isoform 2, expressed under hypoxic conditions; also known as iso-2-cytochrome c; electron carrier of the mitochondrial intermembrane space that transfers electrons from ubiquinone-cytochrome c oxidoreductase to cytochrome c oxidase duri	12638	6	15,9	0
59	YMR230W	Protein component of the small (40S) ribosomal subunit; homologous to mammalian ribosomal protein S10, no bacterial homolog; RPS10B has a paralog, RPS10A, that arose from the whole genome duplication	12731	3	21	0,97
57	YDL075W	Ribosomal 60S subunit protein L31A; associates with karyopherin Sxm1p; loss of both Rpl31p and Rpl39p confers lethality; homologous to mammalian ribosomal protein L31, no bacterial homolog; RPL31A has a paralog, RPL31B, that arose from the whole gen	12945	3	9,7	0,4
14	YPR020W	Subunit g of the mitochondrial F1F0 ATP synthase; reversibly phosphorylated on two residues; unphosphorylated form is required for dimerization of the ATP synthase complex, which in turn determines oligomerization of the complex and the shape of inn	12970	14	36,5	4,29
85	YOR327C	Vesicle membrane receptor protein (v-SNARE); involved in the fusion between Golgi-derived secretory vesicles with the plasma membrane; Snc2p levels regulated by Vps45p; member of the synaptobrevin/VAMP family of R-type v-SNARE proteins; SNC2 has a p	13006	1	10,4	0,4
157	YER131W	Protein component of the small (40S) ribosomal subunit; homologous to mammalian ribosomal protein S26, no bacterial homolog; RPS26B has a paralog, RPS26A, that arose from the whole genome duplication	13666	1	7,6	0,37
146	YFL034C-A	Ribosomal 60S subunit protein L22B; homologous to mammalian ribosomal protein L22, no bacterial homolog; RPL22B has a paralog, RPL22A, that arose from the whole genome duplication	13818	1	5,7	0,37
112	YIL052C	Ribosomal 60S subunit protein L34B; homologous to mammalian ribosomal protein L34, no bacterial homolog; RPL34B has a paralog, RPL34A, that arose from the whole genome duplication	13861	2	6,6	0,37
95	YBL003C	Histone H2A; core histone protein required for chromatin assembly and chromosome function; one of two nearly identical (see also HTA1) subtypes; DNA damage-dependent phosphorylation by Mec1p facilitates DNA repair; acetylated by Nat4p	13981	2	6,8	0,36
96	YER057C	Member of the p14.5 protein family; functionally complements Mmf1p function when targeted to mitochondria; heat shock inducible; high-dosage growth inhibitor; forms a homotrimer in vitro; HMF1 has a paralog, MMF1, that arose from the whole genome du	14011	1	7	0,36
148	YIL163C	Protein of unknown function; mRNA identified as translated by ribosome profiling data	14094	1	6	0,36
105	YGR034W	Ribosomal 60S subunit protein L26B; binds to 5.8S rRNA; non-essential even when paralog is also deleted; deletion has minimal affections on ribosome biosynthesis; homologous to mammalian ribosomal protein L26 and bacterial L24; RPL26B has a paralog,	14226	1	7,1	0,36
49	YBL002W	Histone H2B; core histone protein required for chromatin assembly and chromosome function; nearly identical to HTB1; Rad6p-Bre1p-Lge1p mediated ubiquitination regulates reassembly after DNA replication, transcriptional activation, meiotic DSB format	14229	5	15,3	0,84
39	YJL086C	Dubious open reading frame; unlikely to encode a functional protein, based on available experimental and comparative sequence data; partially overlaps the verified genes YJL085W/EXO70 and YJL087C/TRL1	14259	3	6,6	0
116	YOL012C	Histone variant H2AZ; exchanged for histone H2A in nucleosomes by the SWR1 complex; involved in transcriptional regulation through prevention of the spread of silent heterochromatin; Htz1p-containing nucleosomes facilitate RNA Pol II passage by affe	14274	1	6,7	0,35
72	YBL087C	Ribosomal 60S subunit protein L23A; homologous to mammalian ribosomal protein L23 and bacterial L14; RPL23A has a paralog, RPL23B, that arose from the whole genome duplication	14578	3	11,7	0,82
51	YCR031C	Protein component of the small (40S) ribosomal subunit; required for ribosome assembly and 20S pre-rRNA processing; mutations confer cryptopleurine resistance; homologous to mammalian ribosomal protein S14 and bacterial S11; RPS14A has a paralog, RP	14585	3	17,5	0,82

56	YDR529C	Subunit 7 of ubiquinol cytochrome-c reductase (Complex III); Complex III is a component of the mitochondrial inner membrane electron transport chain; oriented facing the mitochondrial matrix; N-terminus appears to play a role in complex assembly	14613	8	31,5	2,28
44	YCR083W	Mitochondrial thioredoxin; highly conserved oxidoreductase required to maintain the redox homeostasis of the cell, forms the mitochondrial thioredoxin system with Trr2p, redox state is maintained by both Trr2p and Glr1p	14651	4	22	1,43
17	YGL191W	Subunit VIa of cytochrome c oxidase; present in a subclass of cytochrome c oxidase complexes that may have a role in mimimizing generation of reactive oxygen species; not essential for cytochrome c oxidase activity but may modulate activity in respo	15069	12	29,5	4,64
87	YHL001W	Ribosomal 60S subunit protein L14B; homologous to mammalian ribosomal protein L14, no bacterial homolog; RPL14B has a paralog, RPL14A, that arose from the whole genome duplication; protein abundance increases in response to DNA replication stress	15201	2	5,1	0,33
113	YIL069C	Protein component of the small (40S) ribosomal subunit; homologous to mammalian ribosomal protein S24, no bacterial homolog; RPS24B has a paralog, RPS24A, that arose from the whole genome duplication	15319	1	6,7	0,33
128	YHR059W	Protein of unknown function; required for survival upon exposure to K1 killer toxin	15339	1	5,4	0,33
38	YOR369C	Protein component of the small (40S) ribosomal subunit; homologous to mammalian ribosomal protein S12, no bacterial homolog	15462	6	29,4	2,09
46	YOL127W	Ribosomal 60S subunit protein L25; primary rRNA-binding ribosomal protein component of large ribosomal subunit; binds to 25S rRNA via a conserved C-terminal motif; homologous to mammalian ribosomal protein L23A and bacterial L23	15748	4	24,6	1,29
45	YML024W	Ribosomal protein 51 (rp51) of the small (40s) subunit; homologous to mammalian ribosomal protein S17, no bacterial homolog; RPS17A has a paralog, RPS17B, that arose from the whole genome duplication	15836	4	22,8	1,28
36	YOL121C	Protein component of the small (40S) ribosomal subunit; required for assembly and maturation of pre-40 S particles; homologous to mammalian ribosomal protein S19, no bacterial homolog; mutations in human RPS19 are associated with Diamond Blackfan an	15907	5	11,1	0,73
54	YIL051C	Mitochondrial protein required for transamination of isoleucine; but not of valine or leucine; may regulate specificity of branched-chain transaminases Bat1p and Bat2p; induction of expression in response to stress is mediated by a Hog1p-regulated a	15955	4	19,3	1,27
70	YOR286W	Protein with rhodanese activity; contains a rhodanese-like domain similar to Rdl1p, Uba4p, Tum1p, and Ych1p; overexpression causes a cell cycle delay; null mutant displays elevated frequency of mitochondrial genome loss	16744	3	11,4	0,68
75	YDL004W	Delta subunit of the central stalk of mitochondrial F1F0 ATP synthase; F1F0 ATP synthase is a large, evolutionarily conserved enzyme complex required for ATP synthesis; F1 translationally regulates ATP6 and ATP8 expression to achieve a balanced outp	17067	2	11,2	0,67
94	YML026C	Protein component of the small (40S) ribosomal subunit; homologous to mammalian ribosomal protein S18 and bacterial S13; RPS18B has a paralog, RPS18A, that arose from the whole genome duplication; protein abundance increases in response to DNA repli	17084	1	7,5	0,29
136	YDR155C	Cytoplasmic peptidyl-prolyl cis-trans isomerase (cyclophilin); catalyzes the cis-trans isomerization of peptide bonds N-terminal to proline residues; binds the drug cyclosporin A; N-terminally propionylated in vivo; protein abundance increases in re	17494	1	4,9	0,28
114	YEL054C	Ribosomal 60S subunit protein L12A; rpl12a rpl12b double mutant exhibits slow growth and slow translation; homologous to mammalian ribosomal protein L12 and bacterial L11; RPL12A has a paralog, RPL12B, that arose from the whole genome duplication	17869	1	5,5	0,28
143	YOR339C	Ubiquitin-conjugating enzyme; most similar in sequence to Xenopus ubiquitin-conjugating enzyme E2-C, but not a true functional homolog of this E2; unlike E2-C, not required for the degradation of mitotic cyclin Clb2	17913	1	4,5	0,28
40	YKL016C	Subunit d of the stator stalk of mitochondrial F1F0 ATP synthase; F1F0 ATP synthase is a large, evolutionarily conserved enzyme complex required for ATP synthesis	19797	2	7,5	0,25
138	YMR242C	Ribosomal 60S subunit protein L20A; homologous to mammalian ribosomal protein L18A, no bacterial homolog; RPL20A has a paralog, RPL20B, that arose from the whole genome duplication	20424	1	4,1	0,24
126	YEL024W	Ubiquinol-cytochrome-c reductase; a Rieske iron-sulfur protein of the mitochondrial cytochrome bc1 complex; transfers electrons from ubiquinol to cytochrome c1 during respiration; during import, Rip1p is first imported into the mitochondrial matrix	23635	1	4,2	0,21
139	YFL005W	Rab family GTPase; essential for vesicle-mediated exocytic secretion and autophagy; associates with the exocyst component Sec15p and may regulate polarized delivery of transport vesicles to the exocyst at the plasma membrane	23662	1	5,6	0,21
137	YMR090W	Putative protein of unknown function; similar to DTDP-glucose 4,6-dehydratases; GFP-fusion protein localizes to the cytoplasm; up-regulated in response to the fungicide mancozeb; not essential for viability	24981	2	3,5	0,19
132	YNL178W	Protein component of the small (40S) ribosomal subunit; has apurinic/apyrimidinic (AP) endonuclease activity; essential for viability; homologous to mammalian ribosomal protein S3 and bacterial S3	26543	1	2,9	0,18
61	YPL078C	Subunit b of the stator stalk of mitochondrial F1F0 ATP synthase; ATP synthase is a large, evolutionarily conserved enzyme complex required for ATP synthesis; contributes to the oligomerization of the complex, which in turn determines the shape of i	26965	4	13,5	0,93

BAND 16

prot_hit_num	prot_acc	prot_desc	prot_mass	prot_matches	prot_cover	emPAI
16	YML081C-A	Subunit of the mitochondrial F1F0 ATP synthase; F1F0 ATP synthase is a large, evolutionarily conserved enzyme complex required for ATP synthesis; termed subunit I or subunit j; does not correspond to known ATP synthase subunits in other organisms	6683	15	42,4	42,28
8	YPL271W	Epsilon subunit of the F1 sector of mitochondrial F1F0 ATP synthase; which is a large, evolutionarily conserved enzyme complex required for ATP synthesis; F1 translationally regulates ATP6 and ATP8 expression to achieve a balanced output of ATP synt	6738	22	91,9	1670,98
72	YLR388W	Protein component of the small (40S) ribosomal subunit; homologous to mammalian ribosomal protein S29 and bacterial S14; RPS29A has a paralog, RPS29B, that arose from the whole genome duplication	6884	2	32,1	2,38
98	YMR256C	Subunit VII of cytochrome c oxidase (Complex IV); Complex IV is the terminal member of the mitochondrial inner membrane electron transport chain	6928	2	11,7	0,84
39	YDL067C	Subunit VIIa of cytochrome c oxidase (Complex IV); Complex IV is the terminal member of the mitochondrial inner membrane electron transport chain	6959	5	32,2	10,04
56	YGR183C	Subunit 9 of ubiquinol cytochrome-c reductase (Complex III); Complex III is a component of the mitochondrial inner membrane electron transport chain; required for electron transfer at the ubiquinol oxidase site of the complex	7472	2	16,7	0,76
40	YDR119W-A	Putative protein of unknown function; copurifies with respiratory chain supercomplexes composed of Complex III (ubiquinol-cytochrome c reductase) and Complex IV (cytochrome c oxidase)	7504	2	16,7	0,75
32	YOL077W-A	Subunit k of the mitochondrial F1F0 ATP synthase; F1F0 ATP synthase is a large, evolutionarily conserved enzyme complex required for ATP synthesis; associated only with the dimeric form of ATP synthase	7529	7	36,8	15,4
20	YOR167C	Protein component of the small (40S) ribosomal subunit; has an extraribosomal function in regulation of RPS28B, in which Rps28Ap binds to a decapping complex via Edc3p, which then binds to RPS28B mRNA leading to its decapping and degradation; homolo	7587	6	41,8	4,36
97	YIR021W-A	Putative protein of unknown function; identified by expression profiling and mass spectrometry	7732	2	10	0,72
75	YML129C	Mitochondrial cytochrome c oxidase (complex IV) assembly factor; also involved in translational regulation of Cox1p and prevention of Cox1p aggregation before assembly; associates with complex IV assembly intermediates and complex III/complex IV sup	7954	1	10	0,7
62	YIL156W-B	Putative protein of unknown function; originally identified based on homology to <i>Ashbya gossypii</i> and other related yeasts	8273	1	13,7	0,67
95	YNL015W	Cytosolic inhibitor of vacuolar proteinase B (PRB1); required for efficient vacuole inheritance; with thioredoxin forms protein complex LMA1, which assists in priming SNARE molecules and promotes vacuole fusion; protein abundance increases in respon	8585	1	9,3	0,65
26	YHR001W-A	Subunit of the ubiqunol-cytochrome c oxidoreductase complex; this complex comprises part of the mitochondrial respiratory chain; members include Cobp, Rip1p, Cyt1p, Cor1p, Qcr2p, Qcr6p, Qcr7p, Qcr8p, Qcr9p, and Qcr10p and comprises part of the mitoc	8587	7	31,2	3,46
84	YER087C-B	Beta subunit of Sec61p ER translocation complex (Sec61p-Sss1p-Sbh1p); involved in protein translocation into the endoplasmic reticulum; interacts with the exocyst complex and also with Rtn1p; cotranslationally N-acetylated by NatA; SBH1 has a paralo	8706	1	12,2	0,64
34	YLR325C	Ribosomal 60S subunit protein L38; homologous to mammalian ribosomal protein L38, no bacterial homolog	8821	5	41	3,3
51	YDR086C	Subunit of the Sec61p translocation complex (Sec61p-Sss1p-Sbh1p); this complex forms a channel for passage of secretory proteins through the endoplasmic reticulum membrane, and of the Ssh1p complex (Ssh1p-Sbh2p-Sss1p); interacts with Ost4p and Wbp1p	8995	1	12,5	0,61
29	YLR395C	Subunit VIII of cytochrome c oxidase (Complex IV); Complex IV is the terminal member of the mitochondrial inner membrane electron transport chain	9015	4	12,8	0,61
28	YHR021C	Protein component of the small (40S) ribosomal subunit; homologous to mammalian ribosomal protein S27, no bacterial homolog; RPS27B has a paralog, RPS27A, that arose from the whole genome duplication	9145	6	37,8	3,09
47	YER019C-A	Ssh1p-Sss1p-Sbh2p complex component; involved in protein translocation into the endoplasmic reticulum; SBH2 has a paralog, SBH1, that arose from the whole genome duplication	9600	1	14,8	0,57
90	YKR057W	Protein component of the small (40S) ribosomal subunit; homologous to mammalian ribosomal protein S21, no bacterial homolog; RPS21A has a paralog, RPS21B, that arose from the whole genome duplication	9797	1	10,3	0,55
18	YDL181W	Protein that inhibits ATP hydrolysis by the F1F0-ATP synthase; inhibitory function is enhanced by stabilizing proteins Stf1p and Stf2p; has a calmodulin-binding motif and binds calmodulin in vitro; INH1 has a paralog, STF1, that arose from the whole	9864	8	43,5	12,59
43	YCL057C-A	Conserved component of the MICOS complex; MICOS (formerly MINOS or MitOS) is a mitochondrial inner membrane complex that extends into the intermembrane space and has a role in the maintenance of crista junctions, inner membrane architecture, and for	10401	3	22,7	1,28
106	YNR034W-A	Putative protein of unknown function; expression is regulated by Msn2p/Msn4p; YNR034W-A has a paralog, YCR075W-A, that arose from the whole genome duplication	10775	1	8,2	0,49

4	YDR322C-A	Subunit e of mitochondrial F1F0-ATPase; ATPase is a large, evolutionarily conserved enzyme complex required for ATP synthesis; essential for the dimeric and oligomeric state of ATP synthase, which in turn determines the shape of inner membrane crist	10926	31	57,3	34,61
52	YJL166W	Subunit 8 of ubiquinol cytochrome-c reductase (Complex III); Complex III is a component of the mitochondrial inner membrane electron transport chain; oriented facing the intermembrane space; expression is regulated by Abf1p and Cpf1p	10967	2	21,3	1,2
78	YMR194W	Ribosomal 60S subunit protein L36A; N-terminally acetylated; binds to 5.8 S rRNA; homologous to mammalian ribosomal protein L36, no bacterial homolog; RPL36A has a paralog, RPL36B, that arose from the whole genome duplication	11117	1	10	0,48
33	YER048W-A	Cysteine desulfurase (Nfs1p) activator; essential for the formation of the persulfide intermediate at the desulfurase active site during pyridoxal phosphate-dependent desulfuration of cysteine; required for mitochondrial iron-sulfur cluster biosynth	11259	6	39,4	5,88
10	YDR377W	Subunit f of the F0 sector of mitochondrial F1F0 ATP synthase; F1F0 ATP synthase is a large, evolutionarily conserved enzyme complex required for ATP synthesis	11305	17	36,6	20,27
24	YBR009C	Histone H4; core histone protein required for chromatin assembly and chromosome function; one of two identical histone proteins (see also HHF2); contributes to telomeric silencing; N-terminal domain involved in maintaining genomic integrity	11361	6	38,8	3,61
7	YOR020C	Mitochondrial matrix co-chaperonin; inhibits the ATPase activity of Hsp60p, a mitochondrial chaperonin; involved in protein folding and sorting in the mitochondria; 10 kD heat shock protein with similarity to E. coli groES	11365	21	67,9	13,51
21	YGL030W	Ribosomal 60S subunit protein L30; involved in pre-rRNA processing in the nucleolus; autoregulates splicing of its transcript; homologous to mammalian ribosomal protein L30, no bacterial homolog	11408	4	27,6	1,13
92	YGR027C	Protein component of the small (40S) ribosomal subunit; homologous to mammalian ribosomal protein S25, no bacterial homolog; RPS25A has a paralog, RPS25B, that arose from the whole genome duplication	12032	1	10,2	0,44
11	YJR048W	Cytochrome c, isoform 1; also known as iso-1-cytochrome c; electron carrier of the mitochondrial intermembrane space that transfers electrons from ubiquinone-cytochrome c oxidoreductase to cytochrome c oxidase during cellular respiration; mutations	12345	14	48,6	15,79
112	YOL109W	Peripheral membrane protein of the plasma membrane; interacts with Mid2p; regulates the cell integrity pathway mediated by Pkc1p and Slt2p; the authentic protein is detected in a phosphorylated state in highly purified mitochondria	12582	1	6,2	0,41
11	YEL039C	Cytochrome c isoform 2, expressed under hypoxic conditions; also known as iso-2-cytochrome c; electron carrier of the mitochondrial intermembrane space that transfers electrons from ubiquinone-cytochrome c oxidoreductase to cytochrome c oxidase duri	12638	5	15,9	
68	YMR230W	Protein component of the small (40S) ribosomal subunit; homologous to mammalian ribosomal protein S10, no bacterial homolog; RPS10B has a paralog, RPS10A, that arose from the whole genome duplication	12731	2	21	0,98
6	YPR020W	Subunit g of the mitochondrial F1F0 ATP synthase; reversibly phosphorylated on two residues; unphosphorylated form is required for dimerization of the ATP synthase complex, which in turn determines oligomerization of the complex and the shape of inn	12970	27	51,3	6,48
103	YER131W	Protein component of the small (40S) ribosomal subunit; homologous to mammalian ribosomal protein S26, no bacterial homolog; RPS26B has a paralog, RPS26A, that arose from the whole genome duplication	13666	1	7,6	0,38
73	YBL003C	Histone H2A; core histone protein required for chromatin assembly and chromosome function; one of two nearly identical (see also HTA1) subtypes; DNA damage-dependent phosphorylation by Mec1p facilitates DNA repair; acetylated by Nat4p	13981	2	6,8	0,37
91	YER057C	Member of the p14.5 protein family; functionally complements Mmf1p function when targeted to mitochondria; heat shock inducible; high-dosage growth inhibitor; forms a homotrimer in vitro; HMF1 has a paralog, MMF1, that arose from the whole genome du	14011	1	9,3	0,37
122	YIL163C	Protein of unknown function; mRNA identified as translated by ribosome profiling data	14094	1	6	0,36
50	YGR034W	Ribosomal 60S subunit protein L26B; binds to 5.8S rRNA; non-essential even when paralog is also deleted; deletion has minimal affections on ribosome biosynthesis; homologous to mammalian ribosomal protein L26 and bacterial L24; RPL26B has a paralog,	14226	4	12,6	0,85
49	YBL002W	Histone H2B; core histone protein required for chromatin assembly and chromosome function; nearly identical to HTB1; Rad6p-Bre1p-Lge1p mediated ubiquitination regulates reassembly after DNA replication, transcriptional activation, meiotic DSB format	14229	3	6,9	0,36
16	YJL086C	Dubious open reading frame; unlikely to encode a functional protein, based on available experimental and comparative sequence data; partially overlaps the verified genes YJL085W/EXO70 and YJL087C/TRL1	14259	4	6,6	
116	YOL012C	Histone variant H2AZ; exchanged for histone H2A in nucleosomes by the SWR1 complex; involved in transcriptional regulation through prevention of the spread of silent heterochromatin; Htz1p-containing nucleosomes facilitate RNA Pol II passage by affe	14274	1	6,7	0,36
55	YBL087C	Ribosomal 60S subunit protein L23A; homologous to mammalian ribosomal protein L23 and bacterial L14; RPL23A has a paralog, RPL23B, that arose from the whole genome duplication	14578	3	11,7	0,83
41	YCR031C	Protein component of the small (40S) ribosomal subunit; required for ribosome assembly and 20S pre-rRNA processing; mutations confer cryptopleurine resistance; homologous to mammalian ribosomal protein S14 and bacterial S11; RPS14A has a paralog, RP	14585	3	17,5	0,83
53	YKR094C	Ubiquitin-ribosomal 60S subunit protein L40B fusion protein; cleaved to yield ubiquitin and ribosomal protein L40B; ubiquitin may facilitate assembly of the ribosomal protein into ribosomes; homologous to mammalian ribosomal protein L40, no bacteria	14830	3	12,5	0,8
14	YGL191W	Subunit VIa of cytochrome c oxidase; present in a subclass of cytochrome c oxidase complexes that may have a role in mimimizing generation of reactive oxygen species; not essential for cytochrome c oxidase activity but may modulate activity in respo	15069	14	29,5	4,7
93	YHL001W	Ribosomal 60S subunit protein L14B; homologous to mammalian ribosomal protein L14, no bacterial homolog; RPL14B has a paralog, RPL14A, that arose from the whole genome duplication; protein abundance increases in response to DNA replication stress	15201	1	5,1	0,33

25	YOR369C	Protein component of the small (40S) ribosomal subunit; homologous to mammalian ribosomal protein S12, no bacterial homolog	15462	6	30,1	2,12
45	YML024W	Ribosomal protein 51 (rp51) of the small (40s) subunit; homologous to mammalian ribosomal protein S17, no bacterial homolog; RPS17A has a paralog, RPS17B, that arose from the whole genome duplication	15836	3	15,4	0,74
67	YOL121C	Protein component of the small (40S) ribosomal subunit; required for assembly and maturation of pre-40 S particles; homologous to mammalian ribosomal protein S19, no bacterial homolog; mutations in human RPS19 are associated with Diamond Blackfan an	15907	1	6,2	0,32
96	YIL051C	Mitochondrial protein required for transamination of isoleucine; but not of valine or leucine; may regulate specificity of branched-chain transaminases Bat1p and Bat2p; induction of expression in response to stress is mediated by a Hog1p-regulated a	15955	1	6,9	0,32
46	YDL004W	Delta subunit of the central stalk of mitochondrial F1F0 ATP synthase; F1F0 ATP synthase is a large, evolutionarily conserved enzyme complex required for ATP synthesis; F1 translationally regulates ATP6 and ATP8 expression to achieve a balanced outp	17067	3	11,2	0,67
64	YEL054C	Ribosomal 60S subunit protein L12A; rpl12a rpl12b double mutant exhibits slow growth and slow translation; homologous to mammalian ribosomal protein L12 and bacterial L11; RPL12A has a paralog, RPL12B, that arose from the whole genome duplication	17869	2	5,5	0,28
66	YKL016C	Subunit d of the stator stalk of mitochondrial F1F0 ATP synthase; F1F0 ATP synthase is a large, evolutionarily conserved enzyme complex required for ATP synthesis	19797	1	7,5	0,25
70	YPR165W	GTP-binding protein of the rho subfamily of Ras-like proteins; involved in establishment of cell polarity; regulates protein kinase C (Pkc1p) and the cell wall synthesizing enzyme 1,3-beta-glucan synthase (Fks1p and Gsc2p)	23537	2	3,8	0,21
120	YMR090W	Putative protein of unknown function; similar to DTDP-glucose 4,6-dehydratases; GFP-fusion protein localizes to the cytoplasm; up-regulated in response to the fungicide mancozeb; not essential for viability	24981	2	3,5	0,19
107	YNL178W	Protein component of the small (40S) ribosomal subunit; has apurinic/apyrimidinic (AP) endonuclease activity; essential for viability; homologous to mammalian ribosomal protein S3 and bacterial S3	26543	1	2,9	0,18
79	YPL078C	Subunit b of the stator stalk of mitochondrial F1F0 ATP synthase; ATP synthase is a large, evolutionarily conserved enzyme complex required for ATP synthesis; contributes to the oligomerization of the complex, which in turn determines the shape of i	26965	2	7,4	0,39

BAND 17

Sequence coverage	Accession	Mass	Num. of matches	Description	emPAI
0,81	YKL016C	19797	189	Subunit d of the stator stalk of mitochondrial F1F0 ATP synthase; F1F0 ATP synthase is a large, evolutionarily conserved enzyme complex required for ATP synthesis	6876,57
0,92	YPL271W	6738	135	Epsilon subunit of the F1 sector of mitochondrial F1F0 ATP synthase; which is a large, evolutionarily conserved enzyme complex required for ATP synthesis; F1 translationally regulates ATP6 and ATP8 expression to achieve a balanced output of ATP synt	1208,89
0,78	YDR529C	14602	63	Subunit 7 of ubiquinol cytochrome-c reductase (Complex III); Complex III is a component of the mitochondrial inner membrane electron transport chain; oriented facing the mitochondrial matrix; N-terminus appears to play a role in complex assembly	119,3
0,51	YDR377W	11305	126	Subunit f of the F0 sector of mitochondrial F1F0 ATP synthase; F1F0 ATP synthase is a large, evolutionarily conserved enzyme complex required for ATP synthesis	52,52
0,36	YDL067C	6959	10	Subunit VIIa of cytochrome c oxidase (Complex IV); Complex IV is the terminal member of the mitochondrial inner membrane electron transport chain	16,61
0,55	YDR322C-A	10915	29	Subunit e of mitochondrial F1F0-ATPase; ATPase is a large, evolutionarily conserved enzyme complex required for ATP synthesis; essential for the dimeric and oligomeric state of ATP synthase, which in turn determines the shape of inner membrane crist	12,91
0,47	YML081C-A	6683	25	Subunit of the mitochondrial F1F0 ATP synthase; F1F0 ATP synthase is a large, evolutionarily conserved enzyme complex required for ATP synthesis; termed subunit I or subunit j; does not correspond to known ATP synthase subunits in other organisms	10,06
0,56	YHR001W-A	8587	12	Subunit of the ubiqunol-cytochrome c oxidoreductase complex; this complex comprises part of the mitochondrial respiratory chain; members include Cobp, Rip1p, Cyt1p, Cor1p, Qcr2p, Qcr6p, Qcr7p, Qcr8p, Qcr9p, and Qcr10p and comprises part of the mitoc	9,7
0,56	YJL166W	10967	25	Subunit 8 of ubiquinol cytochrome-c reductase (Complex III); Complex III is a component of the mitochondrial inner membrane electron transport chain; oriented facing the intermembrane space; expression is regulated by Abf1p and Cpf1p	8,34
0,52	YBR120C	18667	21	Mitochondrial protein required for translation of the COB mRNA; forms a complex with Cbp3p that binds to mt ribosomes near the polypeptide tunnel exit and promotes efficient translation of the COB mRNA; Cbp3p-Cbp6p complex also interacts with newly	8,27
0,37	YOL077W-A	7529	14	Subunit k of the mitochondrial F1F0 ATP synthase; F1F0 ATP synthase is a large, evolutionarily conserved enzyme complex required for ATP synthesis; associated only with the dimeric form of ATP synthase	7,46

0,36	YOR020W-A	9612	23	Putative protein of unknown function; conserved in A. gossypii; the authentic, non-tagged protein is detected in highly purified mitochondria in high-throughput studies	7,24
0,55	YKR049C	15778	12	Putative redox protein containing a thioredoxin fold; the authentic, non-tagged protein is detected in highly purified mitochondria in high-throughput studies	7,16
0,38	YDL181W	9864	6	Protein that inhibits ATP hydrolysis by the F1F0-ATP synthase; inhibitory function is enhanced by stabilizing proteins Stf1p and Stf2p; has a calmodulin-binding motif and binds calmodulin in vitro; INH1 has a paralog, STF1, that arose from the whole	6,87
0,67	YDL004W	17056	23	Delta subunit of the central stalk of mitochondrial F1F0 ATP synthase; F1F0 ATP synthase is a large, evolutionarily conserved enzyme complex required for ATP synthesis; F1 translationally regulates ATP6 and ATP8 expression to achieve a balanced outp	5,95
0,51	YGL191W	15058	18	Subunit VIa of cytochrome c oxidase; present in a subclass of cytochrome c oxidase complexes that may have a role in mimimizing generation of reactive oxygen species; not essential for cytochrome c oxidase activity but may modulate activity in respo	5,79
0,58	YPR020W	12959	21	Subunit g of the mitochondrial F1F0 ATP synthase; reversibly phosphorylated on two residues; unphosphorylated form is required for dimerization of the ATP synthase complex, which in turn determines oligomerization of the complex and the shape of inn	5,69
0,41	YER048W-A	11259	6	Cysteine desulfurase (Nfs1p) activator; essential for the formation of the persulfide intermediate at the desulfurase active site during pyridoxal phosphate-dependent desulfuration of cysteine; required for mitochondrial iron-sulfur cluster biosynth	5,21
0,28	YER058W	12319	8	Protein required for assembly of cytochrome c oxidase	4,3
0,11	Q0130	7800	10	F0-ATP synthase subunit c (ATPase-associated proteolipid); encoded on the mitochondrial genome; mutation confers oligomycin resistance; expression is specifically dependent on the nuclear genes AEP1 and AEP2	3,75
0,4	YLR295C	14165	16	Subunit h of the F0 sector of mitochondrial F1F0 ATP synthase; F1F0 ATP synthase is a large, evolutionarily conserved enzyme complex required for ATP synthesis; protein abundance increases in response to DNA replication stress	3,27
0,54	YOR020C	11365	7	Mitochondrial matrix co-chaperonin; inhibits the ATPase activity of Hsp60p, a mitochondrial chaperonin; involved in protein folding and sorting in the mitochondria; 10 kD heat shock protein with similarity to E. coli groES	3,25
0,52	YMR158W	17689	7	Mitochondrial ribosomal protein of the small subunit	3,1
0,34	YDR379C-A	9346	7	Mitochondrial protein involved in assembly of succinate dehydrogenase; has a role in maturation of the Sdh2p subunit; member of the LYR protein family; mutations in human ortholog SDHAF1 are associated with infantile leukoencephalopathy	2,7
0,34	YDR513W	15943	10	Cytoplasmic glutaredoxin; thioltransferase, glutathione-dependent disulfide oxidoreductase involved in maintaining redox state of target proteins, also exhibits glutathione peroxidase activity, expression induced in response to stress; GRX2 has two	2,65
0,5	YNL185C	16705	10	Mitochondrial ribosomal protein of the large subunit	2,47
0,38	YNR037C	10269	3	Mitochondrial ribosomal protein of the small subunit; has similarity to E. coli S19 ribosomal protein	2,28
0,22	YCL057C-A	10401	13	Conserved component of the MICOS complex; MICOS (formerly MINOS or MitOS) is a mitochondrial inner membrane complex that extends into the intermembrane space and has a role in the maintenance of crista junctions, inner membrane architecture, and for	2,24
0,25	YNL070W	6866	4	Component of the TOM (translocase of outer membrane) complex; responsible for recognition and initial import steps for all mitochondrially directed proteins; promotes assembly and stability of the TOM complex	2,21
0,36	YIL065C	17814	5	Protein involved in mitochondrial fission and peroxisome abundance; may have a distinct role in tethering protein aggregates to mitochondria in order to retain them in the mother cell; required for localization of Dnm1p and Mdv1p during mitochondria	2,21
0,48	YMR225C	11469	10	Mitochondrial ribosomal protein of the large subunit; protein abundance increases in response to DNA replication stress	1,93
0,35	YHR010W	15522	6	Ribosomal 60S subunit protein L27A; homologous to mammalian ribosomal protein L27, no bacterial homolog; RPL27A has a paralog, RPL27B, that arose from the whole genome duplication	1,9
0,22	YML073C	19949	5	Ribosomal 60S subunit protein L6A; N-terminally acetylated; binds 5.8S rRNA; homologous to mammalian ribosomal protein L6, no bacterial homolog; RPL6A has a paralog, RPL6B, that arose from the whole genome duplication	1,83
0,3	YOL040C	15992	7	Protein component of the small (40S) ribosomal subunit; homologous to mammalian ribosomal protein S15 and bacterial S19	1,82

0,31	YGR027C	12032	3	Protein component of the small (40S) ribosomal subunit; homologous to mammalian ribosomal protein S25, no bacterial homolog; RPS25A has a paralog, RPS25B, that arose from the whole genome duplication	1,79
0,23	YBR262C	12380	4	Component of the MICOS complex; MICOS (formerly MINOS or MitOS) is a mitochondrial inner membrane complex that extends into the intermembrane space and has a role in the maintenance of crista junctions, inner membrane architecture, and formation of	1,7
0,21	YML026C	17073	7	Protein component of the small (40S) ribosomal subunit; homologous to mammalian ribosomal protein S18 and bacterial S13; RPS18B has a paralog, RPS18A, that arose from the whole genome duplication; protein abundance increases in response to DNA repli	1,64
0,29	YNL052W	17130	6	Subunit Va of cytochrome c oxidase; cytochrome c oxidase is the terminal member of the mitochondrial inner membrane electron transport chain; Cox5Ap is predominantly expressed during aerobic growth while its isoform Vb (Cox5Bp) is expressed during a	1,64
0,25	YDR381C-A	12753	3	Protein of unknown function; localized to the mitochondrial outer membrane	1,63
0,28	YLR167W	17389	5	Fusion protein cleaved to yield ribosomal protein S31 and ubiquitin; ubiquitin may facilitate assembly of the ribosomal protein into ribosomes; interacts genetically with translation factor eIF2B; homologous to mammalian ribosomal protein S27A, no b	1,59
0,21	YKL065W-A	8526	6	Putative protein of unknown function	1,58
0,18	YLR395C	8993	9	Subunit VIII of cytochrome c oxidase (Complex IV); Complex IV is the terminal member of the mitochondrial inner membrane electron transport chain	1,47
0,24	YMR286W	9525	2	Mitochondrial ribosomal protein of the large subunit	1,35
0,23	YBL087C	14556	3	Ribosomal 60S subunit protein L23A; homologous to mammalian ribosomal protein L23 and bacterial L14; RPL23A has a paralog, RPL23B, that arose from the whole genome duplication	1,34
0,18	YBL092W	14762	3	Ribosomal 60S subunit protein L32; overexpression disrupts telomeric silencing; homologous to mammalian ribosomal protein L32, no bacterial homolog	1,31
0,28	YCR028C-A	15377	9	ssDNA-binding protein essential for mitochondrial genome maintenance; involved in mitochondrial DNA replication	1,23
0,25	YOL127W	15748	8	Ribosomal 60S subunit protein L25; primary rRNA-binding ribosomal protein component of large ribosomal subunit; binds to 25S rRNA via a conserved C-terminal motif; homologous to mammalian ribosomal protein L23A and bacterial L23	1,2
0,31	YEL020W-A	10379	4	Essential protein of the mitochondrial intermembrane space; forms a complex with Tim10p (TIM10 complex) that delivers hydrophobic proteins to the TIM22 complex for insertion into the inner membrane	1,19
0,23	YHR005C-A	10482	2	Essential protein of the mitochondrial intermembrane space; forms a complex with Tim9p (TIM10 complex) that delivers hydrophobic proteins to the TIM22 complex for insertion into the inner membrane	1,19
0,29	YDL083C	15838	6	Protein component of the small (40S) ribosomal subunit; homologous to mammalian ribosomal protein S16 and bacterial S9; RPS16B has a paralog, RPS16A, that arose from the whole genome duplication	1,19
0,2	YJL104W	16252	6	Subunit of the import motor (PAM complex); the PAM complex is a component of the Translocase of the Inner Mitochondrial membrane (TIM23 complex); forms a 1:1 subcomplex with Pam18p and inhibits its cochaperone activity; contains a J-like domain	1,15
0,28	YOR286W	16733	3	Protein with rhodanese activity; contains a rhodanese-like domain similar to Rdl1p, Uba4p, Tum1p, and Ych1p; overexpression causes a cell cycle delay; null mutant displays elevated frequency of mitochondrial genome loss	1,1
0,19	YDR025W	17830	4	Protein component of the small (40S) ribosomal subunit; homologous to mammalian ribosomal protein S11 and bacterial S17; N-terminally propionylated in vivo; RPS11A has a paralog, RPS11B, that arose from the whole genome duplication	1,01
0,24	YEL054C	17858	5	Ribosomal 60S subunit protein L12A; rpl12a rpl12b double mutant exhibits slow growth and slow translation; homologous to mammalian ribosomal protein L12 and bacterial L11; RPL12A has a paralog, RPL12B, that arose from the whole genome duplication	1,01
0,24	YPL143W	12147	3	Ribosomal 60S subunit protein L33A; N-terminally acetylated; rpl33a null mutant exhibits slow growth while rpl33a rpl33b double null mutant is inviable; homologous to mammalian ribosomal protein L35A, no bacterial homolog; RPL33A has a paralog, RPL3	0,97

0,17	YBR191W	18277	4	Ribosomal 60S subunit protein L21A; homologous to mammalian ribosomal protein L21, no bacterial homolog; RPL21A has a paralog, RPL21B, that arose from the whole genome duplication	0,97
0,19	YML030W	18558	3	Cytochrome c oxidase subunit; required for assembly of the Complex III-Complex IV supercomplex, and for assembly of Cox13p and Rcf2p into cytochrome c oxidase; similar to Rcf2p, and either Rcf1p or Rcf2p is required for late-stage assembly of the Co	0,96
0,25	YGR215W	12385	4	Mitochondrial ribosomal protein of the small subunit	0,94
0,19	YCR046C	19428	4	Mitochondrial ribosomal protein of the large subunit; required for respiration and for maintenance of the mitochondrial genome	0,9
0,24	YDL075W	12945	2	Ribosomal 60S subunit protein L31A; associates with karyopherin Sxm1p; loss of both Rpl31p and Rpl39p confers lethality; homologous to mammalian ribosomal protein L31, no bacterial homolog; RPL31A has a paralog, RPL31B, that arose from the whole gen	0,88
0,24	YLR061W	13685	2	Ribosomal 60S subunit protein L22A; required for the oxidative stress response in yeast; homologous to mammalian ribosomal protein L22, no bacterial homolog; RPL22A has a paralog, RPL22B, that arose from the whole genome duplication	0,83
0,17	YPL013C	13676	2	Mitochondrial ribosomal protein of the small subunit	0,83
0,21	YIL052C	13816	2	Ribosomal 60S subunit protein L34B; homologous to mammalian ribosomal protein L34, no bacterial homolog; RPL34B has a paralog, RPL34A, that arose from the whole genome duplication	0,82
0,17	YGR034W	14226	3	Ribosomal 60S subunit protein L26B; binds to 5.8S rRNA; non-essential even when paralog is also deleted; deletion has minimal affections on ribosome biosynthesis; homologous to mammalian ribosomal protein L26 and bacterial L24; RPL26B has a paralog,	0,79
0,24	YBL039W-B	6914	1	Putative protein of unknown function	0,79
0,2	YMR256C	6928	1	Subunit VII of cytochrome c oxidase (Complex IV); Complex IV is the terminal member of the mitochondrial inner membrane electron transport chain	0,79
0,11	YMR307C-A	7012	1	Dubious open reading frame; unlikely to encode a functional protein, based on available experimental and comparative sequence data; completely overlaps the verified gene GAS1/YMR307W	0,77
0,16	YCR083W	14606	2	Mitochondrial thioredoxin; highly conserved oxidoreductase required to maintain the redox homeostasis of the cell, forms the mitochondrial thioredoxin system with Trr2p, redox state is maintained by both Trr2p and Glr1p	0,76
0,07	YBR230C	14830	3	Mitochondrial outer membrane receptor for cytosolic ribosomes; integral protein of the outer membrane that interacts with the nascent chain-associated complex (NAC) bound to ribosomes, contributing to co-translational mitochondrial import; interacts	0,74
0,23	YKL003C	15011	8	Mitochondrial ribosomal protein of the small subunit; MRP17 exhibits genetic interactions with PET122, encoding a COX3-specific translational activator	0,74
0,14	YHL001W	15190	5	Ribosomal 60S subunit protein L14B; homologous to mammalian ribosomal protein L14, no bacterial homolog; RPL14B has a paralog, RPL14A, that arose from the whole genome duplication; protein abundance increases in response to DNA replication stress	0,72
0,2	YKL170W	15217	3	Mitochondrial ribosomal protein of the large subunit; appears as two protein spots (YmL34 and YmL38) on two-dimensional SDS gels; protein abundance increases in response to DNA replication stress	0,72
0,16	YIL069C	15319	2	Protein component of the small (40S) ribosomal subunit; homologous to mammalian ribosomal protein S24, no bacterial homolog; RPS24B has a paralog, RPS24A, that arose from the whole genome duplication	0,72
0,17	YDR119W-A	7493	6	Putative protein of unknown function; copurifies with respiratory chain supercomplexes composed of Complex III (ubiquinol-cytochrome c reductase) and Complex IV (cytochrome c oxidase)	0,71
0,16	YKL138C	15556	2	Mitochondrial ribosomal protein of the large subunit	0,7
0,26	YPR063C	15569	2	ER-localized protein of unknown function	0,7
0,14	YOL121C	15907	5	Protein component of the small (40S) ribosomal subunit; required for assembly and maturation of pre-40 S particles; homologous to mammalian ribosomal protein S19, no bacterial homolog; mutations in human RPS19 are associated with Diamond Blackfan an	0,68
0,18	YIL051C	15944	2	Mitochondrial protein required for transamination of isoleucine; but not of valine or leucine; may regulate specificity of branched-chain transaminases Bat1p and Bat2p; induction of expression in response to stress is mediated by a Hog1p-regulated a	0,68

0,17	YPR100W	16251	3	Mitochondrial ribosomal protein of the large subunit	0,67
0,11	YPR010C-A	7943	1	Putative protein of unknown function; conserved among Saccharomyces sensu stricto species	0,66
0,13	YML009C	7968	1	Mitochondrial ribosomal protein of the large subunit	0,66
0,17	YOR298C-A	16394	2	Transcriptional coactivator; bridges the DNA-binding region of Gcn4p and TATA-binding protein Spt15p; suppressor of frameshift mutations; protein abundance increases in response to DNA replication stress	0,66
0,14	YCR071C	16554	2	Mitochondrial ribosomal protein of the large subunit; conserved in metazoa, with similarity to human mitochondrial ribosomal protein MRPL49	0,65
0,24	YNL131W	16780	2	Component of the TOM (Translocase of Outer Membrane) complex; responsible for initial import of mitochondrially directed proteins; mediates interaction between TOM and TIM complexes and acts as a receptor for precursor proteins	0,64
0,14	YIL156W-B	8251	1	Putative protein of unknown function; originally identified based on homology to <i>Ashbya gossypii</i> and other related yeasts	0,64
0,1	YNL024C-A	8328	1	Essential protein suggested to function early in the secretory pathway; inviability is suppressed by overexpression of Golgi protein Tvp23p; ortholog of human Kish	0,63
0,19	YIL087C	16913	2	Putative protein of unknown function; mitochondrial protein that physically interacts with Tim23p; null mutant displays reduced respiratory growth	0,63
0,15	YDR462W	17377	3	Mitochondrial ribosomal protein of the large subunit; protein abundance increases in response to DNA replication stress	0,61
0,19	YHR051W	17377	2	Subunit VI of cytochrome c oxidase (Complex IV); Complex IV is the terminal member of the mitochondrial inner membrane electron transport chain; expression is regulated by oxygen levels	0,61
0,12	YER087C-B	8706	1	Beta subunit of Sec61p ER translocation complex (Sec61p-Sss1p-Sbh1p); involved in protein translocation into the endoplasmic reticulum; interacts with the exocyst complex and also with Rtn1p; cotranslationally N-acetylated by NatA; SBH1 has a paralo	0,6
0,12	YGL031C	17603	3	Ribosomal 60S subunit protein L24A; not essential for translation but may be required for normal translation rate; homologous to mammalian ribosomal protein L24, no bacterial homolog; RPL24A has a paralog, RPL24B, that arose from the whole genome du	0,6
0,2	YLR008C	17899	7	Subunit of the import motor (PAM complex); the PAM complex is a component of the Translocase of the Inner Mitochondrial membrane (TIM23 complex); essential J-protein cochaperone that stimulates Ssc1p ATPase activity to drive import; inhibited by Pa	0,59
0,12	YDR086C	8984	1	Subunit of the Sec61p translocation complex (Sec61p-Sss1p-Sbh1p); this complex forms a channel for passage of secretory proteins through the endoplasmic reticulum membrane, and of the Ssh1p complex (Ssh1p-Sbh2p-Sss1p); interacts with Ost4p and Wbp1p	0,57
0,17	YJL096W	18376	7	Mitochondrial ribosomal protein of the large subunit	0,57
0,13	YOR150W	18544	3	Mitochondrial ribosomal protein of the large subunit; localizes to vacuole in response to H2O2	0,57
0,18	YLR164W	18758	3	Putative alternate subunit of succinate dehydrogenase (SDH); mitochondrial inner membrane protein; genetic interaction with SDH4 suggests that Shh4p can function as a functional SDH subunit; a fraction copurifies with SDH subunit Sdh3p; expression i	0,56
0,15	YER019C-A	9600	2	Ssh1p-Sss1p-Sbh2p complex component; involved in protein translocation into the endoplasmic reticulum; SBH2 has a paralog, SBH1, that arose from the whole genome duplication	0,53
0,16	YGR085C	19783	4	Ribosomal 60S subunit protein L11B; expressed at half the level of Rpl11Ap; involved in ribosomal assembly; depletion causes degradation of 60S proteins and RNA; homologous to mammalian ribosomal protein L11 and bacterial L5; RPL11B has a paralog, R	0,52
0,26	YJL062W-A	9875	3	Mitochondrial protein required for cytochrome c oxidase assembly; also involved in translational regulation of Cox1p and prevention of Cox1p aggregation before assembly; located in the mitochondrial inner membrane	0,51
0,22	YDL130W-A	10102	2	Protein involved in regulation of the mitochondrial F1F0-ATP synthase; Stf1p and Stf2p act as stabilizing factors that enhance inhibitory action of the Inh1p protein; protein abundance increases in response to DNA replication stress; STF1 has a para	0,5
0,09	YPR043W	10314	1	Ribosomal 60S subunit protein L43A; null mutation confers a dominant lethal phenotype; homologous to mammalian ribosomal protein L37A, no bacterial homolog; RPL43A has a paralog, RPL43B, that arose from the whole genome duplication	0,49

0,1	YNL211C	10642	4	Putative protein of unknown function; green fluorescent protein (GFP)-fusion protein localizes to mitochondria; YNL211C is not an essential gene	0,47
0,14	YMR194W	11117	1	Ribosomal 60S subunit protein L36A; N-terminally acetylated; binds to 5.8 S rRNA; homologous to mammalian ribosomal protein L36, no bacterial homolog; RPL36A has a paralog, RPL36B, that arose from the whole genome duplication	0,45
0,12	YBR009C	11361	1	Histone H4; core histone protein required for chromatin assembly and chromosome function; one of two identical histone proteins (see also HHF2); contributes to telomeric silencing; N-terminal domain involved in maintaining genomic integrity	0,44
0,22	YJR085C	11243	1	Protein of unknown function; GFP-fusion protein is induced in response to the DNA-damaging agent MMS; the authentic, non-tagged protein is detected in highly purified mitochondria in high-throughput studies; protein abundance increases in response t	0,44
0,1	YBL029C-A	11383	1	Protein of unknown function; green fluorescent protein (GFP)-fusion protein localizes to the cell periphery; protein abundance increases in response to DNA replication stress; has potential orthologs in Saccharomyces species and in Yarrowia lipolyti	0,43
0,16	YJR048W	12312	2	Cytochrome c, isoform 1; also known as iso-1-cytochrome c; electron carrier of the mitochondrial intermembrane space that transfers electrons from ubiquinone-cytochrome c oxidoreductase to cytochrome c oxidase during cellular respiration; mutations	0,4
0,15	YAL044W-A	12575	2	Putative protein of unknown function; similar to S. pombe uvi31 which is a putative DNA repair protein	0,39
0,15	YJR086W	12755	1	G protein gamma subunit; forms a dimer with Ste4p to activate the mating signaling pathway, forms a heterotrimer with Gpa1p and Ste4p to dampen signaling; C-terminus is palmitoylated and farnesylated, which are required for normal signaling	0,38
0,17	YMR230W	12731	1	Protein component of the small (40S) ribosomal subunit; homologous to mammalian ribosomal protein S10, no bacterial homolog; RPS10B has a paralog, RPS10A, that arose from the whole genome duplication	0,38
0,06	YDR079W	13225	1	Chaperone that facilitates the assembly of cytochrome c oxidase; integral to the mitochondrial inner membrane; interacts with a subcomplex of subunits VII, VIIa, and VIII (Cox7p, Cox9p, and Cox8p) but not with the holoenzyme	0,37
0,08	YDL157C	13646	1	Putative protein of unknown function; the authentic, non-tagged protein is detected in highly purified mitochondria in high-throughput studies	0,35
0,12	YHL015W	13899	1	Protein component of the small (40S) ribosomal subunit; overproduction suppresses mutations affecting RNA polymerase III-dependent transcription; homologous to mammalian ribosomal protein S20 and bacterial S10	0,35
0,1	YFR049W	13772	2	Subunit of the mitochondrial alpha-ketoglutarate dehydrogenase; recruits E3 subunit (Lpd1p) to the E1-E2 (Kgd1p, Kgd2p) core; has similarity to human mitochondrial ribosomal protein MRP-S36	0,35
0,13	YDR493W	14010	1	Protein required for assembly of the cytochrome bc(1) complex; acts as a chaperone for Rip1p and facilitates its insertion into the complex at a late stage of assembly; localized to the mitochondrial matrix; null mutant exhibits a respiratory growth	0,34
0,19	YDL136W	13947	2	Ribosomal 60S subunit protein L35B; homologous to mammalian ribosomal protein L35 and bacterial L29; RPL35B has a paralog, RPL35A, that arose from the whole genome duplication	0,34
0,07	YBL002W	14229	1	Histone H2B; core histone protein required for chromatin assembly and chromosome function; nearly identical to HTB1; Rad6p-Bre1p-Lge1p mediated ubiquitination regulates reassembly after DNA replication, transcriptional activation, meiotic DSB format	0,34
0,13	YCR031C	14574	1	Protein component of the small (40S) ribosomal subunit; required for ribosome assembly and 20S pre-rRNA processing; mutations confer cryptopleurine resistance; homologous to mammalian ribosomal protein S14 and bacterial S11; RPS14A has a paralog, RP	0,33
0,05	YJL190C	14663	1	Protein component of the small (40S) ribosomal subunit; homologous to mammalian ribosomal protein S15A and bacterial S8; RPS22A has a paralog, RPS22B, that arose from the whole genome duplication	0,33
0,06	YGL080W	15032	1	Highly conserved subunit of the mitochondrial pyruvate carrier; a mitochondrial inner membrane complex comprised of Mpc1p and either Mpc2p or Mpc3p mediates mitochondrial pyruvate uptake; null mutant displays slow growth that is complemented by expr	0,32
0,07	YOR369C	15462	1	Protein component of the small (40S) ribosomal subunit; homologous to mammalian ribosomal protein S12, no bacterial homolog	0,31
0,07	YBR010W	15347	2	Histone H3; core histone protein required for chromatin assembly, part of heterochromatin-mediated telomeric and HM silencing; one of two identical histone H3 proteins (see HHT2); regulated by acetylation, methylation, and phosphorylation; H3K14 ace	0,31

0,08	YER093C-A	15789	1	Protein of unknown function; null mutant is viable but shows increased loss of mitochondrial genome and synthetic interaction with prohibitin (phb1); contains an intron; YER093C-A has a paralog, YBL059W, that arose from the whole genome duplication	0,3
0,09	YER050C	15871	1	Mitochondrial ribosomal protein of the small subunit; has similarity to E. coli S18 ribosomal protein	0,3
0,08	YNL081C	16171	1	Putative mitochondrial ribosomal protein of the small subunit; has similarity to E. coli S13 ribosomal protein; participates in controlling sporulation efficiency; localizes to vacuole in response to H2O2	0,29
0,08	YGR243W	16266	1	Highly conserved subunit of mitochondrial pyruvate carrier; more highly expressed in glucose-containing minimal medium than in lactate-containing medium; expression regulated by osmotic and alkaline stresses; protein abundance increases in response	0,29
0,09	YNR022C	16365	1	Mitochondrial ribosomal protein of the large subunit; not essential for mitochondrial translation	0,29
0,06	YNL328C	16464	1	Constituent of the mitochondrial import motor; associated with the presequence translocase; function overlaps with that of Pam18p; stimulates the ATPase activity of Ssc1p to drive mitochondrial import; contains a J domain	0,29
0,08	YIL040W	16589	1	Protein required for nuclear envelope morphology; nuclear pore complex localization, mRNA export from the nucleus; exhibits synthetic lethal genetic interactions with genes involved in lipid metabolism	0,28
0,13	YPL037C	17010	1	Subunit beta1 of the nascent polypeptide-associated complex (NAC); involved in protein targeting, associated with cytoplasmic ribosomes; enhances DNA binding of the Gal4p activator; homolog of human BTF3b; EGD1 has a paralog, BTT1, that arose from t	0,28
0,09	YBR282W	16528	1	Mitochondrial ribosomal protein of the large subunit; homolog of human Bcl-2 interacting protein BMRP	0,28
0,06	YGL103W	16711	2	Ribosomal 60S subunit protein L28; homologous to mammalian ribosomal protein L27A and bacterial L15; may have peptidyl transferase activity; can mutate to cycloheximide resistance	0,28
0,08	YDR064W	17018	1	Protein component of the small (40S) ribosomal subunit; homologous to mammalian ribosomal protein S13 and bacterial S15	0,28
0,07	YNR036C	17090	1	Mitochondrial protein; may interact with ribosomes based on co-purification experiments; similar to E. coli and human mitochondrial S12 ribosomal proteins	0,27
0,08	YFR033C	17339	4	Subunit 6 of the ubiquinol cytochrome-c reductase complex; the complex, also known as the cytochrome bc(1) complex or Complex III, is a component of the mitochondrial inner membrane electron transport chain; highly acidic protein; required for matur	0,27
0,06	YPR098C	17857	1	Protein of unknown function; localized to the mitochondrial outer membrane	0,26
0,07	YPL135W	18021	1	Conserved protein of the mitochondrial matrix; performs a scaffolding function during assembly of iron-sulfur clusters, interacts physically and functionally with yeast frataxin (Yfh1p); isu1 isu2 double mutant is inviable; ISU1 has a paralog, ISU2,	0,26
0,08	YIR037W	18768	1	Thiol peroxidase; functions as a hydroperoxide receptor to sense intracellular hydroperoxide levels and transduce a redox signal to the Yap1p transcription factor; HYR1 has a paralog, GPX1, that arose from the whole genome duplication	0,25
0,11	YIL016W	18341	2	Ribosome-associated protein; proposed to act in protein synthesis and nuclear pore complex biogenesis and maintenance as well as protein folding; has similarity to the mammalian BAG-1 protein	0,25
0,06	YFR011C	18936	1	Component of the MICOS complex; MICOS (formerly MINOS or MitOS) is a mitochondrial inner membrane complex that extends into the intermembrane space and has a role in the maintenance of crista junctions, inner membrane architecture, and formation of	0,24
0,12	YPR149W	19185	4	Protein of unknown function; contains transmembrane domains; involved in secretion of proteins that lack classical secretory signal sequences; component of the detergent-insoluble glycolipid-enriched complexes (DIGs); NCE102 has a paralog, FHN1, tha	0,24
0,07	YML078W	19998	1	Mitochondrial peptidyl-prolyl cis-trans isomerase (cyclophilin); catalyzes the cis-trans isomerization of peptide bonds N-terminal to proline residues; involved in protein refolding after import into mitochondria	0,23

BAND 18

Sequence coverage	Accession	Mass	Num. of matches	Description	emPAI
0,71	YML081C-A	6683	27	Subunit of the mitochondrial F1F0 ATP synthase; F1F0 ATP synthase is a large, evolutionarily conserved enzyme complex required for ATP synthesis; termed subunit I or subunit j; does not correspond to known ATP synthase subunits in other organisms	35,78

0,92	YPL271W	6738	90	Epsilon subunit of the F1 sector of mitochondrial F1F0 ATP synthase; which is a large, evolutionarily conserved enzyme complex required for ATP synthesis; F1 translationally regulates ATP6 and ATP8 expression to achieve a balanced output of ATP synt	668,65
0,25	YNL070W	6866	4	Component of the TOM (translocase of outer membrane) complex; responsible for recognition and initial import steps for all mitochondrially directed proteins; promotes assembly and stability of the TOM complex	2,21
0,24	YBL039W-B	6914	1	Putative protein of unknown function	0,79
0,2	YMR256C	6928	5	Subunit VII of cytochrome c oxidase (Complex IV); Complex IV is the terminal member of the mitochondrial inner membrane electron transport chain	2,21
0,36	YDL067C	6959	27	Subunit VIIa of cytochrome c oxidase (Complex IV); Complex IV is the terminal member of the mitochondrial inner membrane electron transport chain	54,49
0,11	YMR307C-A	7012	1	Dubious open reading frame; unlikely to encode a functional protein, based on available experimental and comparative sequence data; completely overlaps the verified gene GAS1/YMR307W	0,77
0,17	YDR119W-A	7493	3	Putative protein of unknown function; copurifies with respiratory chain supercomplexes composed of Complex III (ubiquinol-cytochrome c reductase) and Complex IV (cytochrome c oxidase)	0,71
0,81	YOL077W-A	7529	45	Subunit k of the mitochondrial F1F0 ATP synthase; F1F0 ATP synthase is a large, evolutionarily conserved enzyme complex required for ATP synthesis; associated only with the dimeric form of ATP synthase	120,82
0,11	Q0130	7800	11	F0-ATP synthase subunit c (ATPase-associated proteolipid); encoded on the mitochondrial genome; mutation confers oligomycin resistance; expression is specifically dependent on the nuclear genes AEP1 and AEP2	1,82
0,26	YML129C	7954	1	Mitochondrial cytochrome c oxidase (complex IV) assembly factor; also involved in translational regulation of Cox1p and prevention of Cox1p aggregation before assembly; associates with complex IV assembly intermediates and complex III/complex IV sup	0,66
0,13	YML009C	7968	1	Mitochondrial ribosomal protein of the large subunit	0,66
0,14	YIL156W-B	8251	4	Putative protein of unknown function; originally identified based on homology to <i>Ashbya gossypii</i> and other related yeasts	0,64
0,1	YNL024C-A	8328	1	Essential protein suggested to function early in the secretory pathway; inviability is suppressed by overexpression of Golgi protein Tvp23p; ortholog of human Kish	0,63
0,15	YKL023C-A	8514	1	Putative protein of unknown function	0,61
0,21	YKL065W-A	8526	6	Putative protein of unknown function	1,58
0,56	YHR001W-A	8587	21	Subunit of the ubiqunol-cytochrome c oxidoreductase complex; this complex comprises part of the mitochondrial respiratory chain; members include Cobp, Rip1p, Cyt1p, Cor1p, Qcr2p, Qcr6p, Qcr7p, Qcr8p, Qcr9p, and Qcr10p and comprises part of the mitoc	26,6
0,1	YDR086C	8984	1	Subunit of the Sec61p translocation complex (Sec61p-Sss1p-Sbh1p); this complex forms a channel for passage of secretory proteins through the endoplasmic reticulum membrane, and of the Ssh1p complex (Ssh1p-Sbh2p-Sss1p); interacts with Ost4p and Wbp1p	0,57
0,18	YLR395C	8993	8	Subunit VIII of cytochrome c oxidase (Complex IV); Complex IV is the terminal member of the mitochondrial inner membrane electron transport chain	1,47
0,14	YDR379C-A	9346	1	Mitochondrial protein involved in assembly of succinate dehydrogenase; has a role in maturation of the Sdh2p subunit; member of the LYR protein family; mutations in human ortholog SDHAF1 are associated with infantile leukoencephalopathy	0,55
0,13	YMR286W	9525	1	Mitochondrial ribosomal protein of the large subunit	0,53
0,23	YGL226C-A	9557	6	Zeta subunit of the oligosaccharyltransferase complex of the ER lumen; complex catalyzes asparagine-linked glycosylation of newly synthesized proteins	1,35
0,26	YER019C-A	9600	2	Ssh1p-Sss1p-Sbh2p complex component; involved in protein translocation into the endoplasmic reticulum; SBH2 has a paralog, SBH1, that arose from the whole genome duplication	1,35

0,36	YOR020W-A	9612	8	Putative protein of unknown function; conserved in A. gossypii; the authentic, non-tagged protein is detected in highly purified mitochondria in high-throughput studies	2,54
0,53	YDL181W	9864	9	Protein that inhibits ATP hydrolysis by the F1F0-ATP synthase; inhibitory function is enhanced by stabilizing proteins Stf1p and Stf2p; has a calmodulin-binding motif and binds calmodulin in vitro; INH1 has a paralog, STF1, that arose from the whole	6,87
0,26	YJL062W-A	9875	5	Mitochondrial protein required for cytochrome c oxidase assembly; also involved in translational regulation of Cox1p and prevention of Cox1p aggregation before assembly; located in the mitochondrial inner membrane	0,51
0,19	YLR038C	9965	1	Subunit VIb of cytochrome c oxidase; cytochrome c oxidase is also known as respiratory Complex IV and is the terminal member of the mitochondrial inner membrane electron transport chain; required for assembly of cytochrome c oxidase but not required	0,5
0,18	YPR043W	10314	2	Ribosomal 60S subunit protein L43A; null mutation confers a dominant lethal phenotype; homologous to mammalian ribosomal protein L37A, no bacterial homolog; RPL43A has a paralog, RPL43B, that arose from the whole genome duplication	1,21
0,43	YCL057C-A	10401	15	Conserved component of the MICOS complex; MICOS (formerly MINOS or MitOS) is a mitochondrial inner membrane complex that extends into the intermembrane space and has a role in the maintenance of crista junctions, inner membrane architecture, and for	3,78
0,12	YGR236C	10538	1	Protein required for high temperature survival during stationary phase; not required for growth on nonfermentable carbon sources; the authentic, non-tagged protein is detected in highly purified mitochondria in high-throughput studies	0,47
0,1	YNL211C	10642	3	Putative protein of unknown function; green fluorescent protein (GFP)-fusion protein localizes to mitochondria; YNL211C is not an essential gene	0,47
0,89	YDR322C-A	10915	117	Subunit e of mitochondrial F1F0-ATPase; ATPase is a large, evolutionarily conserved enzyme complex required for ATP synthesis; essential for the dimeric and oligomeric state of ATP synthase, which in turn determines the shape of inner membrane crist	1263,49
0,65	YJL166W	10967	21	Subunit 8 of ubiquinol cytochrome-c reductase (Complex III); Complex III is a component of the mitochondrial inner membrane electron transport chain; oriented facing the intermembrane space; expression is regulated by Abf1p and Cpf1p	12,54
0,14	YMR194W	11117	1	Ribosomal 60S subunit protein L36A; N-terminally acetylated; binds to 5.8 S rRNA; homologous to mammalian ribosomal protein L36, no bacterial homolog; RPL36A has a paralog, RPL36B, that arose from the whole genome duplication	0,45
0,22	YJR085C	11243	1	Protein of unknown function; GFP-fusion protein is induced in response to the DNA-damaging agent MMS; the authentic, non-tagged protein is detected in highly purified mitochondria in high-throughput studies; protein abundance increases in response t	0,44
0,57	YER048W-A	11259	12	Cysteine desulfurase (Nfs1p) activator; essential for the formation of the persulfide intermediate at the desulfurase active site during pyridoxal phosphate-dependent desulfuration of cysteine; required for mitochondrial iron-sulfur cluster biosynth	7,94
0,56	YDR377W	11305	106	Subunit f of the F0 sector of mitochondrial F1F0 ATP synthase; F1F0 ATP synthase is a large, evolutionarily conserved enzyme complex required for ATP synthesis	157,12
0,1	YBL029C-A	11383	1	Protein of unknown function; green fluorescent protein (GFP)-fusion protein localizes to the cell periphery; protein abundance increases in response to DNA replication stress; has potential orthologs in Saccharomyces species and in Yarrowia lipolyti	0,43
0,48	YMR225C	11469	4	Mitochondrial ribosomal protein of the large subunit; protein abundance increases in response to DNA replication stress	1,93
0,1	YKL018C-A	11849	2	Putative protein of unknown function; identified by homology; green fluorescent protein (GFP)-fusion protein localizes to the cytoplasm	0,99
0,06	YHR087W	12002	1	Protein of unknown function involved in RNA metabolism; has structural similarity to SBDS, the human protein mutated in Shwachman-Diamond Syndrome (the yeast SBDS ortholog = SDO1); null mutation suppresses cdc13-1 temperature sensitivity; protein ab	0,41
0,21	YGR027C	12032	3	Protein component of the small (40S) ribosomal subunit; homologous to mammalian ribosomal protein S25, no bacterial homolog; RPS25A has a paralog, RPS25B, that arose from the whole genome duplication	0,98
0,17	YDR115W	12069	2	Putative mitochondrial ribosomal protein of the large subunit; similar to E. coli L34 ribosomal protein; required for respiratory growth, as are most mitochondrial ribosomal proteins; protein increases in abundance and relocalizes to the plasma memb	0,97
0,24	YPL143W	12147	2	Ribosomal 60S subunit protein L33A; N-terminally acetylated; rpl33a null mutant exhibits slow growth while rpl33a rpl33b double null mutant is inviable; homologous to mammalian ribosomal protein L35A, no bacterial homolog; RPL33A has a paralog, RPL3	0,97

0,5	YJR048W	12312	22	Cytochrome c, isoform 1; also known as iso-1-cytochrome c; electron carrier of the mitochondrial intermembrane space that transfers electrons from ubiquinone-cytochrome c oxidoreductase to cytochrome c oxidase during cellular respiration; mutations	19,07
0,28	YER058W	12319	5	Protein required for assembly of cytochrome c oxidase	2,79
0,39	YGR215W	12385	4	Mitochondrial ribosomal protein of the small subunit	1,69
0,15	YAL044W-A	12575	1	Putative protein of unknown function; similar to S. pombe uvi31 which is a putative DNA repair protein	0,39
0,12	YOL109W	12582	1	Peripheral membrane protein of the plasma membrane; interacts with Mid2p; regulates the cell integrity pathway mediated by Pkc1p and Slt2p; the authentic protein is detected in a phosphorylated state in highly purified mitochondria	0,39
0,17	YMR230W	12731	1	Protein component of the small (40S) ribosomal subunit; homologous to mammalian ribosomal protein S10, no bacterial homolog; RPS10B has a paralog, RPS10A, that arose from the whole genome duplication	0,38
0,24	YDR381C-A	12753	2	Protein of unknown function; localized to the mitochondrial outer membrane	0,9
0,07	YDL075W	12945	1	Ribosomal 60S subunit protein L31A; associates with karyopherin Sxm1p; loss of both Rpl31p and Rpl39p confers lethality; homologous to mammalian ribosomal protein L31, no bacterial homolog; RPL31A has a paralog, RPL31B, that arose from the whole gen	0,37
0,87	YPR020W	12959	64	Subunit g of the mitochondrial F1F0 ATP synthase; reversibly phosphorylated on two residues; unphosphorylated form is required for dimerization of the ATP synthase complex, which in turn determines oligomerization of the complex and the shape of inn	297,79
0,06	YDR079W	13225	1	Chaperone that facilitates the assembly of cytochrome c oxidase; integral to the mitochondrial inner membrane; interacts with a subcomplex of subunits VII, VIIa, and VIII (Cox7p, Cox9p, and Cox8p) but not with the holoenzyme	0,37
0,1	YPR166C	13621	1	Mitochondrial ribosomal protein of the small subunit	0,35
0,35	YPL013C	13676	3	Mitochondrial ribosomal protein of the small subunit	1,47
0,24	YFR049W	13772	5	Subunit of the mitochondrial alpha-ketoglutarate dehydrogenase; recruits E3 subunit (Lpd1p) to the E1-E2 (Kgd1p, Kgd2p) core; has similarity to human mitochondrial ribosomal protein MRP-S36	1,45
0,21	YIL052C	13816	2	Ribosomal 60S subunit protein L34B; homologous to mammalian ribosomal protein L34, no bacterial homolog; RPL34B has a paralog, RPL34A, that arose from the whole genome duplication	0,82
0,21	YBR255C-A	13824	2	Putative protein of unknown function; may interact with respiratory chain complexes III (ubiquinol-cytochrome c reductase) or IV (cytochrome c oxidase); identified by sequence comparison with hemiascomycetous yeast species	0,81
0,07	YDL136W	13947	2	Ribosomal 60S subunit protein L35B; homologous to mammalian ribosomal protein L35 and bacterial L29; RPL35B has a paralog, RPL35A, that arose from the whole genome duplication	0,34
0,05	YBL003C	13981	2	Histone H2A; core histone protein required for chromatin assembly and chromosome function; one of two nearly identical (see also HTA1) subtypes; DNA damage-dependent phosphorylation by Mec1p facilitates DNA repair; acetylated by Nat4p	0,34
0,19	YHL018W	14063	2	Putative 4a-hydroxytetrahydrobiopterin dehydratase; green fluorescent protein (GFP)-fusion protein localizes to mitochondria and is induced in response to the DNA-damaging agent MMS	0,79
0,11	YLR295C	14165	7	Subunit h of the F0 sector of mitochondrial F1F0 ATP synthase; F1F0 ATP synthase is a large, evolutionarily conserved enzyme complex required for ATP synthesis; protein abundance increases in response to DNA replication stress	1,39
0,28	YLR344W	14225	4	Ribosomal 60S subunit protein L26A; binds to 5.8S rRNA; non-essential even when paralog is also deleted; deletion has minimal affections on ribosome biosynthesis; homologous to mammalian ribosomal protein L26 and bacterial L24; RPL26A has a paralog,	2,19
0,15	YBL002W	14229	5	Histone H2B; core histone protein required for chromatin assembly and chromosome function; nearly identical to HTB1; Rad6p-Bre1p-Lge1p mediated ubiquitination regulates reassembly after DNA replication, transcriptional activation, meiotic DSB format	1,39

0,31	YBL087C	14556	6	Ribosomal 60S subunit protein L23A; homologous to mammalian ribosomal protein L23 and bacterial L14; RPL23A has a paralog, RPL23B, that arose from the whole genome duplication	1,34
0,08	YCR031C	14574	1	Protein component of the small (40S) ribosomal subunit; required for ribosome assembly and 20S pre-rRNA processing; mutations confer cryptopleurine resistance; homologous to mammalian ribosomal protein S14 and bacterial S11; RPS14A has a paralog, RP	0,33
0,87	YDR529C	14602	60	Subunit 7 of ubiquinol cytochrome-c reductase (Complex III); Complex III is a component of the mitochondrial inner membrane electron transport chain; oriented facing the mitochondrial matrix; N-terminus appears to play a role in complex assembly	118,97
0,31	YCR083W	14606	7	Mitochondrial thioredoxin; highly conserved oxidoreductase required to maintain the redox homeostasis of the cell, forms the mitochondrial thioredoxin system with Trr2p, redox state is maintained by both Trr2p and Glr1p	2,08
0,23	YJL190C	14663	3	Protein component of the small (40S) ribosomal subunit; homologous to mammalian ribosomal protein S15A and bacterial S8; RPS22A has a paralog, RPS22B, that arose from the whole genome duplication	0,76
0,18	YBL092W	14762	3	Ribosomal 60S subunit protein L32; overexpression disrupts telomeric silencing; homologous to mammalian ribosomal protein L32, no bacterial homolog	0,75
0,37	YKR094C	14775	10	Ubiquitin-ribosomal 60S subunit protein L40B fusion protein; cleaved to yield ubiquitin and ribosomal protein L40B; ubiquitin may facilitate assembly of the ribosomal protein into ribosomes; homologous to mammalian ribosomal protein L40, no bacteria	2,06
0,06	YBR230C	14830	4	Mitochondrial outer membrane receptor for cytosolic ribosomes; integral protein of the outer membrane that interacts with the nascent chain-associated complex (NAC) bound to ribosomes, contributing to co-translational mitochondrial import; interacts	0,32
0,07	YBR016W	14837	1	Tail-anchored plasma membrane protein with a conserved CYSTM module; predicted to be palmitoylated; has similarity to hydrophilins, which are involved in the adaptive response to hyperosmotic conditions; YBR016W has a paralog, YDL012C, that arose fr	0,32
0,22	YOR103C	14918	2	Epsilon subunit of the oligosaccharyltransferase complex; located in the ER lumen; catalyzes asparagine-linked glycosylation of newly synthesized proteins	0,74
0,23	YKL003C	15011	10	Mitochondrial ribosomal protein of the small subunit; MRP17 exhibits genetic interactions with PET122, encoding a COX3-specific translational activator	0,74
0,43	YGL191W	15058	25	Subunit VIa of cytochrome c oxidase; present in a subclass of cytochrome c oxidase complexes that may have a role in mimimizing generation of reactive oxygen species; not essential for cytochrome c oxidase activity but may modulate activity in respo	2,93
0,25	YHL001W	15190	7	Ribosomal 60S subunit protein L14B; homologous to mammalian ribosomal protein L14, no bacterial homolog; RPL14B has a paralog, RPL14A, that arose from the whole genome duplication; protein abundance increases in response to DNA replication stress	1,96
0,25	YKL170W	15217	4	Mitochondrial ribosomal protein of the large subunit; appears as two protein spots (YmL34 and YmL38) on two-dimensional SDS gels; protein abundance increases in response to DNA replication stress	1,26
0,38	YIL069C	15319	5	Protein component of the small (40S) ribosomal subunit; homologous to mammalian ribosomal protein S24, no bacterial homolog; RPS24B has a paralog, RPS24A, that arose from the whole genome duplication	2,85
0,07	YBR010W	15347	1	Histone H3; core histone protein required for chromatin assembly, part of heterochromatin-mediated telomeric and HM silencing; one of two identical histone H3 proteins (see HHT2); regulated by acetylation, methylation, and phosphorylation; H3K14 ace	0,31
0,35	YCR028C-A	15377	11	ssDNA-binding protein essential for mitochondrial genome maintenance; involved in mitochondrial DNA replication	2,81
0,26	YHR010W	15522	5	Ribosomal 60S subunit protein L27A; homologous to mammalian ribosomal protein L27, no bacterial homolog; RPL27A has a paralog, RPL27B, that arose from the whole genome duplication	1,22
0,08	YKL138C	15556	1	Mitochondrial ribosomal protein of the large subunit	0,3
0,09	YPR063C	15569	1	ER-localized protein of unknown function	0,3
0,25	YOL127W	15748	5	Ribosomal 60S subunit protein L25; primary rRNA-binding ribosomal protein component of large ribosomal subunit; binds to 25S rRNA via a conserved C-terminal motif; homologous to mammalian ribosomal protein L23A and bacterial L23	1,2
0,38	YKR049C	15778	5	Putative redox protein containing a thioredoxin fold; the authentic, non-tagged protein is detected in highly purified mitochondria in high-throughput studies	1,85

0,16	YML024W	15825	2	Ribosomal protein 51 (rp51) of the small (40s) subunit; homologous to mammalian ribosomal protein S17, no bacterial homolog; RPS17A has a paralog, RPS17B, that arose from the whole genome duplication	0,68
0,27	YDL083C	15838	6	Protein component of the small (40S) ribosomal subunit; homologous to mammalian ribosomal protein S16 and bacterial S9; RPS16B has a paralog, RPS16A, that arose from the whole genome duplication	1,18
0,14	YER050C	15871	4	Mitochondrial ribosomal protein of the small subunit; has similarity to E. coli S18 ribosomal protein	0,68
0,26	YOL121C	15907	5	Protein component of the small (40S) ribosomal subunit; required for assembly and maturation of pre-40 S particles; homologous to mammalian ribosomal protein S19, no bacterial homolog; mutations in human RPS19 are associated with Diamond Blackfan an	1,83
0,1	YIL051C	15944	1	Mitochondrial protein required for transamination of isoleucine; but not of valine or leucine; may regulate specificity of branched-chain transaminases Bat1p and Bat2p; induction of expression in response to stress is mediated by a Hog1p-regulated a	0,3
0,23	YOL040C	15992	4	Protein component of the small (40S) ribosomal subunit; homologous to mammalian ribosomal protein S15 and bacterial S19	0,68
0,13	YKL167C	16056	1	Mitochondrial ribosomal protein of the large subunit; not essential for mitochondrial translation	0,29
0,05	YGR118W	16120	1	Ribosomal protein 28 (rp28) of the small (40S) ribosomal subunit; required for translational accuracy; homologous to mammalian ribosomal protein S23 and bacterial S12; RPS23A has a paralog, RPS23B, that arose from the whole genome duplication; delet	0,29
0,1	YPR100W	16251	2	Mitochondrial ribosomal protein of the large subunit	0,67
0,47	YJL104W	16252	20	Subunit of the import motor (PAM complex); the PAM complex is a component of the Translocase of the Inner Mitochondrial membrane (TIM23 complex); forms a 1:1 subcomplex with Pam18p and inhibits its cochaperone activity; contains a J-like domain	6,71
0,08	YOR298C-A	16394	1	Transcriptional coactivator; bridges the DNA-binding region of Gcn4p and TATA-binding protein Spt15p; suppressor of frameshift mutations; protein abundance increases in response to DNA replication stress	0,29
0,09	YBR282W	16528	1	Mitochondrial ribosomal protein of the large subunit; homolog of human Bcl-2 interacting protein BMRP	0,28
0,14	YCR071C	16554	3	Mitochondrial ribosomal protein of the large subunit; conserved in metazoa, with similarity to human mitochondrial ribosomal protein MRPL49	0,65
0,54	YNL185C	16705	14	Mitochondrial ribosomal protein of the large subunit	3,44
0,06	YGL103W	16711	1	Ribosomal 60S subunit protein L28; homologous to mammalian ribosomal protein L27A and bacterial L15; may have peptidyl transferase activity; can mutate to cycloheximide resistance	0,28
0,22	YOR286W	16733	2	Protein with rhodanese activity; contains a rhodanese-like domain similar to Rdl1p, Uba4p, Tum1p, and Ych1p; overexpression causes a cell cycle delay; null mutant displays elevated frequency of mitochondrial genome loss	0,64
0,19	YIL087C	16913	2	Putative protein of unknown function; mitochondrial protein that physically interacts with Tim23p; null mutant displays reduced respiratory growth	0,63
0,2	YDR064W	17018	3	Protein component of the small (40S) ribosomal subunit; homologous to mammalian ribosomal protein S13 and bacterial S15	0,63
0,66	YDL004W	17056	29	Delta subunit of the central stalk of mitochondrial F1F0 ATP synthase; F1F0 ATP synthase is a large, evolutionarily conserved enzyme complex required for ATP synthesis; F1 translationally regulates ATP6 and ATP8 expression to achieve a balanced outp	10,28
0,36	YML026C	17073	7	Protein component of the small (40S) ribosomal subunit; homologous to mammalian ribosomal protein S18 and bacterial S13; RPS18B has a paralog, RPS18A, that arose from the whole genome duplication; protein abundance increases in response to DNA repli	1,64
0,08	YNR036C	17090	1	Mitochondrial protein; may interact with ribosomes based on co-purification experiments; similar to E. coli and human mitochondrial S12 ribosomal proteins	0,27
0,37	YNL052W	17130	23	Subunit Va of cytochrome c oxidase; cytochrome c oxidase is the terminal member of the mitochondrial inner membrane electron transport chain; Cox5Ap is predominantly expressed during aerobic growth while its isoform Vb (Cox5Bp) is expressed during a	5,95
0,07	YER044C	17216	1	Endoplasmic reticulum membrane protein; may facilitate protein-protein interactions between the Erg26p dehydrogenase and the Erg27p 3-ketoreductase and/or tether these enzymes to the ER, also interacts with Erg6p	0,27
0,45	YGL187C	17316	6	Subunit IV of cytochrome c oxidase; the terminal member of the mitochondrial inner membrane electron transport chain; precursor N-terminal 25 residues are cleaved during mitochondrial import; phosphorylated; spermidine enhances translation	2,31

0,32	YFR033C	17339	6	Subunit 6 of the ubiquinol cytochrome-c reductase complex; the complex, also known as the cytochrome bc(1) complex or Complex III, is a component of the mitochondrial inner membrane electron transport chain; highly acidic protein; required for matur	0,61
0,15	YDR462W	17377	3	Mitochondrial ribosomal protein of the large subunit; protein abundance increases in response to DNA replication stress	0,61
0,53	YHR051W	17377	18	Subunit VI of cytochrome c oxidase (Complex IV); Complex IV is the terminal member of the mitochondrial inner membrane electron transport chain; expression is regulated by oxygen levels	5,7
0,06	YDR155C	17472	1	Cytoplasmic peptidyl-prolyl cis-trans isomerase (cyclophilin); catalyzes the cis-trans isomerization of peptide bonds N-terminal to proline residues; binds the drug cyclosporin A; N-terminally propionylated in vivo; protein abundance increases in re	0,27
0,12	YGL031C	17603	3	Ribosomal 60S subunit protein L24A; not essential for translation but may be required for normal translation rate; homologous to mammalian ribosomal protein L24, no bacterial homolog; RPL24A has a paralog, RPL24B, that arose from the whole genome du	0,6
0,45	YMR158W	17689	6	Mitochondrial ribosomal protein of the small subunit	3,09
0,21	YIL065C	17814	4	Protein involved in mitochondrial fission and peroxisome abundance; may have a distinct role in tethering protein aggregates to mitochondria in order to retain them in the mother cell; required for localization of Dnm1p and Mdv1p during mitochondria	1,01
0,2	YDR025W	17830	8	Protein component of the small (40S) ribosomal subunit; homologous to mammalian ribosomal protein S11 and bacterial S17; N-terminally propionylated in vivo; RPS11A has a paralog, RPS11B, that arose from the whole genome duplication	1,01
0,06	YPR098C	17857	1	Protein of unknown function; localized to the mitochondrial outer membrane	0,26
0,24	YEL054C	17858	9	Ribosomal 60S subunit protein L12A; rpl12a rpl12b double mutant exhibits slow growth and slow translation; homologous to mammalian ribosomal protein L12 and bacterial L11; RPL12A has a paralog, RPL12B, that arose from the whole genome duplication	1,01
0,3	YLR008C	17899	10	Subunit of the import motor (PAM complex); the PAM complex is a component of the Translocase of the Inner Mitochondrial membrane (TIM23 complex); essential J-protein cochaperone that stimulates Ssc1p ATPase activity to drive import; inhibited by Pa	2,19
0,07	YPL135W	18021	1	Conserved protein of the mitochondrial matrix; performs a scaffolding function during assembly of iron-sulfur clusters, interacts physically and functionally with yeast frataxin (Yfh1p); isu1 isu2 double mutant is inviable; ISU1 has a paralog, ISU2,	0,26
0,23	YBR191W	18277	5	Ribosomal 60S subunit protein L21A; homologous to mammalian ribosomal protein L21, no bacterial homolog; RPL21A has a paralog, RPL21B, that arose from the whole genome duplication	1,47
0,16	YIL016W	18341	2	Ribosome-associated protein; proposed to act in protein synthesis and nuclear pore complex biogenesis and maintenance as well as protein folding; has similarity to the mammalian BAG-1 protein	0,57
0,08	YJL096W	18376	2	Mitochondrial ribosomal protein of the large subunit	0,25
0,13	YOR150W	18544	2	Mitochondrial ribosomal protein of the large subunit; localizes to vacuole in response to H2O2	0,56
0,14	YML030W	18558	4	Cytochrome c oxidase subunit; required for assembly of the Complex III-Complex IV supercomplex, and for assembly of Cox13p and Rcf2p into cytochrome c oxidase; similar to Rcf2p, and either Rcf1p or Rcf2p is required for late-stage assembly of the Co	0,56
0,45	YBR120C	18667	19	Mitochondrial protein required for translation of the COB mRNA; forms a complex with Cbp3p that binds to mt ribosomes near the polypeptide tunnel exit and promotes efficient translation of the COB mRNA; Cbp3p-Cbp6p complex also interacts with newly	8,25
0,18	YLR164W	18758	4	Putative alternate subunit of succinate dehydrogenase (SDH); mitochondrial inner membrane protein; genetic interaction with SDH4 suggests that Shh4p can function as a functional SDH subunit; a fraction copurifies with SDH subunit Sdh3p; expression i	0,56
0,09	YIR022W	18796	1	18kDa catalytic subunit of the Signal Peptidase Complex (SPC); the Signal Peptidase Complex cleaves the signal sequence of proteins targeted to the endoplasmic reticulum; other members are Spc1p, Spc2p, Spc3p, and Sec11p	0,25
0,05	YOR357C	18850	1	Sorting nexin for late-Golgi enzymes; required to maintain late-Golgi resident enzymes in their proper location by recycling molecules from the prevacuolar compartment; contains a PX domain and sequence similarity to human Snx3p	0,25
0,27	YPR149W	19185	6	Protein of unknown function; contains transmembrane domains; involved in secretion of proteins that lack classical secretory signal sequences; component of the detergent-insoluble glycolipid-enriched complexes (DIGs); NCE102 has a paralog, FHN1, tha	0,91

0,26	YCR046C	19428	6	Mitochondrial ribosomal protein of the large subunit; required for respiration and for maintenance of the mitochondrial genome	1,35
0,2	YGR085C	19783	4	Ribosomal 60S subunit protein L11B; expressed at half the level of Rpl11Ap; involved in ribosomal assembly; depletion causes degradation of 60S proteins and RNA; homologous to mammalian ribosomal protein L11 and bacterial L5; RPL11B has a paralog, R	0,88
0,91	YKL016C	19797	190	Subunit d of the stator stalk of mitochondrial F1F0 ATP synthase; F1F0 ATP synthase is a large, evolutionarily conserved enzyme complex required for ATP synthesis	10408,91
0,14	YML073C	19949	2	Ribosomal 60S subunit protein L6A; N-terminally acetylated; binds 5.8S rRNA; homologous to mammalian ribosomal protein L6, no bacterial homolog; RPL6A has a paralog, RPL6B, that arose from the whole genome duplication	0,52
0,41	YML078W	19998	18	Mitochondrial peptidyl-prolyl cis-trans isomerase (cyclophilin); catalyzes the cis-trans isomerization of peptide bonds N-terminal to proline residues; involved in protein refolding after import into mitochondria	3,29

BAND 19

prot_hit_num	prot_acc	prot_desc	prot_mass	prot_matches	prot_cover
140	Q5VTU8	ATP synthase subunit epsilon-like protein, mitochondrial OS=Homo sapiens OX=9606 GN=ATP5F1EP2 PE=1 SV=1	5860	1	15,7
86	Q8N4H5	Mitochondrial import receptor subunit TOM5 homolog OS=Homo sapiens OX=9606 GN=TOMM5 PE=1 SV=1	6031	22	45,1
167	Q9P0U1	Mitochondrial import receptor subunit TOM7 homolog OS=Homo sapiens OX=9606 GN=TOMM7 PE=1 SV=1	6244	6	38,2
69	Q96IX5	ATP synthase membrane subunit K, mitochondrial OS=Homo sapiens OX=9606 GN=ATP5MK PE=1 SV=1	6510	1	25,9
78	O75438	NADH dehydrogenase [ubiquinone] 1 beta subcomplex subunit 1 OS=Homo sapiens OX=9606 GN=NDUFB1 PE=1 SV=1	7014	3	15,5
68	Q14061	Cytochrome c oxidase copper chaperone OS=Homo sapiens OX=9606 GN=COX17 PE=1 SV=2	7253	1	25,4
36	Q9UDW1	Cytochrome b-c1 complex subunit 9 OS=Homo sapiens OX=9606 GN=UQCR10 PE=1 SV=3	7304	4	38,1
179	P60059	Protein transport protein Sec61 subunit gamma OS=Homo sapiens OX=9606 GN=SEC61G PE=1 SV=1	7793	2	19,1
63	P62857	40S ribosomal protein S28 OS=Homo sapiens OX=9606 GN=RPS28 PE=1 SV=1	7893	5	30,4
116	P56385	ATP synthase subunit e, mitochondrial OS=Homo sapiens OX=9606 GN=ATP5ME PE=1 SV=2	7928	2	24,6
42	Q96B49	Mitochondrial import receptor subunit TOM6 homolog OS=Homo sapiens OX=9606 GN=TOMM6 PE=1 SV=1	7997	11	44,6
53	Q9UBI6	Guanine nucleotide-binding protein G(I)/G(S)/G(O) subunit gamma-12 OS=Homo sapiens OX=9606 GN=GNG12 PE=1 SV=3	8115	3	37,5
87	P63173	60S ribosomal protein L38 OS=Homo sapiens OX=9606 GN=RPL38 PE=1 SV=2	8270	4	34,3
128	L0R8F8	MIEF1 upstream open reading frame protein OS=Homo sapiens OX=9606 GN=MIEF1 PE=1 SV=1	8440	2	11,4
92	Q8WVI0	Small integral membrane protein 4 OS=Homo sapiens OX=9606 GN=SMIM4 PE=1 SV=2	8691	1	15,7
158	O43677	NADH dehydrogenase [ubiquinone] 1 subunit C1, mitochondrial OS=Homo sapiens OX=9606 GN=NDUFC1 PE=1 SV=1	8729	1	13,2
26	P09669	Cytochrome c oxidase subunit 6C OS=Homo sapiens OX=9606 GN=COX6C PE=1 SV=2	8776	21	52
131	Q15843	NEDD8 OS=Homo sapiens OX=9606 GN=NEDD8 PE=1 SV=1	9066	1	17,3
166	P0DJ07	Protein PET100 homolog, mitochondrial OS=Homo sapiens OX=9606 GN=PET100 PE=1 SV=1	9108	1	11
80	P63220	40S ribosomal protein S21 OS=Homo sapiens OX=9606 GN=RPS21 PE=1 SV=1	9220	1	16,9
124	O95167	NADH dehydrogenase [ubiquinone] 1 alpha subcomplex subunit 3 OS=Homo sapiens OX=9606 GN=NDUFA3 PE=1 SV=1	9273	2	21,4
32	P14406	Cytochrome c oxidase subunit 7A2, mitochondrial OS=Homo sapiens OX=9606 GN=COX7A2 PE=1 SV=1	9390	8	27,7
139	O00483	Cytochrome c oxidase subunit NDUFA4 OS=Homo sapiens OX=9606 GN=NDUFA4 PE=1 SV=1	9421	2	22,2
171	Q9Y5J9	Mitochondrial import inner membrane translocase subunit Tim8 B OS=Homo sapiens OX=9606 GN=TIMM8B PE=1 SV=1	9566	1	8,4
133	Q9NRP2	COX assembly mitochondrial protein 2 homolog OS=Homo sapiens OX=9606 GN=CMC2 PE=1 SV=1	9682	3	21,5

82	P60468	Protein transport protein Sec61 subunit beta OS=Homo sapiens OX=9606 GN=SEC61B PE=1 SV=2	10025	1	15,6
177	P07108	Acyl-CoA-binding protein OS=Homo sapiens OX=9606 GN=DBI PE=1 SV=2	10038	1	23
61	Q9BQ48	39S ribosomal protein L34, mitochondrial OS=Homo sapiens OX=9606 GN=MRPL34 PE=1 SV=1	10159	2	13
90	P14854	Cytochrome c oxidase subunit 6B1 OS=Homo sapiens OX=9606 GN=COX6B1 PE=1 SV=2	10414	7	26,7
154	Q96FJ2	Dynein light chain 2, cytoplasmic OS=Homo sapiens OX=9606 GN=DYNLL2 PE=1 SV=1	10457	2	20,2
155	P63167	Dynein light chain 1, cytoplasmic OS=Homo sapiens OX=9606 GN=DYNLL1 PE=1 SV=1	10530	2	20,2
43	P62072	Mitochondrial import inner membrane translocase subunit Tim10 OS=Homo sapiens OX=9606 GN=TIMM10 PE=1 SV=1	10554	5	22,2
44	Q9Y5J7	Mitochondrial import inner membrane translocase subunit Tim9 OS=Homo sapiens OX=9606 GN=TIMM9 PE=1 SV=1	10599	4	34,8
70	Q9Y5L4	Mitochondrial import inner membrane translocase subunit Tim13 OS=Homo sapiens OX=9606 GN=TIMM13 PE=1 SV=1	10721	1	14,7
66	Q9HD34	LYR motif-containing protein 4 OS=Homo sapiens OX=9606 GN=LYRM4 PE=1 SV=1	10752	2	28,6
127	P62304	Small nuclear ribonucleoprotein E OS=Homo sapiens OX=9606 GN=SNRPE PE=1 SV=1	10854	2	12
81	P05109	Protein S100-A8 OS=Homo sapiens OX=9606 GN=S100A8 PE=1 SV=1	10885	2	11,8
74	P82921	28S ribosomal protein S21, mitochondrial OS=Homo sapiens OX=9606 GN=MRPS21 PE=1 SV=3	10909	1	16,1
34	Q9NP97	Dynein light chain roadblock-type 1 OS=Homo sapiens OX=9606 GN=DYNLRB1 PE=1 SV=3	10915	6	34,4
1	P61604	10 kDa heat shock protein, mitochondrial OS=Homo sapiens OX=9606 GN=HSPE1 PE=1 SV=2	10925	372	81,4
57	A0A096LP55	Cytochrome b-c1 complex subunit 6-like, mitochondrial OS=Homo sapiens OX=9606 GN=UQCRHL PE=3 SV=1	10973	1	23,1
24	P01040	Cystatin-A OS=Homo sapiens OX=9606 GN=CSTA PE=1 SV=1	11000	10	64,3
105	P56134	ATP synthase subunit f, mitochondrial OS=Homo sapiens OX=9606 GN=ATP5MF PE=1 SV=3	11025	1	13,8
71	O43678	NADH dehydrogenase [ubiquinone] 1 alpha subcomplex subunit 2 OS=Homo sapiens OX=9606 GN=NDUFA2 PE=1 SV=3	11029	3	19,2
33	L0R6Q1	SLC35A4 upstream open reading frame protein OS=Homo sapiens OX=9606 GN=SLC35A4 PE=3 SV=1	11183	5	36,9
159	P04080	Cystatin-B OS=Homo sapiens OX=9606 GN=CSTB PE=1 SV=2	11190	1	12,2
49	O60220	Mitochondrial import inner membrane translocase subunit Tim8 A OS=Homo sapiens OX=9606 GN=TIMM8A PE=1 SV=1	11219	3	20,6
39	P62805	Histone H4 OS=Homo sapiens OX=9606 GN=H4C1 PE=1 SV=2	11360	8	38,8
98	P81605	Dermcidin OS=Homo sapiens OX=9606 GN=DCD PE=1 SV=2	11391	2	10
23	P82909	Alpha-ketoglutarate dehydrogenase component 4 OS=Homo sapiens OX=9606 GN=MRPS36 PE=1 SV=2	11459	8	68,9
21	P31151	Protein S100-A7 OS=Homo sapiens OX=9606 GN=S100A7 PE=1 SV=4	11578	20	22,8
14	P05387	60S acidic ribosomal protein P2 OS=Homo sapiens OX=9606 GN=RPLP2 PE=1 SV=1	11658	11	84,3
52	Q9Y5J6	Mitochondrial import inner membrane translocase subunit Tim10 B OS=Homo sapiens OX=9606 GN=TIMM10B PE=1 SV=1	11807	6	46,6
11	P99999	Cytochrome c OS=Homo sapiens OX=9606 GN=CYCS PE=1 SV=2	11855	36	61,9
151	P62942	Peptidyl-prolyl cis-trans isomerase FKBP1A OS=Homo sapiens OX=9606 GN=FKBP1A PE=1 SV=2	12000	1	12
182	Q5U5X0	Complex III assembly factor LYRM7 OS=Homo sapiens OX=9606 GN=LYRM7 PE=1 SV=1	12004	3	21,2
102	P10599	Thioredoxin OS=Homo sapiens OX=9606 GN=TXN PE=1 SV=3	12015	4	28,6
156	P35754	Glutaredoxin-1 OS=Homo sapiens OX=9606 GN=GLRX PE=1 SV=2	12053	2	11,3
168	Q9NZ42	Gamma-secretase subunit PEN-2 OS=Homo sapiens OX=9606 GN=PSENEN PE=1 SV=1	12078	1	14,9
149	P12074	Cytochrome c oxidase subunit 6A1, mitochondrial OS=Homo sapiens OX=9606 GN=COX6A1 PE=1 SV=4	12147	2	11,9
146	Q53S33	BolA-like protein 3 OS=Homo sapiens OX=9606 GN=BOLA3 PE=1 SV=1	12163	3	22,4
17	Q9UII2	ATPase inhibitor, mitochondrial OS=Homo sapiens OX=9606 GN=ATP5IF1 PE=1 SV=1	12241	31	17,9

172	Q9BQC6	Ribosomal protein 63, mitochondrial OS=Homo sapiens OX=9606 GN=MRPL57 PE=1 SV=1	12259	1	12,7
54	Q96EL3	39S ribosomal protein L53, mitochondrial OS=Homo sapiens OX=9606 GN=MRPL53 PE=1 SV=1	12270	5	28,6
130	Q5VUM1	Succinate dehydrogenase assembly factor 4, mitochondrial OS=Homo sapiens OX=9606 GN=SDHAF4 PE=1 SV=1	12319	2	9,3
148	Q9UJC5	SH3 domain-binding glutamic acid-rich-like protein 2 OS=Homo sapiens OX=9606 GN=SH3BGRL2 PE=1 SV=2	12375	1	13,1
22	Q9GZT3	SRA stem-loop-interacting RNA-binding protein, mitochondrial OS=Homo sapiens OX=9606 GN=SLIRP PE=1 SV=1	12398	23	65,1
64	Q96DA6	Mitochondrial import inner membrane translocase subunit TIM14 OS=Homo sapiens OX=9606 GN=DNAJC19 PE=1 SV=3	12491	3	19
47	P14174	Macrophage migration inhibitory factor OS=Homo sapiens OX=9606 GN=MIF PE=1 SV=4	12639	4	10,4
106	P61803	Dolichyl-diphosphooligosaccharide--protein glycosyltransferase subunit DAD1 OS=Homo sapiens OX=9606 GN=DAD1 PE=1 SV=3	12660	1	10,6
142	O14548	Cytochrome c oxidase subunit 7A-related protein, mitochondrial OS=Homo sapiens OX=9606 GN=COX7A2L PE=1 SV=2	12664	1	7,9
45	O75368	SH3 domain-binding glutamic acid-rich-like protein OS=Homo sapiens OX=9606 GN=SH3BGRL PE=1 SV=1	12766	8	40,4
30	P30046	D-dopachrome decarboxylase OS=Homo sapiens OX=9606 GN=DDT PE=1 SV=3	12818	10	36,4
150	Q7Z7K0	COX assembly mitochondrial protein homolog OS=Homo sapiens OX=9606 GN=CMC1 PE=1 SV=1	12823	1	8,5
73	P41567	Eukaryotic translation initiation factor 1 OS=Homo sapiens OX=9606 GN=EIF1 PE=1 SV=1	12839	2	26,5
132	Q96HJ9	Protein FMC1 homolog OS=Homo sapiens OX=9606 GN=FMC1 PE=1 SV=2	12855	2	17,7
29	P06702	Protein S100-A9 OS=Homo sapiens OX=9606 GN=S100A9 PE=1 SV=1	13291	9	42,1
174	P60866	40S ribosomal protein S20 OS=Homo sapiens OX=9606 GN=RPS20 PE=1 SV=1	13478	2	10,1
40	Q16718	NADH dehydrogenase [ubiquinone] 1 alpha subcomplex subunit 5 OS=Homo sapiens OX=9606 GN=NDUFA5 PE=1 SV=3	13507	5	17,2
152	P49207	60S ribosomal protein L34 OS=Homo sapiens OX=9606 GN=RPL34 PE=1 SV=3	13513	1	6,8
67	P14927	Cytochrome b-c1 complex subunit 7 OS=Homo sapiens OX=9606 GN=UQCRB PE=1 SV=2	13522	3	19,8
112	P0C0S5	Histone H2A.Z OS=Homo sapiens OX=9606 GN=H2AZ1 PE=1 SV=2	13545	1	7
83	Q9UHA4	Ragulator complex protein LAMTOR3 OS=Homo sapiens OX=9606 GN=LAMTOR3 PE=1 SV=1	13671	2	8,1
93	Q96BP2	Coiled-coil-helix-coiled-coil-helix domain-containing protein 1 OS=Homo sapiens OX=9606 GN=CHCHD1 PE=1 SV=1	13694	2	22
79	P60520	Gamma-aminobutyric acid receptor-associated protein-like 2 OS=Homo sapiens OX=9606 GN=GABARAPL2 PE=1 SV=1	13715	2	6,8
58	O14907	Tax1-binding protein 3 OS=Homo sapiens OX=9606 GN=TAX1BP3 PE=1 SV=2	13726	1	13,7
111	P62851	40S ribosomal protein S25 OS=Homo sapiens OX=9606 GN=RPS25 PE=1 SV=1	13791	1	7,2
50	Q9Y3D7	Mitochondrial import inner membrane translocase subunit TIM16 OS=Homo sapiens OX=9606 GN=PAM16 PE=1 SV=2	13816	4	39,2
121	O75348	V-type proton ATPase subunit G 1 OS=Homo sapiens OX=9606 GN=ATP6V1G1 PE=1 SV=3	13863	1	7,6
109	P49773	Adenosine 5~-monophosphoramidase HINT1 OS=Homo sapiens OX=9606 GN=HINT1 PE=1 SV=2	13907	1	7,1
76	Q5QNW6	Histone H2B type 2-F OS=Homo sapiens OX=9606 GN=H2BC18 PE=1 SV=3	13912	2	11,9
19	P10606	Cytochrome c oxidase subunit 5B, mitochondrial OS=Homo sapiens OX=9606 GN=COX5B PE=1 SV=2	13915	20	38,8
135	O75380	NADH dehydrogenase [ubiquinone] iron-sulfur protein 6, mitochondrial OS=Homo sapiens OX=9606 GN=NDUFS6 PE=1 SV=1	14045	2	11,3
123	P82932	28S ribosomal protein S6, mitochondrial OS=Homo sapiens OX=9606 GN=MRPS6 PE=1 SV=3	14275	2	12,8
176	Q9BUE6	Iron-sulfur cluster assembly 1 homolog, mitochondrial OS=Homo sapiens OX=9606 GN=ISCA1 PE=1 SV=1	14342	1	10,1
77	Q5JTJ3	Cytochrome c oxidase assembly factor 6 homolog OS=Homo sapiens OX=9606 GN=COA6 PE=1 SV=1	14449	1	11,2
31	Q9NPJ3	Acyl-coenzyme A thioesterase 13 OS=Homo sapiens OX=9606 GN=ACOT13 PE=1 SV=1	15065	6	28,6
15	Q7Z7F7	39S ribosomal protein L55, mitochondrial OS=Homo sapiens OX=9606 GN=MRPL55 PE=1 SV=1	15119	11	37,5
84	P56556	NADH dehydrogenase [ubiquinone] 1 alpha subcomplex subunit 6 OS=Homo sapiens OX=9606 GN=NDUFA6 PE=1 SV=4	15127	2	6,2

18	P07737	Profilin-1 OS=Homo sapiens OX=9606 GN=PFN1 PE=1 SV=2	15216	8	30
62	Q8IXM3	39S ribosomal protein L41, mitochondrial OS=Homo sapiens OX=9606 GN=MRPL41 PE=1 SV=1	15430	3	24,1
94	Q01469	Fatty acid-binding protein 5 OS=Homo sapiens OX=9606 GN=FABP5 PE=1 SV=3	15497	5	12,6
51	Q9Y6H1	Coiled-coil-helix-coiled-coil-helix domain-containing protein 2 OS=Homo sapiens OX=9606 GN=CHCHD2 PE=1 SV=1	15731	3	33,8
162	P26885	Peptidyl-prolyl cis-trans isomerase FKBP2 OS=Homo sapiens OX=9606 GN=FKBP2 PE=1 SV=2	15810	2	9,2
35	Q9NZT1	Calmodulin-like protein 5 OS=Homo sapiens OX=9606 GN=CALML5 PE=1 SV=2	15883	5	28,8
113	Q6GMV3	Putative peptidyl-tRNA hydrolase PTRHD1 OS=Homo sapiens OX=9606 GN=PTRHD1 PE=1 SV=1	15909	1	11,4
129	Q9P0M9	39S ribosomal protein L27, mitochondrial OS=Homo sapiens OX=9606 GN=MRPL27 PE=1 SV=1	16120	2	20,9
46	Q6P1L8	39S ribosomal protein L14, mitochondrial OS=Homo sapiens OX=9606 GN=MRPL14 PE=1 SV=1	16165	6	31,7
101	Q86U28	Iron-sulfur cluster assembly 2 homolog, mitochondrial OS=Homo sapiens OX=9606 GN=ISCA2 PE=1 SV=2	16694	1	7,8
13	P20674	Cytochrome c oxidase subunit 5A, mitochondrial OS=Homo sapiens OX=9606 GN=COX5A PE=1 SV=2	16923	25	64,7
95	P61626	Lysozyme C OS=Homo sapiens OX=9606 GN=LYZ PE=1 SV=1	16982	1	8,1
20	Q9BX68	Adenosine 5~-monophosphoramidase HINT2 OS=Homo sapiens OX=9606 GN=HINT2 PE=1 SV=1	17208	11	41,1
119	P62277	40S ribosomal protein S13 OS=Homo sapiens OX=9606 GN=RPS13 PE=1 SV=2	17212	1	7,9
28	Q04837	Single-stranded DNA-binding protein, mitochondrial OS=Homo sapiens OX=9606 GN=SSBP1 PE=1 SV=1	17249	11	57,4
55	P30049	ATP synthase subunit delta, mitochondrial OS=Homo sapiens OX=9606 GN=ATP5F1D PE=1 SV=2	17479	3	22,6
72	O14561	Acyl carrier protein, mitochondrial OS=Homo sapiens OX=9606 GN=NDUFAB1 PE=1 SV=3	17577	2	9
107	P30050	60S ribosomal protein L12 OS=Homo sapiens OX=9606 GN=RPL12 PE=1 SV=1	17979	1	5,5
25	P62979	Ubiquitin-40S ribosomal protein S27a OS=Homo sapiens OX=9606 GN=RPS27A PE=1 SV=2	18296	15	18,6
59	Q8N5N7	39S ribosomal protein L50, mitochondrial OS=Homo sapiens OX=9606 GN=MRPL50 PE=1 SV=2	18484	3	15,2
141	Q9Y6A9	Signal peptidase complex subunit 1 OS=Homo sapiens OX=9606 GN=SPCS1 PE=1 SV=5	18514	1	8,9
183	Q99643	Succinate dehydrogenase cytochrome b560 subunit, mitochondrial OS=Homo sapiens OX=9606 GN=SDHC PE=1 SV=1	18826	1	8,9
114	P46783	40S ribosomal protein S10 OS=Homo sapiens OX=9606 GN=RPS10 PE=1 SV=1	18886	2	9,1
175	P13073	Cytochrome c oxidase subunit 4 isoform 1, mitochondrial OS=Homo sapiens OX=9606 GN=COX4I1 PE=1 SV=1	19621	1	6,5
164	P00492	Hypoxanthine-guanine phosphoribosyltransferase OS=Homo sapiens OX=9606 GN=HPRT1 PE=1 SV=2	24792	2	4,6
117	Q96CT7	Coiled-coil domain-containing protein 124 OS=Homo sapiens OX=9606 GN=CCDC124 PE=1 SV=1	25820	1	4,5
41	O95292	Vesicle-associated membrane protein-associated protein B/C OS=Homo sapiens OX=9606 GN=VAPB PE=1 SV=3	27439	5	14,8
160	Q9NX40	OCIA domain-containing protein 1 OS=Homo sapiens OX=9606 GN=OCIAD1 PE=1 SV=1	27780	1	4,1
147	P31944	Caspase-14 OS=Homo sapiens OX=9606 GN=CASP14 PE=1 SV=2	27947	2	9,1
178	Q04917	14-3-3 protein eta OS=Homo sapiens OX=9606 GN=YWHAH PE=1 SV=4	28372	1	4,1
161	P21796	Voltage-dependent anion-selective channel protein 1 OS=Homo sapiens OX=9606 GN=VDAC1 PE=1 SV=2	30868	2	10,6
97	Q9Y277	Voltage-dependent anion-selective channel protein 3 OS=Homo sapiens OX=9606 GN=VDAC3 PE=1 SV=1	30981	2	8,1
96	P0C7P4	Putative cytochrome b-c1 complex subunit Rieske-like protein 1 OS=Homo sapiens OX=9606 GN=UQCRFS1P1 PE=5 SV=1	31081	2	7,8
38	P29692	Elongation factor 1-delta OS=Homo sapiens OX=9606 GN=EEF1D PE=1 SV=5	31217	4	17,1
60	Q07021	Complement component 1 Q subcomponent-binding protein, mitochondrial OS=Homo sapiens OX=9606 GN=C1QBP PE=1 SV=1	31742	2	5
65	P35030	Trypsin-3 OS=Homo sapiens OX=9606 GN=PRSS3 PE=1 SV=2	33306	2	3,3
181	P05089	Arginase-1 OS=Homo sapiens OX=9606 GN=ARG1 PE=1 SV=2	34884	1	3,7

89	Q9H0C2	ADP/ATP translocase 4 OS=Homo sapiens OX=9606 GN=SLC25A31 PE=1 SV=1	35285	2	3,8
122	Q92820	Gamma-glutamyl hydrolase OS=Homo sapiens OX=9606 GN=GGH PE=1 SV=2	36340	1	4,7
165	Q9H3Q1	Cdc42 effector protein 4 OS=Homo sapiens OX=9606 GN=CDC42EP4 PE=1 SV=1	38014	1	4,5
126	Q9UHD1	Cysteine and histidine-rich domain-containing protein 1 OS=Homo sapiens OX=9606 GN=CHORDC1 PE=1 SV=2	38264	2	4,5
118	O75787	Renin receptor OS=Homo sapiens OX=9606 GN=ATP6AP2 PE=1 SV=2	38983	1	3,1
163	O00115	Deoxyribonuclease-2-alpha OS=Homo sapiens OX=9606 GN=DNASE2 PE=1 SV=2	40069	1	3,3
110	P60709	Actin, cytoplasmic 1 OS=Homo sapiens OX=9606 GN=ACTB PE=1 SV=1	42052	2	5,3
48	Q8NC51	Plasminogen activator inhibitor 1 RNA-binding protein OS=Homo sapiens OX=9606 GN=SERBP1 PE=1 SV=2	44995	4	10,3
153	P07339	Cathepsin D OS=Homo sapiens OX=9606 GN=CTSD PE=1 SV=1	45037	2	1,9
143	Q92665	28S ribosomal protein S31, mitochondrial OS=Homo sapiens OX=9606 GN=MRPS31 PE=1 SV=3	45405	1	2,3
120	Q6YN16	Hydroxysteroid dehydrogenase-like protein 2 OS=Homo sapiens OX=9606 GN=HSDL2 PE=1 SV=1	45651	3	10,3
100	Q96P63	Serpin B12 OS=Homo sapiens OX=9606 GN=SERPINB12 PE=1 SV=1	46646	1	4
108	Q8IW75	Serpin A12 OS=Homo sapiens OX=9606 GN=SERPINA12 PE=1 SV=1	47259	1	2,2
99	Q6E0U4	Dermokine OS=Homo sapiens OX=9606 GN=DMKN PE=1 SV=3	47282	1	3,6
16	Q04695	Keratin, type I cytoskeletal 17 OS=Homo sapiens OX=9606 GN=KRT17 PE=1 SV=2	48361	33	40,5
125	P49411	Elongation factor Tu, mitochondrial OS=Homo sapiens OX=9606 GN=TUFM PE=1 SV=2	49852	2	5,8
137	Q05639	Elongation factor 1-alpha 2 OS=Homo sapiens OX=9606 GN=EEF1A2 PE=1 SV=1	50780	3	9,9
173	Q16181	Septin-7 OS=Homo sapiens OX=9606 GN=SEPTIN7 PE=1 SV=2	50933	1	2,3
2	P08779	Keratin, type I cytoskeletal 16 OS=Homo sapiens OX=9606 GN=KRT16 PE=1 SV=4	51578	56	52
8	P02533	Keratin, type I cytoskeletal 14 OS=Homo sapiens OX=9606 GN=KRT14 PE=1 SV=4	51872	40	42,4
75	P19013	Keratin, type II cytoskeletal 4 OS=Homo sapiens OX=9606 GN=KRT4 PE=1 SV=5	56508	5	4,8
85	P06576	ATP synthase subunit beta, mitochondrial OS=Homo sapiens OX=9606 GN=ATP5F1B PE=1 SV=3	56525	2	4,3
144	Q8N1N4	Keratin, type II cytoskeletal 78 OS=Homo sapiens OX=9606 GN=KRT78 PE=1 SV=2	57629	3	2,3
91	Q58FF6	Putative heat shock protein HSP 90-beta 4 OS=Homo sapiens OX=9606 GN=HSP90AB4P PE=5 SV=1	58855	1	2,4
12	P13645	Keratin, type I cytoskeletal 10 OS=Homo sapiens OX=9606 GN=KRT10 PE=1 SV=6	59020	33	28,1
56	P22307	Sterol carrier protein 2 OS=Homo sapiens OX=9606 GN=SCP2 PE=1 SV=2	59640	4	5,3
6	P48668	Keratin, type II cytoskeletal 6C OS=Homo sapiens OX=9606 GN=KRT6C PE=1 SV=3	60273	58	46,5
7	P02538	Keratin, type II cytoskeletal 6A OS=Homo sapiens OX=9606 GN=KRT6A PE=1 SV=3	60293	56	44,7
4	P04259	Keratin, type II cytoskeletal 6B OS=Homo sapiens OX=9606 GN=KRT6B PE=1 SV=5	60315	60	46,5
136	Q9UQB8	Brain-specific angiogenesis inhibitor 1-associated protein 2 OS=Homo sapiens OX=9606 GN=BAIAP2 PE=1 SV=1	61115	1	2,4
5	P35527	Keratin, type I cytoskeletal 9 OS=Homo sapiens OX=9606 GN=KRT9 PE=1 SV=3	62255	27	29,2
10	P13647	Keratin, type II cytoskeletal 5 OS=Homo sapiens OX=9606 GN=KRT5 PE=1 SV=3	62568	42	31,4
138	Q86UE4	Protein LYRIC OS=Homo sapiens OX=9606 GN=MTDH PE=1 SV=2	63856	1	1,9
115	Q9UHD8	Septin-9 OS=Homo sapiens OX=9606 GN=SEPTIN9 PE=1 SV=2	65646	1	2
9	P35908	Keratin, type II cytoskeletal 2 epidermal OS=Homo sapiens OX=9606 GN=KRT2 PE=1 SV=2	65678	29	25,8
3	P04264	Keratin, type II cytoskeletal 1 OS=Homo sapiens OX=9606 GN=KRT1 PE=1 SV=6	66170	71	34,2
27	P11940	Polyadenylate-binding protein 1 OS=Homo sapiens OX=9606 GN=PABPC1 PE=1 SV=2	70854	9	4,9

37	P02768	Albumin OS=Homo sapiens OX=9606 GN=ALB PE=1 SV=2	71317	5	6,6
103	Q16891	MICOS complex subunit MIC60 OS=Homo sapiens OX=9606 GN=IMMT PE=1 SV=1	84026	1	1,7
157	Q8IYB8	ATP-dependent RNA helicase SUPV3L1, mitochondrial OS=Homo sapiens OX=9606 GN=SUPV3L1 PE=1 SV=1	88791	1	1,7
169	P13639	Elongation factor 2 OS=Homo sapiens OX=9606 GN=EEF2 PE=1 SV=4	96246	1	1,9
145	O00754	Lysosomal alpha-mannosidase OS=Homo sapiens OX=9606 GN=MAN2B1 PE=1 SV=3	114357	1	1,2
88	Q02413	Desmoglein-1 OS=Homo sapiens OX=9606 GN=DSG1 PE=1 SV=2	114702	3	3,2
134	O14974	Protein phosphatase 1 regulatory subunit 12A OS=Homo sapiens OX=9606 GN=PPP1R12A PE=1 SV=1	115610	1	1
170	Q7Z4L5	Tetratricopeptide repeat protein 21B OS=Homo sapiens OX=9606 GN=TTC21B PE=1 SV=2	152380	1	0,5
180	Q04637	Eukaryotic translation initiation factor 4 gamma 1 OS=Homo sapiens OX=9606 GN=EIF4G1 PE=1 SV=4	176124	1	1,2
104	Q86YZ3	Hornerin OS=Homo sapiens OX=9606 GN=HRNR PE=1 SV=2	283140	3	1,3

BAND 20

prot_hit_num	prot_acc	prot_desc	prot_mass	prot_matches	prot_cover
106	Q5VTU8	ATP synthase subunit epsilon-like protein, mitochondrial OS=Homo sapiens OX=9606 GN=ATP5F1EP2 PE=1 SV=1	5860	6	31,4
7	Q96IX5	ATP synthase membrane subunit K, mitochondrial OS=Homo sapiens OX=9606 GN=ATP5MK PE=1 SV=1	6510	18	46,6
128	P56378	ATP synthase subunit ATP5MJ, mitochondrial OS=Homo sapiens OX=9606 GN=ATP5MJ PE=1 SV=1	6658	17	48,3
60	O75438	NADH dehydrogenase [ubiquinone] 1 beta subcomplex subunit 1 OS=Homo sapiens OX=9606 GN=NDUFB1 PE=1 SV=1	7014	7	15,5
158	Q9UDW1	Cytochrome b-c1 complex subunit 9 OS=Homo sapiens OX=9606 GN=UQCR10 PE=1 SV=3	7304	1	11,1
81	P62857	40S ribosomal protein S28 OS=Homo sapiens OX=9606 GN=RPS28 PE=1 SV=1	7893	3	30,4
4	P56385	ATP synthase subunit e, mitochondrial OS=Homo sapiens OX=9606 GN=ATP5ME PE=1 SV=2	7928	63	72,5
77	Q96B49	Mitochondrial import receptor subunit TOM6 homolog OS=Homo sapiens OX=9606 GN=TOMM6 PE=1 SV=1	7997	5	18,9
6	P03928	ATP synthase protein 8 OS=Homo sapiens OX=9606 GN=MT-ATP8 PE=1 SV=1	8043	37	50
157	Q69YU5	Protein BRAWNIN OS=Homo sapiens OX=9606 GN=BRAWNIN PE=1 SV=2	8074	1	16,9
142	Q9UBI6	Guanine nucleotide-binding protein G(I)/G(S)/G(O) subunit gamma-12 OS=Homo sapiens OX=9606 GN=GNG12 PE=1 SV=3	8115	2	31,9
120	P09669	Cytochrome c oxidase subunit 6C OS=Homo sapiens OX=9606 GN=COX6C PE=1 SV=2	8776	4	22,7
139	Q15843	NEDD8 OS=Homo sapiens OX=9606 GN=NEDD8 PE=1 SV=1	9066	1	13,6
100	P61960	Ubiquitin-fold modifier 1 OS=Homo sapiens OX=9606 GN=UFM1 PE=1 SV=1	9169	2	17,6
165	P63220	40S ribosomal protein S21 OS=Homo sapiens OX=9606 GN=RPS21 PE=1 SV=1	9220	1	12
122	O95167	NADH dehydrogenase [ubiquinone] 1 alpha subcomplex subunit 3 OS=Homo sapiens OX=9606 GN=NDUFA3 PE=1 SV=1	9273	2	13,1
45	P14406	Cytochrome c oxidase subunit 7A2, mitochondrial OS=Homo sapiens OX=9606 GN=COX7A2 PE=1 SV=1	9390	5	42,2
144	O00483	Cytochrome c oxidase subunit NDUFA4 OS=Homo sapiens OX=9606 GN=NDUFA4 PE=1 SV=1	9421	2	22,2
123	Q71UM5	40S ribosomal protein S27-like OS=Homo sapiens OX=9606 GN=RPS27L PE=1 SV=3	9813	1	15,5
33	O14949	Cytochrome b-c1 complex subunit 8 OS=Homo sapiens OX=9606 GN=UQCRQ PE=1 SV=4	9900	10	37,8
98	P60468	Protein transport protein Sec61 subunit beta OS=Homo sapiens OX=9606 GN=SEC61B PE=1 SV=2	10025	1	15,6
57	Q9BQ48	39S ribosomal protein L34, mitochondrial OS=Homo sapiens OX=9606 GN=MRPL34 PE=1 SV=1	10159	2	13
143	Q96FJ2	Dynein light chain 2, cytoplasmic OS=Homo sapiens OX=9606 GN=DYNLL2 PE=1 SV=1	10457	1	12,4

153	P61513	60S ribosomal protein L37a OS=Homo sapiens OX=9606 GN=RPL37A PE=1 SV=2	10497	2	9,8
124	Q9Y5J7	Mitochondrial import inner membrane translocase subunit Tim9 OS=Homo sapiens OX=9606 GN=TIMM9 PE=1 SV=1	10599	1	9
108	Q49B96	Cytochrome c oxidase assembly protein COX19 OS=Homo sapiens OX=9606 GN=COX19 PE=1 SV=1	10615	1	11,1
19	Q9Y5L4	Mitochondrial import inner membrane translocase subunit Tim13 OS=Homo sapiens OX=9606 GN=TIMM13 PE=1 SV=1	10721	8	48,4
111	P0DP57	Secreted Ly-6/uPAR domain-containing protein 2 OS=Homo sapiens OX=9606 GN=SLURP2 PE=1 SV=1	10723	1	13,4
102	Q9HD34	LYR motif-containing protein 4 OS=Homo sapiens OX=9606 GN=LYRM4 PE=1 SV=1	10752	2	15,4
154	Q6IPR1	Electron transfer flavoprotein regulatory factor 1 OS=Homo sapiens OX=9606 GN=ETFRF1 PE=1 SV=2	10857	2	10
119	P05109	Protein S100-A8 OS=Homo sapiens OX=9606 GN=S100A8 PE=1 SV=1	10885	4	28
95	Q8TF09	Dynein light chain roadblock-type 2 OS=Homo sapiens OX=9606 GN=DYNLRB2 PE=1 SV=1	10905	1	12,5
29	P82921	28S ribosomal protein S21, mitochondrial OS=Homo sapiens OX=9606 GN=MRPS21 PE=1 SV=3	10909	4	29,9
2	P61604	10 kDa heat shock protein, mitochondrial OS=Homo sapiens OX=9606 GN=HSPE1 PE=1 SV=2	10925	108	75,5
8	P01040	Cystatin-A OS=Homo sapiens OX=9606 GN=CSTA PE=1 SV=1	11000	20	67,3
12	P56134	ATP synthase subunit f, mitochondrial OS=Homo sapiens OX=9606 GN=ATP5MF PE=1 SV=3	11025	21	25,5
23	O43678	NADH dehydrogenase [ubiquinone] 1 alpha subcomplex subunit 2 OS=Homo sapiens OX=9606 GN=NDUFA2 PE=1 SV=3	11029	6	40,4
115	L0R6Q1	SLC35A4 upstream open reading frame protein OS=Homo sapiens OX=9606 GN=SLC35A4 PE=3 SV=1	11183	2	14,6
66	P04080	Cystatin-B OS=Homo sapiens OX=9606 GN=CSTB PE=1 SV=2	11190	3	24,5
62	P62805	Histone H4 OS=Homo sapiens OX=9606 GN=H4C1 PE=1 SV=2	11360	6	42,7
131	P81605	Dermcidin OS=Homo sapiens OX=9606 GN=DCD PE=1 SV=2	11391	1	10
125	O43676	NADH dehydrogenase [ubiquinone] 1 beta subcomplex subunit 3 OS=Homo sapiens OX=9606 GN=NDUFB3 PE=1 SV=3	11395	1	10,2
25	Q86SG5	Protein S100-A7A OS=Homo sapiens OX=9606 GN=S100A7A PE=1 SV=3	11412	26	21,8
3	O75964	ATP synthase subunit g, mitochondrial OS=Homo sapiens OX=9606 GN=ATP5MG PE=1 SV=3	11421	67	78,6
46	P82909	Alpha-ketoglutarate dehydrogenase component 4 OS=Homo sapiens OX=9606 GN=MRPS36 PE=1 SV=2	11459	5	57,3
13	P31151	Protein S100-A7 OS=Homo sapiens OX=9606 GN=S100A7 PE=1 SV=4	11578	33	42,6
16	P05387	60S acidic ribosomal protein P2 OS=Homo sapiens OX=9606 GN=RPLP2 PE=1 SV=1	11658	9	55,7
11	P99999	Cytochrome c OS=Homo sapiens OX=9606 GN=CYCS PE=1 SV=2	11855	19	54,3
116	P62942	Peptidyl-prolyl cis-trans isomerase FKBP1A OS=Homo sapiens OX=9606 GN=FKBP1A PE=1 SV=2	12000	1	12
99	Q5U5X0	Complex III assembly factor LYRM7 OS=Homo sapiens OX=9606 GN=LYRM7 PE=1 SV=1	12004	1	9,6
47	P10599	Thioredoxin OS=Homo sapiens OX=9606 GN=TXN PE=1 SV=3	12015	4	21
152	P35754	Glutaredoxin-1 OS=Homo sapiens OX=9606 GN=GLRX PE=1 SV=2	12053	2	10,4
146	Q9BV81	ER membrane protein complex subunit 6 OS=Homo sapiens OX=9606 GN=EMC6 PE=1 SV=1	12067	1	10,9
10	Q9UII2	ATPase inhibitor, mitochondrial OS=Homo sapiens OX=9606 GN=ATP5IF1 PE=1 SV=1	12241	49	27,4
68	Q9BQC6	Ribosomal protein 63, mitochondrial OS=Homo sapiens OX=9606 GN=MRPL57 PE=1 SV=1	12259	4	19,6
50	Q96EL3	39S ribosomal protein L53, mitochondrial OS=Homo sapiens OX=9606 GN=MRPL53 PE=1 SV=1	12270	5	22,3
132	Q9NZ45	CDGSH iron-sulfur domain-containing protein 1 OS=Homo sapiens OX=9606 GN=CISD1 PE=1 SV=1	12362	1	12
52	Q9GZT3	SRA stem-loop-interacting RNA-binding protein, mitochondrial OS=Homo sapiens OX=9606 GN=SLIRP PE=1 SV=1	12398	11	40,4
92	Q96DA6	Mitochondrial import inner membrane translocase subunit TIM14 OS=Homo sapiens OX=9606 GN=DNAJC19 PE=1 SV=3	12491	2	19,8
5	P18859	ATP synthase-coupling factor 6, mitochondrial OS=Homo sapiens OX=9606 GN=ATP5PF PE=1 SV=1	12580	25	62

42	O95182	NADH dehydrogenase [ubiquinone] 1 alpha subcomplex subunit 7 OS=Homo sapiens OX=9606 GN=NDUFA7 PE=1 SV=3	12601	3	24,8
150	Q9Y291	28S ribosomal protein S33, mitochondrial OS=Homo sapiens OX=9606 GN=MRPS33 PE=1 SV=1	12621	4	31,1
117	P14174	Macrophage migration inhibitory factor OS=Homo sapiens OX=9606 GN=MIF PE=1 SV=4	12639	1	9,6
67	P61803	Dolichyl-diphosphooligosaccharide--protein glycosyltransferase subunit DAD1 OS=Homo sapiens OX=9606 GN=DAD1 PE=1 SV=3	12660	3	19,5
80	O14548	Cytochrome c oxidase subunit 7A-related protein, mitochondrial OS=Homo sapiens OX=9606 GN=COX7A2L PE=1 SV=2	12664	1	11,4
133	O75368	SH3 domain-binding glutamic acid-rich-like protein OS=Homo sapiens OX=9606 GN=SH3BGRL PE=1 SV=1	12766	3	19,3
82	P30046	D-dopachrome decarboxylase OS=Homo sapiens OX=9606 GN=DDT PE=1 SV=3	12818	3	16,1
88	P41567	Eukaryotic translation initiation factor 1 OS=Homo sapiens OX=9606 GN=EIF1 PE=1 SV=1	12839	1	12,4
76	Q96HJ9	Protein FMC1 homolog OS=Homo sapiens OX=9606 GN=FMC1 PE=1 SV=2	12855	3	20,4
20	P06702	Protein S100-A9 OS=Homo sapiens OX=9606 GN=S100A9 PE=1 SV=1	13291	7	43,9
89	Q16864	V-type proton ATPase subunit F OS=Homo sapiens OX=9606 GN=ATP6V1F PE=1 SV=2	13362	4	33,6
110	P60866	40S ribosomal protein S20 OS=Homo sapiens OX=9606 GN=RPS20 PE=1 SV=1	13478	3	10,1
49	Q16718	NADH dehydrogenase [ubiquinone] 1 alpha subcomplex subunit 5 OS=Homo sapiens OX=9606 GN=NDUFA5 PE=1 SV=3	13507	5	17,2
69	P14927	Cytochrome b-c1 complex subunit 7 OS=Homo sapiens OX=9606 GN=UQCRB PE=1 SV=2	13522	5	36,9
84	P0C0S5	Histone H2A.Z OS=Homo sapiens OX=9606 GN=H2AZ1 PE=1 SV=2	13545	2	7
87	A0A2R8Y619	Histone H2B type 2-K1 OS=Homo sapiens OX=9606 GN=H2BK1 PE=3 SV=1	13597	2	7,4
130	Q9UHA4	Ragulator complex protein LAMTOR3 OS=Homo sapiens OX=9606 GN=LAMTOR3 PE=1 SV=1	13671	2	8,1
63	Q96BP2	Coiled-coil-helix-coiled-coil-helix domain-containing protein 1 OS=Homo sapiens OX=9606 GN=CHCHD1 PE=1 SV=1	13694	4	13,6
127	P62851	40S ribosomal protein S25 OS=Homo sapiens OX=9606 GN=RPS25 PE=1 SV=1	13791	1	7,2
34	Q9Y3D7	Mitochondrial import inner membrane translocase subunit TIM16 OS=Homo sapiens OX=9606 GN=PAM16 PE=1 SV=2	13816	6	39,2
141	Q9Y237	Peptidyl-prolyl cis-trans isomerase NIMA-interacting 4 OS=Homo sapiens OX=9606 GN=PIN4 PE=1 SV=1	13858	1	9,2
53	O75348	V-type proton ATPase subunit G 1 OS=Homo sapiens OX=9606 GN=ATP6V1G1 PE=1 SV=3	13863	5	16,9
56	P10606	Cytochrome c oxidase subunit 5B, mitochondrial OS=Homo sapiens OX=9606 GN=COX5B PE=1 SV=2	13915	5	38
161	P62318	Small nuclear ribonucleoprotein Sm D3 OS=Homo sapiens OX=9606 GN=SNRPD3 PE=1 SV=1	14021	1	7,1
103	O95298	NADH dehydrogenase [ubiquinone] 1 subunit C2 OS=Homo sapiens OX=9606 GN=NDUFC2 PE=1 SV=1	14235	3	24,4
135	P82932	28S ribosomal protein S6, mitochondrial OS=Homo sapiens OX=9606 GN=MRPS6 PE=1 SV=3	14275	5	22,4
163	O14737	Programmed cell death protein 5 OS=Homo sapiens OX=9606 GN=PDCD5 PE=1 SV=3	14276	1	10,4
83	P62861	FAU ubiquitin-like and ribosomal protein S30 OS=Homo sapiens OX=9606 GN=FAU PE=1 SV=2	14438	2	7,5
105	P05496	ATP synthase F(0) complex subunit C1, mitochondrial OS=Homo sapiens OX=9606 GN=ATP5MC1 PE=1 SV=2	14439	4	27,9
104	Q5JTJ3	Cytochrome c oxidase assembly factor 6 homolog OS=Homo sapiens OX=9606 GN=COA6 PE=1 SV=1	14449	1	11,2
118	Q8N6L1	Keratinocyte-associated protein 2 OS=Homo sapiens OX=9606 GN=KRTCAP2 PE=1 SV=2	14840	1	12,5
75	Q9NPJ3	Acyl-coenzyme A thioesterase 13 OS=Homo sapiens OX=9606 GN=ACOT13 PE=1 SV=1	15065	3	15,7
14	Q7Z7F7	39S ribosomal protein L55, mitochondrial OS=Homo sapiens OX=9606 GN=MRPL55 PE=1 SV=1	15119	9	37,5
166	P47929	Galectin-7 OS=Homo sapiens OX=9606 GN=LGALS7 PE=1 SV=2	15123	1	8,1
109	P56556	NADH dehydrogenase [ubiquinone] 1 alpha subcomplex subunit 6 OS=Homo sapiens OX=9606 GN=NDUFA6 PE=1 SV=4	15127	5	25
147	Q4U2R6	39S ribosomal protein L51, mitochondrial OS=Homo sapiens OX=9606 GN=MRPL51 PE=1 SV=1	15199	2	14,8
21	P07737	Profilin-1 OS=Homo sapiens OX=9606 GN=PFN1 PE=1 SV=2	15216	8	42,9

44	O60783	28S ribosomal protein S14, mitochondrial OS=Homo sapiens OX=9606 GN=MRPS14 PE=1 SV=1	15243	4	21,9
22	O95168	NADH dehydrogenase [ubiquinone] 1 beta subcomplex subunit 4 OS=Homo sapiens OX=9606 GN=NDUFB4 PE=1 SV=3	15256	6	31,8
26	Q8IXM3	39S ribosomal protein L41, mitochondrial OS=Homo sapiens OX=9606 GN=MRPL41 PE=1 SV=1	15430	6	33,6
78	Q01469	Fatty acid-binding protein 5 OS=Homo sapiens OX=9606 GN=FABP5 PE=1 SV=3	15497	7	19,3
164	O15235	28S ribosomal protein S12, mitochondrial OS=Homo sapiens OX=9606 GN=MRPS12 PE=1 SV=1	15562	1	5,8
72	Q9Y3D3	28S ribosomal protein S16, mitochondrial OS=Homo sapiens OX=9606 GN=MRPS16 PE=1 SV=1	15563	2	19
58	Q5T1J5	Putative coiled-coil-helix-coiled-coil-helix domain-containing protein CHCHD2P9, mitochondrial OS=Homo sapiens OX=9606 GN=CHCHD2P9 PE=5 SV=1	15708	2	15,9
107	P26885	Peptidyl-prolyl cis-trans isomerase FKBP2 OS=Homo sapiens OX=9606 GN=FKBP2 PE=1 SV=2	15810	1	8,5
136	Q6P161	39S ribosomal protein L54, mitochondrial OS=Homo sapiens OX=9606 GN=MRPL54 PE=1 SV=1	15866	1	7,2
18	Q9NZT1	Calmodulin-like protein 5 OS=Homo sapiens OX=9606 GN=CALML5 PE=1 SV=2	15883	6	21,2
51	Q9P0M9	39S ribosomal protein L27, mitochondrial OS=Homo sapiens OX=9606 GN=MRPL27 PE=1 SV=1	16120	3	20,9
31	Q6P1L8	39S ribosomal protein L14, mitochondrial OS=Homo sapiens OX=9606 GN=MRPL14 PE=1 SV=1	16165	7	31,7
101	Q9Y3D5	28S ribosomal protein S18c, mitochondrial OS=Homo sapiens OX=9606 GN=MRPS18C PE=1 SV=1	16238	4	12,7
30	Q86SX6	Glutaredoxin-related protein 5, mitochondrial OS=Homo sapiens OX=9606 GN=GLRX5 PE=1 SV=2	16732	4	28
162	Q4VC31	Protein MIX23 OS=Homo sapiens OX=9606 GN=MIX23 PE=1 SV=1	16838	1	6,2
27	P20674	Cytochrome c oxidase subunit 5A, mitochondrial OS=Homo sapiens OX=9606 GN=COX5A PE=1 SV=2	16923	7	34
55	Q9Y6G3	39S ribosomal protein L42, mitochondrial OS=Homo sapiens OX=9606 GN=MRPL42 PE=1 SV=1	16935	4	21,8
91	P61626	Lysozyme C OS=Homo sapiens OX=9606 GN=LYZ PE=1 SV=1	16982	1	8,1
74	P60660	Myosin light polypeptide 6 OS=Homo sapiens OX=9606 GN=MYL6 PE=1 SV=2	17090	4	28,5
15	Q9BX68	Adenosine 5~-monophosphoramidase HINT2 OS=Homo sapiens OX=9606 GN=HINT2 PE=1 SV=1	17208	9	35
70	P62277	40S ribosomal protein S13 OS=Homo sapiens OX=9606 GN=RPS13 PE=1 SV=2	17212	2	7,9
168	Q04837	Single-stranded DNA-binding protein, mitochondrial OS=Homo sapiens OX=9606 GN=SSBP1 PE=1 SV=1	17249	2	17,6
1	P30049	ATP synthase subunit delta, mitochondrial OS=Homo sapiens OX=9606 GN=ATP5F1D PE=1 SV=2	17479	109	51,2
41	O14561	Acyl carrier protein, mitochondrial OS=Homo sapiens OX=9606 GN=NDUFAB1 PE=1 SV=3	17577	6	14,7
38	P62979	Ubiquitin-40S ribosomal protein S27a OS=Homo sapiens OX=9606 GN=RPS27A PE=1 SV=2	18296	10	24,4
40	Q8N5N7	39S ribosomal protein L50, mitochondrial OS=Homo sapiens OX=9606 GN=MRPL50 PE=1 SV=2	18484	7	26,6
96	O75947	ATP synthase subunit d, mitochondrial OS=Homo sapiens OX=9606 GN=ATP5PD PE=1 SV=3	18537	1	5,6
159	P46783	40S ribosomal protein S10 OS=Homo sapiens OX=9606 GN=RPS10 PE=1 SV=1	18886	3	20
43	Q969H8	Myeloid-derived growth factor OS=Homo sapiens OX=9606 GN=MYDGF PE=1 SV=1	18897	8	33,5
37	Q13405	39S ribosomal protein L49, mitochondrial OS=Homo sapiens OX=9606 GN=MRPL49 PE=1 SV=1	19243	6	37,3
114	Q96EL2	28S ribosomal protein S24, mitochondrial OS=Homo sapiens OX=9606 GN=MRPS24 PE=1 SV=1	19345	1	4,8
169	P13073	Cytochrome c oxidase subunit 4 isoform 1, mitochondrial OS=Homo sapiens OX=9606 GN=COX4I1 PE=1 SV=1	19621	1	6,5
71	Q9BU61	NADH dehydrogenase [ubiquinone] 1 alpha subcomplex assembly factor 3 OS=Homo sapiens OX=9606 GN=NDUFAF3 PE=1 SV=1	20566	2	12
65	P52815	39S ribosomal protein L12, mitochondrial OS=Homo sapiens OX=9606 GN=MRPL12 PE=1 SV=2	21563	4	17,2
90	Q9BYC8	39S ribosomal protein L32, mitochondrial OS=Homo sapiens OX=9606 GN=MRPL32 PE=1 SV=1	21733	2	12,2
148	P30044	Peroxiredoxin-5, mitochondrial OS=Homo sapiens OX=9606 GN=PRDX5 PE=1 SV=4	22301	2	3,7
94	P04792	Heat shock protein beta-1 OS=Homo sapiens OX=9606 GN=HSPB1 PE=1 SV=2	22826	1	7,8

59	P31944	Caspase-14 OS=Homo sapiens OX=9606 GN=CASP14 PE=1 SV=2	27947	5	13,6
134	Q04917	14-3-3 protein eta OS=Homo sapiens OX=9606 GN=YWHAH PE=1 SV=4	28372	1	4,1
126	P0C7P4	Putative cytochrome b-c1 complex subunit Rieske-like protein 1 OS=Homo sapiens OX=9606 GN=UQCRFS1P1 PE=5 SV=1	31081	2	4,6
129	P29692	Elongation factor 1-delta OS=Homo sapiens OX=9606 GN=EEF1D PE=1 SV=5	31217	1	4,3
86	P35030	Trypsin-3 OS=Homo sapiens OX=9606 GN=PRSS3 PE=1 SV=2	33306	3	3,3
138	Q9H0C2	ADP/ATP translocase 4 OS=Homo sapiens OX=9606 GN=SLC25A31 PE=1 SV=1	35285	2	3,8
145	P04406	Glyceraldehyde-3-phosphate dehydrogenase OS=Homo sapiens OX=9606 GN=GAPDH PE=1 SV=3	36201	3	6,3
64	P60709	Actin, cytoplasmic 1 OS=Homo sapiens OX=9606 GN=ACTB PE=1 SV=1	42052	3	8,8
17	Q8NC51	Plasminogen activator inhibitor 1 RNA-binding protein OS=Homo sapiens OX=9606 GN=SERBP1 PE=1 SV=2	44995	6	7,8
113	Q92665	28S ribosomal protein S31, mitochondrial OS=Homo sapiens OX=9606 GN=MRPS31 PE=1 SV=3	45405	2	4,6
79	Q8IW75	Serpin A12 OS=Homo sapiens OX=9606 GN=SERPINA12 PE=1 SV=1	47259	2	2,2
137	Q6E0U4	Dermokine OS=Homo sapiens OX=9606 GN=DMKN PE=1 SV=3	47282	1	1,5
73	Q05639	Elongation factor 1-alpha 2 OS=Homo sapiens OX=9606 GN=EEF1A2 PE=1 SV=1	50780	3	8,6
54	P02533	Keratin, type I cytoskeletal 14 OS=Homo sapiens OX=9606 GN=KRT14 PE=1 SV=4	51872	8	11,4
149	P12268	Inosine-5~-monophosphate dehydrogenase 2 OS=Homo sapiens OX=9606 GN=IMPDH2 PE=1 SV=2	56226	1	2,1
61	P06576	ATP synthase subunit beta, mitochondrial OS=Homo sapiens OX=9606 GN=ATP5F1B PE=1 SV=3	56525	3	7
97	Q58FF6	Putative heat shock protein HSP 90-beta 4 OS=Homo sapiens OX=9606 GN=HSP90AB4P PE=5 SV=1	58855	1	2,4
35	P13645	Keratin, type I cytoskeletal 10 OS=Homo sapiens OX=9606 GN=KRT10 PE=1 SV=6	59020	13	15,2
160	P22307	Sterol carrier protein 2 OS=Homo sapiens OX=9606 GN=SCP2 PE=1 SV=2	59640	1	2
85	P25705	ATP synthase subunit alpha, mitochondrial OS=Homo sapiens OX=9606 GN=ATP5F1A PE=1 SV=1	59828	1	2,4
48	P02538	Keratin, type II cytoskeletal 6A OS=Homo sapiens OX=9606 GN=KRT6A PE=1 SV=3	60293	7	10,1
39	P04259	Keratin, type II cytoskeletal 6B OS=Homo sapiens OX=9606 GN=KRT6B PE=1 SV=5	60315	7	8,9
24	Q6UWP8	Suprabasin OS=Homo sapiens OX=9606 GN=SBSN PE=1 SV=2	60562	7	11,5
36	P35527	Keratin, type I cytoskeletal 9 OS=Homo sapiens OX=9606 GN=KRT9 PE=1 SV=3	62255	9	12,5
28	P35908	Keratin, type II cytoskeletal 2 epidermal OS=Homo sapiens OX=9606 GN=KRT2 PE=1 SV=2	65678	9	11,6
9	P04264	Keratin, type II cytoskeletal 1 OS=Homo sapiens OX=9606 GN=KRT1 PE=1 SV=6	66170	23	25,8
93	Q9H361	Polyadenylate-binding protein 3 OS=Homo sapiens OX=9606 GN=PABPC3 PE=1 SV=2	70215	2	2,5
155	P11142	Heat shock cognate 71 kDa protein OS=Homo sapiens OX=9606 GN=HSPA8 PE=1 SV=1	71082	1	2
32	P02768	Albumin OS=Homo sapiens OX=9606 GN=ALB PE=1 SV=2	71317	6	3,9
156	Q8TF61	F-box only protein 41 OS=Homo sapiens OX=9606 GN=FBXO41 PE=2 SV=5	95920	1	0,8
167	Q9Y6H5	Synphilin-1 OS=Homo sapiens OX=9606 GN=SNCAIP PE=1 SV=2	101316	2	0,8
140	Q08554	Desmocollin-1 OS=Homo sapiens OX=9606 GN=DSC1 PE=1 SV=2	101406	1	1,5
121	Q02413	Desmoglein-1 OS=Homo sapiens OX=9606 GN=DSG1 PE=1 SV=2	114702	1	1,8
112	Q5D862	Filaggrin-2 OS=Homo sapiens OX=9606 GN=FLG2 PE=1 SV=1	249296	1	0,5
151	P22105	Tenascin-X OS=Homo sapiens OX=9606 GN=TNXB PE=1 SV=5	465114	3	0,2
