# Supplementary material for: ATP synthase interactome analysis identifies a new subunit l as a modulator of permeability transition pore in yeast
Source: Sci Rep. 2023 Mar 7;13:3839. doi: 10.1038/s41598-023-30966-5 (PMC9992712; doi:10.1038/s41598-023-30966-5)
Supplement: Supplementary file 7 — Supplementary Information 7. [file 41598_2023_30966_MOESM7_ESM.docx]

**Supplementary**

**ATP synthase interactome analysis identifies a new subunit *l* as a modulator of permeability transition pore in yeast**

Chiranjit Panja*, Aneta Wiesyk, Katarzyna Niedźwiecka, Emilia Baranowska, Roza Kucharczyk*

Institute of Biochemistry and Biophysics, Polish Academy of Sciences, Warsaw, Poland

**Table of Content Page no.**

Fig. S1 2

Fig. S2 3

Fig. S3 4

Fig. S4 5

Fig. S5 and S6 6

Fig. S7 7

Fig. S8 8

Fig. S9 9

In the resubmitted revision files 10

**(a)**

**Pull down of *S. cerevisiae* ATP synthase by Atp6-HA-His**

**2D-BN-SDS PAGE**

**(b)**


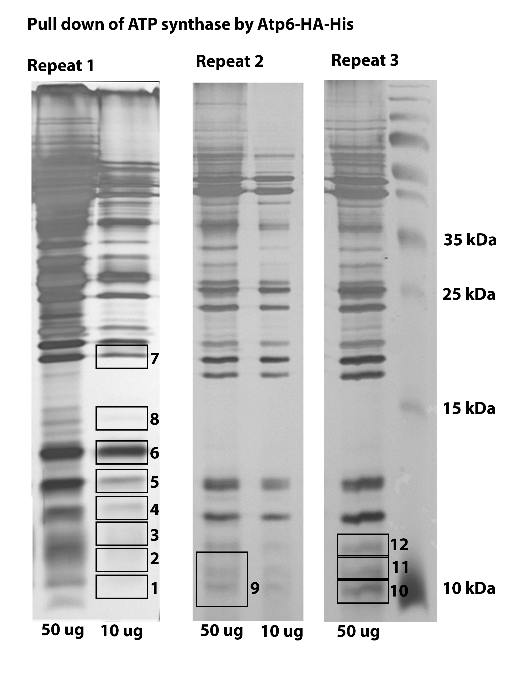


**Human**

***S. cerevisiae* Repeat 2**

***S. cerevisiae* Repeat 1**


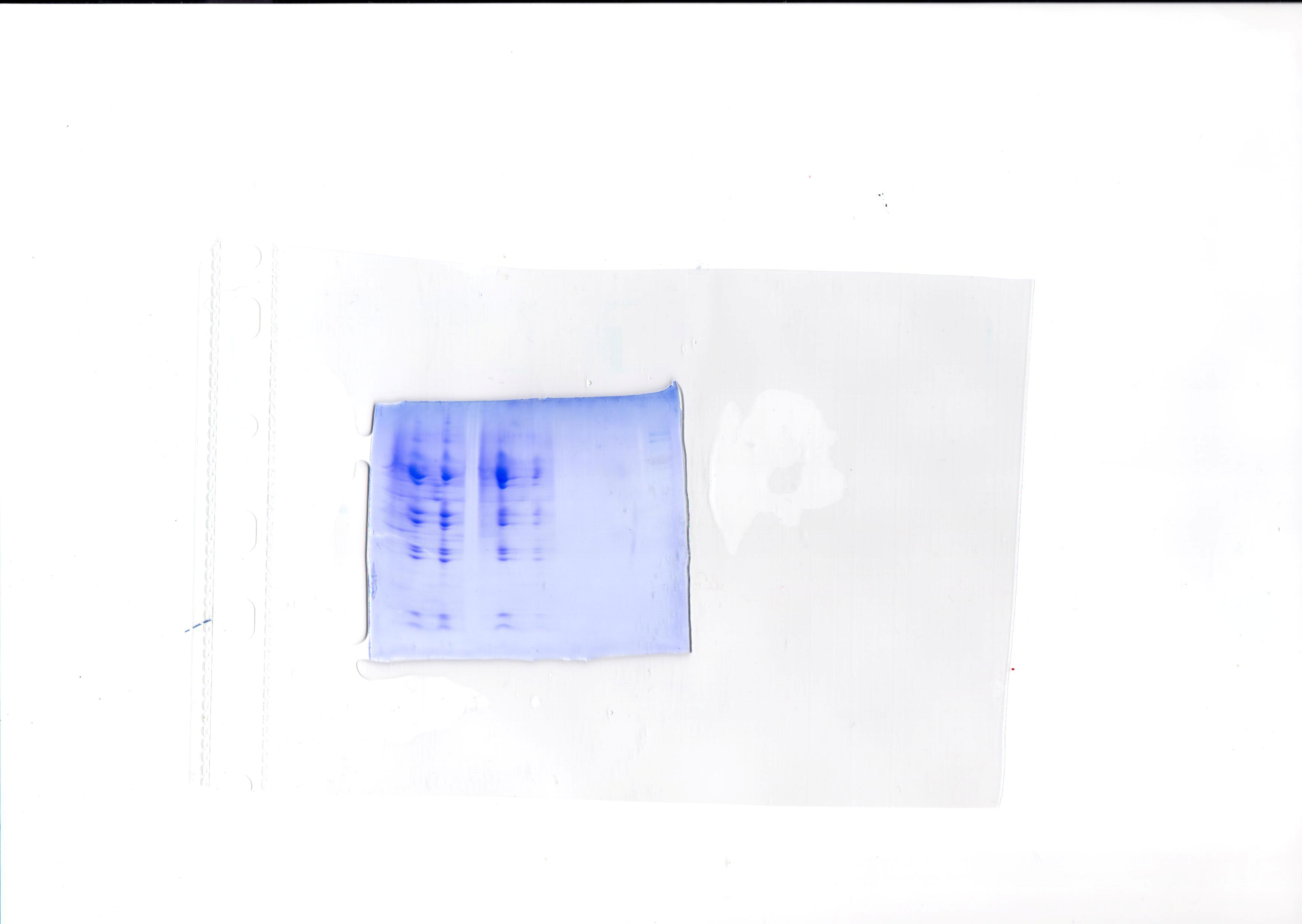

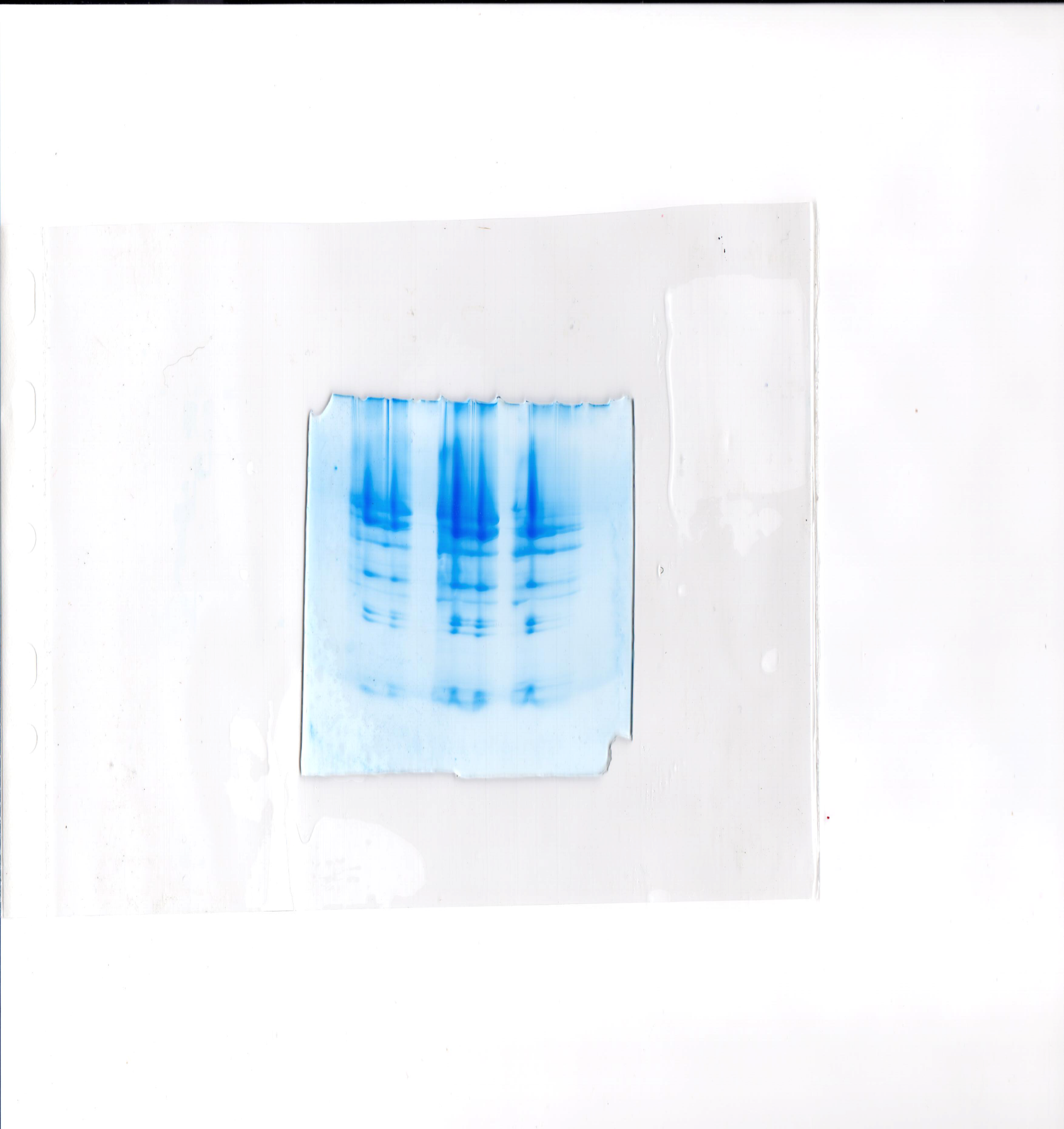


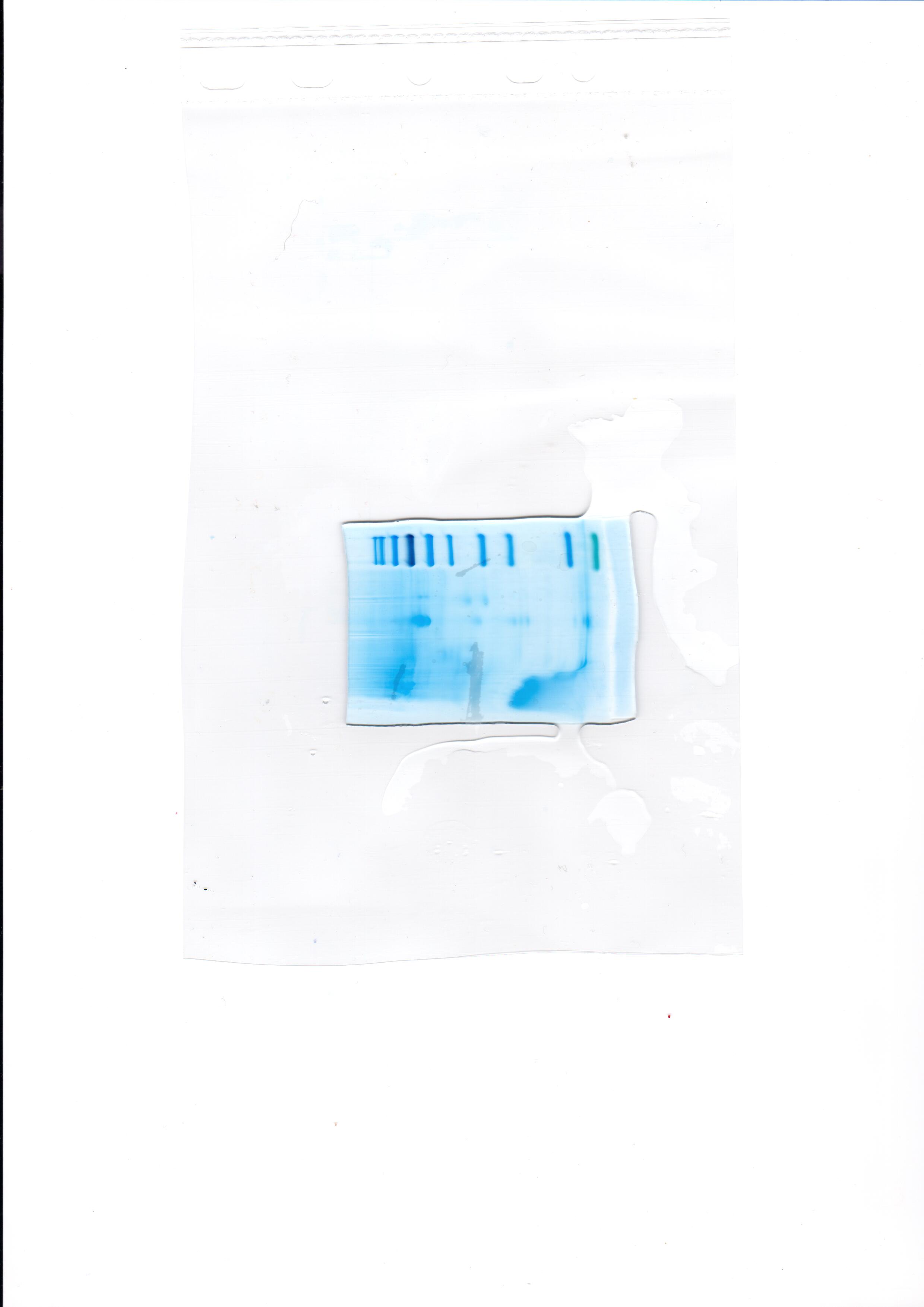


**20**

**19**

**16**

**15**

**14**

**13**

**Fig. S1. a)** Repeat of pulldown experiments and excised gel regions used for mass spectrometric identification. The numbers correspond to the band numbers as described in Supp. Table S1 and S2**. b)** Gel regions used for identification from 2D-BN-SDS-PAGE separation of monomers and dimers. Bands 17 and 18 (repeat 3) were cut from whole monomer and dimer from BN-PAGE and not shown. Figure related to Fig. 1a. Original blots/gels are presented in SupplementaryRowImages pages 9 and 10. See Supplementary Table S1 for details.

**(a)**

**(b)**

**Count**


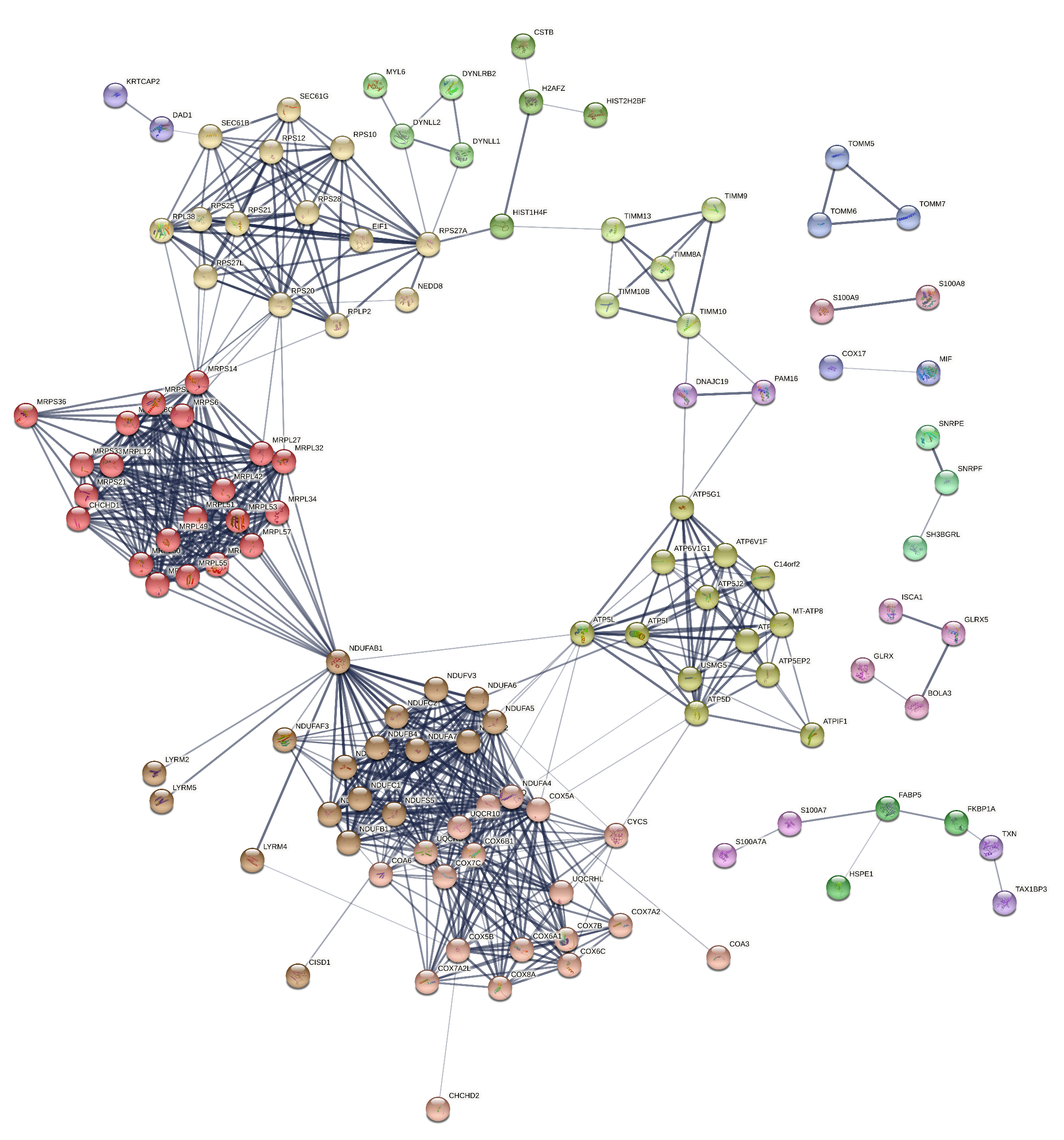

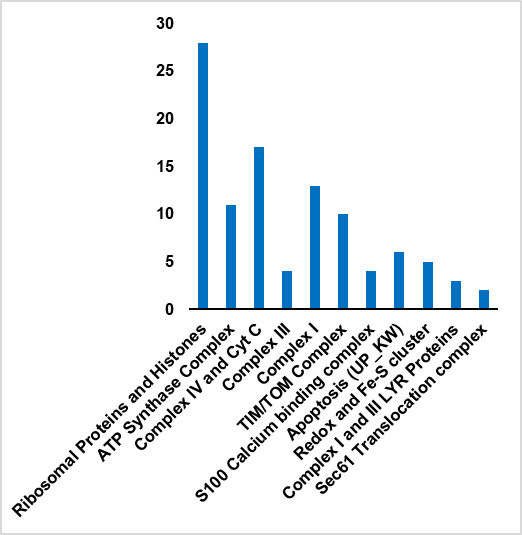


**Count**

**Fig. S2. Human ATP synthase interactome.** Mitochondria were isolated from HEK293T. The monomers and dimers of ATP synthase were extracted by digitonin and separated in two-dimensional BN-SDS-PAGE gel and visualized by Coomassie. Gel pieces from ≤ 20 kDa region from monomer and dimer were cut-off and analyzed by LC/MS analysis. **a)** Interactome was connected into a network using the STRING database. **b)** Classification of the identified proteins manually or by Gene Ontology terms. See Supplementary Table S2 for details.

**(a)**

**Identification of Mco10 from pull down**


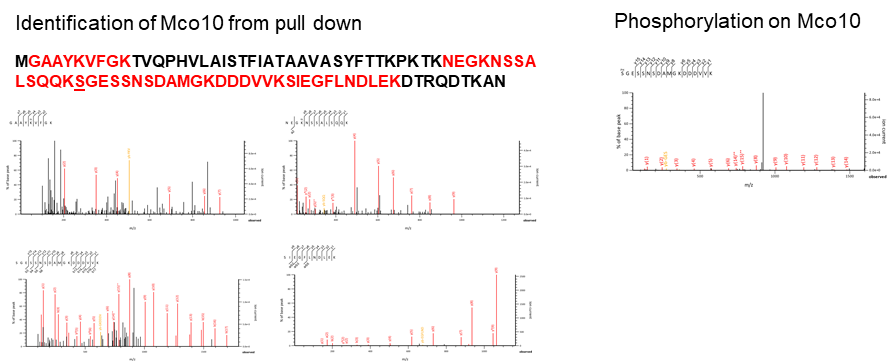


**Sequence Coverage: 57%**


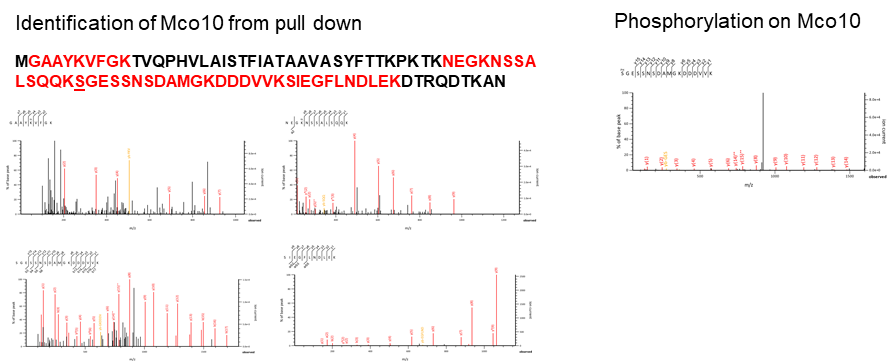


**Fig. S3.** Identification of Mco10 in the ATP synthase interactome from pull down of ATP synthase by ATP6-HA-His. **a)** The LC/MS spectra of four independent Mco10 peptides. Sequence marked in red were identified in the analysis. **b)** Phosphopeptide (phosphorylation at Ser53) of Mco10 identified in the LC/MS analysis. Related to Fig. 1.

**Phosphorylation on Mco10**

**(b)**


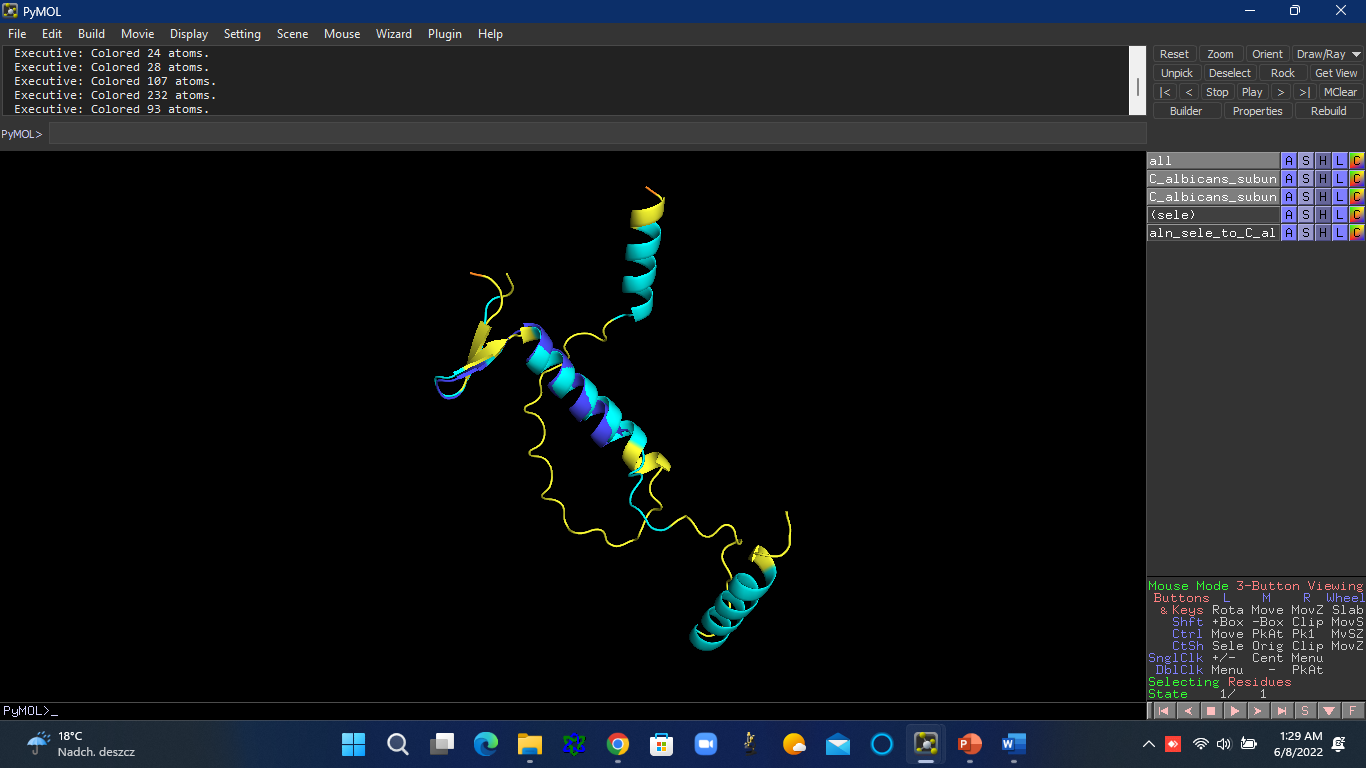


***Candida albicans***


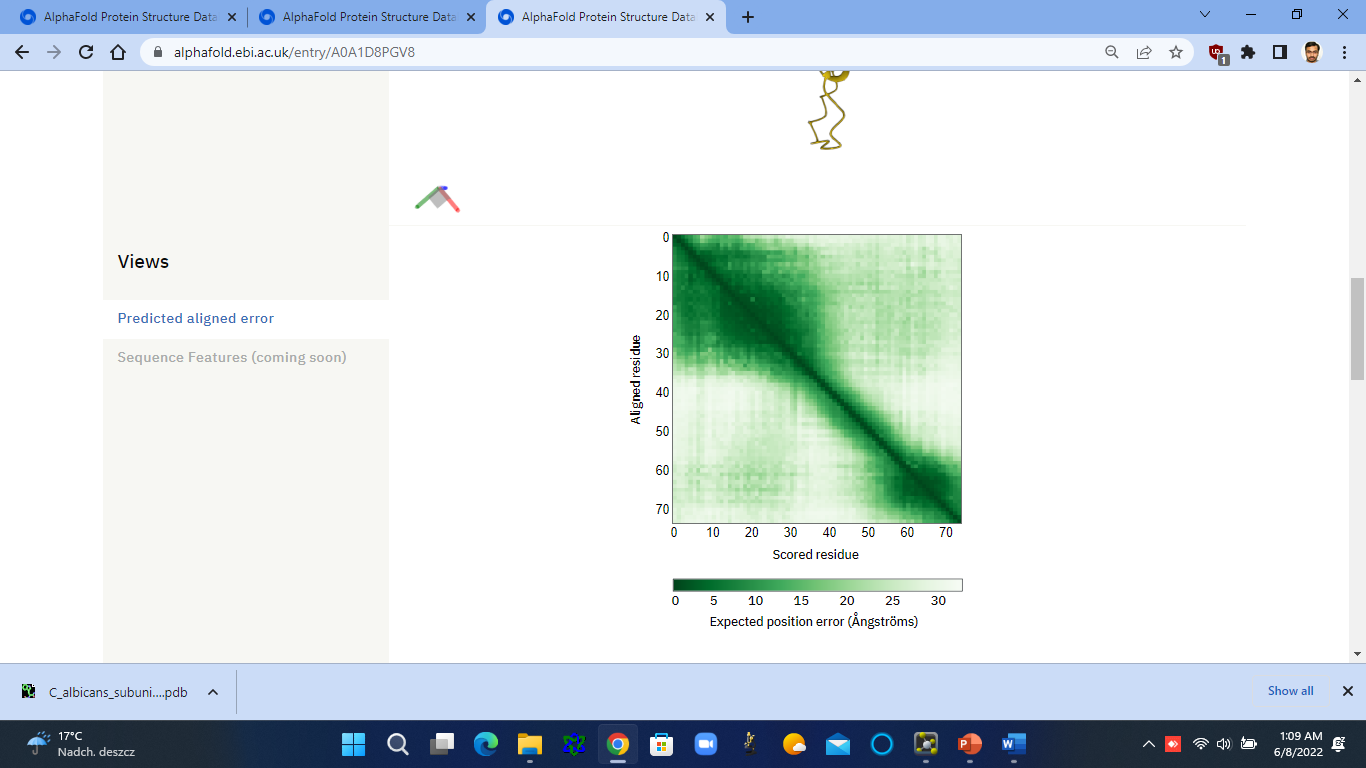


**orf19.909.1**


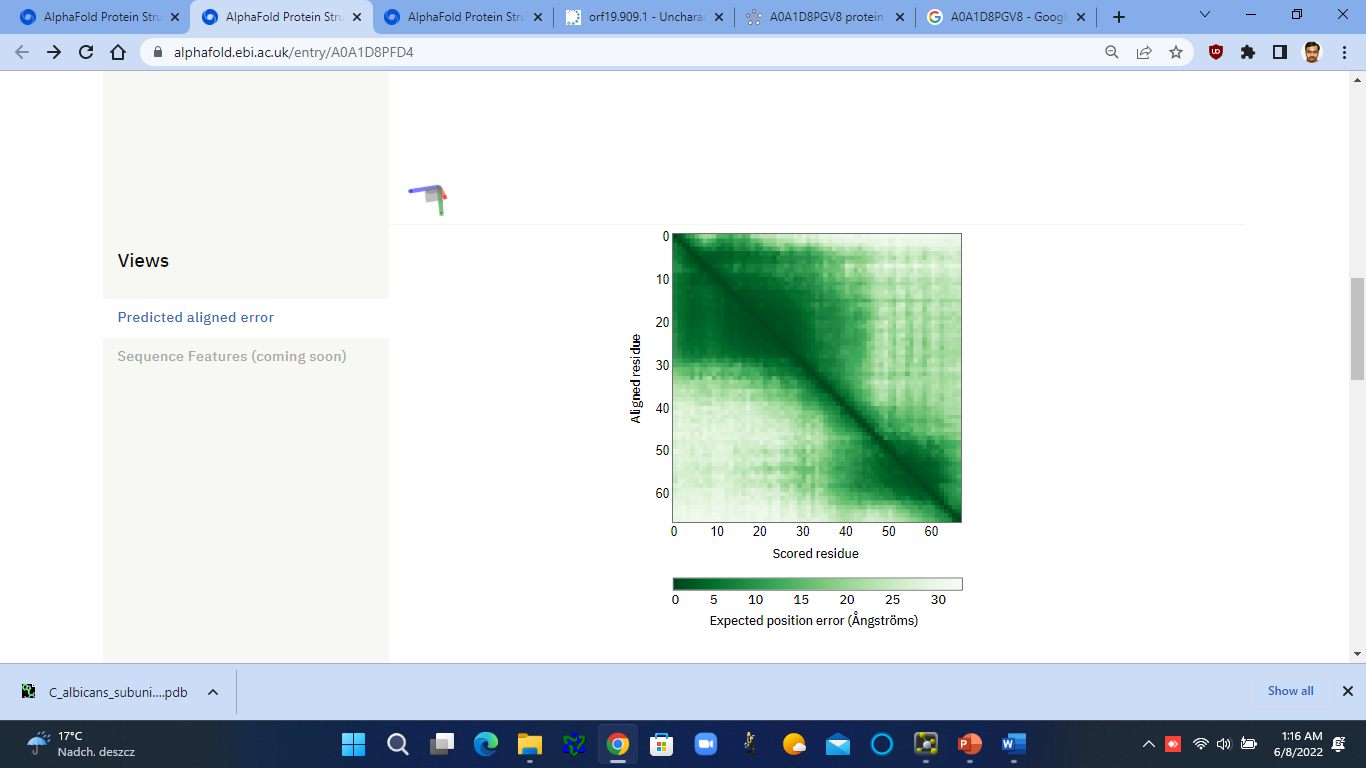


**Atp19**

**orf19.909.1**

**Atp19**

**N-ter**

**C-ter**

**C-ter**

***Pichia angusta***


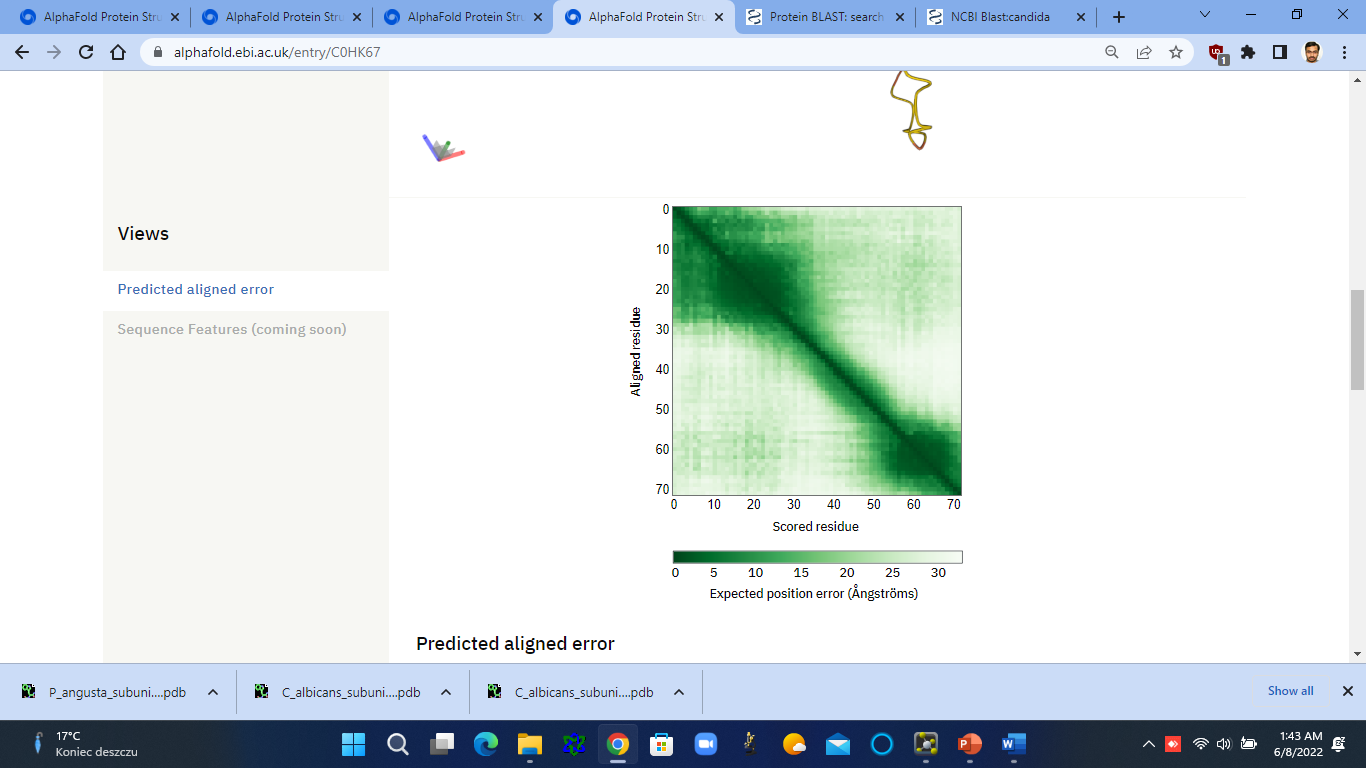


**C0HK67 (ATPLN)**


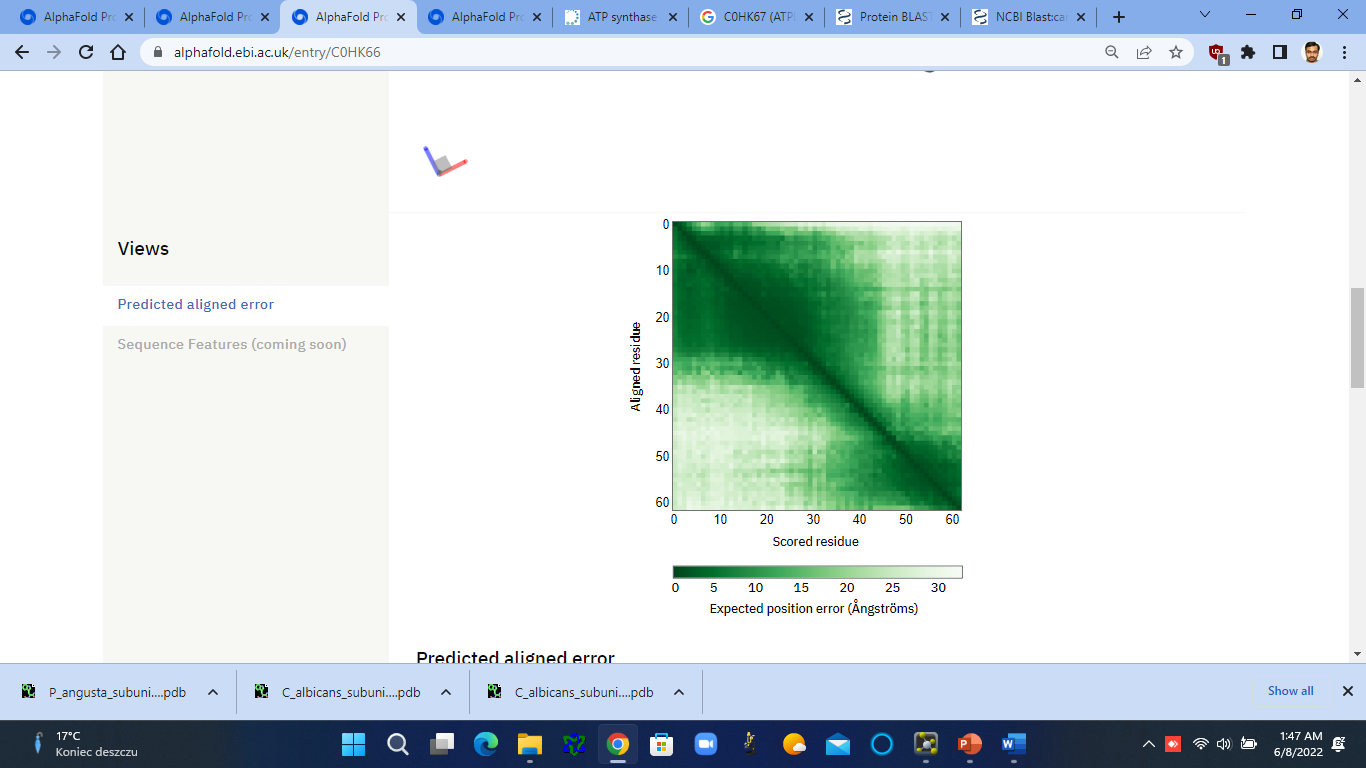


**Atp19**


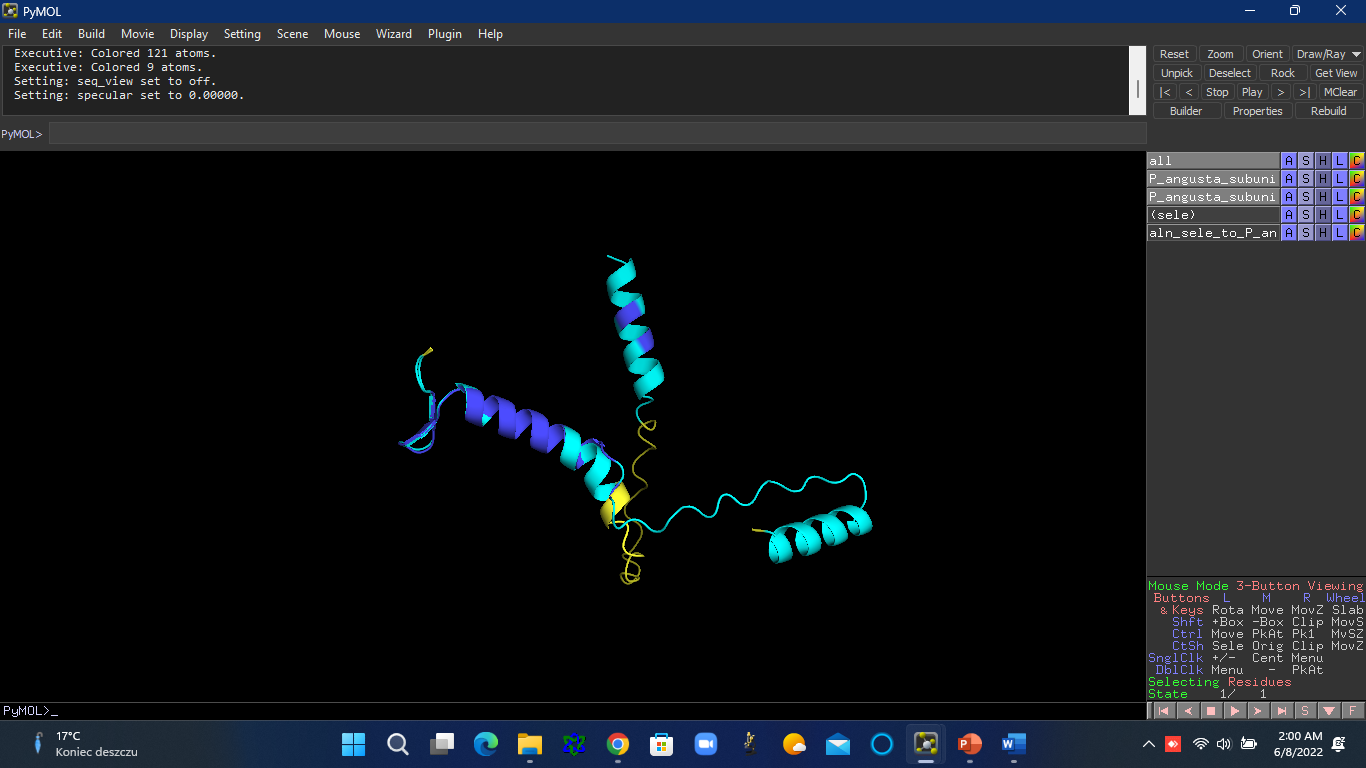


**Atp_L**

**Atp19**

**N-ter**

**C-ter**

**C-ter**


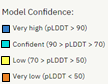

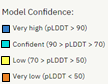


**Fig. S4. Superposition of subunits *k* and *l* from *Candida albicans* and *Pichia angusta****.* The AlphaFold2 predicted structure of Atp19 (subunit *k*) and orf19.909.1 (subunit *l*) of *Candida albicans* were aligned using PyMol software. Color code represents the model confidence and PAE plots of the models are shown below. Homologous proteins from *Pichia angusta* were similarly modelled and visualized in PyMOL. Related to Fig. 2.

**Fig S5.** Sequence alignment of Atp19 and Mco10 homologs in fungi. (1) and (2) represents Mco10 or Atp19 related homolog where two of them were present in the fungal genome. Related to Fig. 2e. See Supplementary Table S3 for details.


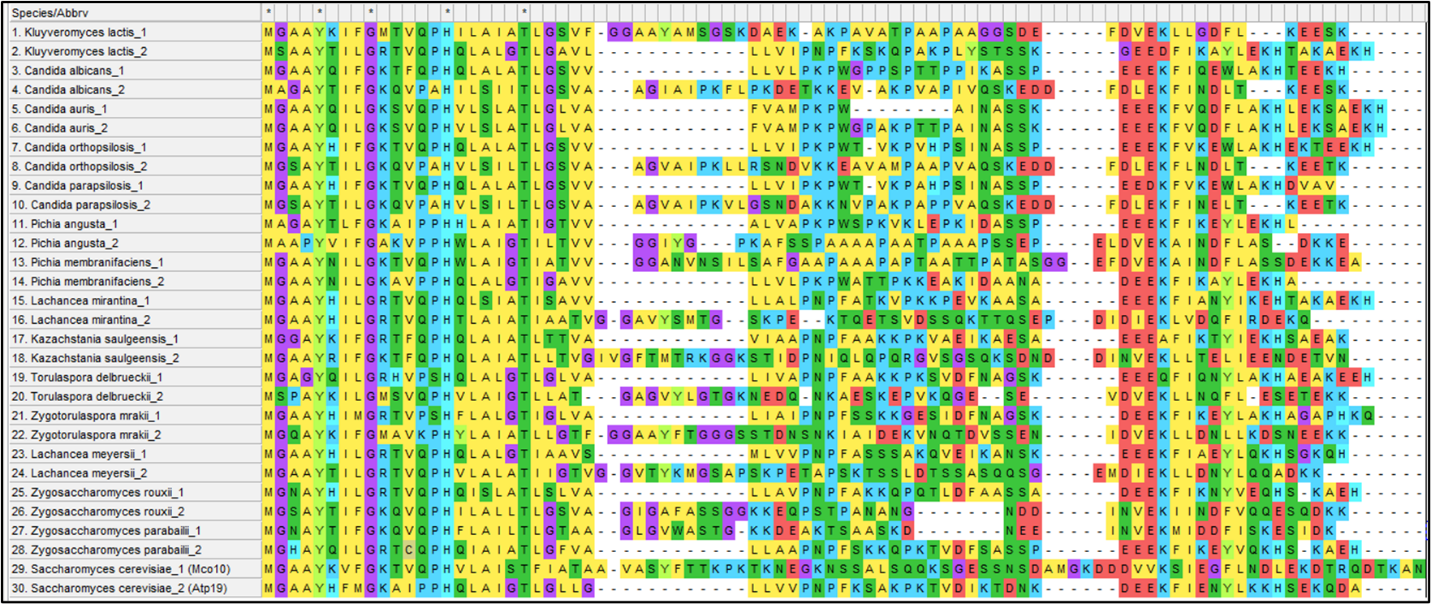


***Δatp19Δmco10***

**(b)**

***Δatp21***

***Δatp20***

***Δmco10***

***Δatp19***

***Wt***

**(a)**

***Wt***

***Δatp19***

***Δmco1a***

***Δatp21***


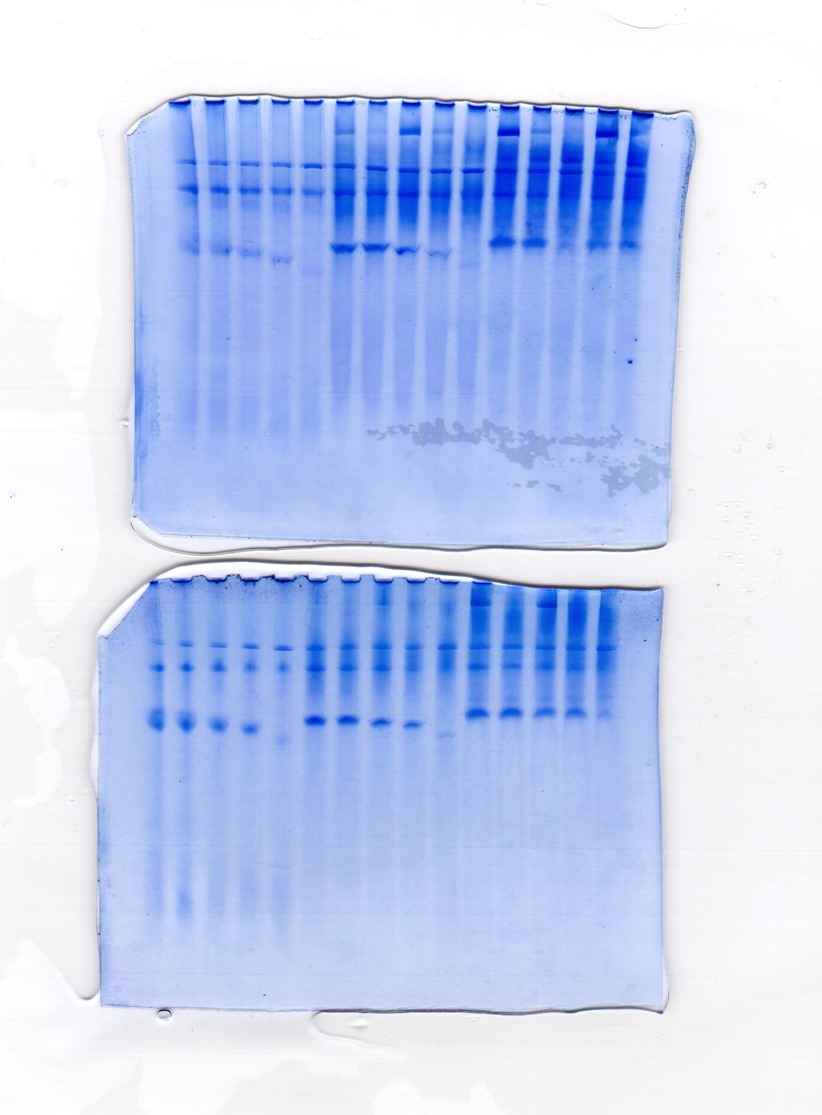

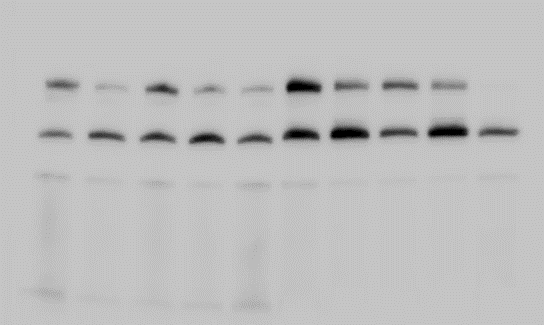

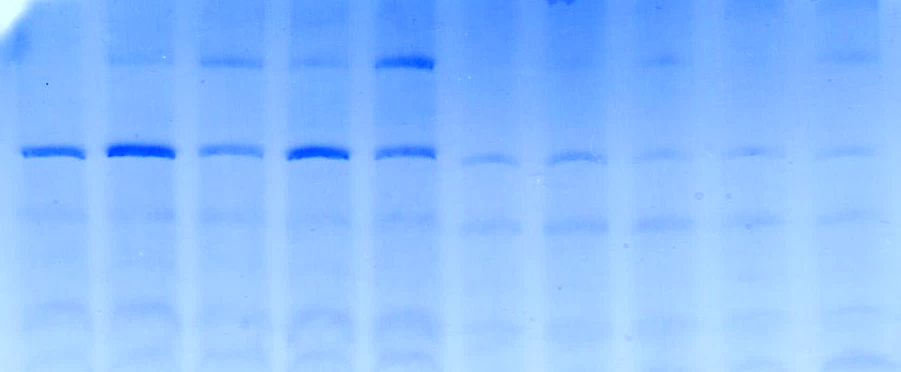


**V2**

**V1**

**F1**

**Atp2**

**V1**

**V2**

**1% Digitonin**

**F1**


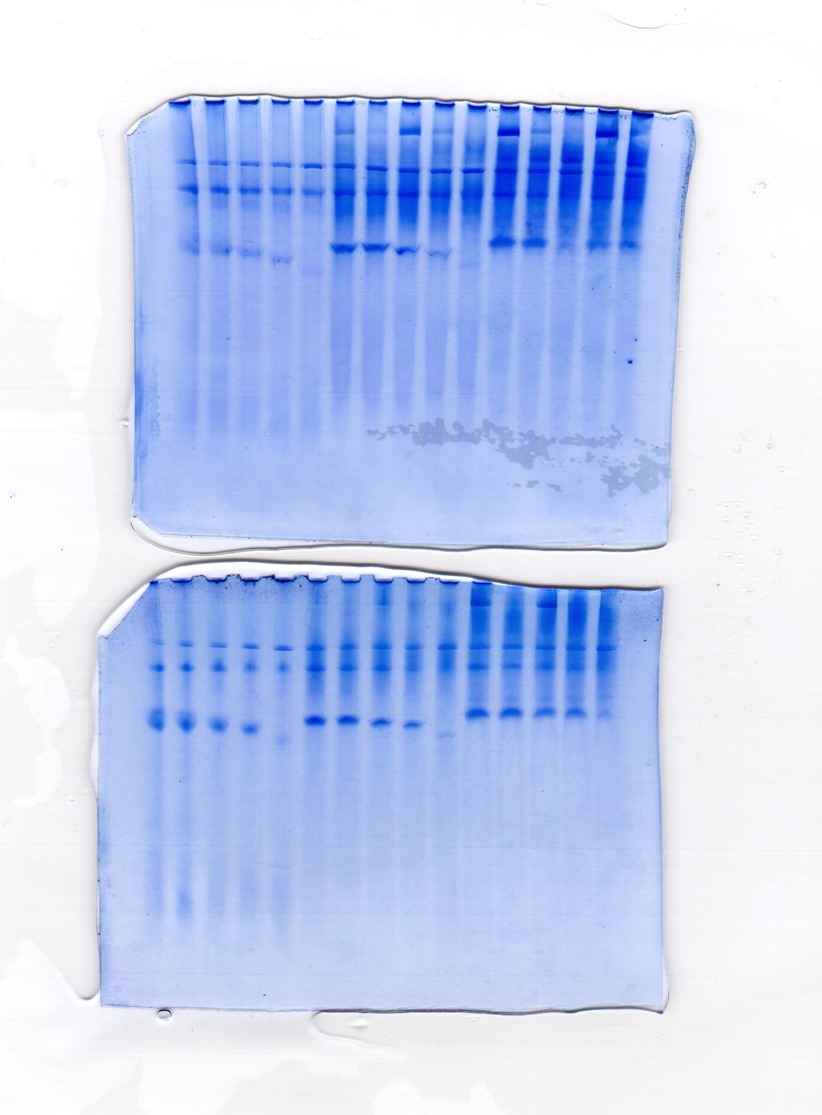


**V2**

**2% Digitonin**

**F1**

**V1**

**Coomassie gel**


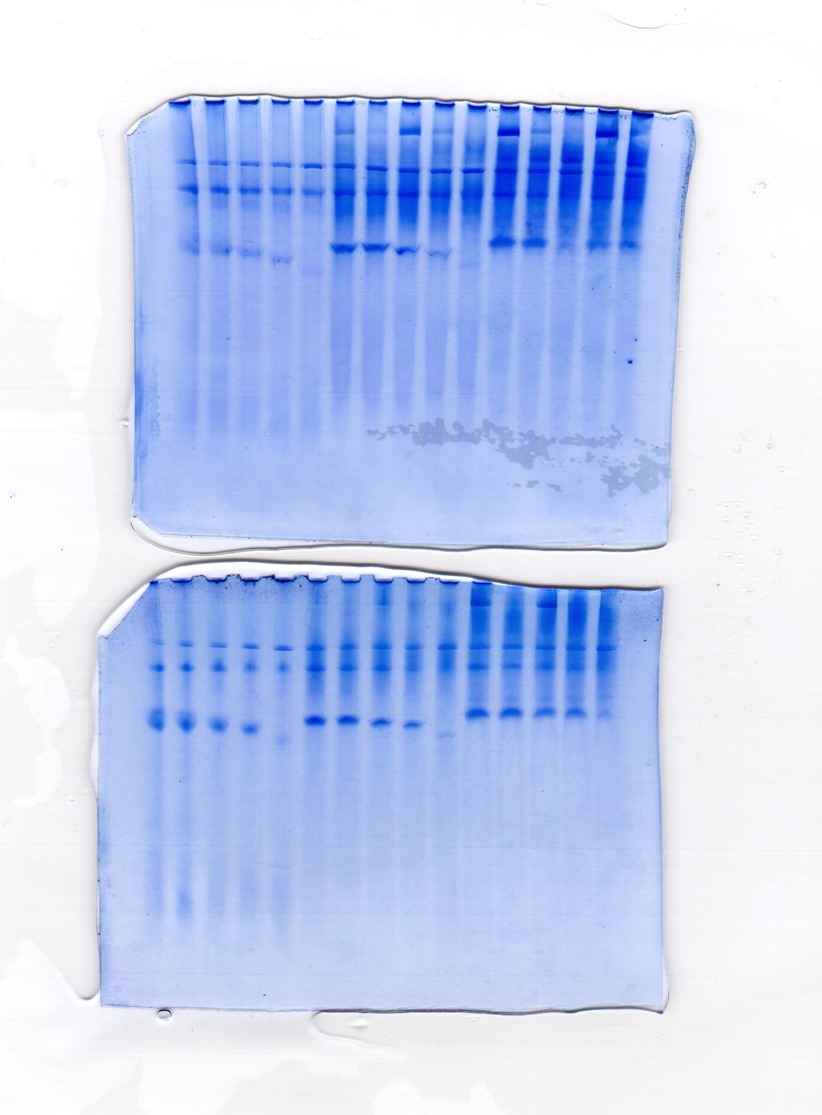


**V2**

**3% Digitonin**

**F1**

**V1**

**Coomassie gel**

**Fig. S6. Mco10 deletion makes ATP synthase more susceptible to digitonin extraction. a)** Monomers and dimers of ATP synthase from the wild type and indicated mutants were extracted by 2 % digitonin and western blot was performed using anti-Atp2 antibody. **b)** Monomers and dimers extracted using 1 %, 2 % or 3 % digitonin and visualized by Coomassie staining. Related to Fig. 5a. Original blots/gels are presented in SupplementaryRowImages page 11.


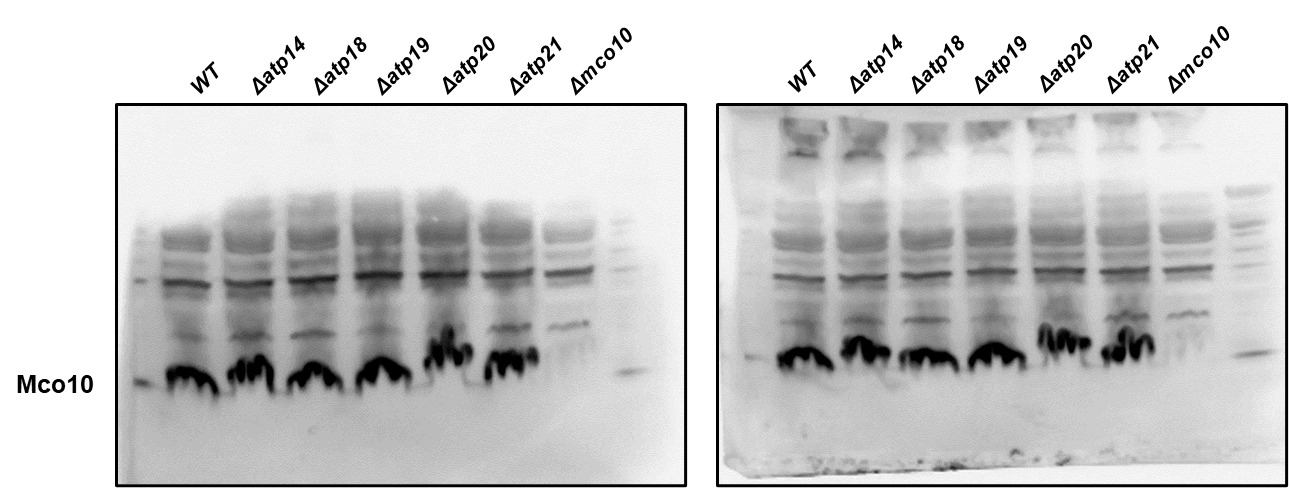


**Mco10**


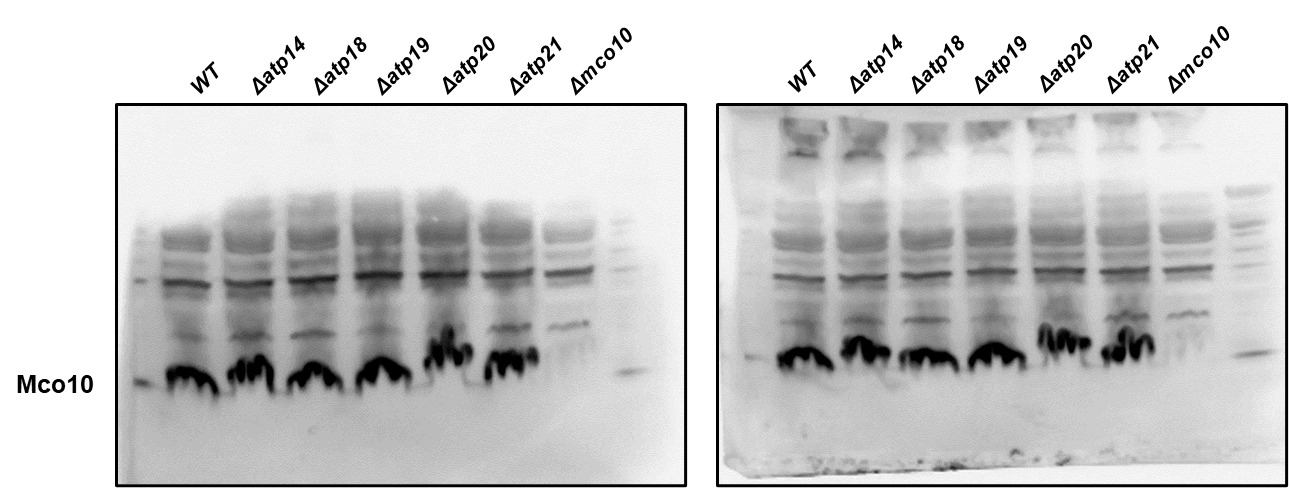


**Mco10**

**Fig. S7.** Steady-state levels of Mco10 in wild type and indicated mutants determined with anti-Mco10 antibody. Two duplicate experiments are shown. Related to Fig. 5b. Original blots/gels are presented in SupplementaryRowImages page 12.


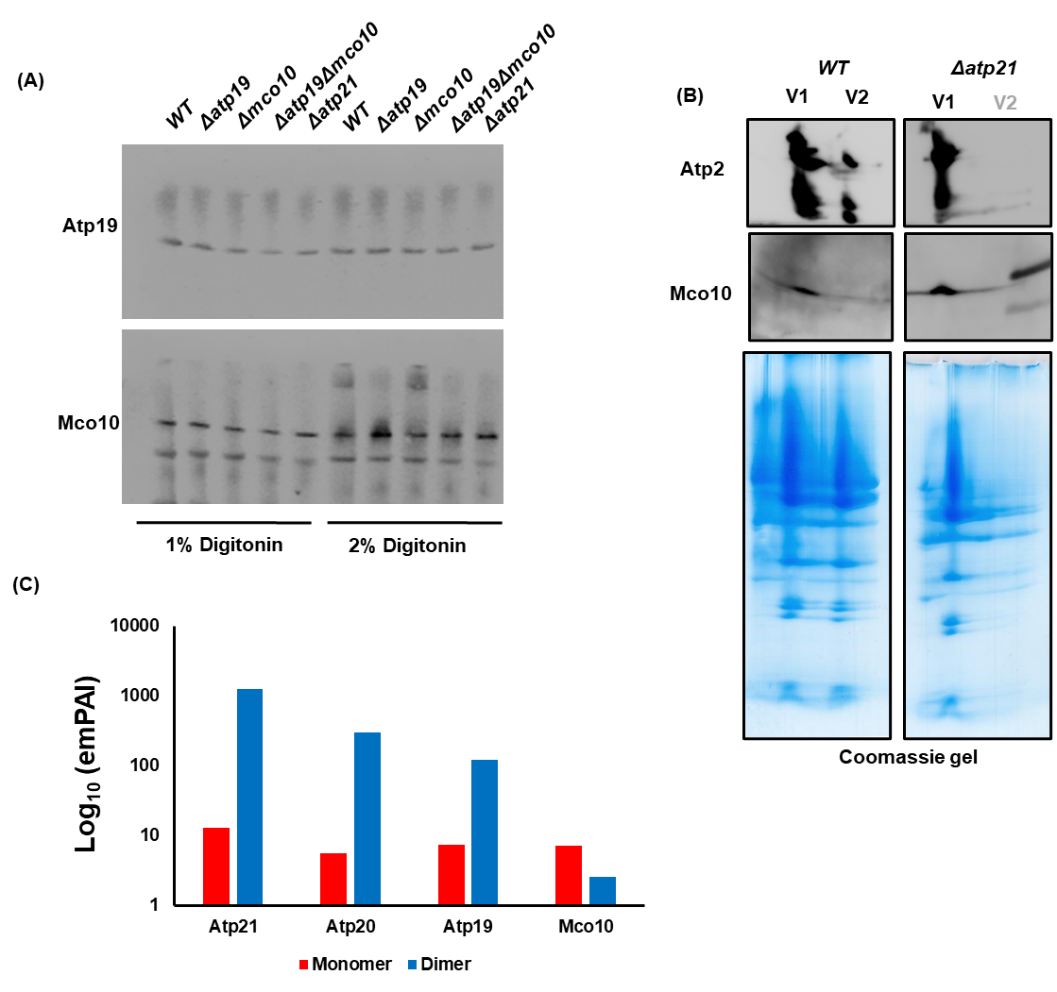

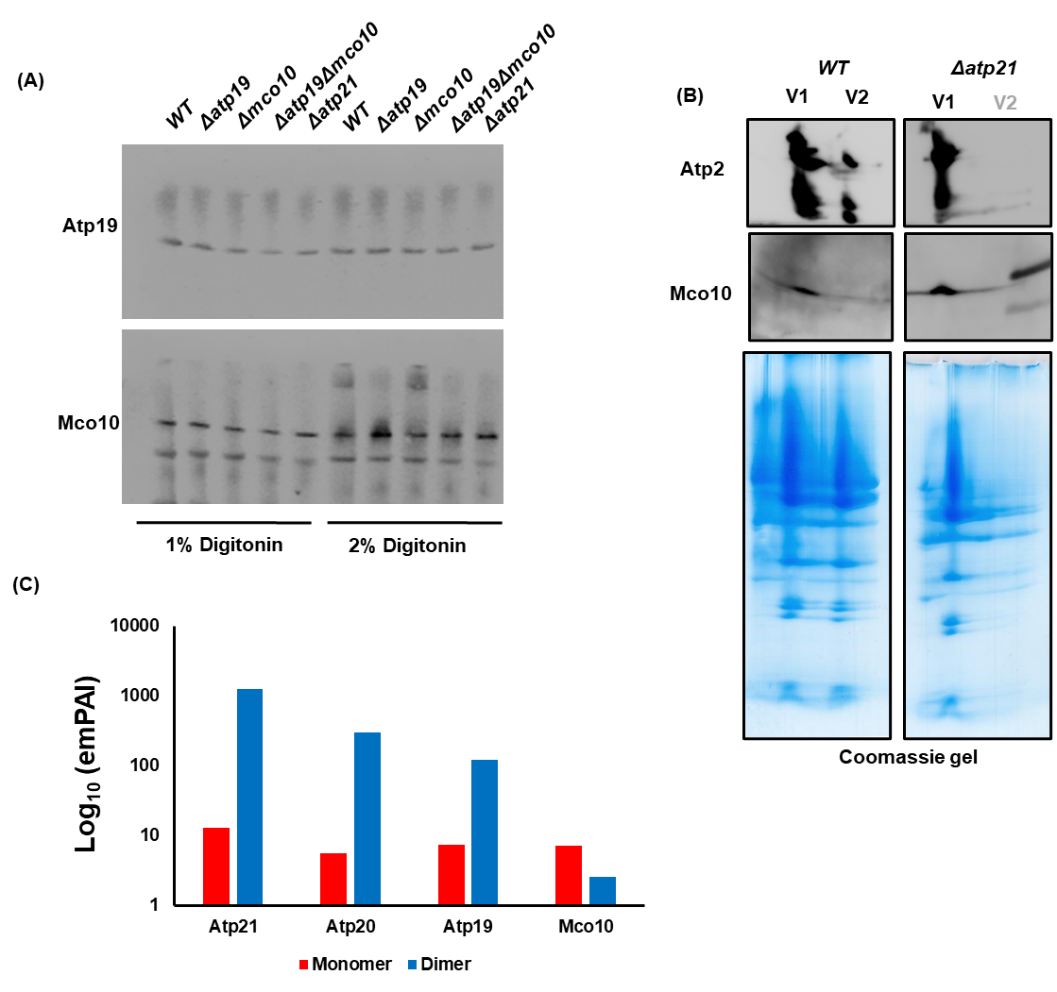


**(a)**

**(b)**


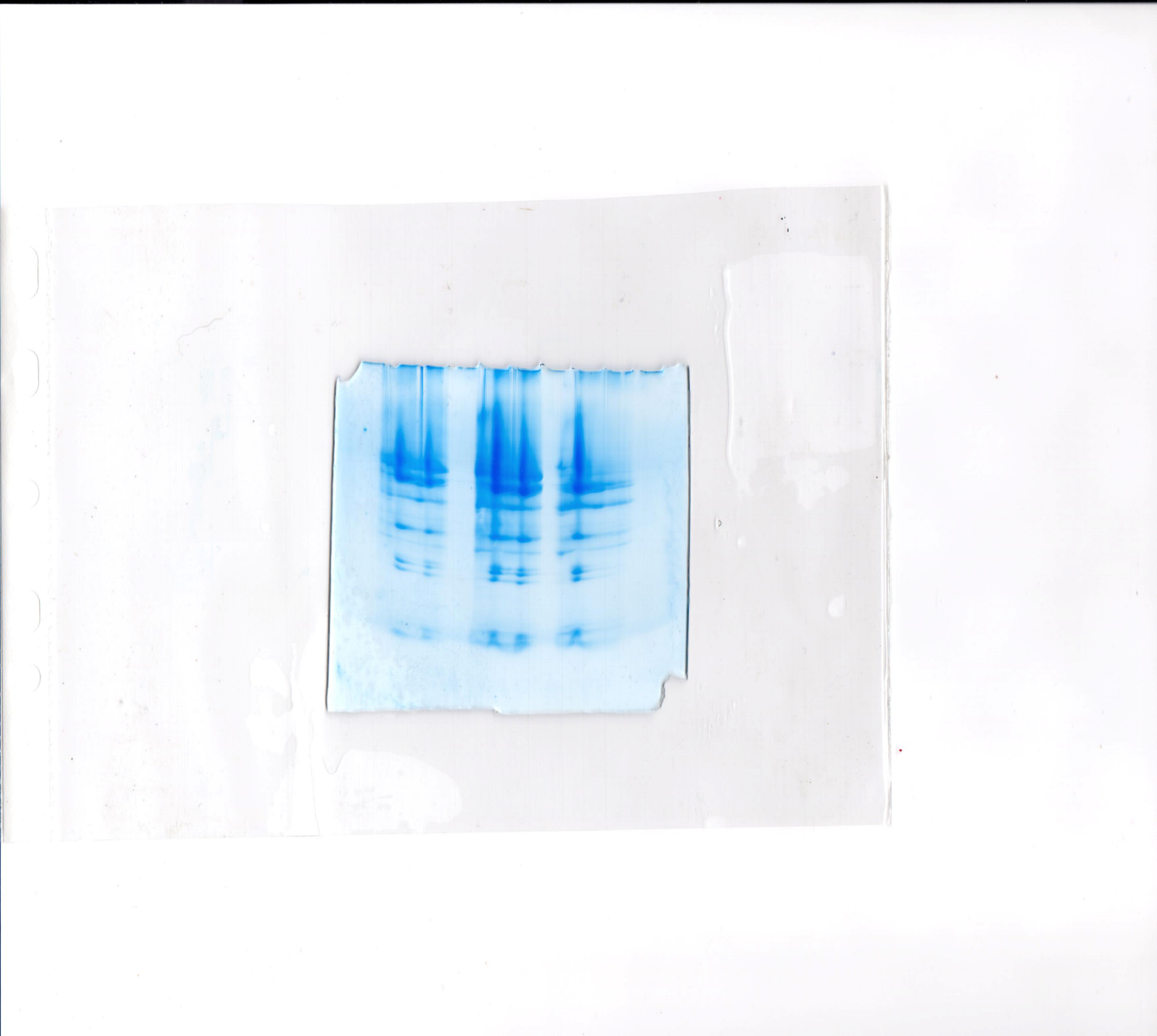

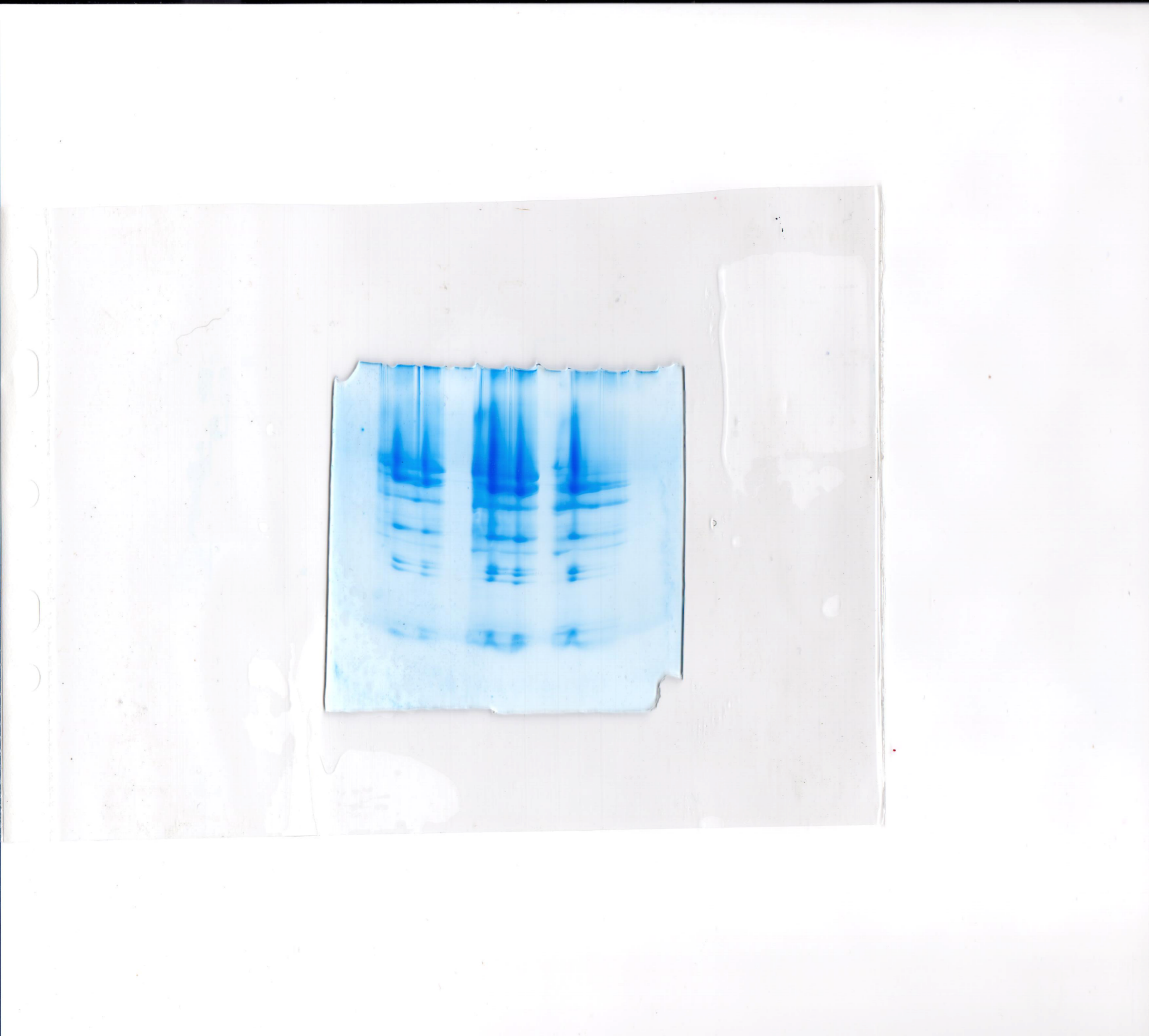


**(c)**

**Fig. S8.** Abundance of Atp19 and Mco10 in the monomers and dimers of ATP synthase. **a)** Mco10 and Atp19 antibody nonspecifically bind to monomer or dimer extracted by digitonin and separated in B-PAGE. **b)** Monomer and dimer subunits from the wild type and *Δatp21* from two-dimensional BN-SDS-PAGE gel separation were transferred to PVDF membrane and visualized by western blotting with respective antibodies. Mco10 is also detected in the monomer when Atp21 is deleted. **c)** Protein abundance index (emPAI) values of Atp19/*k*, Atp20/*g*, Atp21/*e* and Mco10 as determined from interactome analysis of the monomers and dimers of ATP synthase in *S. cerevisiae.* Related to Fig. 6. Original blots/gels are presented in SupplementaryRowImages page 13.


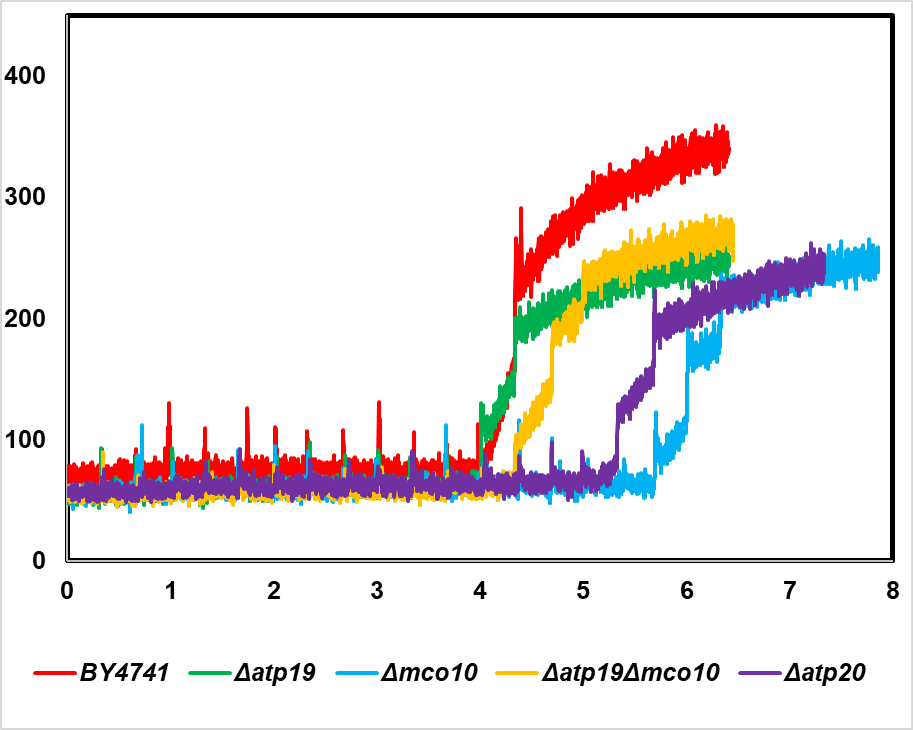


**Fluorescence intensity (A.U.)**

**Time (mins)**

**Fig. S9.** The PTP induction time measured in the CRC experiment. 1 mg of mitochondria was added to CRC buffer containing the calcium ionophore ETH129 and the calcium green-5N calcium indicator, and 10 µM CaCl_2_ was added every 20 seconds intervals until the mitochondria stops taking calcium and rapidly releases it into the buffer. Traces are representative of at least 3 independent experiments. Related to Fig. 7b.

**Supplementary Table Legends**

**Table S1. Small ≤ 20 kDa proteins interactome of ATP synthase in *S. cerevisiae*.** The proteins were identified from two different approaches using pulldown of ATP synthase by Atp6 tagged with HA-6His, or the monomers and dimers extracted by digitonin and separated in BN-PAGE or a further 2^nd^ dimensional SDS-PAGE identified by mass spectrometric analysis.

**Table S2. Small ≤ 20 kDa proteins interactome of ATP synthase in HEK293T cell line.** The monomers and dimers were extracted from isolated mitochondria from HEK293T cells by digitonin and separated in a 2^nd^ dimensional BN-SDS-PAGE. Gel pieces from molecular weight ≤ 20 kDa were cut-off and proteins were identified by mass spectrometry.

**Table S3.** Protein sequences of Atp19 and Mco10 homologs in different fungal genomes classified according to subphylum.

**Table S4.** Mascot search result of proteins identified in each band by LC/MS analysis from all experiments performed in this study. The details of bands excised from gels are present in Supplementary Fig. S1.
