# Supplementary figures and images for: ATP synthase interactome analysis identifies a new subunit l as a modulator of permeability transition pore in yeast
Source: Sci Rep. 2023 Mar 7;13:3839. doi: 10.1038/s41598-023-30966-5 (PMC9992712; doi:10.1038/s41598-023-30966-5)

Fig. 1a

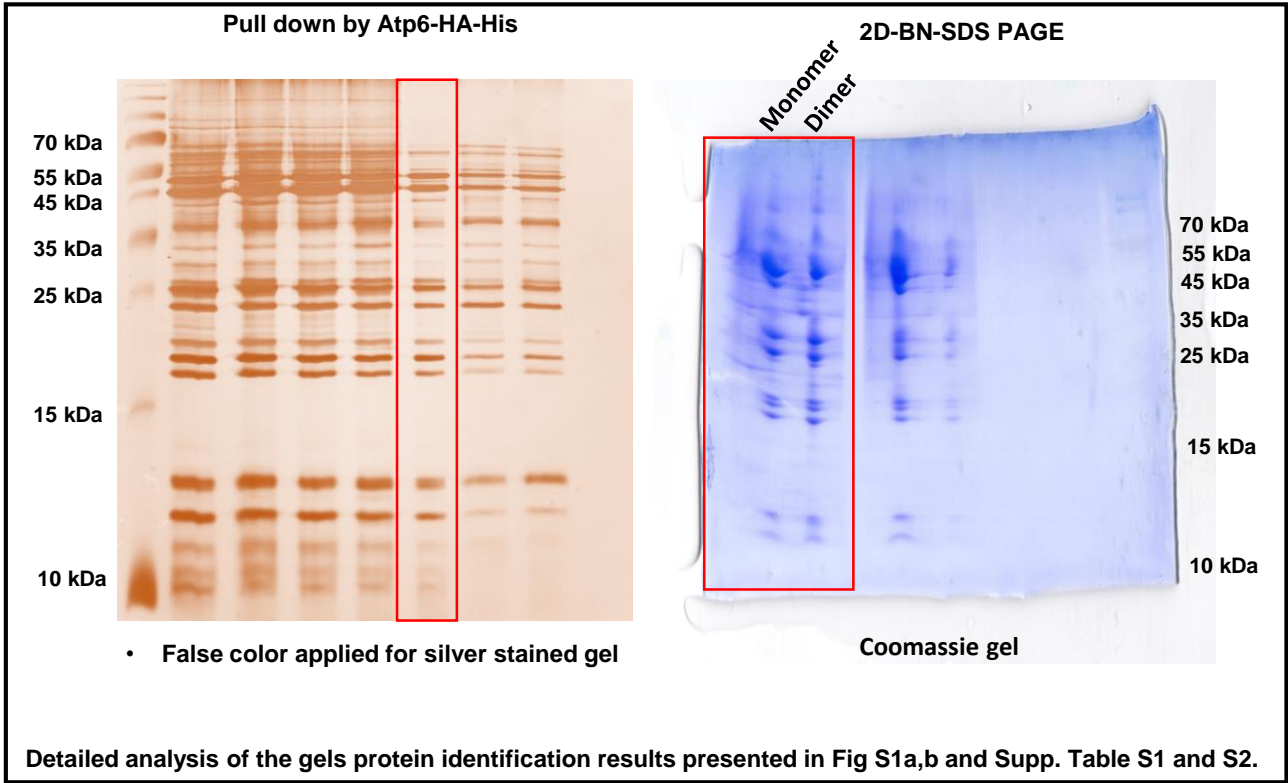

Figure 5a

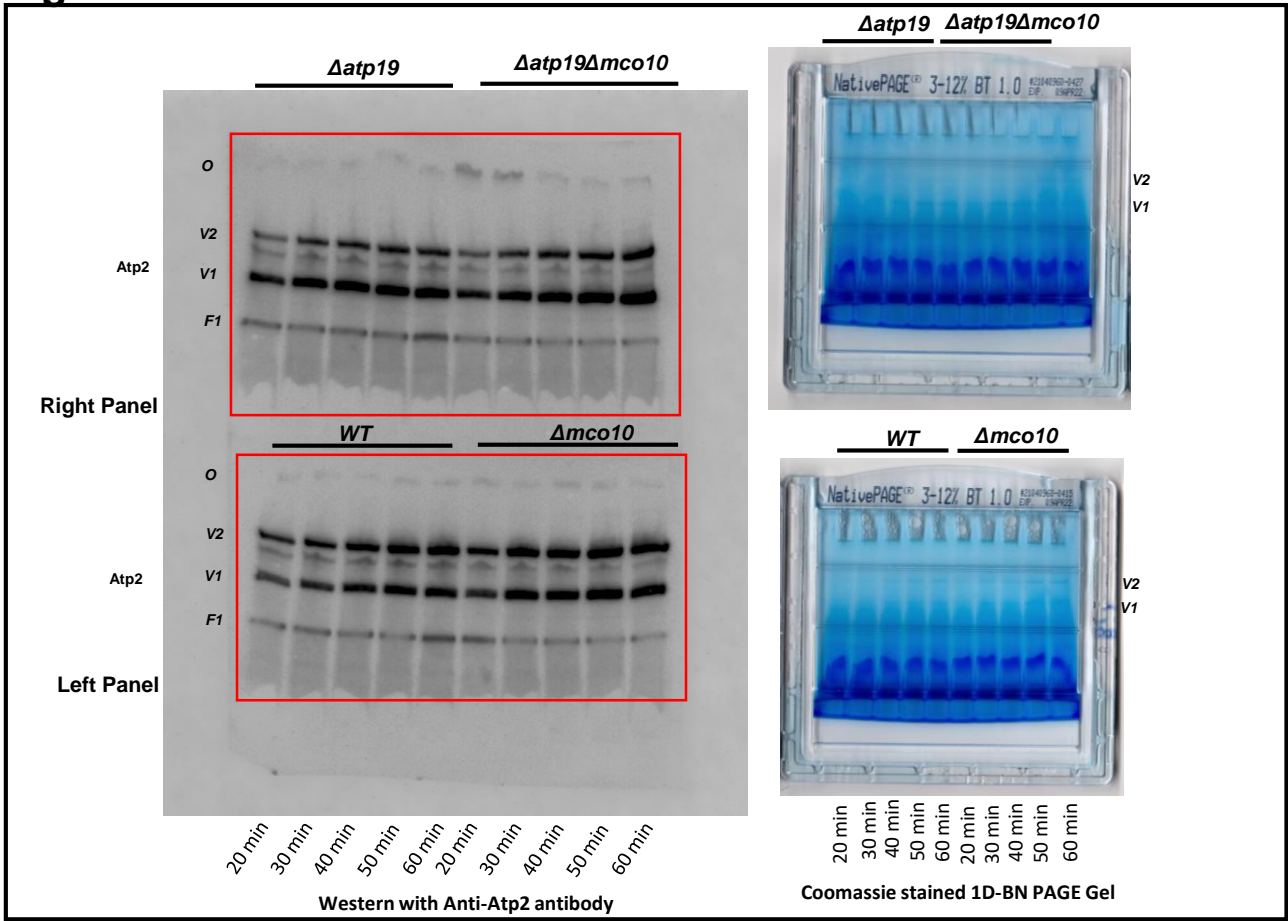

Fig. 5b

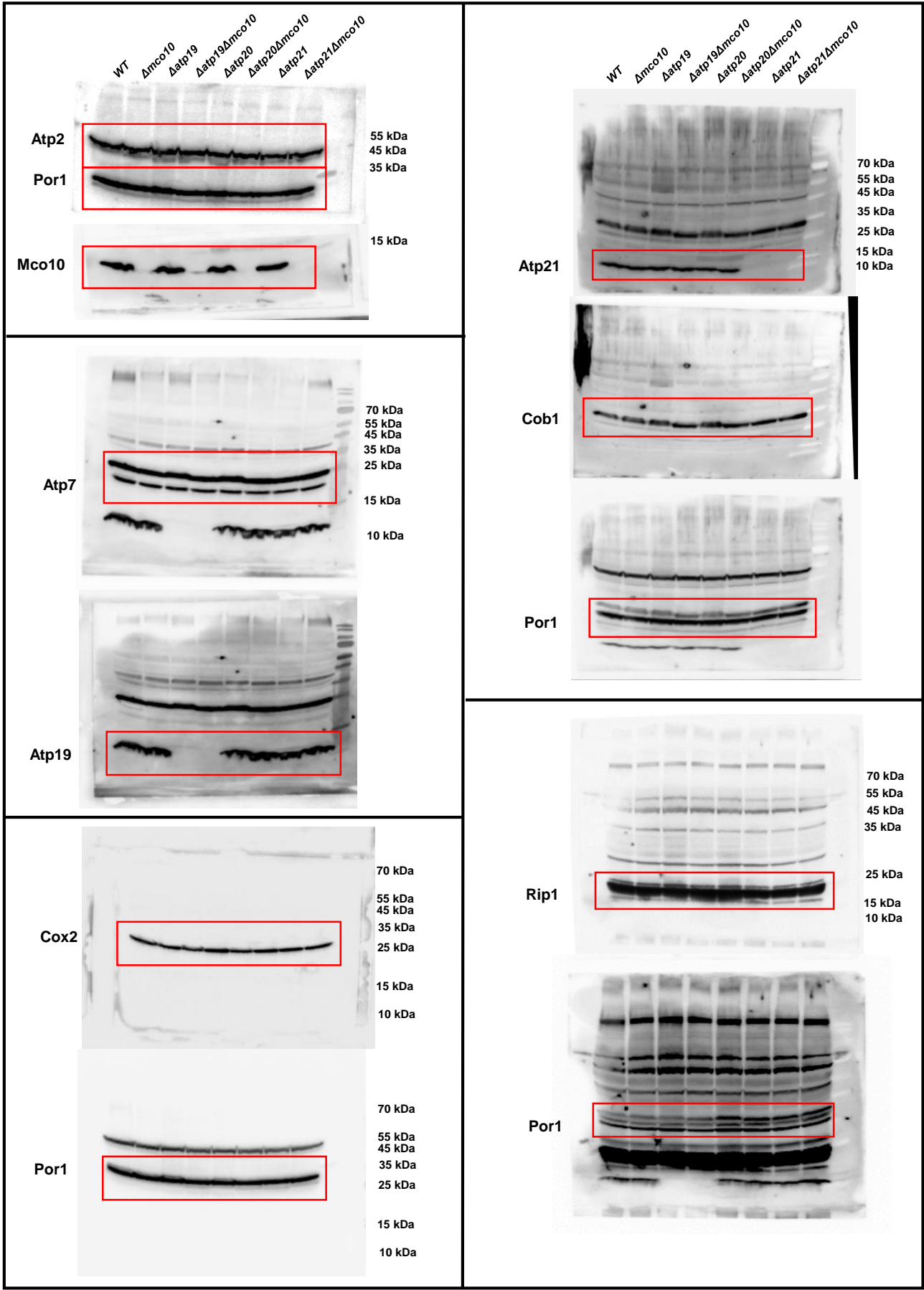

Fig. 5c

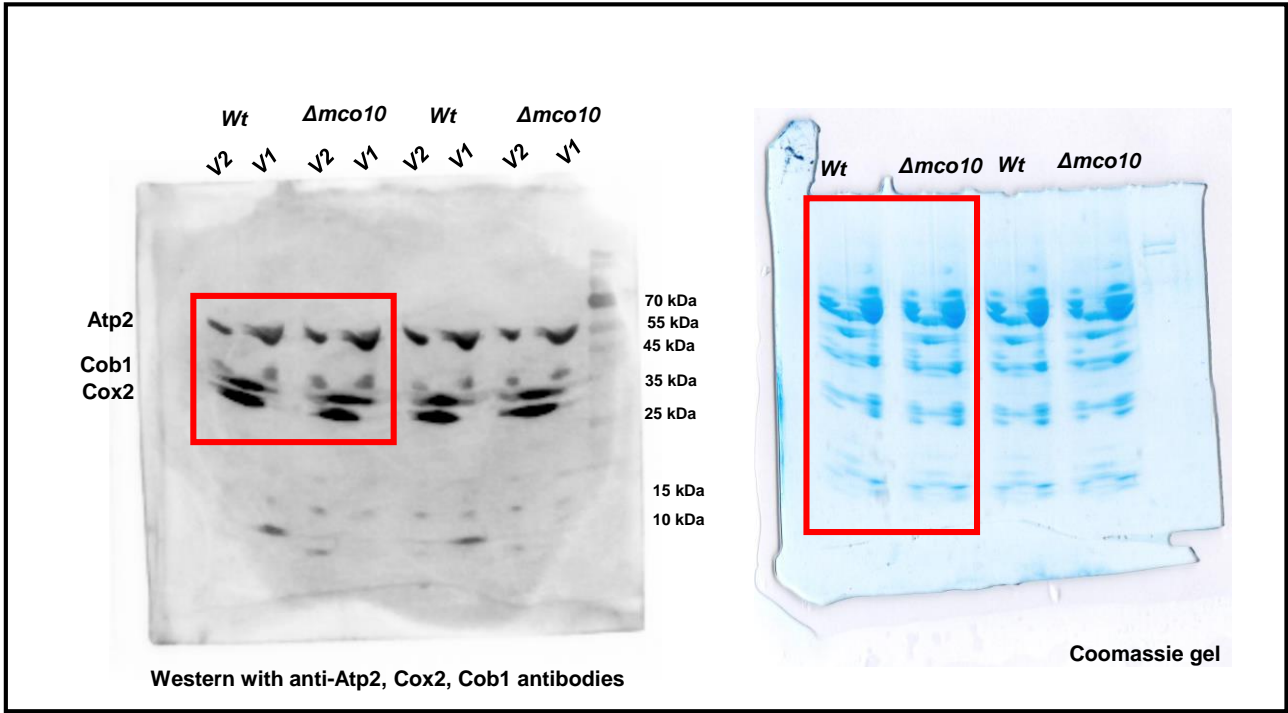

Fig. 6

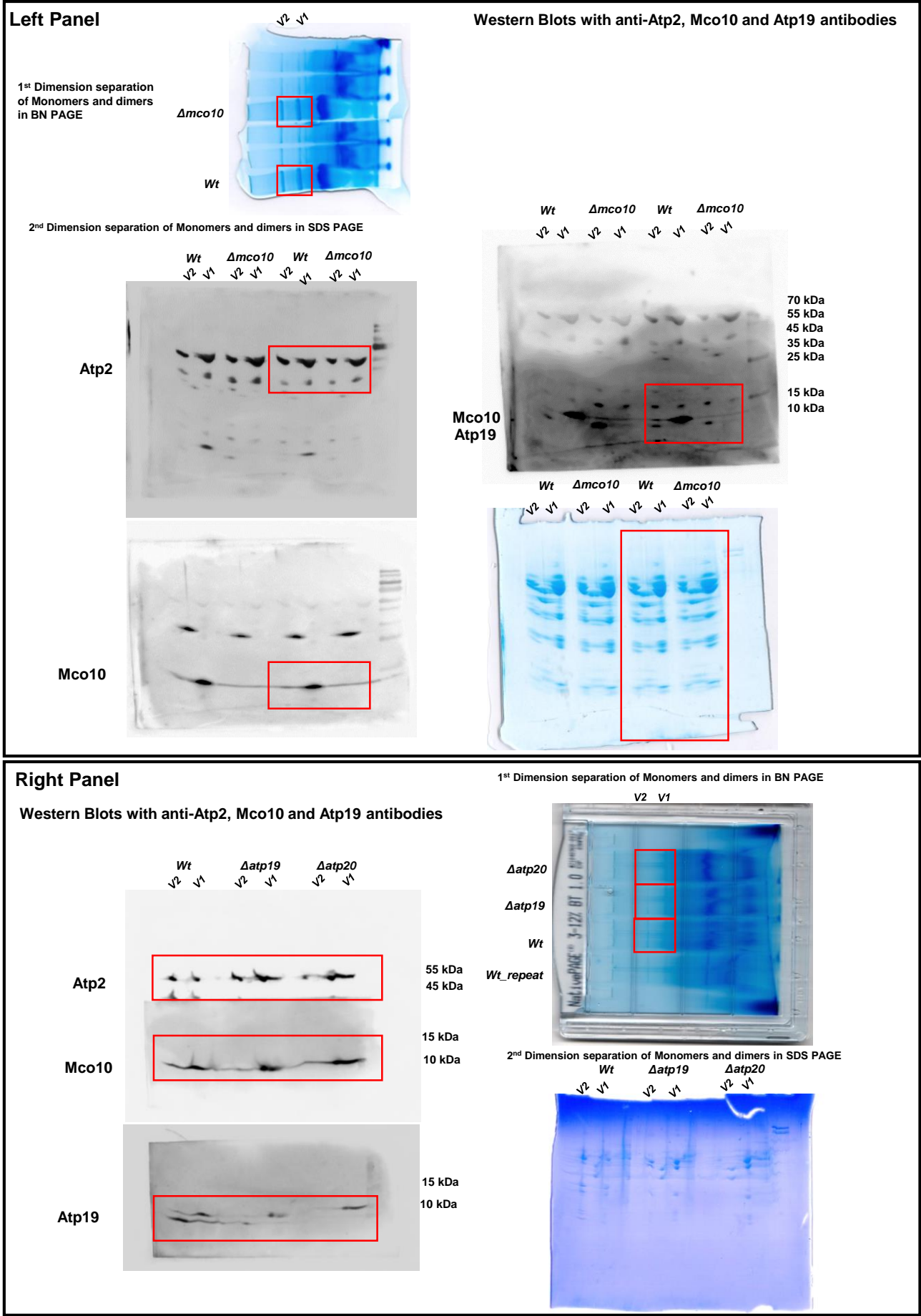

Fig. S1

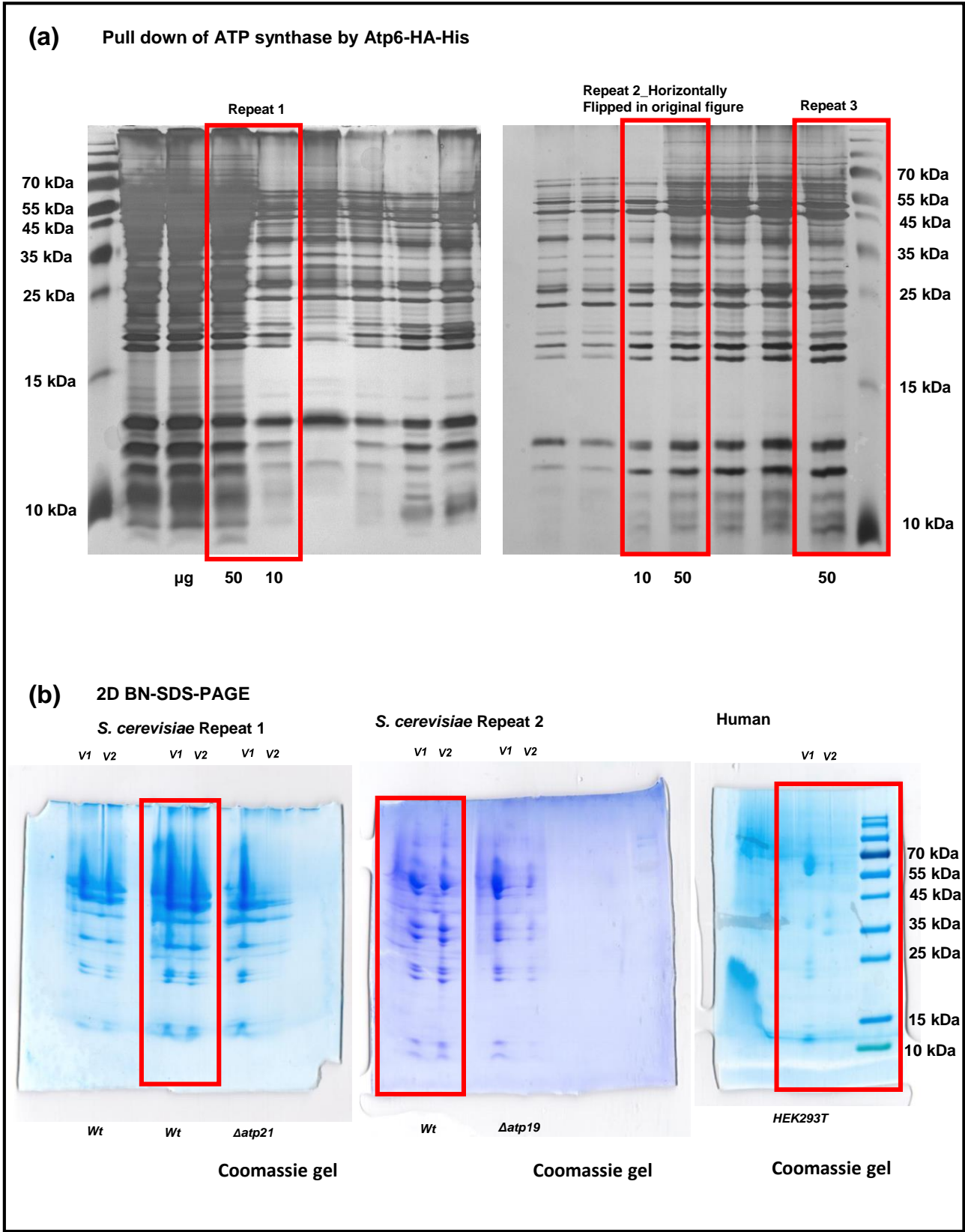

Fig. S6

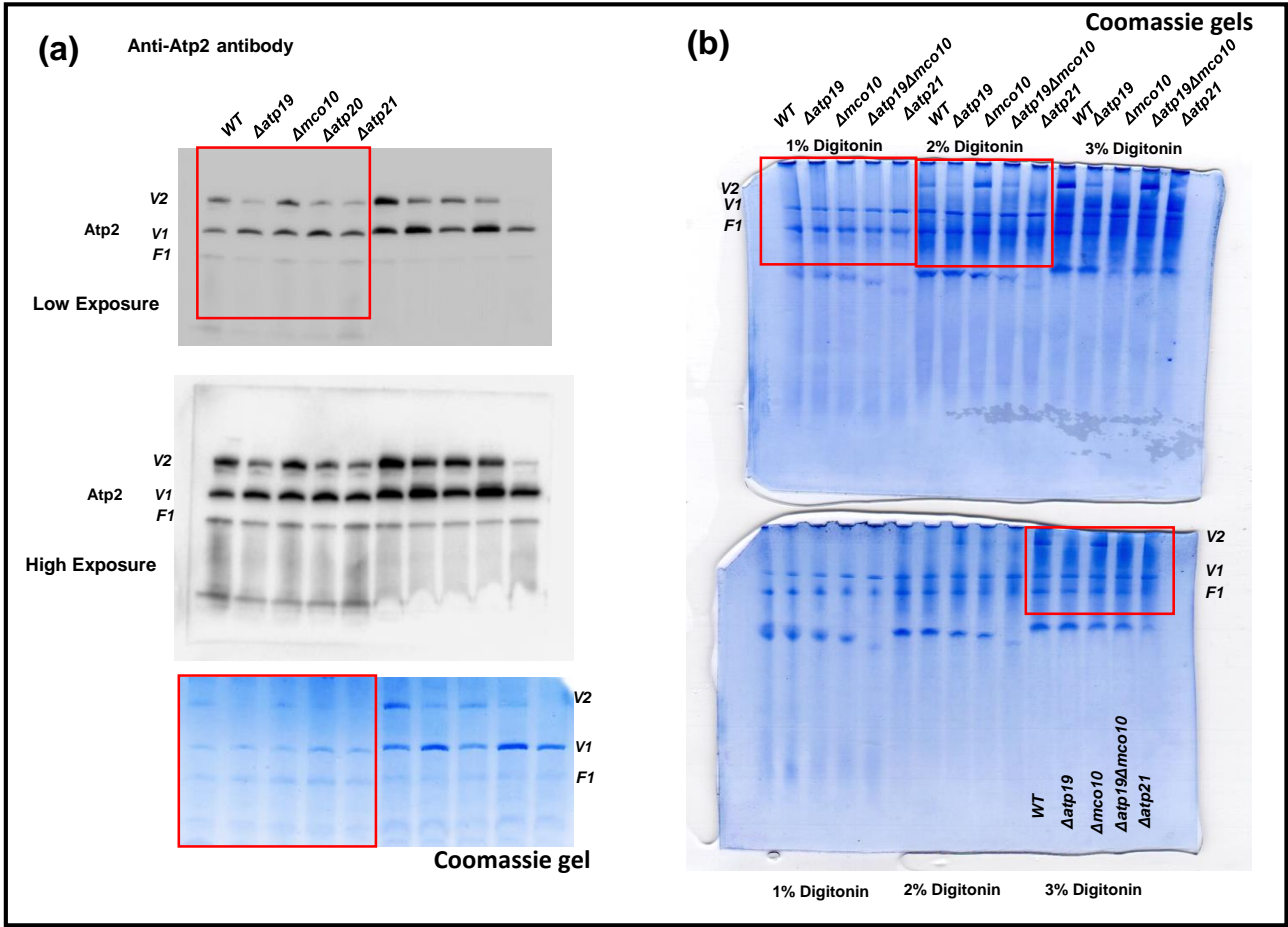

Fig. S7

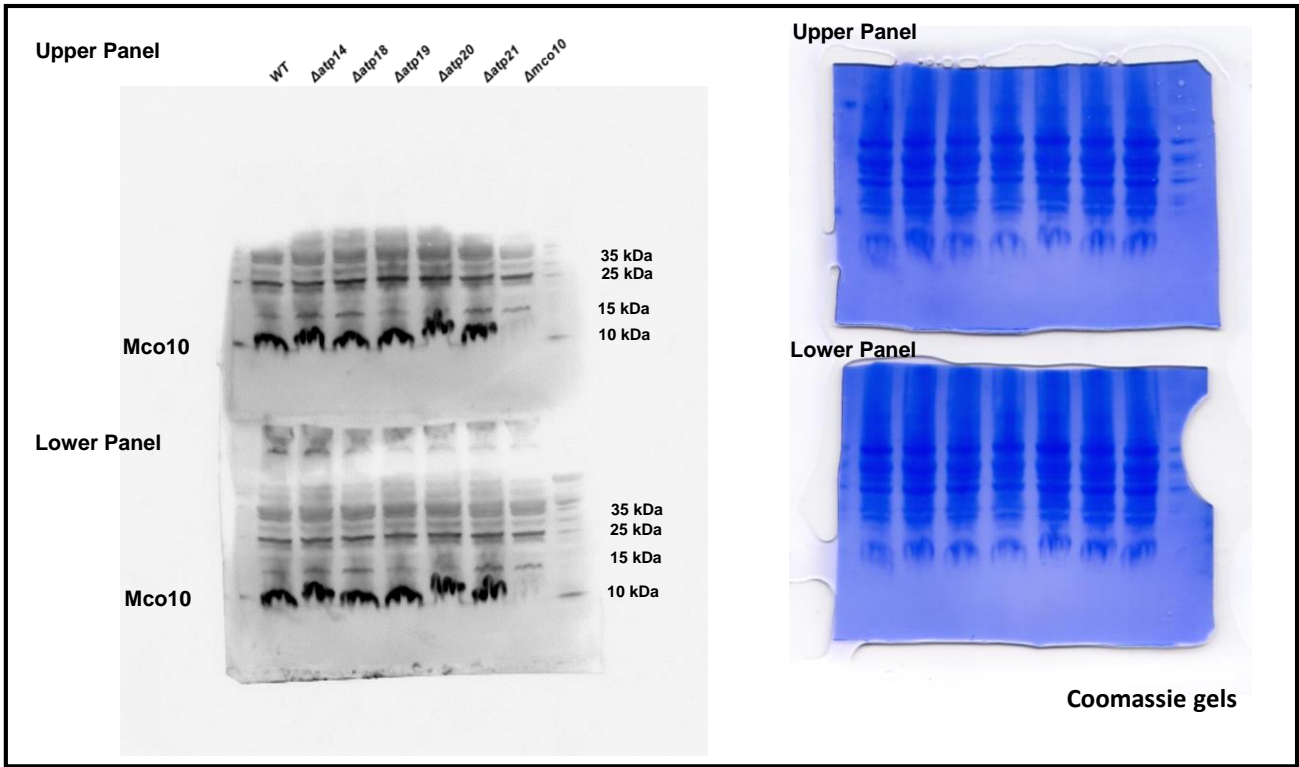

Fig. S8

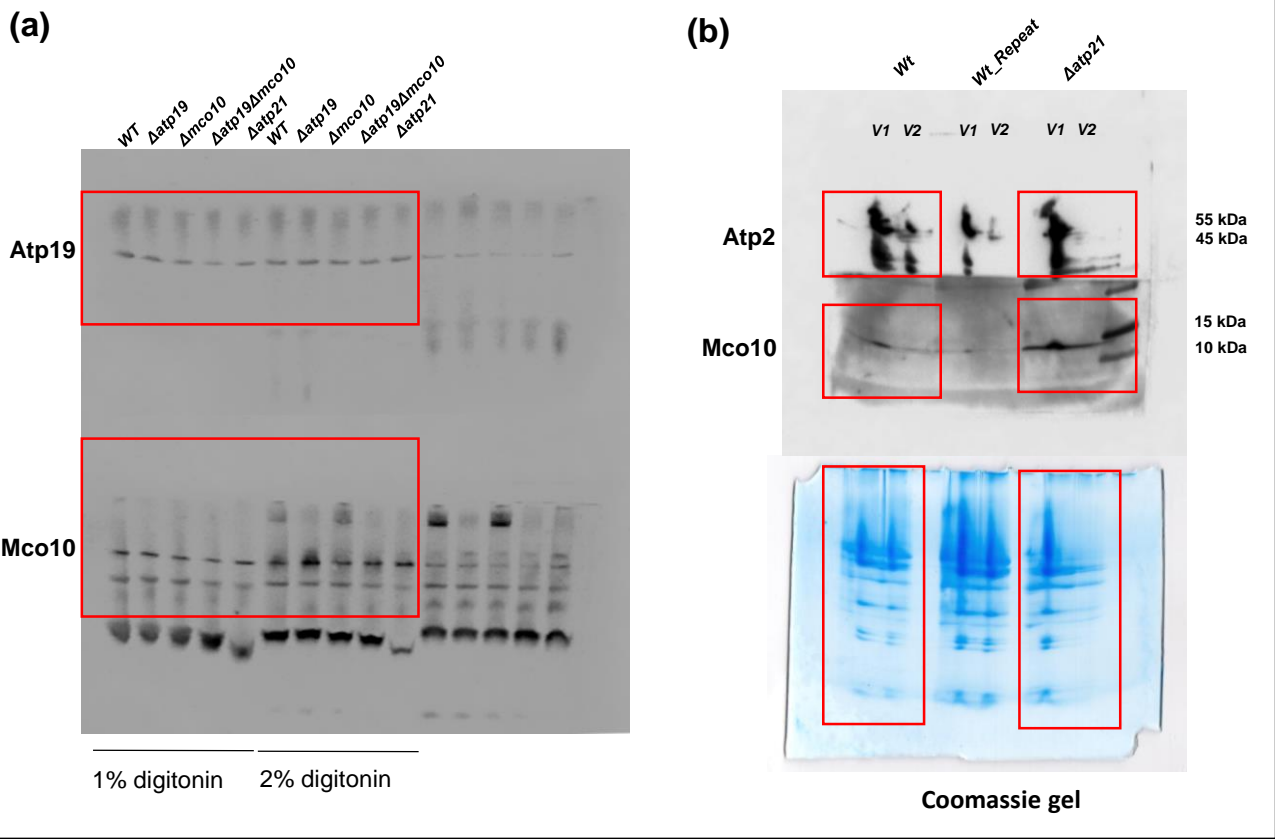

Supplement: Supplementary file 8 — Supplementary Information 8. [file 41598_2023_30966_MOESM8_ESM.pdf]
